# Copper coordination compounds with (5*Z*,5*Z*′)-2,2′-(alkane-α,ω-diyldiselenyl)-bis-5-(2-pyridylmethylene)-3,5-dihydro-4*H*-imidazol-4-ones. Comparison with sulfur analogue†

**DOI:** 10.1039/d1ra08995a

**Published:** 2022-03-02

**Authors:** Alexander V. Finko, Anatolii I. Sokolov, Dmitry A. Guk, Victor A. Tafeenko, Anna A. Moiseeva, Dmitry A. Skvortsov, Andrei A. Stomakhin, Andrei A. Beloglazkin, Roman S. Borisov, Vladimir I. Pergushov, Mikhail Ya. Melnikov, Nikolay V. Zyk, Alexander G. Majouga, Elena K. Beloglazkina

**Affiliations:** Moscow State University, Department of Chemistry Leninskie Gory, Building 1/3 Moscow 119991 Russia bel@org.chem.msu.ru; Topchiev Institute of Petrochemical Synthesis RAS Leninskii pr., 29 Moscow 119991 Russia; Higher School of Economics Myasnitskaya 13 Moscow 101000 Russia; Engelhardt Institute of Molecular Biology RAS Vavilova 32 Moscow 119991 Russia; National University of Science and Technology Leninskii pr., 4 Moscow 119049 Russia; Mendeleev University of Chemical Technology Miusskaya pl. 9 Moscow 125047 Russia

## Abstract

A series of new organic ligands (5*Z*,5*Z*′)-2,2′-(alkane-α,ω-diyldiselenyl)-bis-5-(2-pyridylmethylene)-3,5-dihydro-4*H*-imidazol-4-ones (L) consisting of two 5-(2-pyridylmethylene)-3,5-dihydro-4*H*-imidazol-4-one units linked with polymethylene chains of various lengths (*n* = 2–10, where *n* is the number of CH_2_ units) have been synthesized. The reactions of these ligands with CuCl_2_·2H_2_O and CuClO_4_·6H_2_O gave Cu^2+^ or Cu^1+^ containing mono- and binuclear complexes with Cu_2_LCl_*x*_ (*x* = 2–4) or CuL(ClO_4_)_*y*_ (*y* = 1, 2) composition. It was shown that the agents reducing Cu^2+^ to Cu^1+^ in the course of complex formation can be both a ligand and an organic solvent in which the reaction is carried out. This fundamentally distinguishes the selenium-containing ligands L from their previously described sulfur analogs, which by themselves are not capable of reducing Cu^2+^ during complexation under the same conditions. A higher cytotoxicity and reasonable selectivity to cancer cell lines for synthesized complexes of selenium-containing ligands was shown; unlike sulfur analogs, ligands L themselves demonstrate a high cytotoxicity, comparable in some cases to the toxicity of copper-containing complexes.

## Abstract 2

Mono- and binuclear Cu(+1/+2) complexes of bis-5-(2-pyridyimethylene)-2-selenohydantoins were obtained by the reactions of corresponding ligands with copper(+2) chloride or perchlorate in BuOH/DCM mixtures.

## Graphical abstract

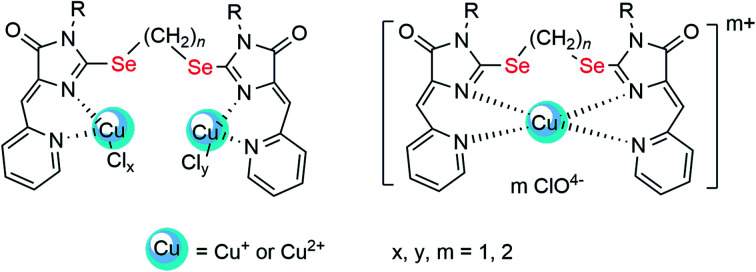

## 1. Introduction

2-Chalcogen-imidazolones (hydantoins and analogs) and their derivatives are attracting attention due to the wide range of their biological activity, including cytotoxicity.^1–9^ For example, enzalutamide (4-[3-[4-cyano-3-(trifluoromethyl)phenyl]-5,5-dimethyl-4-oxo-2-sulfanylideneimidazolidin-1-yl]-2-fluoro-*N*-methylbenzamide) is an antiandrogenic anticancer drug;^10^ spiro-derivatives of hydantoins and thiohydantoins have been shown to induce apoptosis in cancer cells by inhibiting the interaction of MDM2-p53 proteins;^11–14^ copper- and cobalt-containing complexes of *S*-alkylated thiohydantoins are effective antineoplastic agents with various mechanisms of action.^15–18^

Tetradentate ligands of the 2,2′-(alkane-α,ω-diyldisulfanyl)-bis-5-(2-pyridylmethylene)-3,5-dihydro-4*H*-imidazol-4-one series in the reactions with CuCl_2_ form coordination compounds containing Cu^2+^ or Cu^1+^, depending on the substituents at the nitrogen atoms N(3) of the ligand thioimidazolone fragments.^15–19^ Wherein the reduction of Cu^2+^ to Cu^1+^ in the process of complexation occurs exclusively under the action of solvents (alcohols), and the organic ligand does not participate in the redox reaction.

**Fig. 1:**
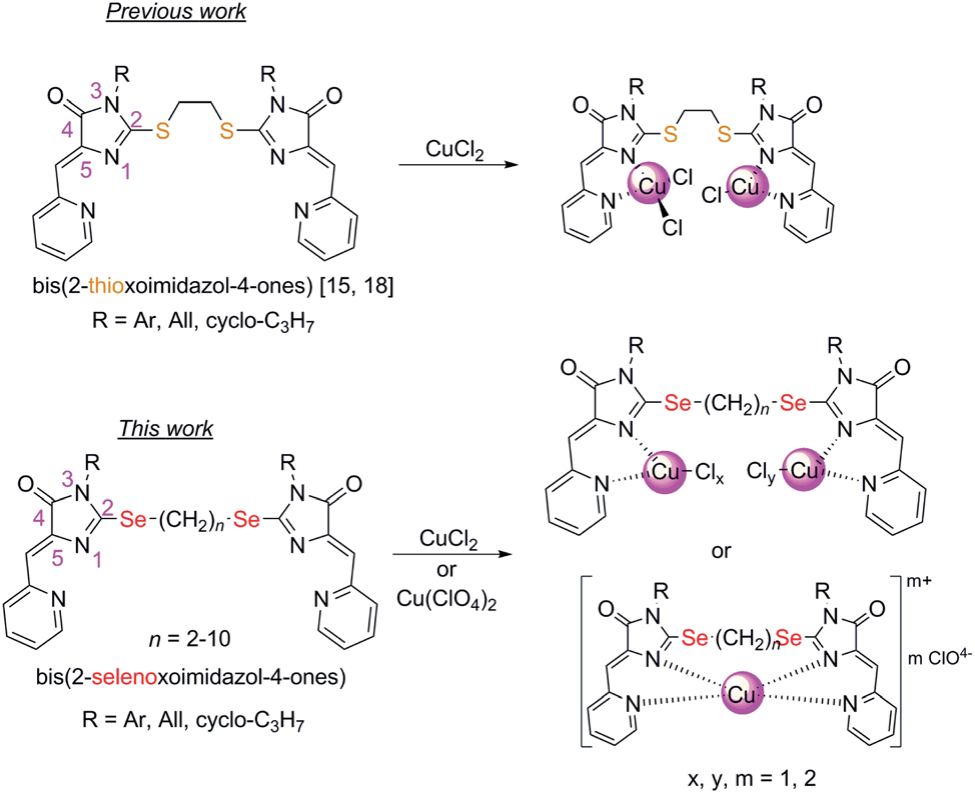
Compounds described previously^15,18^ and in this work.

In this work, we investigated the possibility to obtain copper-containing coordination compounds with the ligands 4 (Fig. 1), containing two 5-(2-pyridylmethylene)-2-selenoxo-imidazole-4-one fragments linked by polymethylene linkers of different lengths; they are selenium-containing analogues of the aforementioned 2-thioimidazolones. We assumed that the replacement of sulfur by selenium in the structure of the organic ligand should stabilize complexes containing copper in a lower oxidation state (+1), due to the higher donor ability of selenium compared to sulfur, and may also lead to a change in the mechanism of copper reduction in the complexation process.

Complexes of copper with organic ligands may be potentially of interest as anticancer drugs to replace highly toxic platinum derivatives in clinical practice.^20–24^ Copper stabilization of in the +1 oxidation state is important for the cytotoxic activity of the complexes, since Cu effectively penetrates into the cell only in this oxidation state.^25–29^ Besides, selective absorption of selenium is observed in some tumor cells, which can potentially lead to an increase in the antitumor activity of selenium-containing compounds in comparison with their sulfur analogues.^30–33^

## 2. Results and discussion

### 2.1. Synthesis of Se-containing ligands

**Scheme 1:**
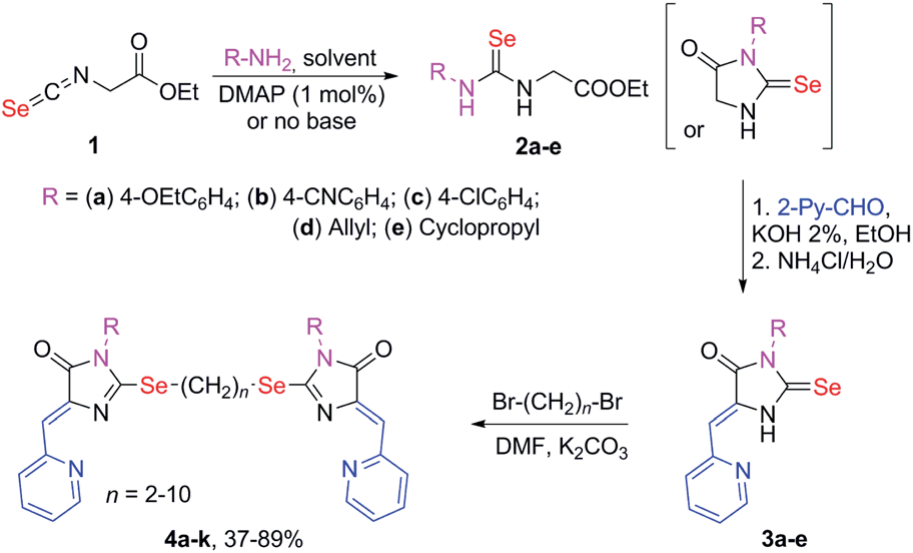
Synthesis of ligands 4a-k.

Selenium-containing ligands 4a-k were obtained by the reaction sequence shown in Scheme 1, starting from ethyl isoselenocyanatoacetate and the corresponding alkyl or aryl amines. Compounds 1, 2a-d and 3c,d have been previously described;^34,35^ compounds 3a and 3b were synthesized in the same way (see ESI†).

**Scheme 2:**
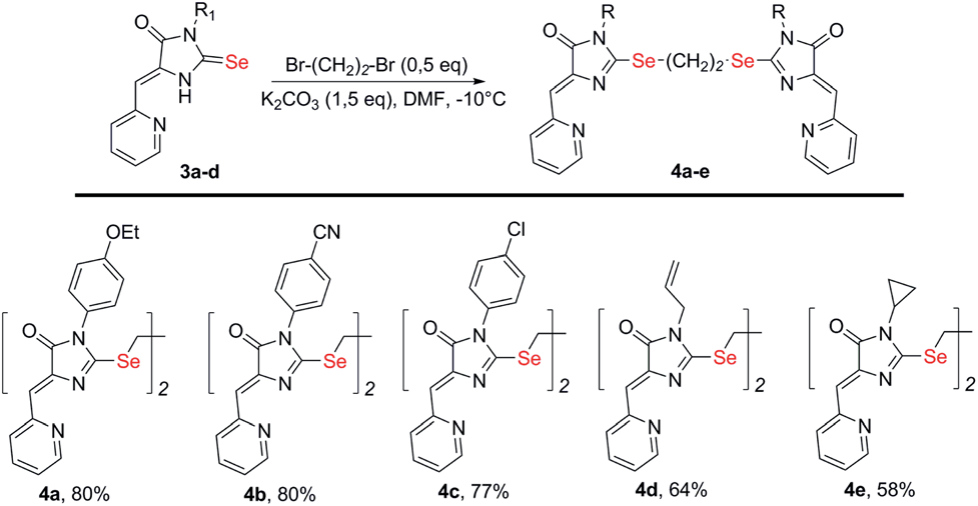
Synthesis of bis-5-pyridylmethylene-2-selenohydantoins 4a-e with the (CH_2_)_2_ linker between selenium atoms.

**Scheme 3:**
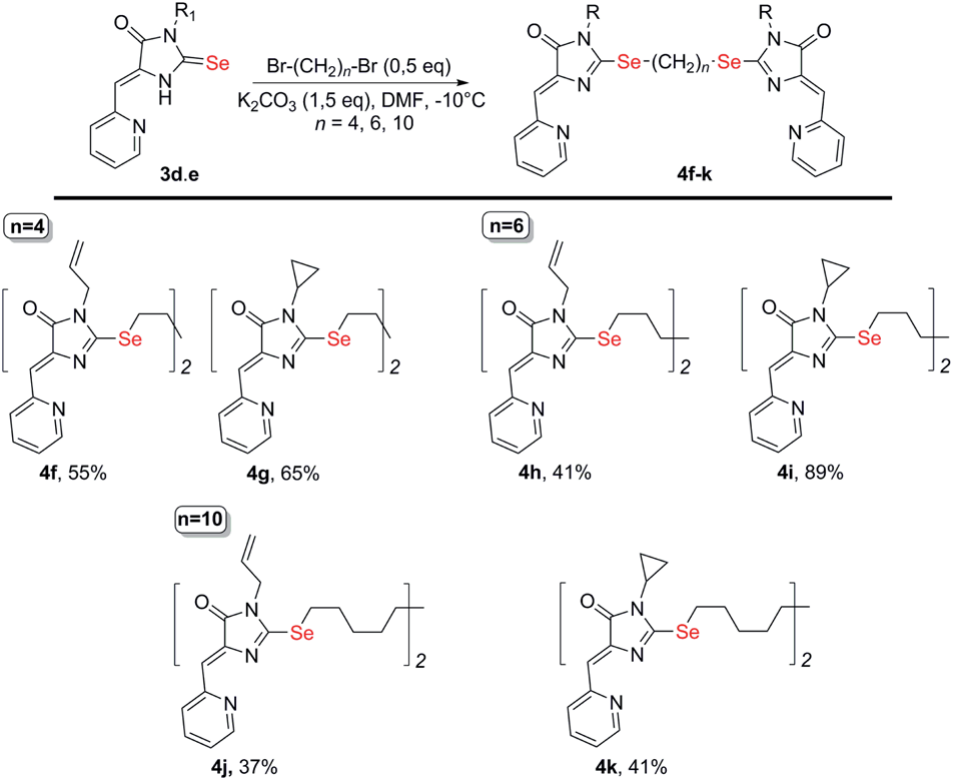
Synthesis of bis-5-pyridylmethylene-2-selenohydantoins 4e-j with the (CH_2_)_*n*_ (*n* = 4–10) linkers between selenium atoms.

To obtain ligands 4a-k, compounds 3a-e were alkylated with α,ω-dibromoalkanes in the presence of excess potassium carbonate in DMF (Schemes 2 and 3). When optimizing the reaction conditions, cesium carbonate was also tested as a base, and DMSO was used as a solvent. The difference in the target products yields when using Cs_2_CO_3_ or K_2_CO_3_ was insignificant, and we preferred the latter due to its lower cost. In DMSO, the quantity of the monosubstitution product in the starting dibromoalkane increased, and a significant tarring of the reaction mixture was observed.

Ligands with a two-carbon linker between selenium atoms (compounds 4a-e, Scheme 2) contained substituents of different nature at the N(3) atom of the imidazolone fragment (allyl, cyclopropyl, aromatic with donor (OEt) or acceptor (CN, Cl) substituents). For ligands with N(3)-allyl and cyclopropyl substituents, the length of the polymethylene linker was varied (2, 4, 6, and 10 carbon atoms between selenium atoms – compounds 4d-k, Schemes 2 and 3).

**Fig. 2:**
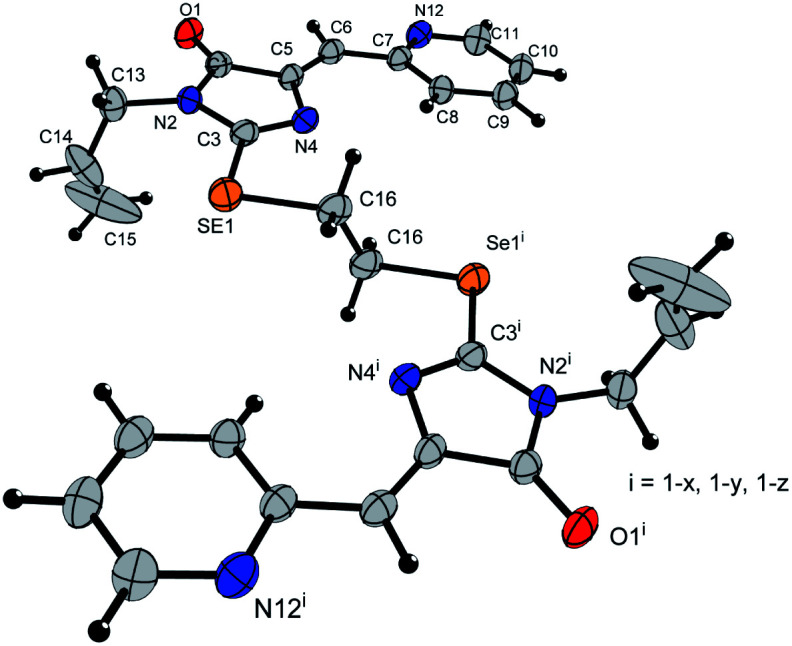
Molecular structure of compound 4d (CCDC 2017202). Thermal ellipsoids are shown with 30% probability.

Ligands 4a-k were characterized by ^1^H and ^13^C NMR spectroscopy, FTIR and HRMS data; the structure of compound 4d was additionally confirmed by X-ray data (Fig. 2). The molecule 4d has an inversion center located in the middle of the C16–C16′ bond. The imidazolone fragments of the ligand are almost flat, and conjugated pyridine rings are near coplanar to the imidazolone cycles, as was earlier observed for structurally similar sulfur-containing ligands.^36^ The pyridine nitrogen atoms are in the *anti*-position with respect to the nitrogen atom of the neighboring imidazolone rings, which is probably due to the repulsion of the electron pairs of the N4–N12 and N4′–N12′ atoms of the imidazolone and pyridine rings in the case of *syn*-conformation of these atoms.

### 2.2. Synthesis of coordination compounds

**Scheme 4:**
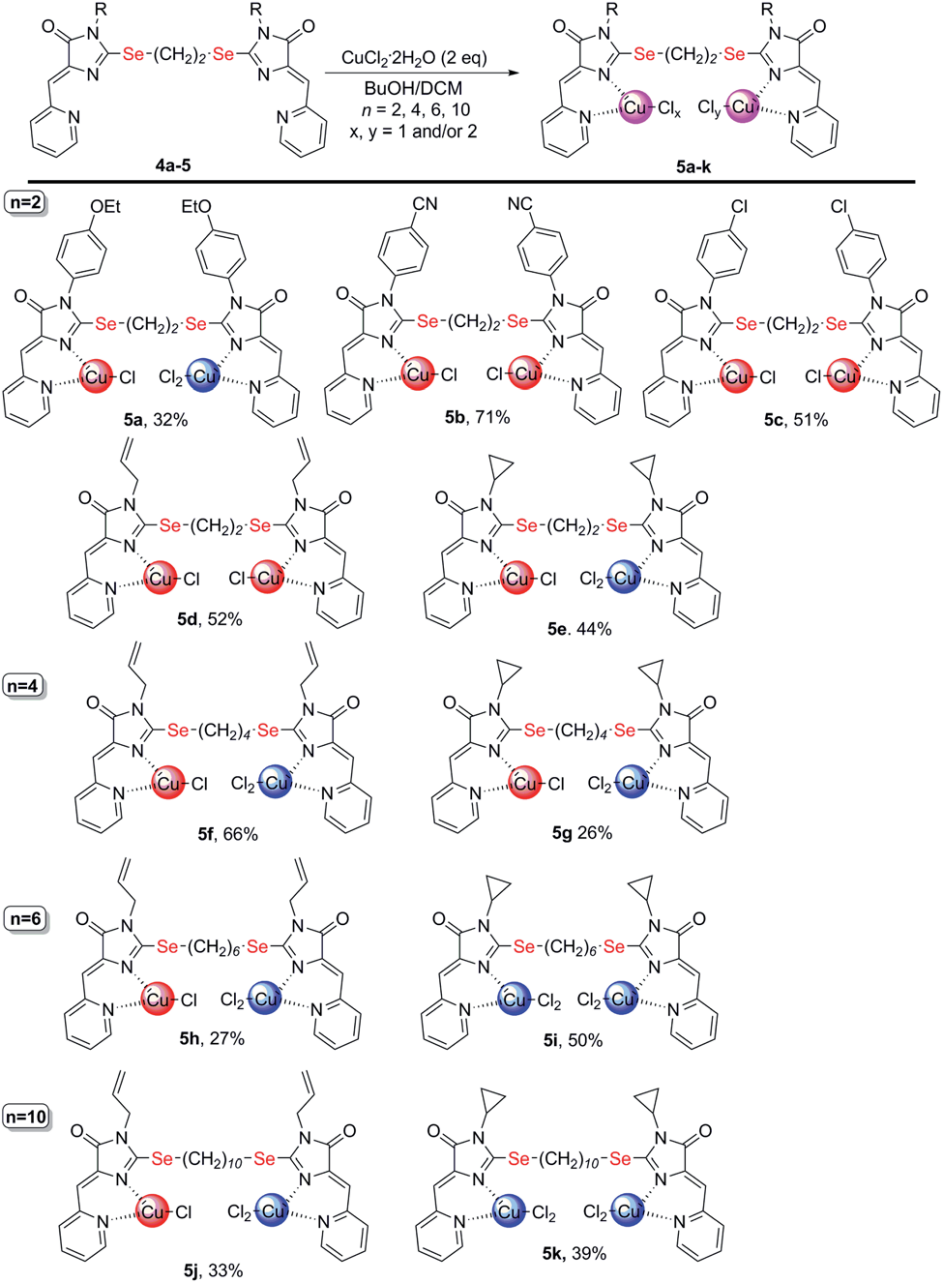
Coordination compounds 5a-k formed in the reactions of ligands 4 with CuCl_2_·2H_2_O. The red circles represent Cu^1+^, the blue circles represent Cu^2+^. The structures of the main reaction products are given, see also Table S1† and discussion in the text.

Copper-containing coordination compounds of ligands 4 were obtained by slow diffusion of a solution of a metal salt in *n*-butanol into a solution of a ligand in methylene chloride at a ligand/copper salt ratio of 1 : 2. The copper source was CuCl_2_·2H_2_O and Cu(ClO_4_)_2_·6H_2_O (Schemes 4 and 7). The structures of the obtained complexes 5, 6 were established based on the data of mass spectrometry (MALDI and HRMS), FTIR and electron spectroscopy, as well as X-ray analysis. The oxidation state of copper in the complexes was confirmed by electrochemical studies. For some coordination compounds, the composition was also confirmed by elemental analysis data; in cases of the formation of mixtures of coordination compounds with different oxidation states of copper (see below), elemental analysis did not give adequate results and its results are not presented.

#### 2.2.1 Reactions with copper(ii) chloride

**Fig. 3:**
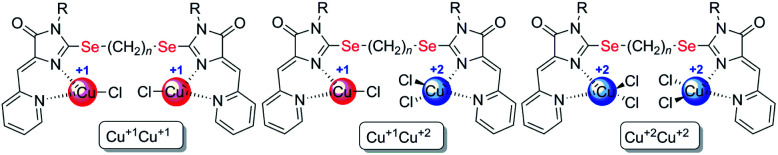
Structural types of complexes formed during the interaction of ligands 4 with CuCl_2_·2H_2_O.

It was found that the reactions of copper(ii) chloride with ligands 4 leads to binuclear coordination compounds 5a-k of three different structural types containing Cu^1+^ and Cu^2+^ ions in various combinations – Cu^1+^Cu^1+^, Cu^1+^Cu^2+^ or Cu^2+^Cu^2+^ (Fig. 3 and Scheme 4).

In the FTIR spectra of complexes 5 (see ESI†), the vibration bands of the cross-conjugated system of C=N, C=O, and C=C bonds at 1550–1750 cm^−1^ shift to longer wavelengths compared to the initial ligands, which confirms the participation of this system in coordination of copper ions.

**Fig. 4:**
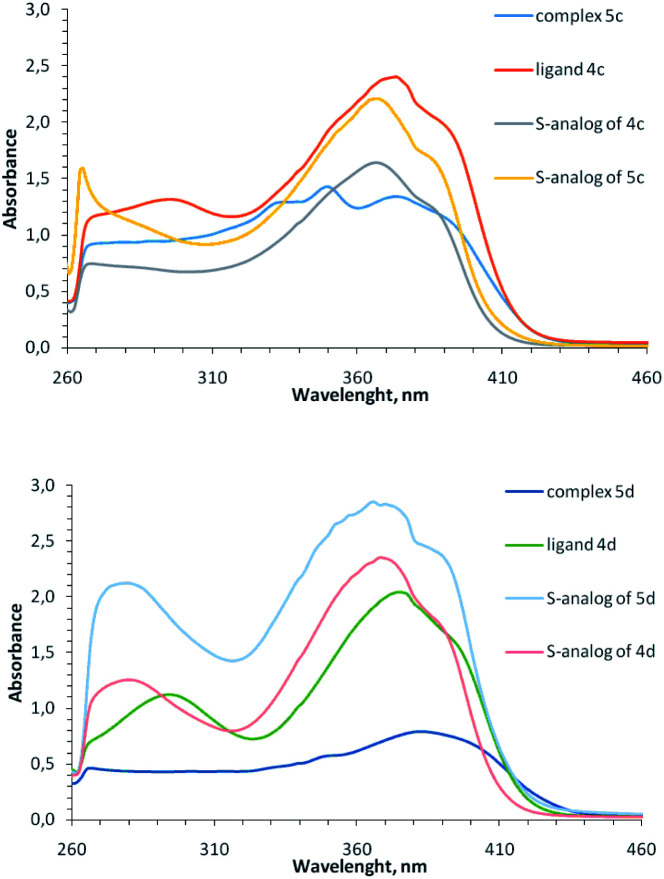
Electronic spectra of ligand 4c, 4d and their coordination compounds 5c, 5d in comparison with similar S-containing ligands and complexes (DMF, 5 × 10^−4^ M, 0.1 M).

In the UV-vis spectra of complexes 5 (concentration 10^−4^ to 5 × 10^−4^ M) there are intense bands of intra-ligand transitions in the region of 260–420 nm, which are similar to the bands of free ligands (Fig. 4); any absorption bands of noticeable intensity are absent in the visible region and the observed reddish-brown color of coordination compounds 5 (from dark brown for Cu^2+^ containing complexes 5i, 5k to dark-red for Cu^1+^ containing complexes 5b-5d) apparently, is due to the presence of broad electronic absorption bands in the UV region (Fig. S25–S31†). When recording UV-vis spectra of complexes 5 at concentration 10^−3^ to 2 × 10^−3^ M, low-intensity bands at 500–600 nm appear for the complexes containing both Cu^2+^ and Cu^1+^ (Fig. S31†), but these bands are absent for homovalent copper complexes, similar to previously observed for copper-containing coordination compounds with the analogous sulfur-containing ligands.^18^

Note that although Cu^1+^-containing complexes are typically colorless and selenium-containing ligands 4 are yellow, the copper(+1)-containing coordination compounds of the ligands 4 are colored red, similar to previously described monovalent copper complexes with 2-methylthio-5-pyridylmethylene-3-aryl-imidazol-4-ones.^15,37^ Apparently, the red color of the complexes is these cases is due to the strong ligands absorption band from the UV region, undergo bathochromic shift at complexation of the corresponding ligands with Cu^1+^.

Comparison of absorption spectra of selenium-containing complexes with their previously described sulfur analogs^15,18,37^ shows an interesting difference: for 2-thioimidazolone ligands, the intensity of bands at 260–420 nm increases upon complexation, while for 2-selenoimidazolone ligands, on the contrary, decreases. This may be due to the different electron density distribution between the ligand and the copper ion for S- and Se-containing complexes. In both cases, a ligand can exhibit two properties when interacting with a metal ion: it could be σ-donor due to the electrons transfer to copper from lone electron pairs of pyridine and imidazolone nitrogen atoms, and it could be π-acceptor due to the back-donation from metal to ligand. It may be assumed that in the case of S-containing complexes, the acceptor effect of the ligand predominates, and in the case of complexes with a less electronegative Se atom, on the contrary, the donor effect is manifested.

In the mass spectra of complexes 5 with different ionization methods (ESI, MALDI using different matrices), we was not observe the peaks of molecular ions, and the peaks with the highest intensity correspond to the ions with [4Cu]^+^ compositions (see ESI†). The peaks of [4CuCl]^+^ ions were also observed in most cases. In MALDI spectra of complexes 5a-e with a two-carbon bridge between selenium atoms, there were also the peaks of ions with the [(4-C_2_Н_4_)Cu]^+^ and [4-C_2_Н_4_]^+^composition; apparently, these ions were formed upon the loss of ethylene molecules and further copper chloride by metal complexes. Since the copper(+2) to copper(+1) reduction can occur in the course of ionization,^38,39^ it was impossible to establish the oxidation state of copper in the complexes based on their mass spectra.

**Fig. 5:**
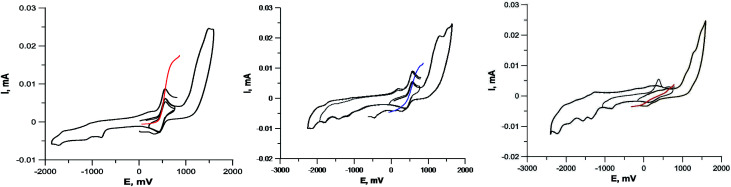
Cyclic voltammograms of complexes containing Cu^1+^Cu^1+^ (compound 5c, *left*), Cu^1+^Cu^2+^ (compound 5a, *center*) and Cu^2+^Cu^2+^ (compound 5i, *right*). GC electrode, DMF, 5 × 10^−4^ M, 0.1 M Bu_4_NClO_4_. Black curves – CVA, colored curves – RDE.

To determine the oxidation state of copper ions in coordination compounds 5, we applied the previously proposed^37^ electrochemical criterion, namely the evaluation of the anodic or cathodic nature of the 
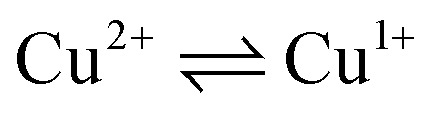
 transition at the voltammetric study of the complexes on a rotating disk electrode (RDE). RDE voltammograms allow to identificate the nature of 
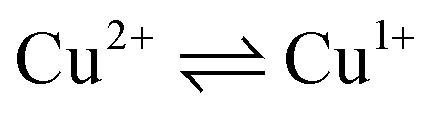
 redox transition currents (Fig. 5): for Cu^1+^/Cu^1+^ complexes only the oxidation current is observed (anodic process Cu^1+^ → Cu^2+^), for Cu^2+^/Cu^2+^ complexes only the reduction current is observed (cathodic reaction Cu^2+^ → Cu^+^1), for mixed valence complexes Cu^1+^/Cu^2+^ the corresponding current on the voltammogram has both anodic and cathodic components.

The CVA curves for non-copper-containing compounds may be recorded from 0 V to the cathodic or anodic region of potentials. For complexes 5 and 6, if the copper in their composition had an +2 oxidation state (a wave with a cathodic current on RDE), then the potential scanning was started from +0.7 V to register the direct reduction process Cu^2+^ → Cu^1+^ in order to obtain the true potential. In this case, if we start the reduction process from 0 V, then copper will already be reduced to Cu^1+^; the complexes of copper(i), as a rule, are less soluble than copper(ii) complexes, and often settle on the electrode, modifying it, which leads to erroneous determination of Cu^2+^ → Cu^1+^ transitions potential. The measured electrochemical oxidation and reduction potentials are presented in Table S1,† typical cyclic voltammograms (CV) and RDE curves are shown in Fig. 5 and in ESI.† Both Cu^1+^ and Cu^2+^ complexes have very similar redox potentials of 
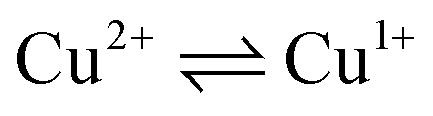
 transitions in the region of 0.05–0.6 V, see Table 1S†), which does not allow to distinguish their basing on CV curves, but the RDE curves let unambiguously determine that the above redox transitions correspond to oxidation processes for complexes 5b,c,d (and therefore copper in these complexes has the +1 oxidation state), the reduction processes for complexes 5i,k (therefore, these complexes contain copper(+2)), and for other complexes 5 this redox transition has both anodic and cathodic components (and therefore, they contain copper in both the +1 and +2 oxidation states).

Note that in all cases oxidation peaks on cyclic voltammograms have a significantly higher intensity compared to reduction peaks (Fig. 5) and, probably, represent a superposition of several peaks with similar potentials. Apparently, these peaks correspond to the processes of oxidation of coordinated chloride ions, as well as selenium ether fragments with cleavage of the Se–C bond, occurring at close potentials, analogously to that described in ref. 40.

**Fig. 6:**
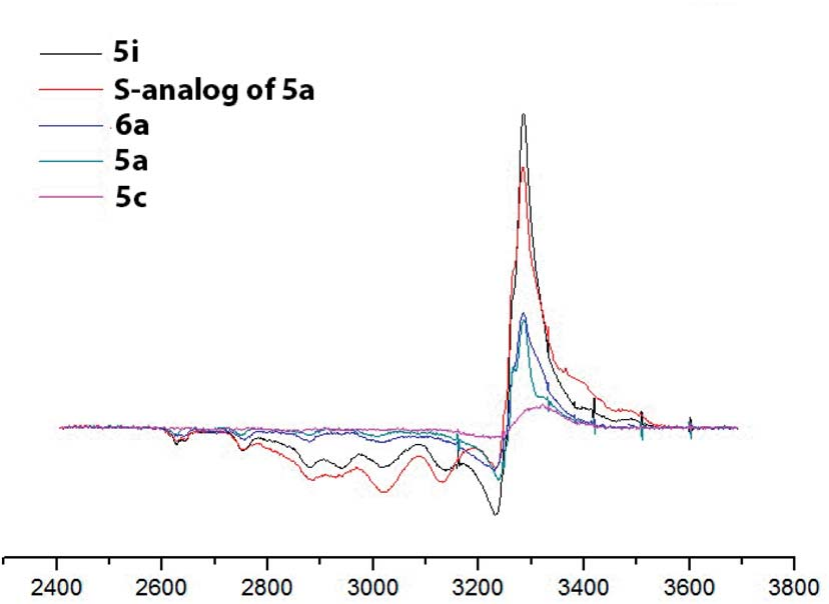
EPR spectra of the obtained copper coordination compound with different structural types. DMF, 77 K, *C* = 3 × 10^−2^ M for complexes 5, 6 and 5 × 10^−2^ M for 5a sulphur analog).

To confirm the redox state of copper in complexes 5 (as well as in perchlorate complexes 6 discussed below, see Section 2.2.2), we studied the EPR spectra for complexes of various structural types (Cu^2+^-containing complexes 5i, 6a; Cu^1+^ containing complex 5c; Cu^2+^Cu^1+^-containing complex 5a and, for comparison, its previously known sulfur-containing analogue^15,18^). The EPR spectroscopy is an invaluable method for characterizing the ligand environment and oxidation states of paramagnetic metal complexes, which enables unambiguously distinguishing Cu^1+^ (d^10^, S = 1) and Cu^2+^ (d^9^, S = 1/2) metal cations in the coordination compound based on their different magnetic susceptibilities. When conducting the EPR experiment, we dissolved corresponding complex in DMF (concentration 3 × 10^−2^ M for complexes 5, 6 and 4 × 10^−2^ M for 5a sulphur analog) and measured the intensity of the electron spin resonance (ESR) signal of Cu^2+^ in these solutions. The data obtained are presented in Fig. 6 and S32–S35†).

For the complexes, containing only Cu^2+^ ions (compounds 5i, 6a) the concentration of paramagnetic centers correspond to expected based on the mass of the dissolved sample calculation (he signal intensity for complex 5i is approximately twice the intensity of the signal of complex 6a, since the first complex is binuclear and the second is mononuclear; consequently at the same molar concentration the number of paramagnetic centers in the first case is twice as large). Mixed-valent complex 5a and its sulfur analog show the presence of a half of paramagnetic cents comparatively to the total number of presenting in them copper atoms. Cu^1+^ complex 5c demonstrated a very weak EPR signal, apparently associated with spontaneous oxidation of Cu^1+^ to Cu^2+^ in DMF solution, as was previously observed for its sulfur analogs,^15^ or with some admixture (about 5–7%) of the Cu^2+^ coordination compounds in the original solid sample used for solution preparation.

EPR also gave additional structural information on the symmetry of copper(ii) ions' coordination environment. A comparison of the EPR spectra of complexes 5a (Cu^2+^Cu^1+^), 5c (Cu^1+^Cu^1+^), 5i (Cu^2+^Cu^2+^) (Fig. 6 and S32–S35†) shows that the shape of the EPR spectrum are the same for all studied complexes, meaning that the Cu^2+^ coordination mode in all cases coincides. The experimental EPR spectra correlate with the sum of the simulated spectra for octahedrally and square pyramidally coordinated Cu^2+^, as was previously observed for the sulfur analogs of complexes 5 and was explained by the coordination of copper ions in solution with one or two DMF molecules.^41^

At the same time, integral intensity for complexes, containing Cu^2+^Cu^2+^, Cu^2+^Cu^1+^ or Cu^1+^Cu^1+^, of paramagnetic Cu2+ ions expectedly gradually decreases.

**Fig. 7:**
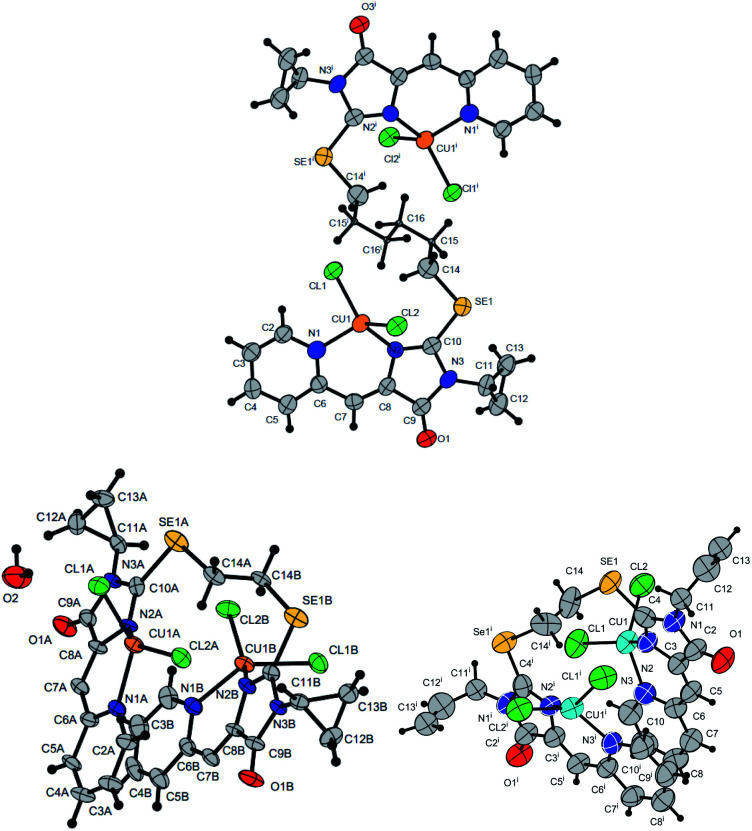
Molecular structures of complexes 5i (CCDC 2125344, *top*), 5e′ (CCDC 2017198, *left bottom*) and 5d′ (CCDC 2064245, *right bottom*). Thermal ellipsoids are given with 30% probability.

In some cases, the formed complexes, apparently, contain ∼5–20% of the admixtures of coordination compounds with the same ligand, but with copper in a different oxidation state (compounds 5b,d,e,g, see Table 1S†). For such cases, Scheme 4 shows the structure of the main product, and Table 1S† also shows the minor products, indicated by the number of the corresponding complex with a prime. The presence of a minor product is easily determined from the RDE curves, the ratio of the oxidation and reduction currents of copper on which in the case of the formation of an admixture products have no integer value. In the reaction with ligands 4d and 4e, the minor complex 5d′ and 5e′ was isolated from the reaction mixtures as the of crystals suitable for X-ray diffraction studies (Fig. 7).

The structures of compounds 5i, 5d′ and 5e′ of Cu^2+^Cu^2+^–type were confirmed by X-ray data (Fig. 7). In contrast to ligand 4d (Fig. 2), two pyridylmethylene-imidazolone fragments of the ligand in the complexes are located close to each other, so that two copper ions are on top of each other, and the nitrogen atoms of the pyridine and imidazolone rings are located in the *syn*-position, which allows them to coordinate Cu ions with the formation of six-membered chelate cycles. Coordination polyhedra of copper atoms in complexes 5i, 5d and 5e′ are distorted tetrahedra; each copper ion is coordinated by two nitrogen atoms of the organic ligand and two chloride anions. Apparently, the distorted tetrahedral environment of copper(+2) is also characteristic of other coordination compounds of the Cu^2+^Cu^2+^ and Cu^1+^Cu^2+^ types. The coordination environment of copper(+1) in complexes of the Cu^1+^Cu^1+^ and Cu^1+^Cu^2+^ types is apparently trigonal, similar to the previously described complexes with bis(5-pyridylmethylene-2-thio-imidazol-4-ones).^15,18,37^

Comparison of the reaction products of ligands 4a, 4c, 4d and 4e with similar S-containing ligands^15,18^ with CuCl_2_·2H_2_O under the same conditions (Table 1) shows that in the case of ligands with donor substituents (4-OEt-C_6_H_4_, all) at the N(3) nitrogen atom, copper is reduced more deeply upon complexation than for corresponding S-containing ligands. In the case of more acceptor substituents (4-Cl-C_6_H_4_, cyclo-C_3_H_7_) at the N(3) atom, complexation with sulfur- and selenium-containing ligands leads to the formation of products with the same structural type.

**Table 1:** Cu oxidation states in the complex forming as a result of CuCl_2_·2H_2_O reactions with ligands 4a,c,d,e and their sulfur-containing analogs

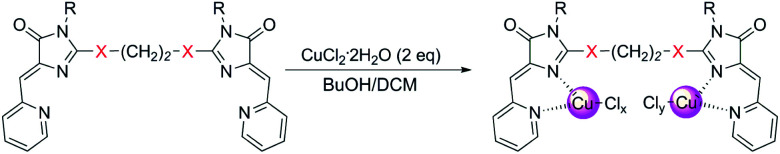								
Ligand	R = 4-OEt-C_6_H_4_, X = Se (4a)	R = 4-OEt-C_6_H_4_, X = S^15^	R = 4-Cl-C_6_H_4_, X = Se (4c)	R = 4-Cl-C_6_H_4_, X = S^15^	R = all, X = Se (4d)	R = all, X = S^18^	R = cyclo-C_3_H_7_, X = Se (4e)	R = cyclo-C_3_H_7_, X = S^18^
Cu oxidation states	+1/+2	+2/+2	+1/+1	+1/+1	+1/+1	+1/+2	+1/+2	+1/+2

To reveal the nature of the reducing agent, provoke the Cu^2+^ → Cu^1+^ transformation, we tested the mother liquor remaining after crystallization of complex 5c for ligand 4c reaction with CuCl_2_·2H_2_O in different solvents (CH_2_Cl_2_/*n*-butanol CH_2_Cl_2_/cyclohexanol, CH_2_Cl_2_/acetone), using LC-MS, and found that the main peak in the chromatograms with an intensity of 20 to 92% gives a substance with the *m*/*z* = 341; the second most intense peak with *m*/*z* = 300 (∼8%) corresponds to the hydrolysis product of the initial ligand ((*Z*)-3-(4-chlorophenyl)-5-(pyridin-2-ylmethylene)imidazolidine-2,4-dione).

**Scheme 5:**
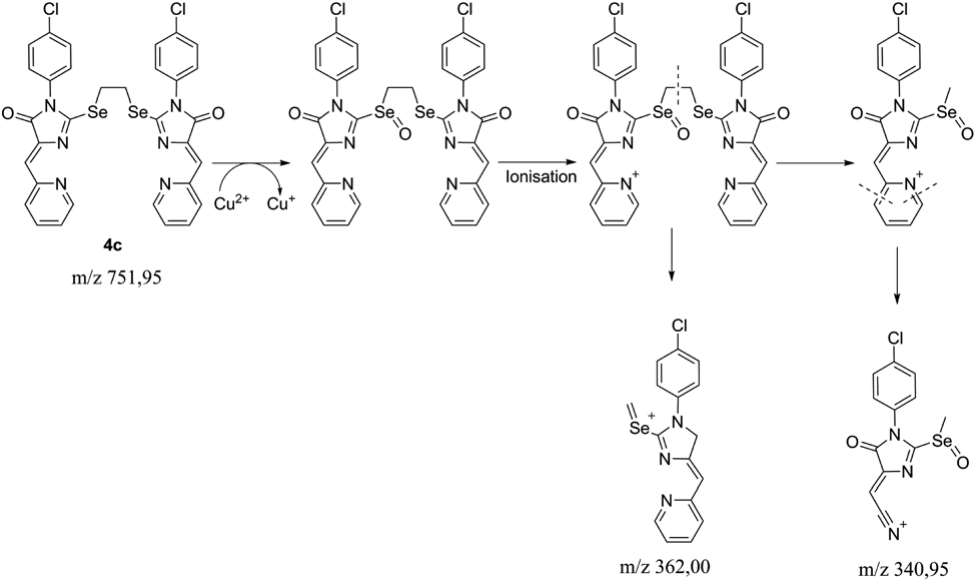
The proposed fragmentation scheme of the ligand 4c – CuCl_2_·2H_2_O reaction product.

The isotope splitting shows that the ion with *m*/*z* = 341 contains one Cl atom and does not contain copper; therefore, this ion may be a product of the transformation of the initial ligand 4c. In addition, in the case of using acetone as one of the solvents, a peak with 21% intensity with *m*/*z* = 325 (341-CH_3_) was found, which suggests the presence of labile methyl moiety in the structure of the compound with *m*/*z* = 341. Based on these data and the previously described mechanism of fragmentation of substituted pyridines,^42^ we can assume the following sequence of processes occurring during the reduction of some Cu^2+^ ions to Cu^1+^ and subsequent ionization of the Se-containing ligand oxidation products (Scheme 5). The peak with *m*/*z* = 362 is found as a minor one in all spectra containing the ion with *m*/*z* = 341, which also confirms the proposed fragmentation scheme.

Thus, it can be argued that ligand 4c is a direct reducing agent of copper in complexation reactions. For sulfur ligands, in a previous study, it was proved that the ligand does not participate in reduction, and it proceeds only when the reaction is carried out in reducing solvents (alcohols or DMF).^15,37^ In the case of ligands 4, copper reduction occurs even during complexation in CH_2_Cl_2_/acetone, none of which is capable of reducing Cu^2+^. Thus, the replacement in the ligand of sulfur with selenium fundamentally changes the copper reduction scheme.

It should be noted that, in general, some sulfur-containing ligands can reduce CuCl2 to Cu^+^ with the oxidation of divalent sulfur to a disulfide or sulfoxide fragment without the participation of a solvent.^43–45^ However, in the case of sulfur analogs of ligand 4, such a process does not occur.^15,37^

**Fig. 8:**
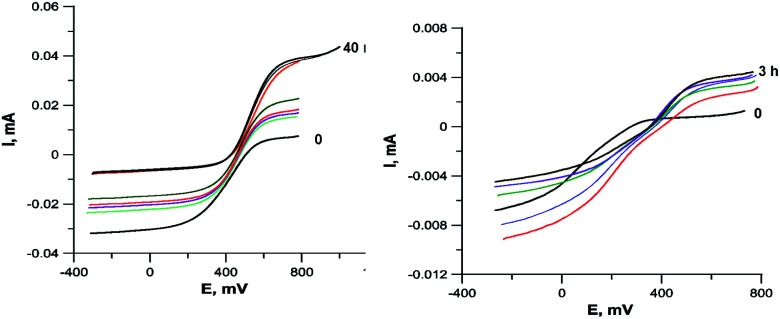
RDE curves demonstrating the Cu^2+^ to Cu^1+^ reduction during the interaction of ligand 4c with CuCl_2_·2H_2_O in DMF (left, the entire process in 40 min) and DMSO (right, the entire process in 3 hours). C = 5 × 10^−4^ M, 2 equiv. CuCl_2_·2H_2_O, 0.1 M Bu_4_NClO_4_.

To confirm that the reduction of Cu^2+^ may occurs under the action of an organic ligand, not a solvent, we carried out an electrochemical study of the 4c + CuCl_2_·2H_2_O reaction in the reducing (DMF) and non-reducing (DMSO) solvents. The results are shown in Fig. 8. In both cases, the current of the Cu^2+^Cu^1+^ redox transition on the RDE is initially completely cathodic, *i.e.*, corresponds to the reduction of Cu^2+^, but the contribution of the anodic current component increases with time. In the case of DMF, the reduction of copper is completed in 40 min by its complete transformation into Cu^1+^. In DMSO, the reduction proceeds more slowly and is completed in 3 h, and the final solution contains Cu^2+^ and Cu^1+^ in a 1 : 1 ratio, apparently due to the fact that the ligand oxidation product is incapable of further reduction of copper.

**Scheme 6:**
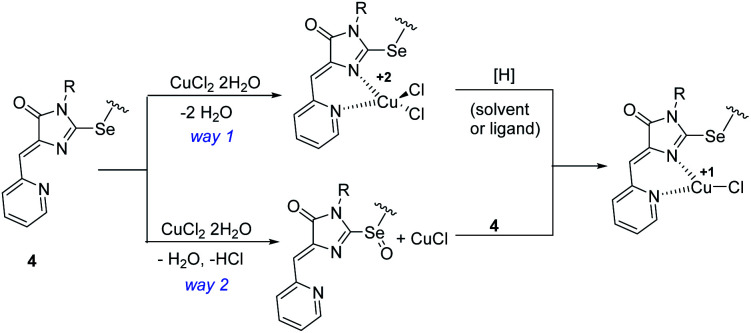
Alternative sequences of the oxidation-reduction and complexation stages in the reactions of ligands 4 with copper(ii) chloride.

Considering the possible sequence of reduction and complexation stages during the formation of Cu^1+^ containing products 5a-h,j, two possible reaction ways may be assumed: the initial coordination of the metal ion and its subsequent reduction (Scheme 6, way (1) and the initial reduction of CuCl_2_ in solution with the ligand molecule oxidation to sulfoxide derivative (the oxygen atom appears to be derived from water molecules coordinated by the original inorganic copper salt) and subsequent coordination of the formed CuCl with ligand 4 (Scheme 6, way 2). In the first case, both the starting ligand 4 and the solvent can potentially be reducing agents; in the second case, it can be only the initial ligand, since CuCl_2_ is not reduced to CuCl when dissolved in alcohol or DMF. We performed ^1^H NMR monitoring of the reaction mixture of ligand 4c with CuCl_2_·2H_2_O in DMF and DMSO. The results are shown in Fig. S1.† When the reaction is carried out in DMF, the signals of the CH_2_Se groups appearing at 4.11 ppm for the free ligand and at 5.42 for its coordination compound 5c were tracked; in DMSO, the changes in the vinyl protons signals (6.57 and 6.68 for ligand 4c and complex 5c, respectively) were more characteristic.

**Scheme 7:**
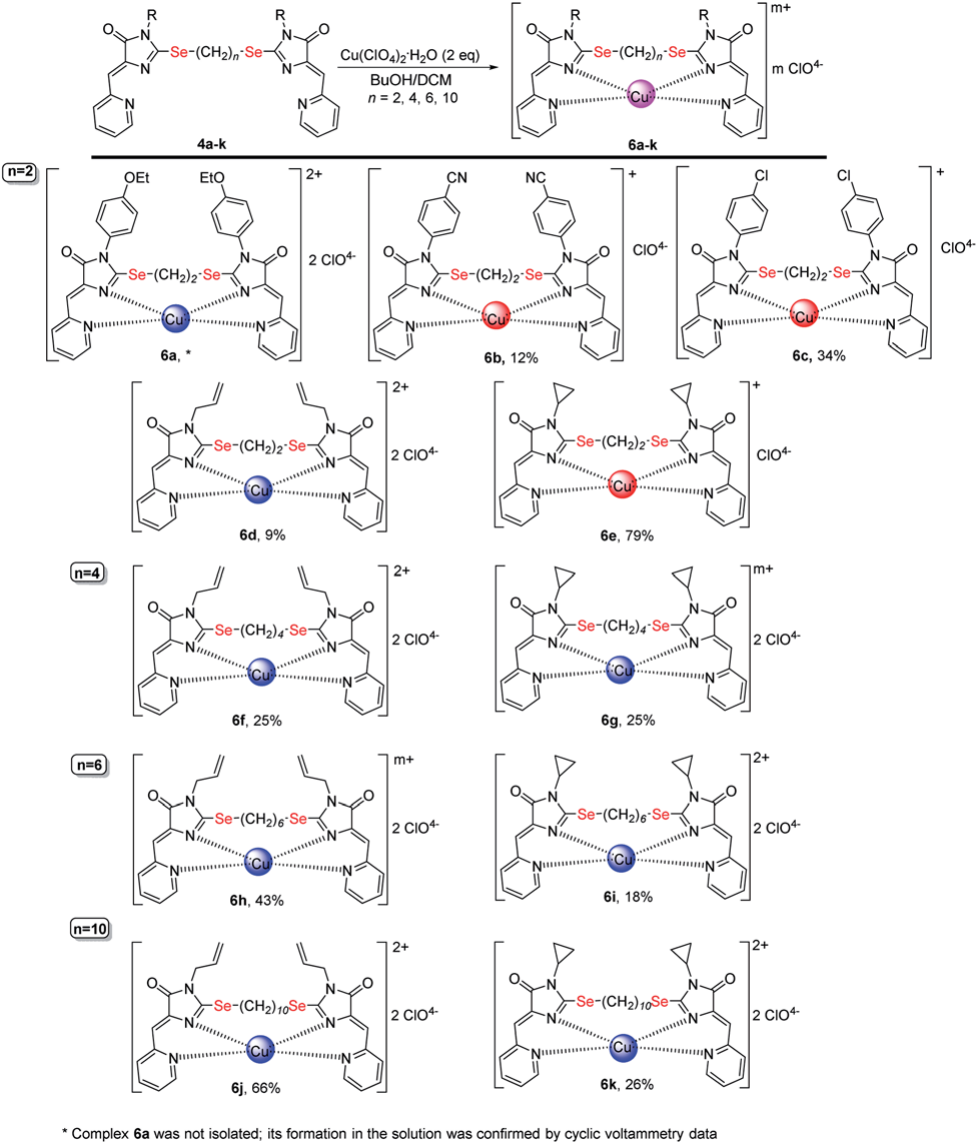
Coordination compounds 6a-k formed in the reactions of ligands 4 with Cu(ClO_4_)_2_·6H_2_O. The red circles represent Cu^1+^, the blue circles represent Cu^2+^. The structures of the main reaction products are given, see also Table 1S† and discussion in the text.

According to the NMR data, in DMF no additional peaks in the region of CH_2_Se protons were detected, besides the peaks of the ligand and final complex. As expected, the intensity of the ligand peak decreases with time, while the complex increases; however, the peak of the starting ligand still has a rather high intensity (4a/5a ratio = 1 : 3) 100 min after the reagents mixing, whereas, according to the RDE study, copper reduction is almost completed after 40 min of reaction. A similar pattern is observed in DMSO: the signal of free ligand 4a in NMR spectrum completely disappears only after 24 hours of the reaction, while the reduction of copper according to the data of electrochemical studies completed in ∼3 hours. Thus, the reduction proceeds faster than complexation, which confirms that way 2 on Scheme 6 is at least one of the possible reaction routes, although apparently not the only one in a reducing solvent, which is confirmed by a faster course of reduction in DMF as compared to DMSO.

The reduction of Cu^2+^ in DMSO under the action of the ligand is also confirmed by the incomplete Cu^2+^ → Cu^1+^ reduction when ligand 4 and CuCl_2_·2H_2_O mixing in an electrochemical cell in a 1 : 2 ratio (Fig. 8). In this case, apparently, the oxidized ligand does not further participate in the redox reaction and does not form copper complex. However, the formation of complex 5c, even in this case, confirms that both ways of complex formation shown in Scheme 6 run in parallel, since if only the second path was implemented in this case, all the ligand introduced into the reaction would be spent on copper reduction. It is also possible that the reduction of the first copper atom in the complex can proceed under the action of both the ligand and the solvent, while the second one can proceed only under the action of the solvent.

Thus, selenium-containing ligands 4 can be direct copper(ii) reducing agents in complexation reactions, which confirms the assumption of their higher donor ability as compared to sulfur analogues. However, when the reaction is carried out in reducing solvents (alcohols, DMF), the redox process proceeds faster and leads to a deeper reduction of copper, and, probably, the reduction of the metal can proceed *via* two alternative pathways, with a ligand or solvent as a reducing agent.

#### 2.2.2 Reactions with copper(ii) perchlorate

**Fig. 9:**
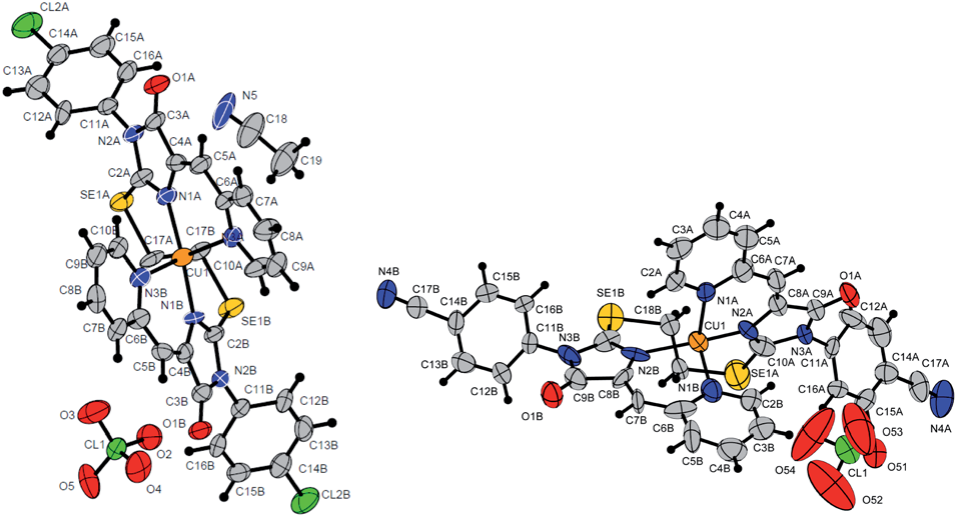
Molecular structure of complex 6c (CCDC 2125345; a solvate with CH_3_CN) and 6b (CCDC 2017203). Termal ellipsoids are given in 30% probability.

In the reactions with Cu(ClO_4_)_2_·6H_2_O, ligands 4a-k react as tetradentate ligands, forming mononuclear coordination compounds 6a-k (Scheme 7), which structures were confirmed by mass spectrometry data (HRMS and MALDI), FTIR spectroscopy, and in the case of compounds 6b, 6c by X-ray data (Fig. 9). The distorted tetrahedral coordination polyhedron of copper in complexes 6b, 6c is formed by two nitrogen atoms of the pyridine and two nitrogen atoms of the imidazolone ring of the organic molecule. According to IR spectra, all complexes 6 contain perchlorate anions in the outer sphere, which is confirmed by the presence of broad bands near 1100 cm^−1^ and a sharp band near 625 cm^−1^ without splitting pattern, which could be due to the antisymmetric stretching and bending of not coordinated with the metal perchlorate ions.^46,47^ We suppose that such a dramatic change in the reaction outcome at switching from CuCl_2_ to Cu(ClO_4_)_2_ at complexation process is due to the different coordinating abilities of chloride and perchlorate anions: the more donor chloride easily binds directly to the copper ion, entering its internal coordination sphere, while the non-nucleophilic perchlorate prefers to be located in the external coordination sphere, which leads to the coordination of the copper by all four donor nitrogen atoms of the ligand.

As in the case of complexation with copper(ii) chloride, in the reactions of ligands 4 with Cu(ClO_4_)_2_·6H_2_O, in some cases, complete reduction of Cu^2+^ to Cu^1+^ occurs, or the mixtures containing a certain amount of a complex with copper to a different oxidation state, were formed (see Table and S1;†Scheme 7 shows the main products of complexation reactions).

The oxidation state of copper in perchlorate complexes 6 was established according to the data of electrochemical study by RDE method (Fig. S9, ESI†) and EPR spectra, as in the case of complexes 5. According to RDE data, in most complexes 6 copper retains the initial oxidation state +2. At the same time, upon complexation with ligands 4b, 4c and 4e, copper is reduced during the reaction with the formation of Cu^1+^-containing complexes 6b, 6c and 6e. All three ligands 4b, 4c, 4e have (CH_2_)_2_ groups between selenium atoms and π-acceptor substituent (4-CN-C_6_H_4_, 4-Cl-C_6_H_4_, and cyclopropyl, respectively) at the N(3) atoms of the imidazolone fragments; thus, these ligands are the least donor of all studied ligands 4 and, therefore, are most capable to coordinate copper in a low oxidation state.

Some general patterns may be noted in the course of copper reduction at the reaction of ligands 4 with CuCl_2_·2H_2_O and Cu(ClO_4_)_2_·6H_2_O.

(1) Mesomeric acceptor substituent (CN, Cl) in the benzene ring at the N(3) imidazolone atoms (ligands 4b, 4c) decreases the donor properties of the ligand, which, as a result, better stabilizes copper in the oxidation state +1 than +2, and forms Cu^1+^-containing complexes (compounds 5b,c and 6b,c) in the reactions with both CuCl_2_ and Cu(ClO_4_) _2_.

(2) Ligands with electron-donating substituents (4-EtO-C_6_H_4_, All) at the N(3) atom and a short 2-carbon linker between selenium atoms (compounds 4a and 4d) can form both Cu^1+^ (compound 5d) and Cu^2+^ (compound 6a, 6d) containing complexes, or the complex of Cu^1+^Cu^2+^ type (compound 5a). However, with a longer and, accordingly, more donor alkyl chain of the linker containing 4–10 carbon atoms (ligands 4f, 4h, 4j), the formed complexes contain exclusively or at least one Cu^2+^ ion (compounds 5f, 5h, 5j, 6f, 6h, 6j).

(2) The weak electron-donor effect of the cyclopropyl group in ligands 4e, 4g, 4i, 4k leads to the stabilization of Cu^2+^ in the complexes, which, in reactions with CuCl_2_, contributes to the formation of coordination compounds of Cu^2+^Cu^2+^ or Cu^2+^Cu^1+^ types, and with an increase in the length of alkyl linkers between selenium atoms leads to the formation of only Cu^2+^Cu^2+^ complexes.

Thus, to summarize, stronger ligands (donor substituents at imidazolone N(3) atoms, long polymethylene chains between selenium atoms) generally form Cu^2+^ complexes; weaker ligands (acceptor substituents at N(3), short polymethylene chains between selenium atoms) generally stabilize Cu^2+^ complexes. Such influence of electronic effects of the substituent may be explained by a change in the HOMO energy of the ligand passing through the cross-conjugated π-electron system of pyridylmethylene-chalcogenimidazolone ligands, and by a significant contribution to it from the exocyclic chalcogen atom, although not directly involved in the coordination of the metal.^48^

### 2.3. Cytotoxicity of ligands and coordination compounds

Some of the obtained ligands and compounds were tested for cytotoxicity using the standard 3-(4,5-dimethylthiazol-2-yl)2,5-diphenyl tetrazolium bromide (MTT) test.^49^ The results are presented in Table 2. This study was realized using the cell lines of breast cancer MCF7, human lung carcinoma A549, non-cancer human embryonic kidney cell line HEK293T, and the non-cancer lung fibroblast VA13 cell line, along with the results obtained for doxorubicin and cisplatin as known cytotoxic drugs used in clinical practice. A549 and MCF7 were selected for the lung tumor model and breast cancer, because (i) these cell lines are well-studied and often used in cytotoxicity investigations in the literature, (ii) they have a high proliferation rate typical for cancer cells and (iii) for cell lines MCF7 and HEK293, there are literature data on the cytotoxicity of sulfur analogs, which make it possible to assess the significance for cytotoxic activity of the replacement of sulfur with selenium in the composition of ligands and complexes.

**Table 2:** Cytotoxicity (CC_50_) of some ligands and coordination compounds, measured by MTT test (nt – non toxic; dash – not tested)

Compound	CC_50_, μM				
	A549	MCF7	MCF10A	VA13	HEK293T
4e	4.11 ± 0.97	8.38 ± 2.26	3.28 ± 0.43	4.26 ± 0.94	—
4g	nt	18.79 ± 5.42	nt	61.49 ± 3.68	—
4h	77.26 ± 32.39	33.91 ± 12.97	10.48 ± 2.73	51.66 ± 5.1	—
4i	nt	nt	3.28 ± 0.43	47.1 ± 5.51	—
4j	11.59 ± 0.16	2.32 ± 0.48	7.74 ± 1.03	10.83 ± 2.36	—
4k	105 ± 21.92	33.6 ± 2.63	11.45 ± 0.76	45.5 ± 0.31	—
5b	3.1 ± 0.1	2.7 ± 0.3	—	2.1 ± 0.3	2.4 ± 0.2
5d	2.47 ± 0.53	0.49 ± 0.07	1.71 ± 0.52	4.18 ± 2.58	—
5e	1.23 ± 0.22	1.49 ± 0.38	1.2 ± 0.12	5.18 ± 3	—
5f	7.9 ± 2.4	5.9 ± 0.7	—	3.9 ± 0.2	6.8 ± 2.7
5h	7.4 ± 0.6	5 ± 0.6	—	—	—
5i	1.74 ± 0.34	1.09 ± 0.02	0.87 ± 0.04	1.13 ± 0.07	—
5j	4.8 ± 0.2	4.2 ± 0.5	—	—	—
5k	6.13 ± 0.23	4.66 ± 3.89	3.63 ± 1.95	2.04 ± 1.23	—
6e	0.7 ± 0.1	1.8 ± 0.2	—	0.5 ± 0	1.6 ± 0.2
6f	1.8 ± 0.1	1.9 ± 0.1	—	1.6 ± 0.1	2.4 ± 0.1
6i	9.2 ± 0.5	6.9 ± 0.3	—	5.2 ± 0.3	7.4 ± 3.2
6k	9.3 ± 0.5	8.4 ± 0.4	—	6 ± 0.4	6.8 ± 1.0
Cisplatin, μM	>30	a14 ± 2.3 (48 h)	a11 ± 3 (48 h)	2.9 ± 0.3	12.4 ± 3.9
		a9 ± 1.7 (72 h)	a7 ± 0.8 (72 h)		
Doxorubicin, nM	47.9 ± 7.9	55.4 ± 11.8	—	159.9 ± 27.4	11.5 ± 3.2

a Ref. 50.

Most of the studied copper-containing complexes demonstrated high cytotoxicity, exceeding the cytotoxicity of cisplatin and doxorubicin. On the whole, copper complexes are expected to be more toxic than free ligands, which can be traced for the series of compounds 4-6e, 4-6f, 4-6i, 4-6k.

Table 3 demonstrates a comparison of the cytotoxicity of some compounds tested in this work with similar ligands and complexes containing sulfur atoms instead of selenium. The presented data show that the complexes of selenium-containing ligands are in most cases several times more toxic than their sulfur analogs; moreover, they show a higher selectivity in relation to cancer cell lines.

**Table 3:** Comparative cytotoxicity of some selenium-containing ligands and complexes and their sulfur-containing analogs (cell lines MCF7 and HEK293; MTT or MTS test)

Compound	CC_50_, μM		Selectivity Index
	MCF7	HEK293	
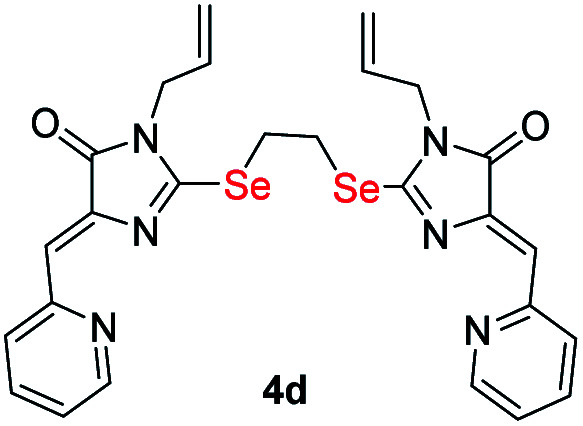	2.97 ± 0.15	3.21 ± 0.27	1.3
S-analog of 4d^18^	15.9 ± 1.4	>100	>6.3
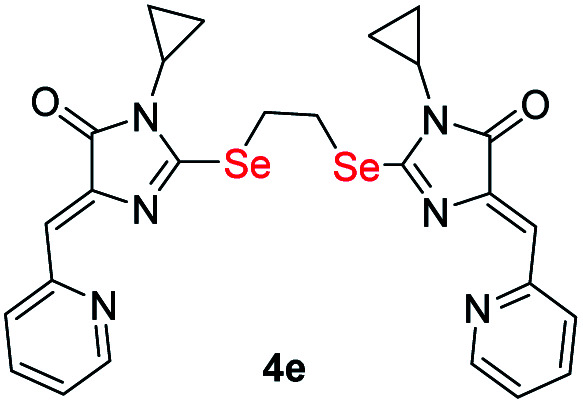	8.38 ± 2.26	4.26 ± 0.94	0.5
S-analog of 4e^18^	>100	>100	
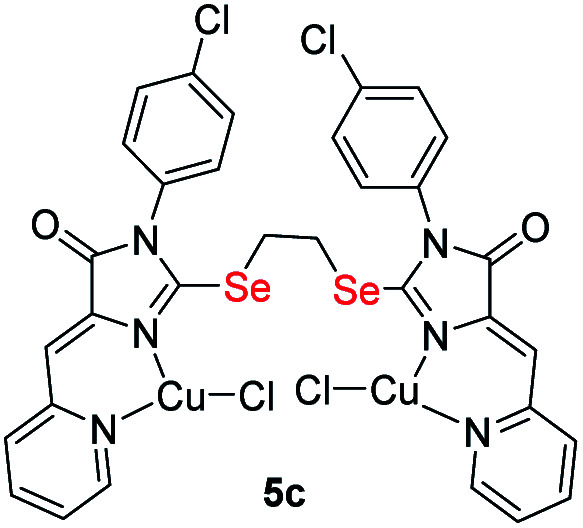	4.18 ± 0.65	0.87 ± 0.14	0.2
S-analog of 5c^15^	0.6 ± 0.0	—	1.3
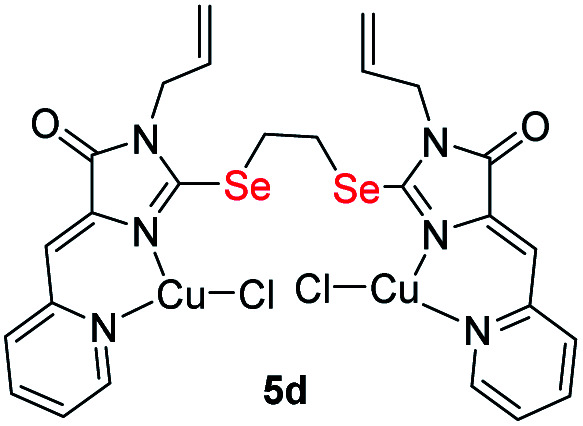	0.49 ± 0.07	3.7 ± 0.6	**8.5**
S-analog of 5d^18^	3.7 ± 1.6	2.5 ± 0.4	0.7
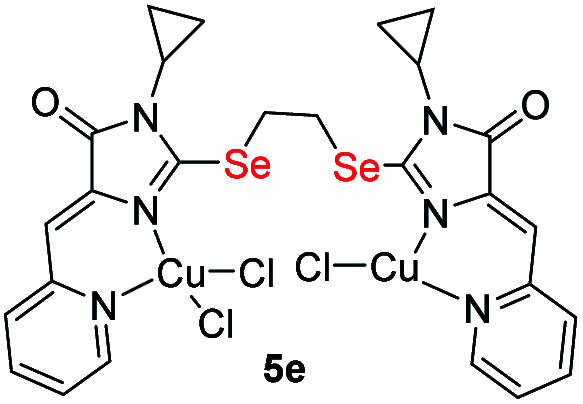	1.49 ± 0.38	3.9 ± 0.6	**2.6**
S-analog of 5e^18^	7.4 ± 1.4	25.3 ± 1.2	3.4

It should also be noted that some selenium organic ligands obtained in this work, exhibit a sufficiently high cytotoxicity, not less in some cases than the cytotoxicity of coordination compounds (for example, ligand 4e). This result makes it possible to consider bis-5-pyridylmethylene-2-selenohydantoins derivatives themselves as cytotoxic agents.

## 3. Experimental

### 3.1. Materials and methods

Reagent-grade chemicals were used throughout, and the solvents were purified by standard methods. Column chromatography was done on 60 Å silica gel from Merck; thin-layer chromatography was performed on Merck 60F254 plates. Melting points were determined using OptiMelt MPA100, 1 °C min^−1^, 0.1 °C resolution.

NMR spectra were acquired on Bruker Avance 600, Bruker Avance 400, Agilent 400-MR, and Bruker Fourier 300 at room temperature; the chemical shifts δ were referenced to the solvents (CDCl_3_: *δ*_H_ = 7.26, *δ*_C_ = 77.0; DMSO-d6: *δ*_H_ = 2.50, *δ*_C_ = 39.5).

Infrared spectra were recorded on Thermo Nicolet iS5 FTIR, with 32 scans, 4 cm^−1^ resolution, and attenuated total reflectance (ATR) sampling.

Reactions were monitored by LCMS using Thermo Dionex Ultimate 3000 with ABSciex 3200 Qtrap with a Thermo Acclaim RSLC 120 C18 3 μm (150 × 4.6 mm2) column.

Elemental analysis was performed using PerkinElmer 2400 Series II elemental analyzer. In the cases when mixtures of coordination compounds with copper in various oxidation states were formed in the reactions (according to the data of electrochemical research), elemental analysis did not give reproducible results and its data are not presented.

High resolution mass spectra (HRMS) were recorded on an Orbitrap Elite mass spectrometer (Thermo Scientific). For the solutions with a concentration of 0.1–9 μg ml^−1^ (in 1% formic acid in acetonitrile), direct injection into the ion source was used by a syringe pump (5 μL min^−1^). Spray voltage ± 3.5 kV, capillary temperature 275 °C. Mass spectra were recorded using an Orbitrap analyzer with a resolution of 480 000 (1 microscan). Maximum input time 900 ms, averaging over 9 spectra, mass range 90–2000 Da, in some cases 200–4000 Da. For internal calibration, the signals of DMSO and diisooctyl phthalate (*m*/*z* 157.03515 and 413.26623) in the positive mode and the signal of dodecyl sulfate (*m*/*z* 265.14790) in the negative mode were used.

Mass spectra of matrix-activated laser desorption/ionization (MALDI) were recorded on a Bruker Autoflex II instrument (resolution FWHM 18000) equipped with a nitrogen laser with a working wavelength of 337 nm and a time-of-flight mass analyzer operating in the reflectron mode. Accelerating voltage 20 kV. The samples were applied to a polished steel substrate. The spectra were recorded in the positive ion mode. The resulting spectrum was the sum of 50 spectra obtained at different points in the sample. *trans*-2-[3-(4-*tert*-Butylphenyl)-2-methyl-2-propenylidene]malononitrile (DCTB) and 9-nitroanthracene (Ant) (Acros, 99%) were used as matrices where needed to facilitate ionization.

Electronic absorption spectra were measured on a Hitachi U2900 instrument with an operating wavelength range of 190–1100 nm in a quartz cuvette from Agilent Technologies with an optical path of 10 mm. Before recording each spectrum, the background signal was recorded in pure solvent; the background signal was subtracted by the spectrophotometer in an automatic mode.

Electrochemical studies were conducting using an IPC Pro M potentiostat. The working electrode was a glassy carbon disk (*d* = 2 mm), the reference electrode was Ag/AgCl/KCl (sat.). The auxiliary electrode was a platinum plate, and the supporting electrolyte was a 0.1 M Bu_4_NClO_4_ solution in DMF. In the study by the CV method, the potential sweep rate is 100 mV s^−1^, in the study by the VDE method −20 mV s^−1^. All measurements were carried out in a dry argon atmosphere; samples were dissolved in a previously de-aerated solvent.

For X-ray studies the data were collected by using an STOE diffractometer, a Pilatus100K detector, focusing mirror collimation Cu Kα (1.54086 Å) radiation, and the rotation method mode. STOE X-AREA software was used for cell refinement and data reduction. Data collection and image processing were performed with X-Area 1.67 (STOE & Cie GmbH, Darmstadt, Germany, 2013). Intensity data were scaled with LANA (part of X-Area) to minimize differences of intensities of symmetry-equivalent reflections (multiscan method). The structures were solved and refined with the SHELX program.^51^ The non hydrogen atoms were refined by using the anisotropic full matrix least square procedure. Hydrogen atoms were placed in the calculated positions and allowed to ride on their parent atoms. The molecular graphics were prepared by using DIAMOND software.^52^

The MTT assay was carried out according to^49^ with few modifications. 3000 Cells (for HEK293T, A549 and MCF7 and MCF10A cell lines) or 4000 cells (for VA13 cell line) were seeded in each well of a 96-well plate. After 20 h incubation, the tested compounds diluted in culture medium were added to the cells and incubated 72 h at 37 °C under CO_2_ (5%) atmosphere. Assays were performed in triplicates. The MTT (3-[4,5-dimethylthiazol-2-yl]-2,5 diphenyl-tetrazolium bromide) reagent was then added to the cells up to final concentration of 0.5 g l^−1^ (10× stock solution in PBS was used) and incubated for 2 h at 37 °C (5% CO_2_). The MTT solution was then discarded and 140 μl of DMSO was added. The plates were swayed on a shaker (60 rpm) to solubilize the formazan. The absorbance was measured using a microplate reader at a wavelength of 565 nm. The analysis of cytotoxicity and the estimation of IC50 values were carried out with the built-in functions in the GraphPad Prism program (GraphPad Software, Inc., San Diego, CA).P53 activation.

EPR spectra were recorded on Varian E-3 X-band radiospectrometer at 77 K in a capillary with an inner diameter of 1 mm. DMF grade “pure” was purified by stirring over freshly calcined CuSO_4_ for 3 days, followed by distillation in vacuum over CaH_2_ at *t* ≤45 °C. The diamagnetically diluted solution of Mn^2+^ ions in MgO was used an internal standard for the value of the magnetic field induction.

### 3.2. Synthetic procedures

Synthesis of selenourea (1 and 2a-e) and 2-selenoxoimidazolidin-4-one (3c-d): see.^34^ The new compounds 3a, 3b, 3e were synthesized in the same way.

#### 3.2.1 (*Z*)-3-(4-Ethoxyphenyl)-5-(pyridin-2-ylmethylene)-2-selenoxoimidazolidin-4-one (3a)

Red solid. Yield: 270 mg, 49%. mp 213–214 °C. ^1^H NMR (400 MHz, DMSO-d6) *δ* = 1.36 (t, *J* = 7.0, 3H, OEt), 4.08 (q, *J* = 6.9, 2H, OEt), 6.92 (s, 1H, CH), 7.04 (d, *J* = 8.9, 2H, H_Ar_), 7.31 (d, *J* = 8.9, 2H, H_Ar_), 7.47 (ddd, *J* = 7.6, 4.9, 1.0, 1H, H_Py_), 7.81 (d, *J* = 7.8, 1H, H_Py_), 7.92 (td, *J* = 7.7, 1.7, 1H, H_Py_), 8.80 (d, *J* = 4.0, 1H, H_Py_), 12.22 (s, 1H, NH). ^13^C {^1^H} NMR (101 MHz, DMSO-d6) *δ* = 14.6, 63.4, 109.2, 114.5 (2C), 123.5, 126.2, 127.0, 130.1 (2C), 137.7, 150.1, 153.3, 158.8, 163.0, 179.0. FTIR (Diamond, cm^−1^): 525, 581, 616, 642, 690, 735, 746, 782, 801, 829, 855, 929, 961, 1047, 1120, 1142, 1159, 1174, 1248, 1272, 1304, 1412, 1428, 1472, 1451, 1588, 1512, 1657, 1733, 2873, 2935, 2979, 3061, 3351. HRMS (FTMS + cESI) *m*/*z*: [M + H]^+^, calculated for C_17_H_15_N_3_O_2_Se 374.0402; found 374.0391.

#### 3.2.2 (*Z*)-4-(5-Oxo-4-(pyridin-2-ylmethylene)-2-selenoxoimidazolidin-1-yl)benzonitrile (3b)

Orange solid. Yield: 465 mg, 58%. mp 284–285 °C. ^1^H NMR (400 MHz, DMSO-d6) *δ* = 6.97 (s, 1H, CH), 7.48 (dd, *J* = 6.8, 5.4, 1H, H_Py_), 7.71 (d, *J* = 8.4, 2H, H_Ar_), 7.84 (d, *J* = 7.7, 1H, H_Py_), 7.94 (td, *J* = 7.7, 1.1, 1H, H_Py_), 8.04 (d, *J* = 8.3, 2H, H_Ar_), 8.81 (d, *J* = 4.2, 1H H_Py_), 12.37 (s, 1H, NH). ^13^C {^1^H} NMR (75 MHz, DMSO-d6) *δ* = 109.7, 111.8, 118.2, 123.6, 127.0, 130.0, 130.3 (2C), 132.9 (2C), 137.7, 138.0, 150.1, 153.1, 162.5, 177.7. FTIR (Diamond, cm^−1^): 545, 560, 583, 681, 735, 783, 832, 848, 911, 870, 956, 1079, 1101, 1163, 1144, 1178, 1217, 1271, 1308, 1371, 1409, 1425, 1453, 1510, 1473, 1589, 1605, 1651, 1737, 2233, 3064, 3103, 3291. HRMS (FTMS + cESI) *m*/*z*: [M + H]^+^, calculated for C_16_H_10_N_4_OSe 355.0093; found 355.0093.

#### 3.2.3 (*Z*)-3-(4-Chlorophenyl)-5-(pyridin-2-ylmethylene)-2-selenoxoimidazolidin-4-one (3c)

Brown solid. Yield: 940 mg, 72%. mp 255–256 °C. ^1^H NMR (400 MHz, DMSO-d6) *δ* = 6.95 (s, 1H, CH), 7.46–7.50 (m, 3H, 2H_Ar_+1H_Py_), 7.61 (d, *J* = 8.6 Hz, 2H, H_Ar_), 7.83 (d, *J* = 7.7 Hz, 1H, H_Py_), 7.93 (td, *J* = 7.7, 1.3 Hz, 1H, H_Py_), 8.81 (d, *J* = 4.3 Hz, 1H, H_Py_), 12.30 (s, 1H, NH). ^13^C {^1^H} NMR (101 MHz, DMSO-d6) *δ* = 109.5, 123.6, 127.1, 129.0 (2C), 130.2, 131.0 (2C), 132.8 (s), 133.8, 137.7, 150.1, 153.2, 162.8, 178.4. FTIR (Diamond, cm^−1^): 546, 579, 675, 721, 782, 823, 953, 1021, 1093, 1156, 1143, 1180, 1214, 1266, 1307, 1404, 1423, 1452, 1471, 1588, 1494, 1651, 1737, 3066, 3290. HRMS (FTMS + cESI) *m*/*z*: [M + H]^+^, calculated for C_15_H_11_ClN_3_OSe 363.9750; found 363.9748.

#### 3.2.4 General procedure for synthesis of (4*Z*,4′*Z*)-2,2′-(alkanes-1,2-diylbis(selanediyl))bis(1-substituted-4-(pyridin-2-ylmethylene)-1*H*-imidazol-5(4*H*)-ones) (4)

To a solution of corresponding 3-substituted-5-((alkyl/aryl)ide)-2-selenoximidazolidin-4-one 3 in DMF (1 eq.) K_2_CO_3_ (1.5 eq.) was added. The resulting mixture was stirred for 10 min and cooled to −10 °C, then α,ω-dibromoalkane (0.5 eq.) was added to the chilled solution. After 3–4 h. The reaction mixture was warmed to room temperature, wherein a color change from red to yellow was observed. Upon completion of the reaction (TLC control), the reaction mixture was diluted with distilled water, the formed precipitate was filtered, washed successively with EtOH and Et_2_O. The products 4 were isolated by column chromatography on silica gel with the subsequent recrystallization from DCM/petroleum ether mixture.

#### 3.2.5 (4*Z*,4′*Z*)-2,2′-(Ethane-1,2-diylbis(selanediyl))bis(1-(4-ethoxyphenyl)-4-(pyridin-2-ylmethylene)-1*H*-imidazol-5(4*H*)-one) (4a)

In a solution of 150 mg of (3a) in 3 ml of DMF with 38 mg of 1,2-dibromoethane and 95 mg K_2_CO_3_. Yellow solid. Yield: 135 mg, 80%. mp 212–213 °C. ^1^H NMR (400 MHz, CDCl_3_) *δ* = 1.45 (t, *J* = 7.0 Hz, 6H, OEt), 3.88–3.91 (m, 4H, 2 × CH_2_), 4.08 (q, *J* = 7.0 Hz, 4H, OEt), 6.99 (d, *J* = 8.9 Hz, 4H, H_Ar_), 7.13 (s, 2H, CH), 7.19 (ddd, *J* = 7.6, 4.9, 0.8 Hz, 2H, H_Py_), 7.24 (d, *J* = 8.9 Hz, 4H, H_Ar_), 7.59 (td, *J* = 7.8, 1.7 Hz, 2H, H_Py_), 8.64 (d, *J* = 4.4 Hz, 2H, H_Py_), 8.72 (d, *J* = 8.1 Hz, 2H, H_Py_). ^13^C {^1^H} NMR (101 MHz, CDCl_3_) *δ* = 14.9 (2C), 26.7 (2C), 64.0 (2C), 115.5 (4C), 123.5 (2C), 124.9 (2C), 125.1 (2C), 127.1 (2C), 128.6 (4C), 136.1 (2C), 140.4 (2C), 150.1 (2C), 153.6 (2C), 159.9 (2C), 164.3 (2C), 168.6 (2C). FTIR (Diamond, cm^−1^): 526, 538, 570, 622, 638, 662, 696, 745, 825, 777, 838, 904, 923, 950, 1041, 1086, 1115, 1161, 1173, 1210, 1228, 1296, 1253, 1392, 1332, 1430, 1475, 1512, 1564, 1578, 1638, 1605, 1735, 2943, 2988, 3057. HRMS (FTMS + cESI) *m*/*z*: [1/2M + 2H]^+^, calculated for 1/2C_36_H_32_N_6_O_4_Se_2_ 387.0481; found 387.0471.

#### 3.2.6 4,4′-((4*Z*,4′*Z*)-2,2′-(Ethane-1,2-diylbis(selanediyl))bis(5-oxo-4-(pyridin-2-ylmethylene)-4,5-dihydro-1*H*-imidazole-2,1-diyl))dibenzonitrile (4b)

In a solution of 160 mg of (3b) in 3 ml of DMF with 43 mg of 1,2-dibromoethane and 94 mg K_2_CO_3_. Yellow solid. Yield: 132 mg, 80%. mp 204–205 °C. ^1^H NMR (400 MHz, CDCl_3_) *δ* = 4.02 (s, 4H, CH2), 4.25 (dt, *J* = 5.6, 1.5, 4H, CH2Alyl), 5.26–5.31 (m, 4H, CH2Alyl), 5.78–5.87 (m, 2H, CH Alyl), 7.08 (s, 2H, CH), 7.31–7.34 (m, 2H, HPy), 7.76 (t, *J* = 7.8, 2H, HPy), 8.66 (dt, *J* = 8.1, 1.0, 2H, HPy), 8.74 (d, *J* = 4.4, 2H, HPy). ^13^C {^1^H} NMR (101 MHz, DMSO-d6) *δ* = 21.7 (2C), 26.5, 55.4, 114.0 (2C), 125.6, 126.5, 128.2, 130.1 (2C), 159.3, 162.4, 178.2. FTIR (Diamond, cm^−1^): 544, 737, 745, 777, 838, 921, 946, 993, 1048, 1089, 1148, 1161, 1191, 1265, 1225, 1283, 1300, 1415, 1355, 1432, 1506, 1473, 1583, 1604, 1640, 1718, 2227, 2998, 3049. HRMS (FTMS + cESI) *m*/*z*: [M + H]^+^, calculated for C_34_H_23_N_8_O_2_Se_2_ 735.0269; found 735.0262.

#### 3.2.7 (4*Z*,4′*Z*)-2,2′-(Ethane-1,2-diylbis(selanediyl))bis(1-(4-chlorophenyl)-4-(pyridin-2-ylmethylene)-1*H*-imidazol-5(4*H*)-one) (4c)

In a solution of 250 mg of (3c) in 6 ml of DMF with 38 mg of 1,2-dibromoethane and 250 mg Cs_2_CO_3_. Yellow solid. Yield: 400 mg, 77%. mp 210–211 °C. ^1^H NMR (400 MHz, CDCl_3_) *δ* = 3.93 (s, 4H, CH_2_), 7.17 (s, 2H, CH), 7.22 (ddd, *J* = 7.5, 4.9, 0.9 Hz, 2H, H_Py_), 7.31 (d, *J* = 8.7 Hz, 4H, H_Ar_), 7.50 (d, *J* = 8.6 Hz, 4H, H_Ar_), 7.60 (td, *J* = 7.8, 1.7 Hz, 2H, H_Py_), 8.66–8.70 (m, 4H, H_Py_). ^13^C {^1^H} NMR (101 MHz, CDCl_3_) *δ* = 27.0 (2C), 123.7 (2C), 125.9 (2C), 127.1 (2C), 128.4 (4C), 130.2 (4C), 131.2 (2C), 135.7 (2C), 136.1 (2C), 139.9 (2C), 150.3 (2C), 153.3 (2C), 162.6 (2C), 168.1 (2C). FTIR (Diamond, cm^−1^): 573, 622, 670, 737, 753, 780, 829, 920, 949, 990, 1016, 1048, 1093, 1148, 1161, 1200, 1223, 1263, 1277, 1298, 1338, 1353, 1407, 1430, 1465, 1494, 1563, 1580, 1627, 1724, 3019. HRMS (FTMS + cESI) *m*/*z*: [M + H]^+^, calculated for C_32_H_23_Cl_2_N_6_O_2_Se_2_ 752.9584; found 752.9588.

#### 3.2.8 (4*Z*,4′*Z*)-2,2′-(Ethane-1,2-diylbis(selanediyl))bis(1-allyl-4-(pyridin-2-ylmethylene)-1*H*-imidazol-5(4*H*)-one) (4d)

In a solution of 200 mg of (3d) in 3 ml of DMF with 64 mg of 1,2-dibromoethane and 141 mg K_2_CO_3_. Yellow solid. Yield: 133 mg, 64%. mp 156–157 °C. ^1^H NMR (400 MHz, CDCl_3_) *δ* = 3.96 (s, 4H, CH_2_), 4.25 (d, *J* = 5.6 Hz, 4H, CH_2Alyl_), 5.28 (dd, *J* = 13.6, 2.9 Hz, 4H, CH_2Alyl_), 5.84 (ddd, *J* = 15.7, 10.6, 5.6 Hz, 2H, CH_Alyl_), 7.08 (s, 2H, CH), 7.19 (dd, *J* = 7.1, 5.0 Hz, 2H, H_Py_), 7.61 (td, *J* = 7.8, 1.8 Hz, 2H, H_Py_), 8.63 (dd, *J* = 4.8, 0.9 Hz, 2H, H_Py_), 8.67 (d, *J* = 8.0 Hz, 2H, H_Py_). ^13^C {^1^H} NMR (101 MHz, CDCl_3_) *δ* = 26.9, 43.8, 119.0, 123.5, 125.1, 127.1, 131.4, 136.0, 140.3, 150.1, 153.6, 163.4, 168.9. FTIR (Diamond, cm^−1^): 542, 572, 621, 721, 742, 790, 895, 906, 923, 971, 988, 1086, 1125, 1144, 1172, 1185, 1221, 1263, 1307, 1337, 1353, 1402, 1432, 1476, 1560, 1577, 1635, 1718, 2995, 3041. HRMS (FTMS + cESI) *m*/*z*: [M + H]^+^, calculated for C_26_H_25_N_6_O_2_Se_2_ 613.0364; found 613.0337.

#### 3.2.9 (4*Z*,4′*Z*)-2,2′-(Ethane-1,2-diylbis(selanediyl))bis(1-cyclopropyl-4-(pyridin-2-ylmethylene)-1*H*-imidazol-5(4*H*)-one) (4e)

In a solution of 70 mg of (3e) in 3 ml of DMF with 19 mg of 1,2-dibromoethane and 43 mg K_2_CO_3_. Yellow solid. Yield: 42 mg, 58%. mp 200–201 °C. ^1^H NMR (400 MHz, DMSO-d6) *δ* = 0.94 (m, 8H, H_Cyclo_), 2.78 (tt, *J* = 6.9, 3.6, 2H, H_Cyclo_), 3.91 (s, 4H, CH_2_), 6.49 (s, 2H, CH), 7.25 (dd, *J* = 6.8, 5.3, 2H, H_Py_), 7.58 (t, *J* = 7.5, 2H, H_Py_), 8.53 (d, *J* = 4.3, 2H, H_Py_), 8.61 (d, *J* = 8.0, 2H, H_Py_). ^13^C {^1^H} NMR (101 MHz, DMSO-d6) *δ* = 21.7 (2C), 26.5, 55.4, 114.0 (2C), 125.6, 126.5, 128.2, 130.1 (2C), 159.3, 162.4, 178.2. FTIR (Diamond, cm^−1^): 537, 576, 623, 677, 695, 733, 773, 861, 891, 929, 980, 943, 1035, 1084, 1179, 1222, 1321, 1356, 1373, 1403, 1427, 1413, 1568, 1453, 1638, 1702, 2934, 2982, 3023, 3257, 3393. HRMS (FTMS + cESI) *m*/*z*: [M + H]^+^, calculated for C_26_H_25_N_6_O_2_Se_2_ 613.0364; found 613.0382.

#### 3.2.10 (4*Z*,4′*Z*)-2,2′-(Butane-1,4-diylbis(selanediyl))bis(1-allyl-4-(pyridin-2-ylmethylene)-1*H*-imidazol-5(4*H*)-one) (4f)

In a solution of 250 mg of (3f) in 3 ml of DMF with 93 mg of 1,4-dibromobutane and 177 mg K_2_CO_3_. Yellow solid. Yield: 150 mg, 55%. mp 182–183 °C. ^1^H NMR (400 MHz, CDCl_3_) *δ* = 2.12 (s, 4H, CH_2_), 3.46 (t, *J* = 5.2, 4H, CH_2_), 4.17 (d, *J* = 4.5, 4H, H_Alyl_), 5.21 (d, *J* = 6.3, 2H, H_Alyl_), 5.24 (s, 2H, H_Alyl_), 5.77 (ddt, *J* = 15.8, 10.2, 5.2, 2H, H_Alyl_), 7.07 (s, 2H, CH), 7.15–7.18 (m, 2H, H_Py_), 7.61 (t, *J* = 7.6, 2H, H_Py_), 8.62 (d, *J* = 4.0, 2H, H_Py_), 8.71 (d, *J* = 7.9, 2H, H_Py_). ^13^C {^1^H} NMR (101 MHz, CDCl_3_) *δ* = 26.8 (2C), 30.4 (2C), 43.7 (2C), 118.9 (2C), 122.7 (2C), 123.5 (2C), 127.1 (2C), 131.2 (2C), 136.8 (2C), 141.0 (2C), 149.3 (2C), 153.1 (2C), 165.2 (2C), 168.8 (2C). FTIR (KBr, cm^−1^): 553, 624, 653, 743, 791, 887, 929, 966, 990, 1084, 1116, 1141, 1180, 1224, 1261, 1307, 1350, 1435, 1480, 1562, 1581, 1642, 1707, 2849, 2906. HRMS (FTMS + cESI) *m*/*z*: [M + H]^+^, calculated for C_28_H_29_N_6_O_2_Se_2_ 641.0677; found 641.0649.

#### 3.2.11 (4*Z*,4′*Z*)-2,2′-(Butane-1,4-diylbis(selanediyl))bis(1-cyclopropyl-4-(pyridin-2-ylmethylene)-1*H*-imidazol-5(4*H*)-one) (4g)

In a solution of 200 mg of (3g) in 3 ml of DMF with 74 mg of 1,4-dibromobutane and 143 mg K_2_CO_3_. Yellow solid. Yield: 127 mg, 65%. mp 219–220 °C. ^1^H NMR (400 MHz, CDCl_3_) *δ* = 0.9–0.96 (m, 8H, H_Cyclo_), 2.08–2.10 (m, 4H, CH_2_), 2.55 (dq, *J* = 6.8, 4.0 Hz, 2H, H_Cyclo_), 3.38 (t, *J* = 6.5 Hz, 4H, CH_2_), 6.91 (s, 2H, CH), 7.18 (ddd, *J* = 7.4, 5.0, 1.0 Hz, 2H, H_Py_), 7.64 (td, *J* = 7.8, 1.6 Hz, 2H, H_Py_), 8.57–8.59 (m, 2H, H_Py_), 8.67 (d, *J* = 8.1 Hz, 2H, H_Py_). ^13^C {^1^H} NMR (101 MHz, CDCl_3_) *δ* = 6.4 (4C), 22.5 (2C), 26.5 (2C), 30.3 (2C), 121.1 (2C), 123.6 (2C), 127.1 (2C), 137.3 (2C), 141.7 (2C), 148.8 (2C), 152.7 (2C), 168.1 (2C), 169.4 (2C). FTIR (Diamond, cm^−1^): 454, 541, 557, 624, 654, 699, 732, 741, 792, 832, 887, 956, 989, 1006, 1032, 1078, 1145, 1209, 1236, 1264, 1333, 1359, 1371, 1434, 1464, 1486, 1562, 1578, 1639, 1712, 1741, 3050. HRMS (FTMS + cESI) *m*/*z*: [M + H]^+^, calculated for C_28_H_29_N_6_O_2_Se_2_ 641.0677; found 641.0681.

#### 3.2.12 (4*Z*,4′*Z*)-2,2′-(Hexane-1,6-diylbis(selanediyl))bis(1-allyl-4-(pyridin-2-ylmethylene)-1*H*-imidazol-5(4*H*)-one) (4h)

In a solution of 250 mg of (3h) in 3 ml of DMF with 105 mg of 1,6-dibromohexane and 279 mg K_2_CO_3_. Yellow solid. Yield: 150 mg, 41%. mp 123–124 °C. ^1^H NMR (400 MHz, CDCl_3_) *δ* = 1.57 (s, 4H, CH_2_), 1.96 (s, 4H, CH_2_), 3.41 (t, *J* = 6.7 Hz, 4H, CH_2_), 4.21 (d, *J* = 5.2 Hz, 4H, CH_2Alyl_), 5.22–5.27 (m, 4H, CH_2Alyl_), 5.80 (ddd, *J* = 15.7, 10.7, 5.4 Hz, 2H, CH_Alyl_), 7.17 (s, 2H, CH), 7.31 (s, 2H, H_Py_), 7.83 (t, *J* = 6.7 Hz, 2H, H_Py_), 8.72 (s, 2H, H_Py_), 8.82 (d, *J* = 7.6 Hz, 2H, H_Py_). ^13^C {^1^H} NMR (101 MHz, CDCl_3_) *δ* = 27.7 (2C), 29.5 (2C), 29.9 (2C), 43.7 (2C), 118.7 (2C), 123.1 (2C), 123.3 (2C), 127.1 (2C), 131.3 (2C), 136.5 (2C), 140.9 (2C), 149.6 (2C), 153.6 (2C), 165.1 (2C), 168.9 (2C). FTIR (KBr, cm^−1^): 404, 456, 512, 540, 569, 623, 641, 669, 714, 725, 740, 754, 769, 839, 784, 891, 902, 910, 934, 968, 1022, 1050, 989, 1090, 1125, 1176, 1222, 1255, 1297, 1311, 1335, 1352, 1403, 1362, 1427, 1435, 1465, 1563, 1478, 1581, 1634, 1717, 2857, 2913. HRMS (FTMS + cESI) *m*/*z*: [M + H]^+^, calculated for C_30_H_33_N_6_O_2_Se_2_ 669.0990; found 669.0983.

#### 3.2.13 (4*Z*,4′*Z*)-2,2′-(Hexane-1,6-diylbis(selanediyl))bis(1-cyclopropyl-4-(pyridin-2-ylmethylene)-1*H*-imidazol-5(4*H*)-one) (4i)

In a solution of 300 mg of (3i) in 3 ml of DMF with 126 mg of 1,6-dibromohexane and 210 mg K_2_CO_3_. Yellow solid. Yield: 203 mg, 89%. mp 189–190 °C. ^1^H NMR (400 MHz, CDCl_3_) *δ* = 0.98–1.00 (m, 8H, H_Cyclo_), 1.57–1.59 (m, 4H, CH_2_), 1.95–1.98 (m, 4H, CH_2_), 2.59–2.64 (m, 2H, H_Cyclo_), 3.33 (t, *J* = 7.1 Hz, 4H, CH_2_), 7.04 (s, 2H, CH), 7.17–7.20 (m, 2H, H_Py_), 7.69 (t, *J* = 7.6 Hz, 2H, H_Py_), 8.64 (d, *J* = 4.2 Hz, 2H, H_Py_), 8.75 (d, *J* = 7.9 Hz, 2H, H_Py_). ^13^C {^1^H} NMR (101 MHz, CDCl_3_) *δ* = 6.5 (4C), 22.6 (2C), 27.3 (2C), 29.6 (2C), 29.8 (2C), 122.1 (2C), 123.2 (2C), 127.0 (2C), 136.7 (2C), 141.6 (2C), 149.2 (2C), 153.5 (2C), 167.8 (2C), 169.5 (2C). FTIR (Diamond, cm^−1^): 552, 623, 733, 791, 825, 894, 988, 1006, 1027, 1050, 1075, 1142, 1158, 1172, 1209, 1235, 1266, 1332, 1358, 1371, 1435, 1463, 1486, 1562, 1577, 1636, 1704, 2861, 2932, 3049. HRMS (FTMS + cESI) *m*/*z*: [M + H]^+^, calculated for C_30_H_33_N_6_O_2_Se_2_ 669.0990; found 669.0979.

#### 3.2.14 (4*Z*,4′*Z*)-2,2′-(Decane-1,10-diylbis(selanediyl))bis(1-allyl-4-(pyridin-2-ylmethylene)-1*H*-imidazol-5(4*H*)-one) (4j)

In a solution of 200 mg of (3j) in 3 ml of DMF with 103 mg of 1,10-dibromodecane and 142 mg K_2_CO_3_. Yellow solid. Yield: 91 mg, 37%. mp 85–86 °C. ^1^H NMR (400 MHz, CDCl_3_) *δ* = 1.25–1.49 (m, 12H, CH_2_), 1.87–1.94 (m, 4H, CH_2_), 3.39 (t, *J* = 7.1, 4H, CH_2_), 4.23 (d, *J* = 5.5, 4H, CH_2Alyl_), 5.23–5.27 (m, 4H, CH_2Alyl_), 5.81 (ddt, *J* = 17.2, 10.3, 5.5, 2H, CH_Alyl_), 7.17 (s, 2H, CH), 7.31–7.34 (m, 2H, H_Py_), 7.84 (t, *J* = 7.2, 2H, H_Py_), 8.71 (d, *J* = 3.1, 2H, H_Py_), 8.88 (d, *J* = 8.0, 2H, H_Py_). ^13^C {^1^H} NMR (101 MHz, CDCl_3_) *δ* = 28.0 (2C), 29.2 (2C), 29.6 (2C), 30.0 (2C), 30.1 (2C), 43.7 (2C), 118.7 (2C), 122.7 (2C), 123.3 (2C), 127.2 (2C), 131.4 (2C), 136.7 (2C), 141.1 (2C), 149.3 (2C), 153.6 (2C), 165.5 (2C), 168.94 (2C). FTIR (Diamond, cm^−1^): 456, 545, 623, 666, 719, 740, 783, 885, 901, 924, 961, 990, 1088, 1106, 1142, 1193, 1224, 1263, 1306, 1339, 1371, 1406, 1431, 1466, 1481, 1562, 1583, 1635, 1714, 2850, 2917, 2996, 3047. HRMS (FTMS + cESI) *m*/*z*: [M + H]^+^, calculated for C_34_H_40_N_6_O_2_Se_2_ 725.1616; found 725.1577.

#### 3.2.15 (4*Z*,4′*Z*)-2,2′-(Decane-1,10-diylbis(selanediyl))bis(1-cyclopropyl-4-(pyridin-2-ylmethylene)-1*H*-imidazol-5(4*H*)-one) (4k)

In a solution of 200 mg of (3k) in 3 ml of DMF with 103 mg of 1,10-dibromodecane and 142 mg K_2_CO_3_. Yellow solid. Yield: 102 mg, 41%. mp 157–158 °C. ^1^H NMR (400 MHz, CDCl_3_) *δ* = 1.00–1.02 (m, 8H, CH_2_), 1.25–1.35 (m, 8H, CH_2_), 1.43–1.48 (m, 4H, CH_2_), 1.87–1.94 (m, 4H, H_Cyclo_), 2.61–2.66 (m, 2H, H_Cyclo_), 3.35 (t, *J* = 7.2, 4H, CH_2_), 7.11 (s, 2H, CH), 7.35–7.38 (m, 2H, H_Py_), 7.88 (t, *J* = 7.6, 2H, H_Py_), 8.71 (d, *J* = 4.4, 2H, H_Py_), 8.88 (d, *J* = 8.1, 2H, H_Py_). ^13^C {^1^H} NMR (101 MHz, CDCl_3_) *δ* = 6.5 (4C), 22.7 (2C), 27.7 (2C), 29.2 (2C), 29.6 (2C), 29.9 (2C), 30.2 (2C), 121.5 (2C), 123.3 (2C), 127.2 (2C), 137.0 (2C), 141.8 (2C), 148.9 (2C), 153.4 (2C), 168.2 (2C), 169.4 (2C). FTIR (Diamond, cm^−1^): 426, 448, 477, 534, 545, 592, 616, 652, 689, 725, 768, 815, 783, 884, 941, 977, 995, 1016, 1065, 1038, 1129, 1163, 1196, 1206, 1249, 1223, 1281, 1314, 1354, 1414, 1445, 1464, 1543, 1560, 1617, 1692, 2820, 2888, 3010. HRMS (FTMS + cESI) *m*/*z*: [M + H]^+^, calculated for C_34_H_41_N_6_O_2_Se_2_ 725.1616; found 725.1618.

#### 3.2.16 General procedure for coordination compounds (5) and (6) preparation

0.1 ml of *n*-BuOH was carefully added to a solution of 15 mg of the corresponding ligand 4 in 1 ml of dichloromethane to achieve separated layers. A solution of 2 eq. of CuCl_2_·2H_2_O or Cu(ClO_4_)_2_·6H_2_O in 1 ml of *n*-BuOH was then carefully added, keeping the separation in a two-phase system. Tightly closed reaction mixture was left for 1–2 days in the dark place until homogenous solution formed. Crystallization was activated by ether diffusion: an open vial with a reaction solution was placed in a larger vial containing a small amount of diethyl ether, tightly closed and left to stand for 24 hours in the dark.

The precipitate was separated by decanting, washed with a small amount of ice-cold dichloromethane and then by diethyl ether until the washing solvent become colorless. The final products were obtained as crystalline powders after drying on air.

#### 3.2.17 (4*Z*,4′*Z*)-2,2′-(Ethane-1,2-diylbis(selanediyl))bis(1-(4-ethoxyphenyl)-4-(pyridin-2-ylmethylene)-1*H*-imidazol-5(4*H*)-one)dicopper(i,ii) trichloride (5a). Dark brown crystals

Yield 32%. Mp 158–159 °C. FTIR (Diamond, cm^−1^): 773, 834, 1050, 1130, 1180, 1232, 1259, 1280, 1302, 1396, 1410, 1440, 1484, 1517, 1552, 1600, 1615, 1640, 1694, 1748, 2878, 2990, 3072, 3428. MALDI *m*/*z*: 741.1 [L + Cu]^+^. Elemental analysis: calculated for C_36_H_32_Cl_3_Cu_2_N_6_O_4_Se_2_: C, 43.07; H, 3.21; N, 8.37. Found: C, 42.87; H, 3.34; N, 8.38.

#### 3.2.18 (4*Z*,4′*Z*)-2,2′-(Ethane-1,2-diylbis(selanediyl))bis(1-(4-cyanophenyl)-4-(pyridin-2-ylmethylene)-1*H*-imidazol-5(4*H*)-one)dicopper(i) dichloride (5b)

Dark red crystals. Yield 71%. Mp 268–269 °C. FTIR (Diamond, cm^−1^): 546, 689, 749, 773, 837, 954, 1130, 1190, 1168, 1240, 1279, 1320, 1397, 1441, 1487, 1515, 1607, 1645, 1699, 1748, 2231, 3053, 3539.

#### 3.2.19 (4*Z*,4′*Z*)-2,2′-(Ethane-1,2-diylbis(selanediyl))bis(1-(4-chloro-phenyl)-4-(pyridin-2-ylmethylene)-1*H*-imidazol-5(4*H*)-one)dicopper(i) dichloride (5c)

Dark red crystals. Yield 51%. Mp 255–259 °C. FTIR (Diamond, cm^−1^): 592, 746, 969, 1017, 1090, 1164, 1214, 1255, 1313, 1349, 1449, 1494, 1587, 1653, 1733, 3060. MALDI *m*/*z*: 885.8 [L + CuCl_2_ + H]^+^.

#### 3.2.20 (4*Z*,4′*Z*)-2,2′-(Ethane-1,2-diylbis(selanediyl))bis(1-(4-allyl)-4-(pyridin-2-ylmethylene)-1*H*-imidazol-5(4*H*)-one)dicopper(i) dichloride (5d)

Dark red crystals. Yield 53%. Mp 183–184 °C. FTIR (Diamond, cm^−1^): 778, 1175, 1249, 1312, 1367, 1413, 1439, 1482, 1553, 1628, 1699, 1733, 2933, 3428. MALDI *m*/*z*: 675.4 [L + Cu]^+^. HRMS (FTMS + cESI) *m*/*z*: [L + Cu]^+^ calculated for C_26_H_24_CuN_6_O_2_Se_2_ 674.9582; found 674.9602.

#### 3.2.21 (4*Z*,4′*Z*)-2,2′-(Ethane-1,2-diylbis(selanediyl))bis(1-(4-cyclopropyl)-4-(pyridin-2-ylmethylene)-1*H*-imidazol-5(4*H*)-one)dicopper(i,ii) trichloride (5e)

Dark reddish-brown crystals. Yield 44%. Mp 174–175 °C. FTIR (Diamond, cm^−1^): 576, 736, 781, 831, 885, 925, 1033, 1094, 1160, 1222, 1243, 1271, 1315, 1349, 1384, 1444, 1483, 1454, 1594, 1658, 1739, 3027, 3062, 3097, 3460, 3525. MALDI *m*/*z*: 674.9 [L + Cu]^+^. HRMS (FTMS + cESI) *m*/*z*: [L + Cu]^+^ calculated for C_26_H_24_CuN_6_O_2_Se_2_ 674.9587; found 674.9600.

#### 3.2.22 (4*Z*,4′*Z*)-2,2′-(Butane-1,4-diylbis(selanediyl))bis(1-allyl-4-(pyridin-2-ylmethylene)-1*H*-imidazol-5(4*H*)-one)dicopper(i,ii) trichloride (5f)

Dark green crystals. Yield 66%. Mp 180–181 °C. FTIR (Diamond, cm^−1^): 644, 685, 779, 1174, 1275, 1310, 1397, 1436, 1482, 1552, 1599, 1634, 1699, 1738, 2934, 3428. Elemental analysis: calculated for C_30_H_36_Cl_2_Cu_2_N_6_O_2_Se_2_: C, 41.49; H, 4.18; N, 9.68. Found: C, 41.14; H, 4.29; N, 9.49.

#### 3.2.23 (4*Z*,4′*Z*)-2,2′-(Butane-1,4-diylbis(selanediyl))bis(1-cyclopropyl-4-(pyridin-2-ylmethylene)-1*H*-imidazol-5(4*H*)-one)dicopper(i,ii) trichloride (5g)

Dark reddish-brown crystals. Yield 26%. Mp 177–178 °C.). FTIR (Diamond, cm^−1^): 576, 733, 776, 1011, 1034, 1086, 1161, 1218, 1266, 1311, 1441, 1596, 1631, 1733, 2869, 2933, 3024, 3295. Elemental analysis: calculated for C_28_H_28_Cl_3_Cu_2_N_6_O_2_Se_2_: C, 38.57; H, 3.24; N, 9.64. Found: C, 38.17; H, 3.42; N, 9.44.

#### 3.2.24 (4*Z*,4′*Z*)-2,2′-(Hexane-1,6-diylbis(selanediyl))bis(1-allyl-4-(pyridin-2-ylmethylene)-1*H*-imidazol-5(4*H*)-one)dicopper(i,ii) trichloride (5h)

Dark reddish-brown crystals. Yield 27%. Mp 153–154 °C. FTIR (Diamond, cm^−1^): 645, 779, 913, 935, 974, 1017, 1189, 1244, 1311, 1440, 1481, 1553, 1600, 1627, 1733, 2859, 2933, 3293. MALDI *m*/*z*: 730.4 [L + Cu]^+^. Elemental analysis: calculated for C_30_H_32_Cl_3_Cu_2_N_6_O_2_Se_2_: C, 40.04; H, 3.58; N, 9.34. Found: C, 39.81; H, 3.49; N, 9.18.

#### 3.2.25 (4*Z*,4′*Z*)-2,2′-(Hexane-1,6-diylbis(selanediyl))bis(1-cyclopropyl-4-(pyridin-2-ylmethylene)-1*H*-imidazol-5(4*H*)-one)dicopper(ii) tetrachloride (5i)

Dark brown crystals. Yield 50%. Mp 188–189 °C. FTIR (Diamond, cm^−1^): 576, 734, 782, 839, 920, 1038, 1092, 1161, 1221, 1265, 1309, 1347, 1434, 1482, 1594, 1661, 1744, 1978, 2850, 2930, 3021, 3101. MALDI *m*/*z*: 730.2 [L + Cu]^+^. HRMS (FTMS + cESI) *m*/*z*: [L + Cu]^+^ calculated for C_30_H_32_CuN_6_O_2_Se_2_ 731.0208; found 731.0232.

#### 3.2.26 (4*Z*,4′*Z*)-2,2′-(Decane-1,10-diylbis(selanediyl))bis(1-allyl-4-(pyridin-2-ylmethylene)-1*H*-imidazol-5(4*H*)-one)dicopper(i,ii) trichloride (5j)

Dark brown crystals. Yield 33%. Mp 152–154 °C. FTIR (Diamond, cm^−1^): 617, 646, 779, 1111, 1173, 1409, 1438, 1599, 1630, 1737, 2032, 2850, 2924, 3181, 3285. HRMS (FTMS + cESI) *m*/*z*: [L + Cu]^+^ calculated for C_26_H_24_CuN_6_O_2_Se_2_ 946,0796; found 946.0793.

#### 3.2.27 (4*Z*,4′*Z*)-2,2′-(Decane-1,10-diylbis(selanediyl))bis(1-cyclopropyl-4-(pyridin-2-ylmethylene)-1*H*-imidazol-5(4*H*)-one)dicopper(ii) tetrachloride (5k)

Dark reddish-brown crystals. Yield 39%. Mp 135–136 °C. FTIR (Diamond, cm^−1^): 572, 775, 827, 867, 957, 1013, 1045, 1091, 1163, 1220, 1268, 1310, 1340, 1376, 1436, 1477, 1592, 1652, 1743, 2853, 2925, 3022, 3092. MALDI *m*/*z*: 886.3. HRMS (FTMS + cESI) *m*/*z*: [L+2Cu + Cl]^+^ calculated for C_34_H_40_ClCu_2_N_6_O_2_Se_2_ 884,9824; found 884.9808.

#### 3.2.28 (4*Z*,4′*Z*)-2,2′-(Ethane-1,2-diylbis(selanediyl))bis(1-(4-ethoxyphenyl)-4-(pyridin-2-ylmethylene)-1*H*-imidazol-5(4*H*)-one)copper(ii) diperchlorate (6a)

Was not isolated; the formation was established based on an electrochemical study data.

#### 3.2.29 (4*Z*,4′*Z*)-2,2′-(Ethane-1,2-diylbis(selanediyl))bis(1-(4-cyanophenyl)-4-(pyridin-2-ylmethylene)-1*H*-imidazol-5(4*H*)-one)copper(i) perchlorate (6b)

Dark red crystals. Yield 39%. Mp 135–136 °C. FTIR (Diamond, cm^−1^): 576, 736, 781, 831, 885, 925, 1033, 1094, 1160, 1222, 1243, 1271, 1315, 1349, 1384, 1444, 1483, 1454, 1594, 1658, 1739, 3027, 3062, 3097, 3460, 3525. MALDI (ESI) *m*/*z*: 675.3 [L + Cu]^+^. Elemental analysis: calculated for C_34_H_22_ClCuN_8_O_6_Se_2_: C, 45.60; H, 2.48; N, 12.51. Found: C, 45.06H, 2.73 N, 12.18.

#### 3.2.30 (4*Z*,4′*Z*)-2,2′-(Ethane-1,2-diylbis(selanediyl))bis(1-(4-chlorphenyl)-4-(pyridin-2-ylmethylene)-1*H*-imidazol-5(4*H*)-one)copper(i) perchlorate (6c)

Dark red crystals, Yield 34%. Mp 259–262 °C. FTIR (Diamond, cm^−1^): 622, 744, 778, 833, 959, 1090, 1165, 1217, 1247, 1406, 1462, 1494, 1632, 1697, 1737, 2855, 2930, 3061, 3097. MALDI *m*/*z*: 814.6 [L + Cu]^+^. HRMS (FTMS + cESI) *m*/*z*: [L + Cu]^+^ calculated for C_32_H_22_Cl_2_CuN_6_O_2_Se_2_ 814.8808; found 814.8836. Elemental analysis: calculated for C_32_H_22_C_3_lCuN_6_O_6_Se_2_: C, 42.03; H, 2.43; N, 9.19. Found: C, 41.77, 2.50 N, 9.12.

#### 3.2.31 (4*Z*,4′*Z*)-2,2′-(Ethane-1,2-diylbis(selanediyl))bis(1-(4-allyl)-4-(pyridin-2-ylmethylene)-1*H*-imidazol-5(4*H*)-one)copper(ii) diperchlorate (6d)

Dark brown crystals, Yield 53%. Mp 183–184 °C. FTIR (Diamond, cm^−1^): 621, 777, 928, 1089, 1188, 1238, 1291, 1360, 1462, 1643, 1727, 2013, 2315, 2869, 2930, 2957, 3067, 3098, 3247, 3536. HRMS (FTMS + cESI) *m*/*z*: [L + Cu]^+^ calculated for C_26_H_24_CuN_6_O_2_Se_2_ 674,9587; found 674.9601. Elemental analysis: calculated for C_32_H_22_Cl_2_CuN_6_O_10_Se_2_: C, 35.78; H, 2.77; N, 9.63. Found: C, 35.36H, 2.55 N, 9.68.

#### 3.2.32 (4*Z*,4′*Z*)-2,2′-(Ethane-1,2-diylbis(selanediyl))bis(1-(4-allyl)-4-(pyridin-2-ylmethylene)-1*H*-imidazol-5(4*H*)-one)copper(ii) diperchlorate (6e)

Dark red crystals, Yield 47%. Mp 174–175 °C. FTIR (Diamond, cm^−1^): 571, 622, 735, 780, 1089, 1159, 1220, 1260, 1306, 1345, 1374, 1434, 1470, 1587, 1641, 1728, 3016, 3527. HRMS (FTMS + cESI) *m*/*z*: [L + Cu]^+^ calculated for C_26_H_24_CuN_6_O_2_Se_2_ 674,9582; found 674.9608. Elemental analysis: calculated for C_26_H_24_ClCuN_6_O_6_Se_2_: C, 40.38; H, 3.13; N, 10.87. Found: C, 40.18 H, 3.57 N, 10.39.

#### 3.2.33 (4*Z*,4′*Z*)-2,2′-(Butane-1,4-diylbis(selanediyl))bis(1-allyl-4-(pyridin-2-ylmethylene)-1*H*-imidazol-5(4*H*)-one)copper(ii) diperchlorate (6f)

Dark brown crystals, Yield 66%. Mp 180–181 °C. FTIR (Diamond, cm^−1^): 623, 778, 847, 1092, 1394, 1437, 1555, 1604, 1628, 1731, 1974, 2041, 2160, 2231, 2361, 2450, 2568, 2868, 2956, 3376, 3523. HRMS (FTMS + cESI) *m*/*z*: [L + Cu]^+^ calculated for C_28_H_28_CuN_6_O_2_Se_2_ 702,9900; found 702.9882. Elemental analysis: calculated for C_28_H_28_Cl_2_CuN_6_O_10_Se_2_: C, 37.33; H, 3.13; N, 9.33. Found: C, 37.36 H, 3.19; N, 9.29.

#### 3.2.34 (4*Z*,4′*Z*)-2,2′-(Butane-1,4-diylbis(selanediyl))bis(1-cyclopropyl-4-(pyridin-2-ylmethylene)-1*H*-imidazol-5(4*H*)-one)copper(ii) diperchlorate (6g)

Dark brown crystals, Yield 56%. Mp 211–212 °C. FTIR (Diamond, cm^−1^): 544, 565, 605, 622, 659, 713, 726, 746, 781, 833, 879, 904, 952, 1035, 1085, 1158, 1202, 1220, 1252, 1306, 1262, 1341, 1375, 1421, 1433, 1465, 1559, 1588, 1647, 1731, 2924, 3019, 3438. HRMS (FTMS + cESI) *m*/*z*: [L + Cu]^+^ calculated for C_28_H_28_CuN_6_O_2_Se_2_ 702,9895; found 702,9890. Elemental analysis: calculated for C_28_H_28_Cl_2_CuN_6_O_10_Se_2_: C, 37.33; H, 3.13; N, 9.33. Found: C, 37.25; H, 3.02; N, 9.59.

#### 3.2.35 (4*Z*,4′*Z*)-2,2′-(Hexane-1,6-diylbis(selanediyl))bis(1-allyl-4-(pyridin-2-ylmethylene)-1*H*-imidazol-5(4*H*)-one)copper(ii) diperchlorate (6h)

Dark brown crystals, Yield 37%. Mp 166–167 °C. FTIR (Diamond, cm^−1^): 622, 780, 928, 1093, 1190, 1241, 1311, 1362, 1462, 1557, 1635, 1729, 2859, 2930, 3101, 3502, 3585, HRMS (FTMS + cESI) *m*/*z*: [L + Cu]^+^ calculated for C_30_H_32_CuN_6_O_2_Se_2_ 731,0202; found 731,0192.

#### 3.2.36 (4*Z*,4′*Z*)-2,2′-(Hexane-1,6-diylbis(selanediyl))bis(1-cyclopropyl-4-(pyridin-2-ylmethylene)-1*H*-imidazol-5(4*H*)-one)copper(ii) diperchlorate (6i)

Dark brown crystals, Yield 50%. Mp 188–189 °C. FTIR (Diamond, cm^−1^): 576, 736, 781, 831, 885, 925, 1033, 1094, 1160, 1222, 1243, 1271, 1315, 1349, 1384, 1444, 1483, 1454, 1594, 1658, 1739, 3027, 3062, 3097, 3460, 3525. MALDI (ESI) *m*/*z*: 730.2. HRMS (FTMS + cESI) *m*/*z*: [L + Cu + H]^+^ calculated for C_26_H_24_CuN_6_O_2_Se_2_ 674,9587; found 674.9600.

#### 3.2.37 (4*Z*,4′*Z*)-2,2′-(Decane-1,10-diylbis(selanediyl))bis(1-allyl-4-(pyridin-2-ylmethylene)-1*H*-imidazol-5(4*H*)-one)dicopper(i,i) trichloride (6j)

Dark brown crystals, Yield 30%. Mp 176–179 °C. FTIR (Diamond, cm^−1^): 621, 779, 1092, 1162, 1248, 1310, 1362, 1439, 1555, 1634, 1732, 2853, 2927, 3457. HRMS (FTMS + cESI) *m*/*z*: [L + Cu]^+^ calculated for C_34_H_40_CuN_6_O_2_Se_2_ 787,0834; found 787,0842.

#### 3.2.38 (4*Z*,4′*Z*)-2,2′-(Decane-1,10-diylbis(selanediyl))bis(1-cyclopropyl-4-(pyridin-2-ylmethylene)-1*H*-imidazol-5(4*H*)-one)dicopper(i,ii) trichloride (6k)

Dark brown crystals, Yield 39%. Mp 136–137 °C.). FTIR (Diamond, cm^−1^): 576, 736, 781, 831, 885, 925, 1033, 1094, 1160, 1222, 1243, 1271, 1315, 1349, 1384, 1444, 1483, 1454, 1594, 1658, 1739, 3027, 3062, 3097, 3460, 3525. MALDI (ESI) *m*/*z*: 886.3. HRMS (FTMS + cESI) *m*/*z*: [L + Cu + H]^+^ calculated for C_26_H_24_CuN_6_O_2_Se_2_ 674,9587; found 674.9600.

## 4. Conclusions

In conclusion, a series of novel organic ligands 4 with two 5-(2-pyridylmethylene)-3,5-dihydro-4*H*-imidazol-4-one moieties were firstly synthesized and their interaction with CuCl_2_·2H_2_O and Cu(ClO_4_)_2_·6H_2_O was studying. It should be noted that, in reactions with CuCl_2_ and Cu(ClO_4_)_2_, coordination compounds of various types are formed (binuclear complexes with coordination of copper by chloride anions in the case of CuCl_2_ and mononuclear complexes with perchlorate anions on the outer sphere in the case of Cu(ClO_4_)_2_) that may be related to different coordinating abilities of chloride and perchlorate anions.

It was shown that full or partial Cu^2+^ → Cu^1+^ reduction can occur during the complexation reactions, wherein the reducing agent can be either an organic solvent or a selenium-containing ligand. This is a fundamental difference between selenium-containing bis-5-(2-pyridylmethylene)-3,5-dihydro-4*H*-imidazol-4-ones and their previously described sulfur analogs, which are unable themselves to reduce copper during the formation of coordination compounds, and reactions with which led to the formation of products of Cu^2+^ → Cu^1+^ reduction only in reducing solvents (alcohols, DMF).

In reducing solvents under the same conditions, the reactions of CuCl_2_·2H_2_O with bis-5-(2-pyridylmethylene)-2-seleno-3,5-dihydro-4*H*-imidazol-4-ones in some cases occur with deeper reduction of Cu^2+^ in comparison with sulfur analogues.

A possible scheme for the Cu^2+^ → Cu^1+^ reduction during the complexation reactions with ligands 4 was proposed, based on the data of MS, real time NMR and electrochemical study using RDE technique.

The preliminary *in vitro* biological studies of the obtained selenium-containing ligands and their copper complexes were carried out, which showed high cytotoxicity of most of the synthesized compounds with a higher selectivity to cancer cell lines in comparison with sulfur analogs. Moreover, unlike corresponding sulfur derivatives, synthesized selenium containing ligands themselves demonstrate a high cytotoxicity, comparable in some cases to the toxicity of copper-containing complexes.

## Author contributions

Conceptualization, A. V. F., E. K. B., N. V. Z, A. G. M.; methodology, A. V. F., A. I. S., validation, A. V. F., E. K. B., V. K. N., D. A. G., D. A. S., V. A. T.; formal analysis, A. I. S., D. A. G; investigation, A. V. F., A. I. S., D. A. G., A. A. M., V. A. T., D. A. G., D. A. S., A. A. S., A. A. B., V. I. P.; data curation, A. V. F., R. S. B., E. K. B.; writing—original draft preparation, A. V. F., E. K. B.; writing—review and editing, A. V. F., E. K. B., A. G. M., M. Y. M.; visualization, A. V. F., A. I. S., D. A. G., A. A. M., V. A. T., D. A. G., D. A. S., A. A. S., V. I. P.; supervision, E. K. B., A. G. M.; project administration, E. K. B.; funding acquisition, E. K. B. All authors have read and agreed to the published version of the manuscript.

## Conflicts of interest

There are no conflicts to declare.

## Supplementary Material

RA-012-D1RA08995A-s001

RA-012-D1RA08995A-s002
