# Supplementary material for: Copper coordination compounds with (5Z,5Z′)-2,2′-(alkane-α,ω-diyldiselenyl)-bis-5-(2-pyridylmethylene)-3,5-dihydro-4H-imidazol-4-ones. Comparison with sulfur analogue
Source: RSC Adv. 2022 Mar 2;12(12):7133–48. doi: 10.1039/d1ra08995a (PMC8982280; doi:10.1039/d1ra08995a)
Supplement: RA-012-D1RA08995A-s001 [file RA-012-D1RA08995A-s001.pdf]

Electronic Supplementary Material (ESI) for RSC Advances.
This journal is © The Royal Society of Chemistry 2022

Copper coordination compounds of with (5Z,5Z')-2,2'-(alkane-α,ω-diyldiselenyl)-bis-5-(2-pyridylmethylene)-3,5-dihydro-4H-imidazol-4-ones. Comparison with sulfur analogues

Alexander V. Finko, [a,b] Anatolii I. Sokolov, [a] Dmitry A. Guk, [a] Victor A. Tafeenko, [a] Anna A. Moiseeva, [a] Dmitry A. Skvortsov, [a,c] Andrei A. Stomakhin, [d] Andrei A. Beloglazkin, [a,b] Roman S. Borisov, [b] Vladimir I.Pergushov, [a] Mikhail Ya. Melnikov, [a] Nikolay V. Zyk, [a] Alexander G. Majouga, [a,e,f] and Elena K. Beloglazkina [a,]*

a. *Moscow State University, Department of Chemistry, Leninskie Gory, Building 1/3, Moscow 119991, Russia.*
b. *Topchiev Institute of Petrochemical Synthesis RAS, Leninskii pr., 29, Moscow 119991, Russia*
c. *Higher School of Economics, Myasnitskaya 13, Moscow 101000, Russia.*
d. *Engelhardt Institute of Molecular Biology RAS, Vavilova 32, Moscow 119991, Russia.*
e. *National University of Science and Technology, Leninskii pr., 4, Moscow 119049, Russia.*
f. *Mendeleev University of Chemical Technology, Miusskaya pl. 9, Moscow 125047, Russia.*

Supplementary Information

Table of Content

NMR (^{1}H and ^{13}C) and FTIR spectra	Page 2
FTIR spectra of complex compounds	Page 44
Voltammograms (Figures 1S - 22S).	Page 56
Table S1. Electrochemical reduction and oxidation potentials of ligands and complexes	Page 78
Figure 23S. ^{1}H NMR monitoring of the reaction mixture of ligand **4c** with $CuCl_2 \cdot 2H_2O$ in DMF and DMSO.	Page 79
Figure 24 S. MALDI spectra of complex **5c** on different matrices	Page 80
X-Ray analysis (Tables 2S - 25S)	Page 84
UV-vis spectra (Figures 25S-31S)	Page 110
EPR spectra (Figures 32S-36S)	Page 113

NMR ^{1}H (Z)-3-(4-ethoxyphenyl)-5-(pyridin-2-ylmethylene)-2-selenoxoimidazolidin-4-one (**3a**)

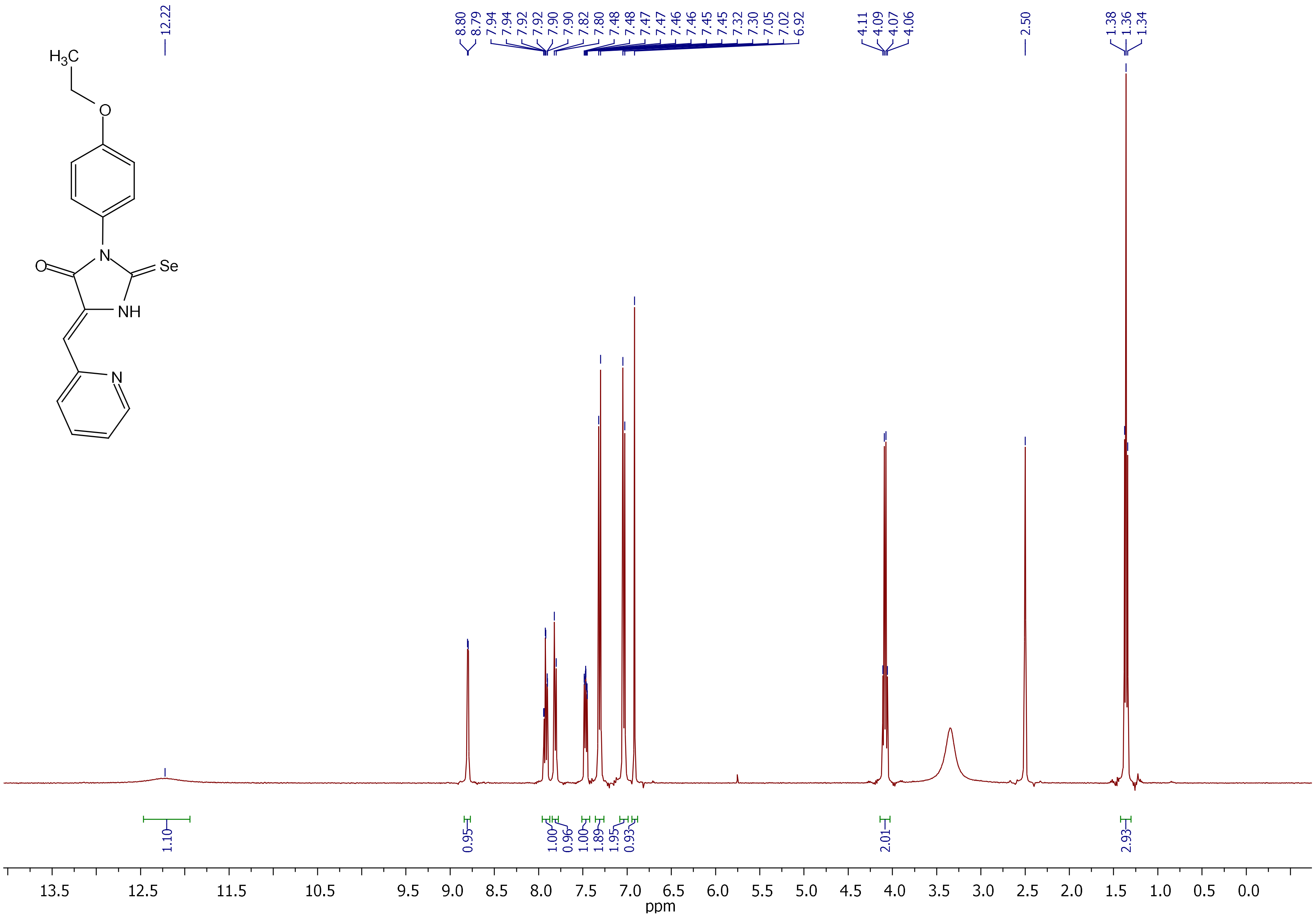

NMR ^{13}C (Z)-3-(4-ethoxyphenyl)-5-(pyridin-2-ylmethylene)-2-selenoxoimidazolidin-4-one (**3a**)

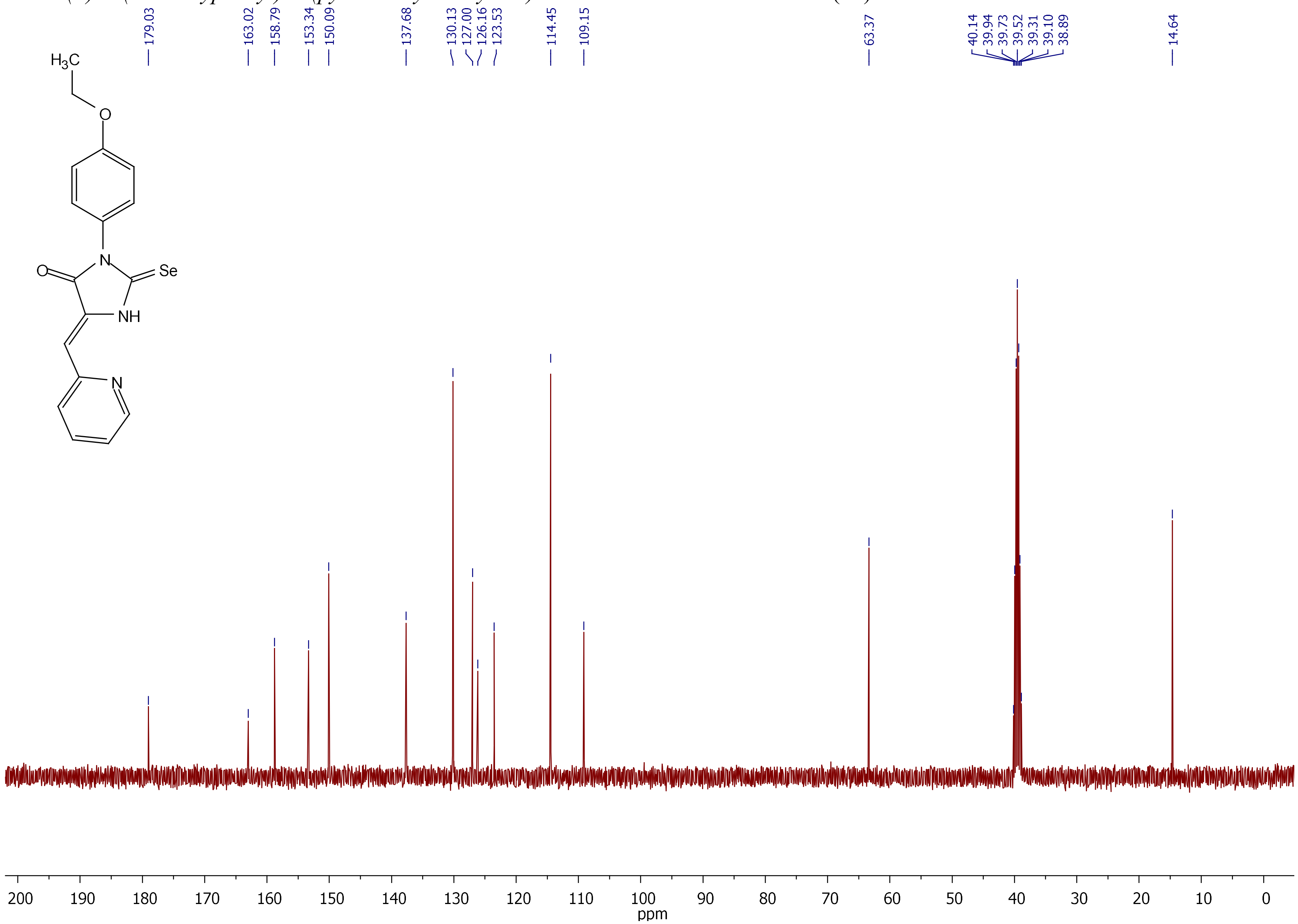

FTIR (Z)-3-(4-ethoxyphenyl)-5-(pyridin-2-ylmethylene)-2-selenoxoimidazolidin-4-one (**3a**)

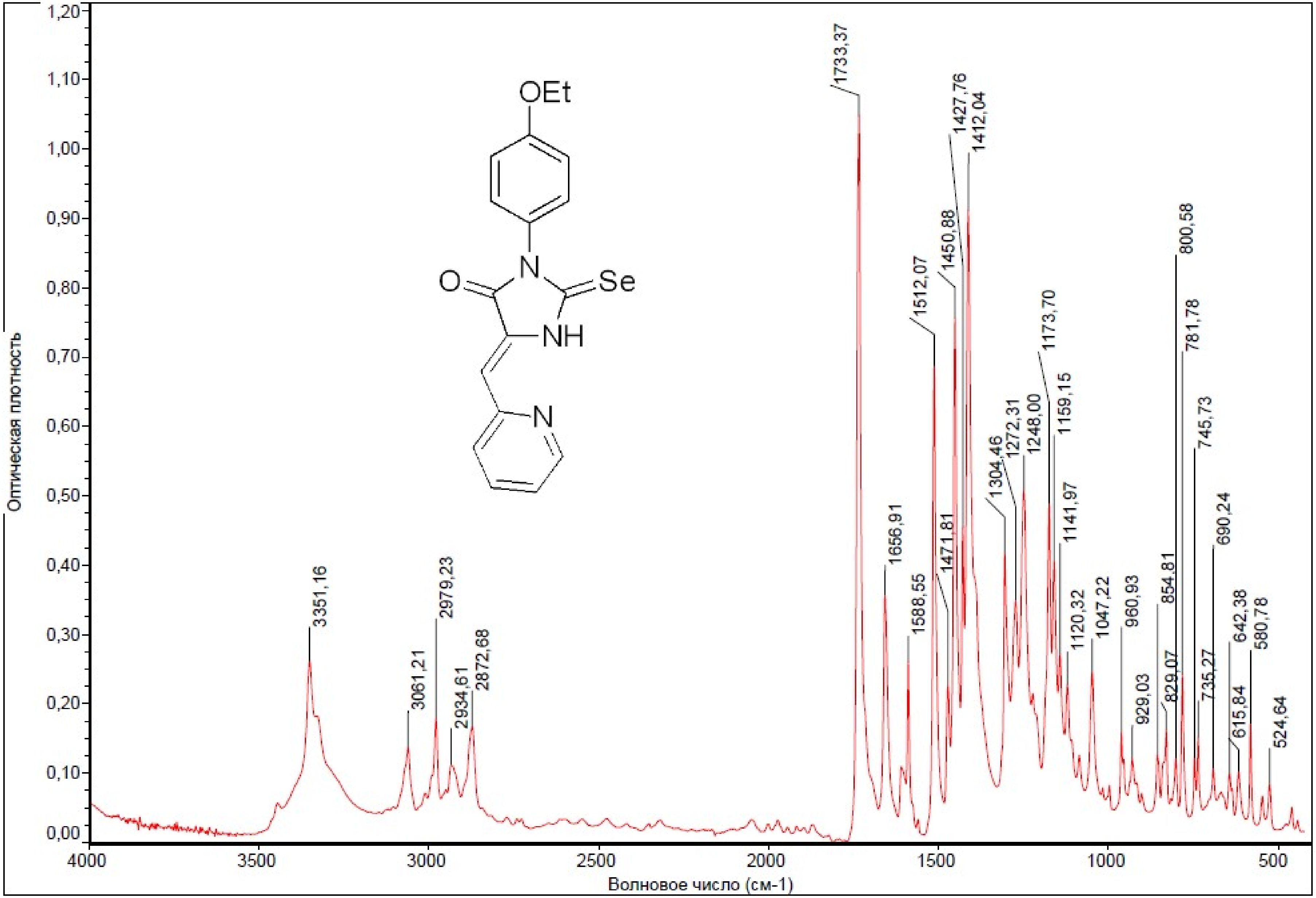

NMR ^{1}H (Z)-4-(5-oxo-4-(pyridin-2-ylmethylene)-2-selenoxoimidazolidin-1-yl)benzonitrile (**3b**).

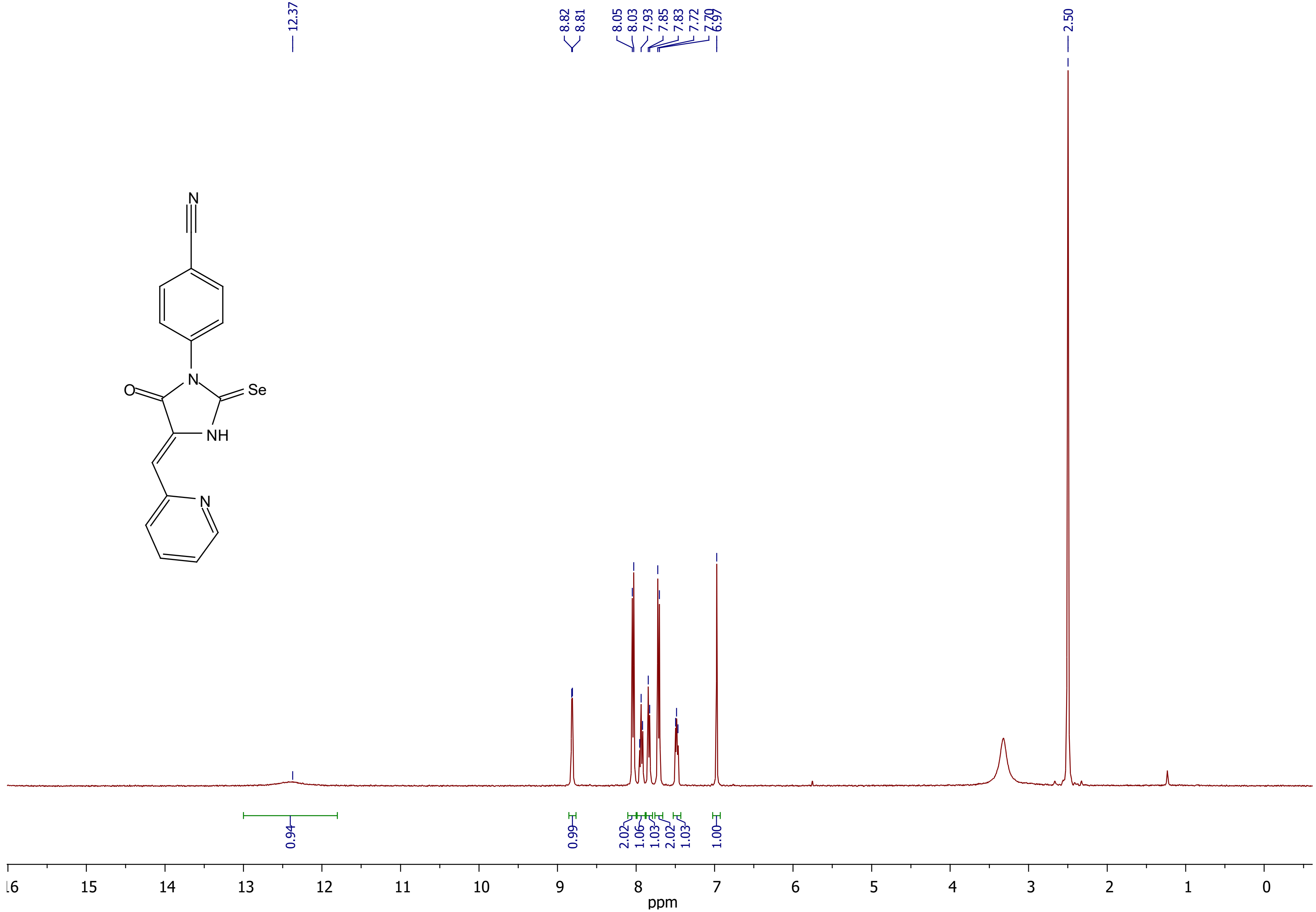

NMR ^{13}C (Z)-4-(5-oxo-4-(pyridin-2-ylmethylene)-2-selenoxoimidazolidin-1-yl)benzonitrile (**3b**).

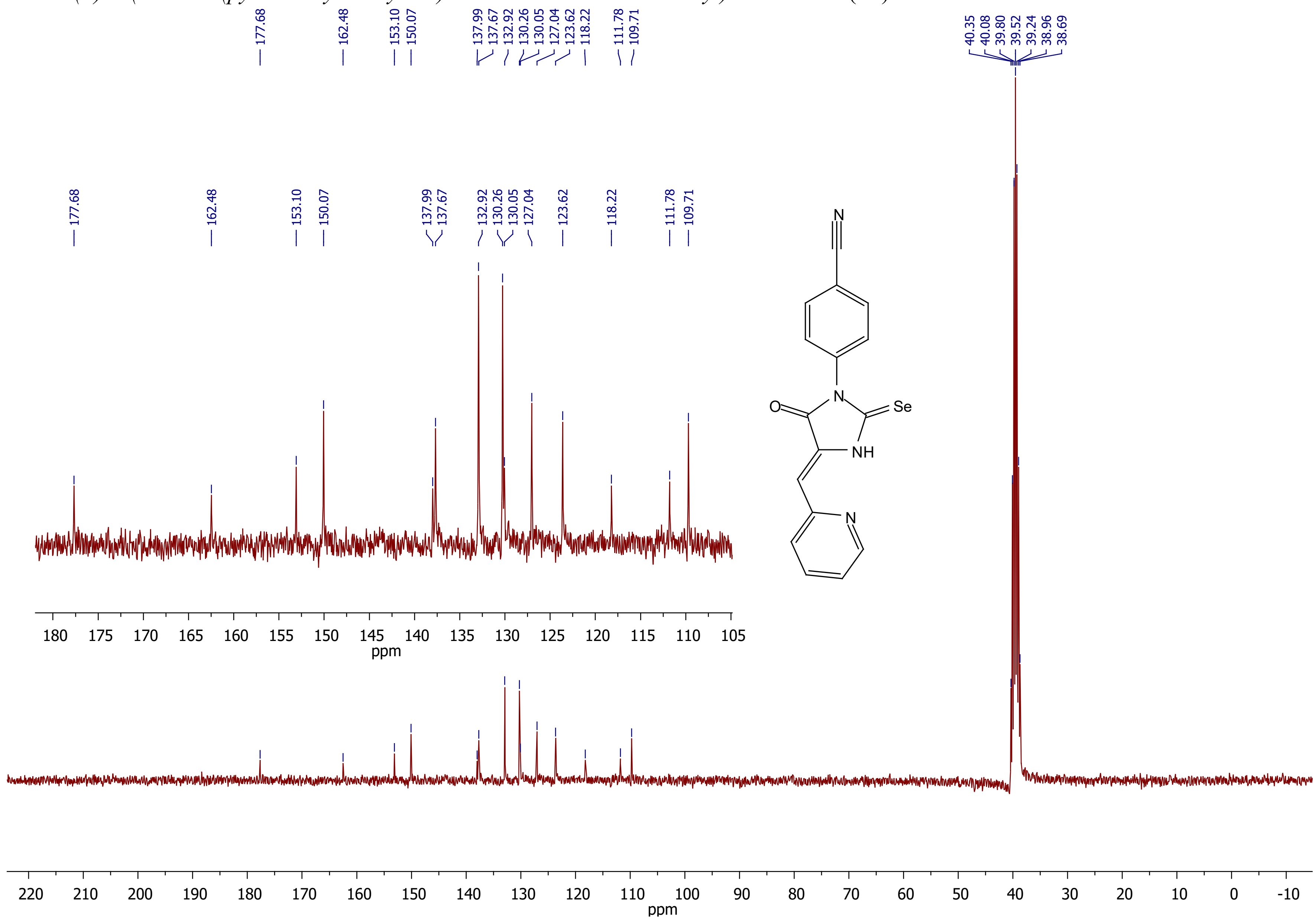

FTIR (Z)-4-(5-oxo-4-(pyridin-2-ylmethylene)-2-selenoxoimidazolidin-1-yl)benzonitrile (**3b**).

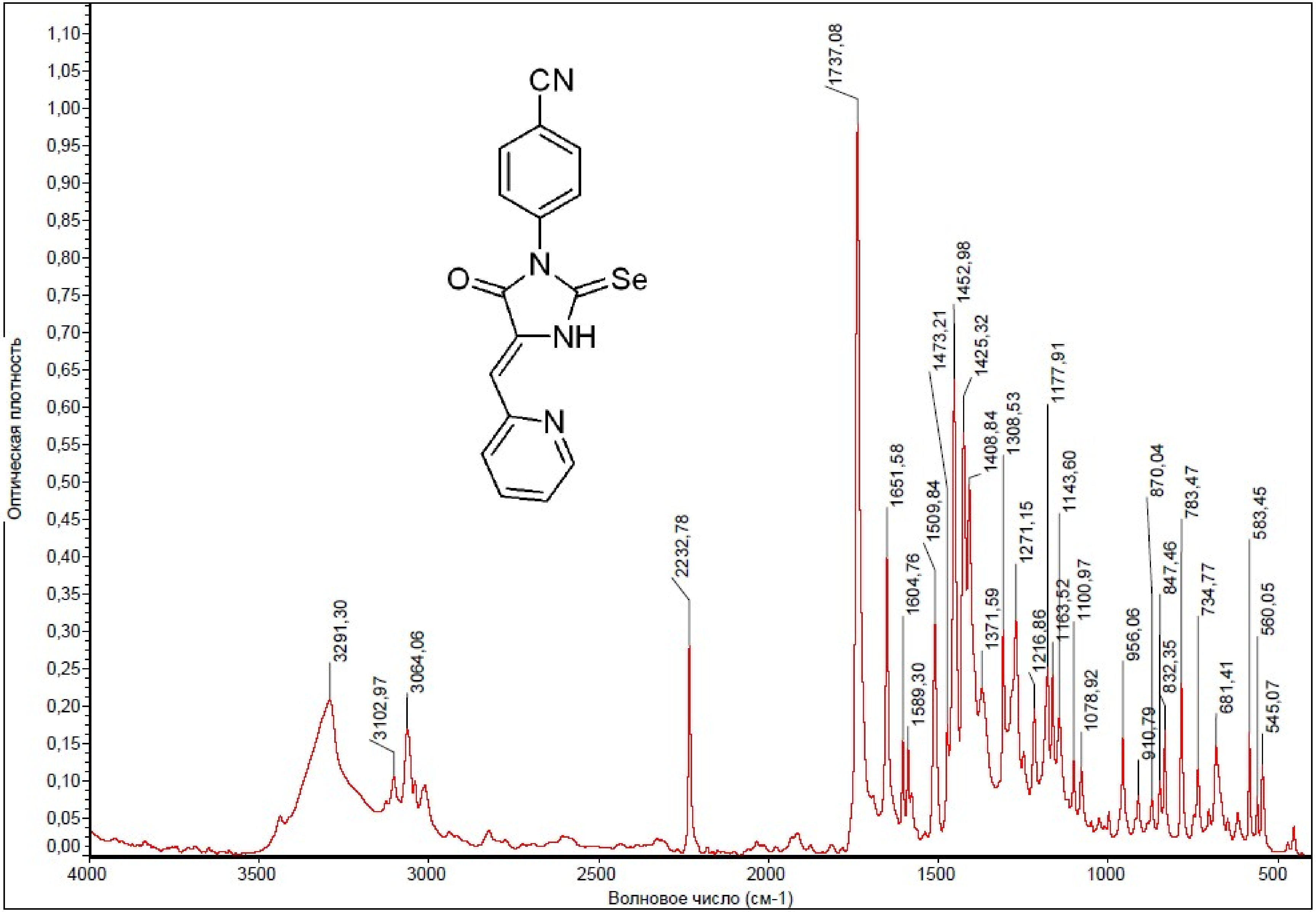

NMR ^{1}H (Z)-3-(4-chlorophenyl)-5-(pyridin-2-ylmethylene)-2-selenoxoimidazolidin-4-one (**3c**).

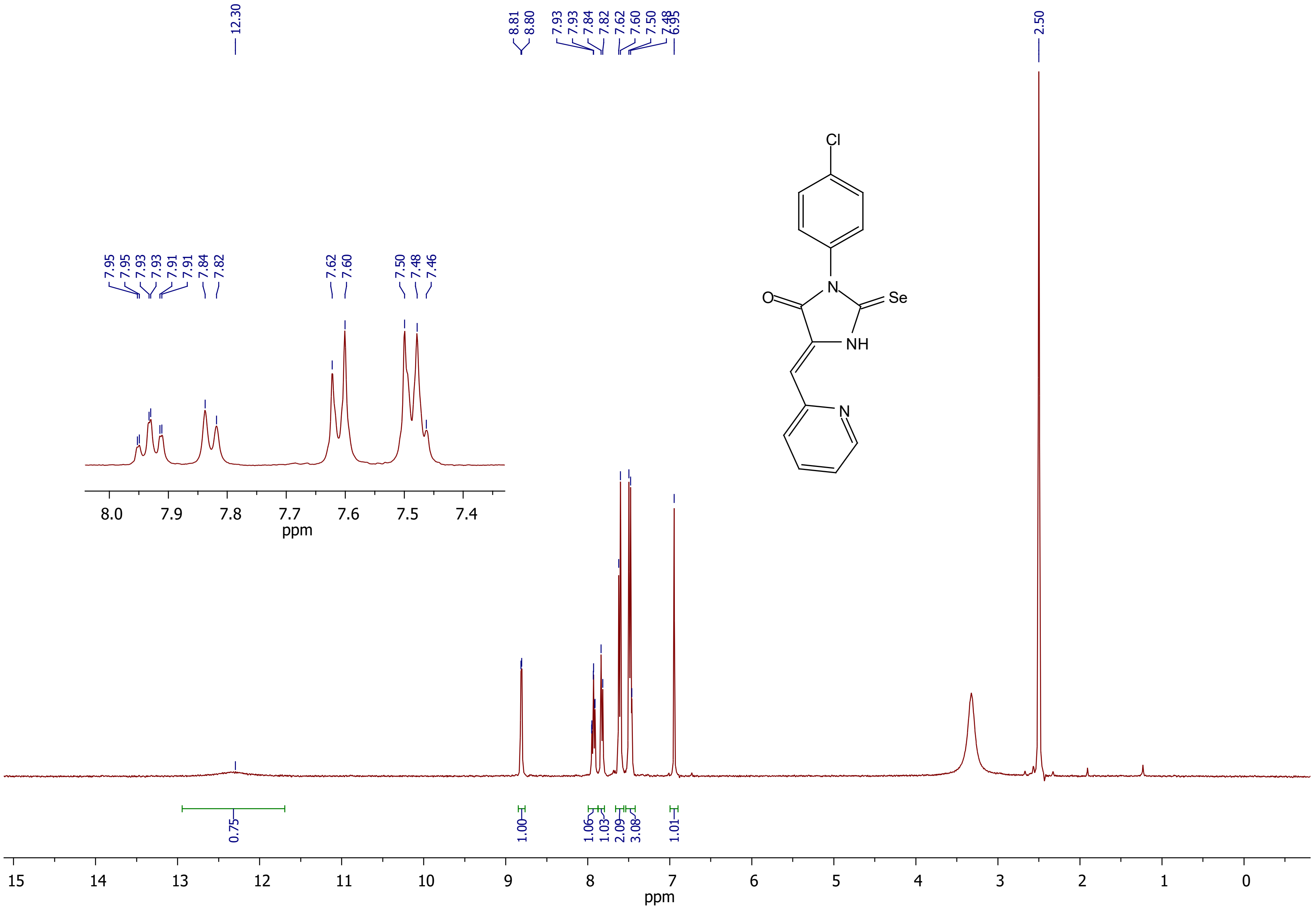

NMR ^{13}C (Z)-3-(4-chlorophenyl)-5-(pyridin-2-ylmethylene)-2-selenoxoimidazolidin-4-one (**3e**).

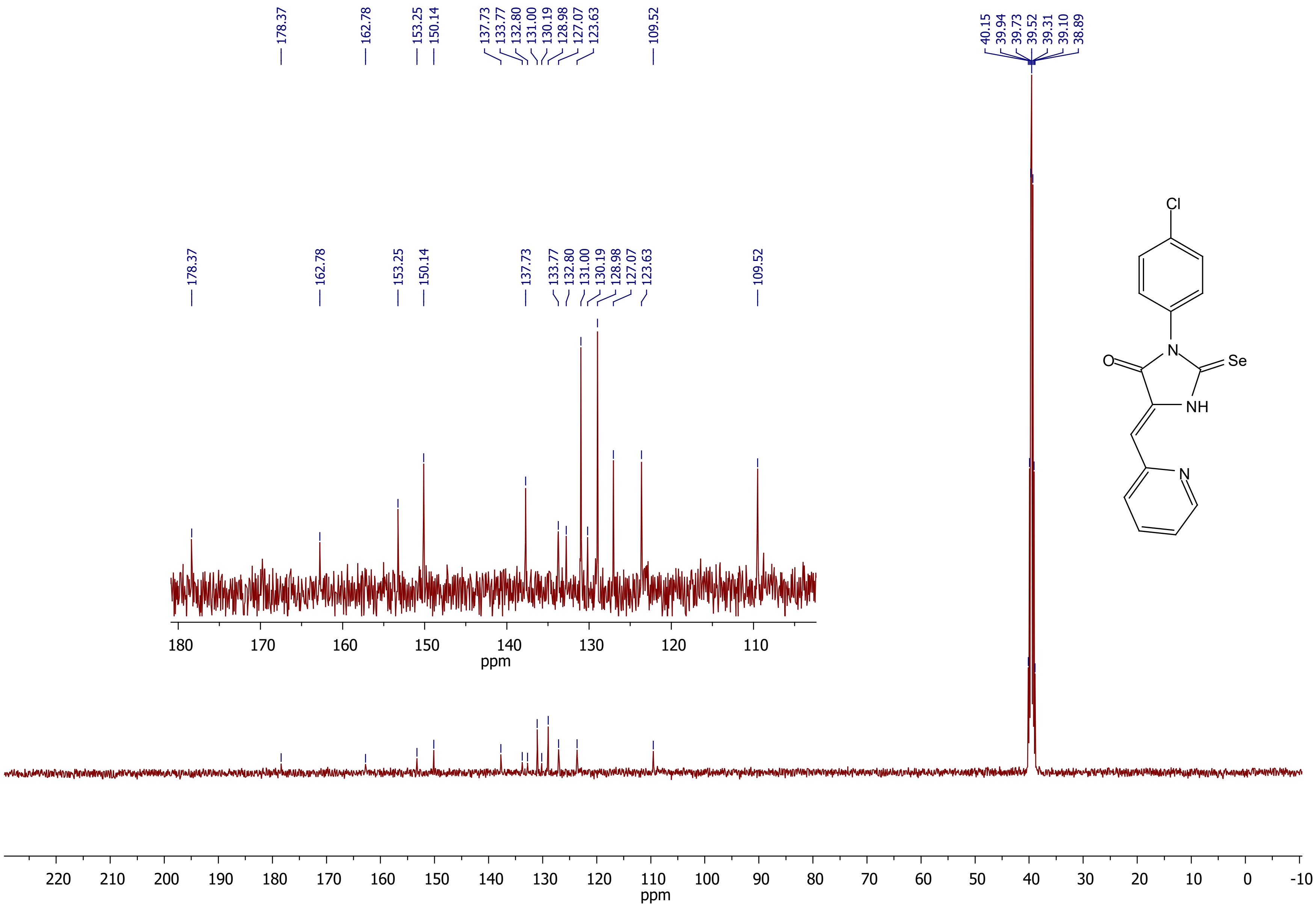

FTIR (Z)-3-(4-chlorophenyl)-5-(pyridin-2-ylmethylene)-2-selenoxoimidazolidin-4-one (**3c**).

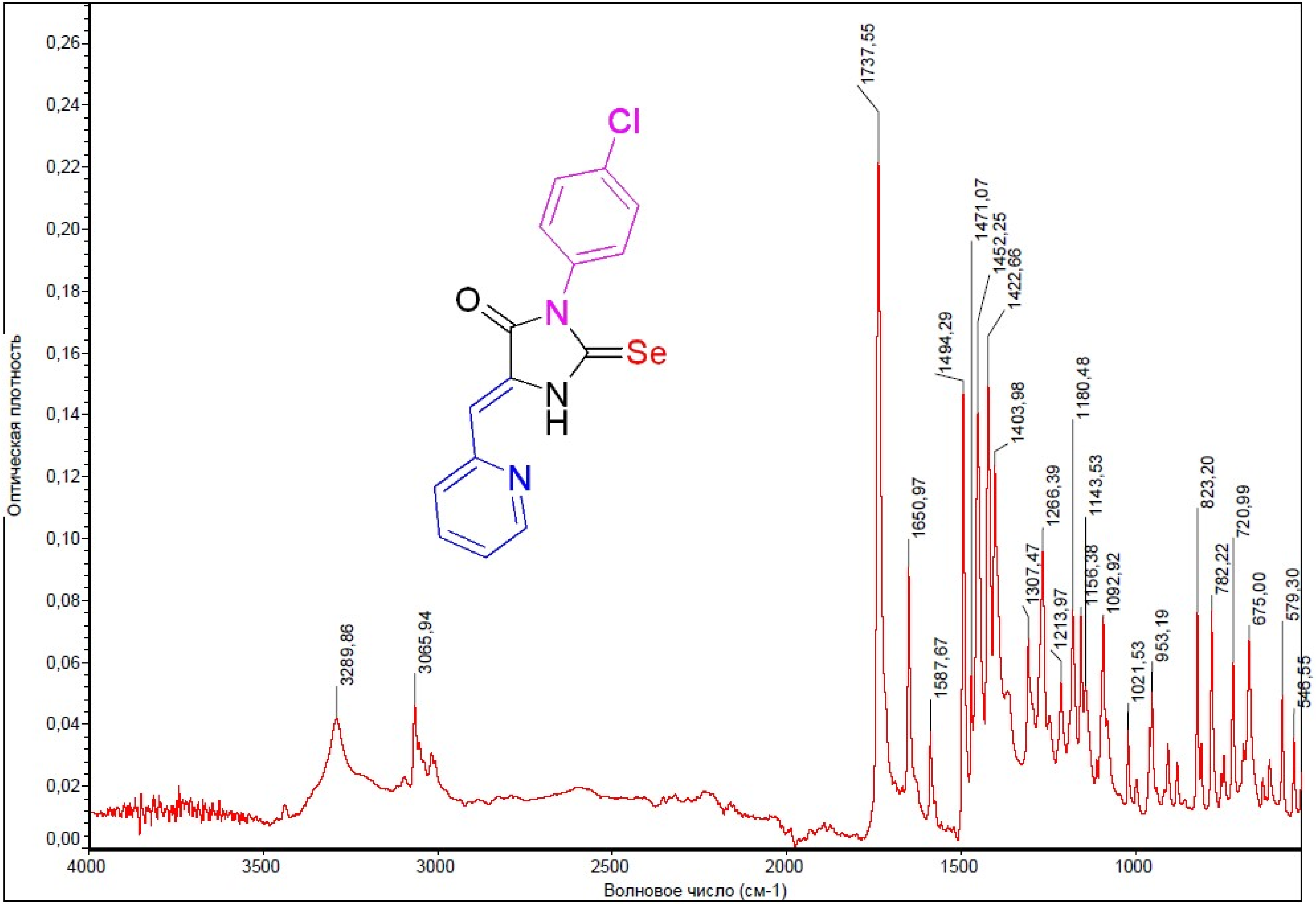

NMR ^{1}H (4Z,4'Z)-2,2'-(ethane-1,2-diylbis(selanediyl))bis(1-(4-ethoxyphenyl)-4-(pyridin-2-ylmethylene)-1H-imidazol-5(4H)-one) (**4a**).

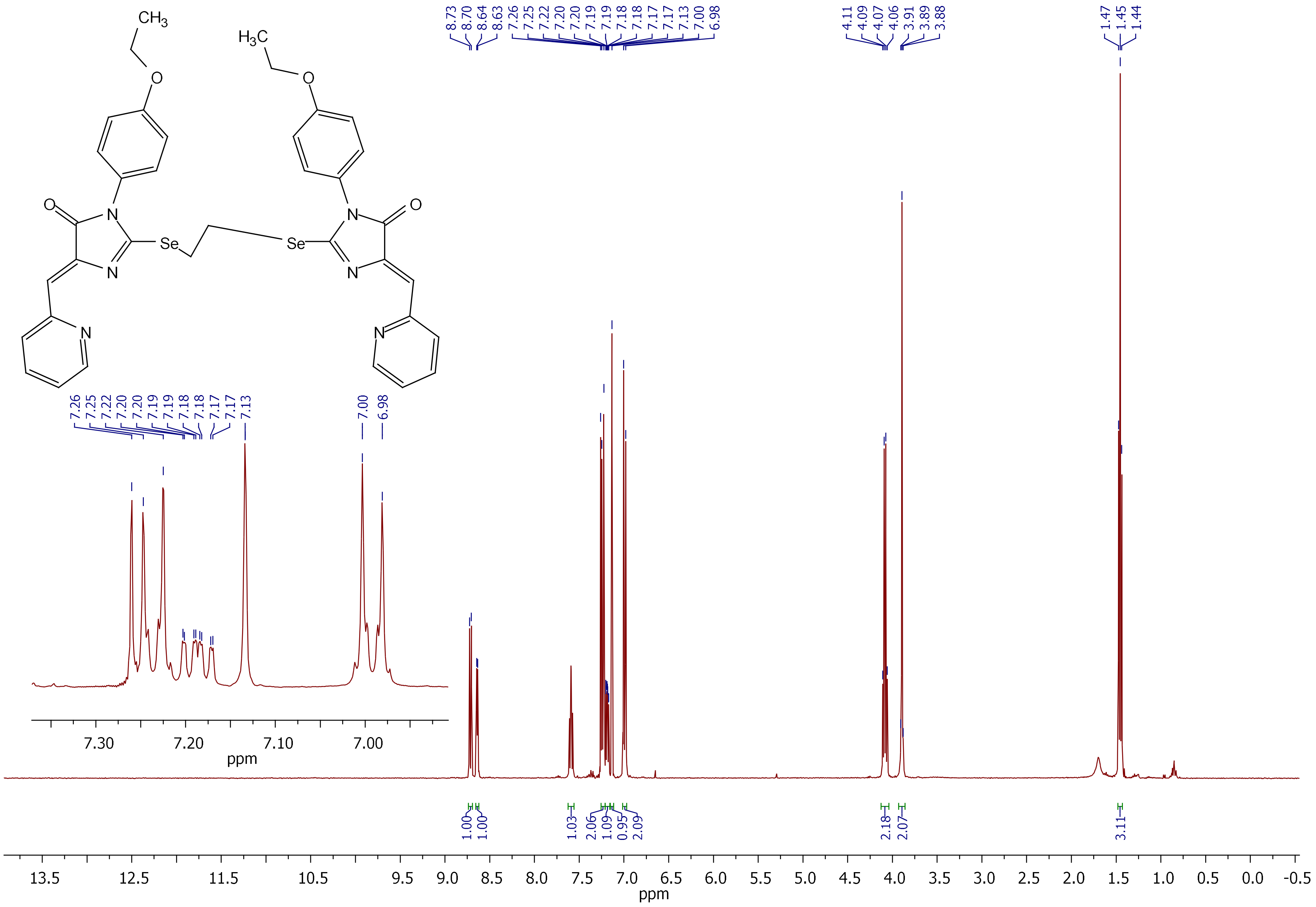

NMR ^{13}C (4Z,4'Z)-2,2'-(ethane-1,2-diylbis(selanediyl))bis(1-(4-ethoxyphenyl)-4-(pyridin-2-ylmethylene)-1H-imidazol-5(4H)-one) (**4a**).

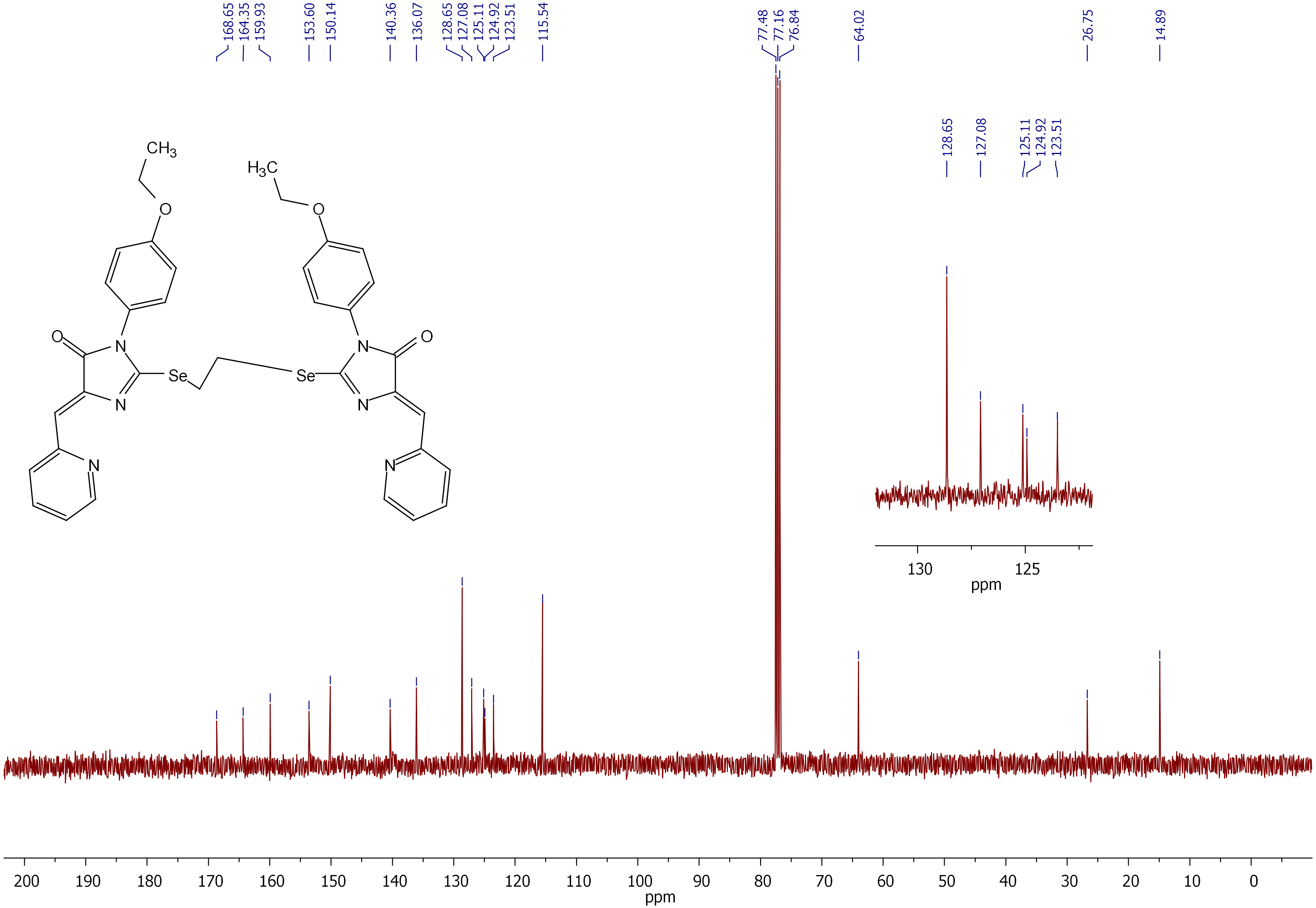

FTIR (4Z,4'Z)-2,2'-(ethane-1,2-diylbis(selanediyl))bis(1-(4-ethoxyphenyl)-4-(pyridin-2-ylmethylene)-1H-imidazol-5(4H)-one) (**4a**).

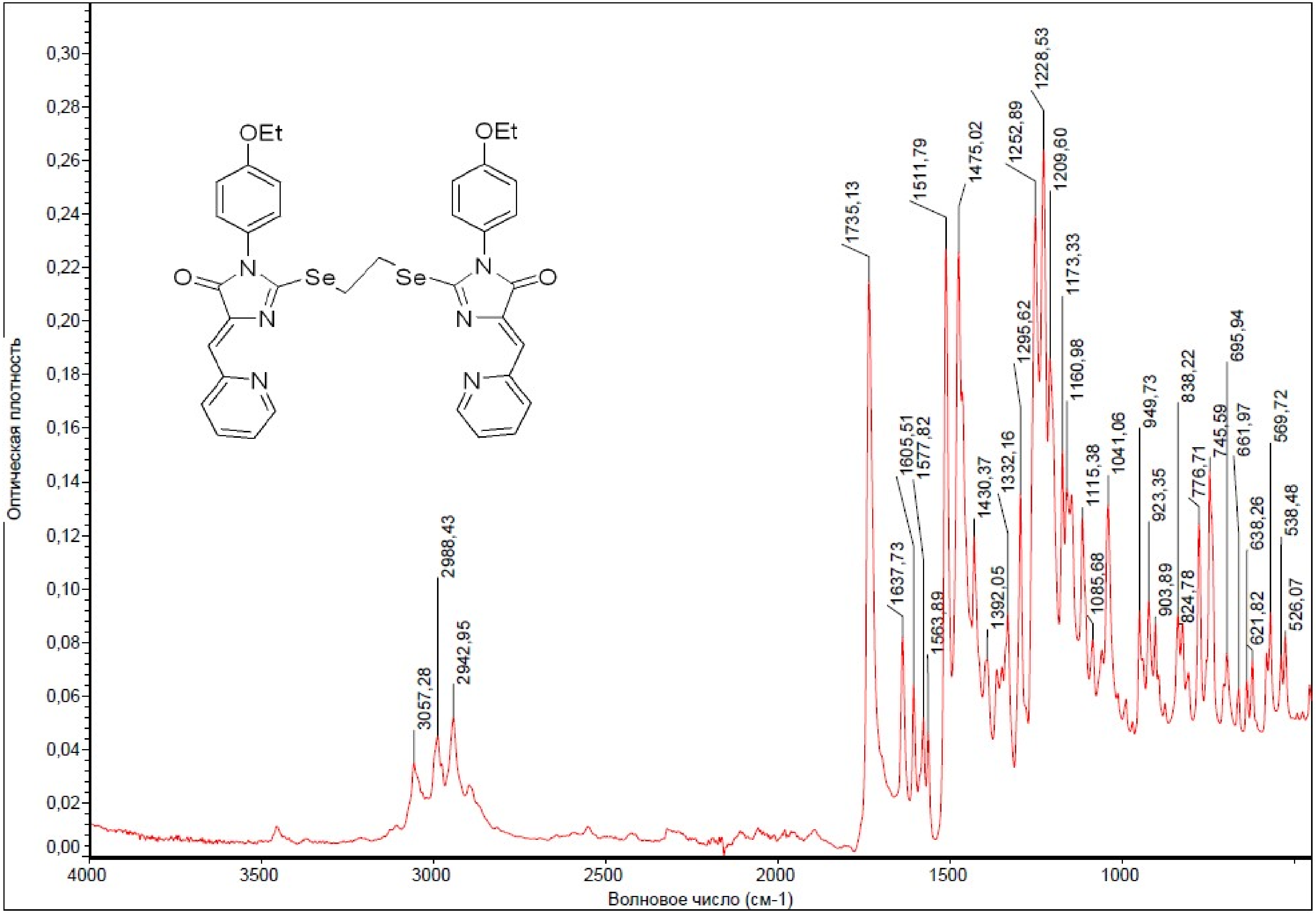

NMR ¹H 4,4'-((4Z,4'Z)-2,2'-(ethane-1,2-diylbis(selanediyl))bis(5-oxo-4-(pyridin-2-ylmethylene)-4,5-dihydro-1H-imidazole-2,1-diyl))dibenzonitrile (**4b**).

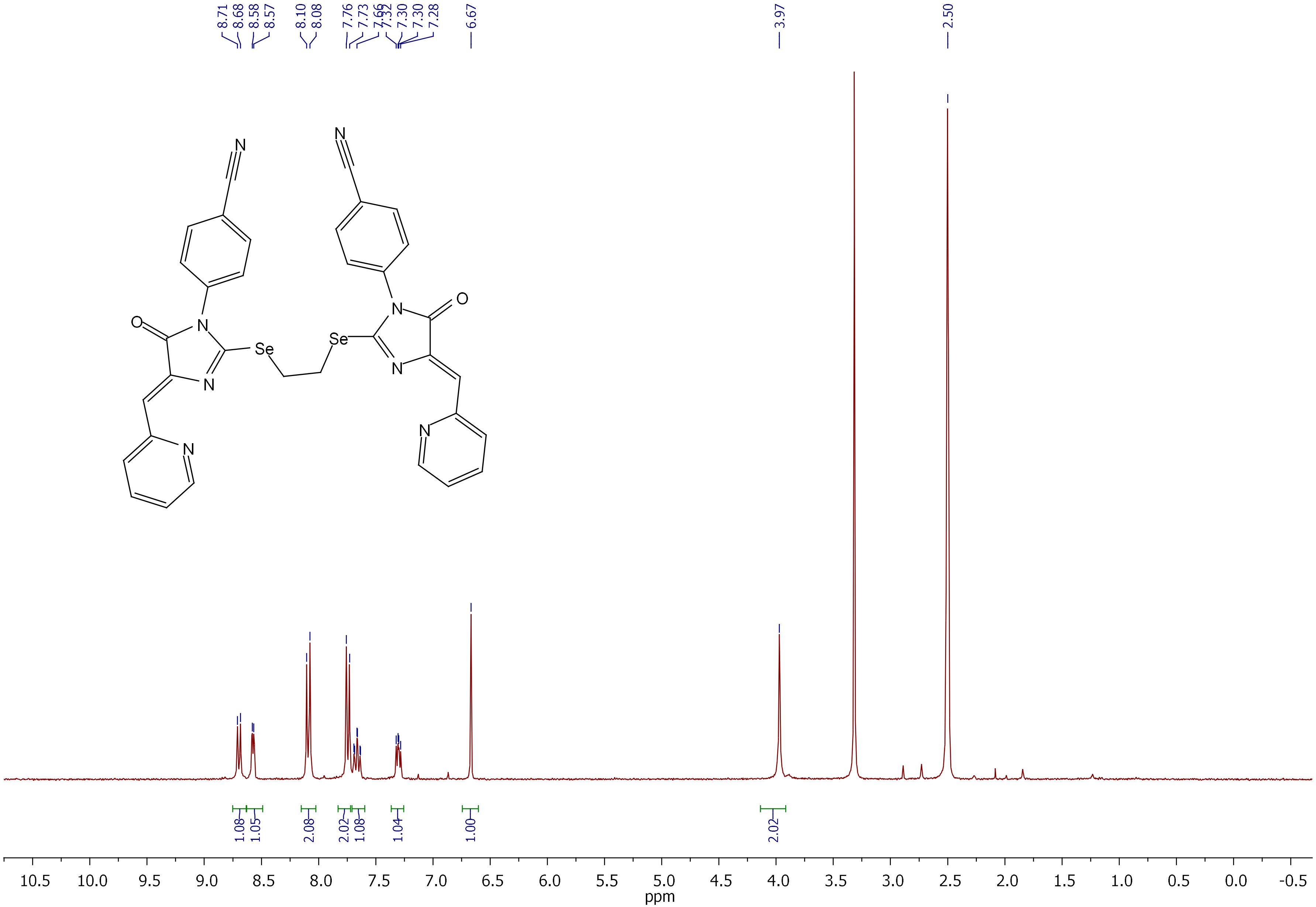

NMR ^{13}C 4,4'-((4Z,4'Z)-2,2'-(ethane-1,2-diylbis(selanediyl))bis(5-oxo-4-(pyridin-2-ylmethylene)-4,5-dihydro-1H-imidazole-2,1-diyl))dibenzonitrile (**4b**).

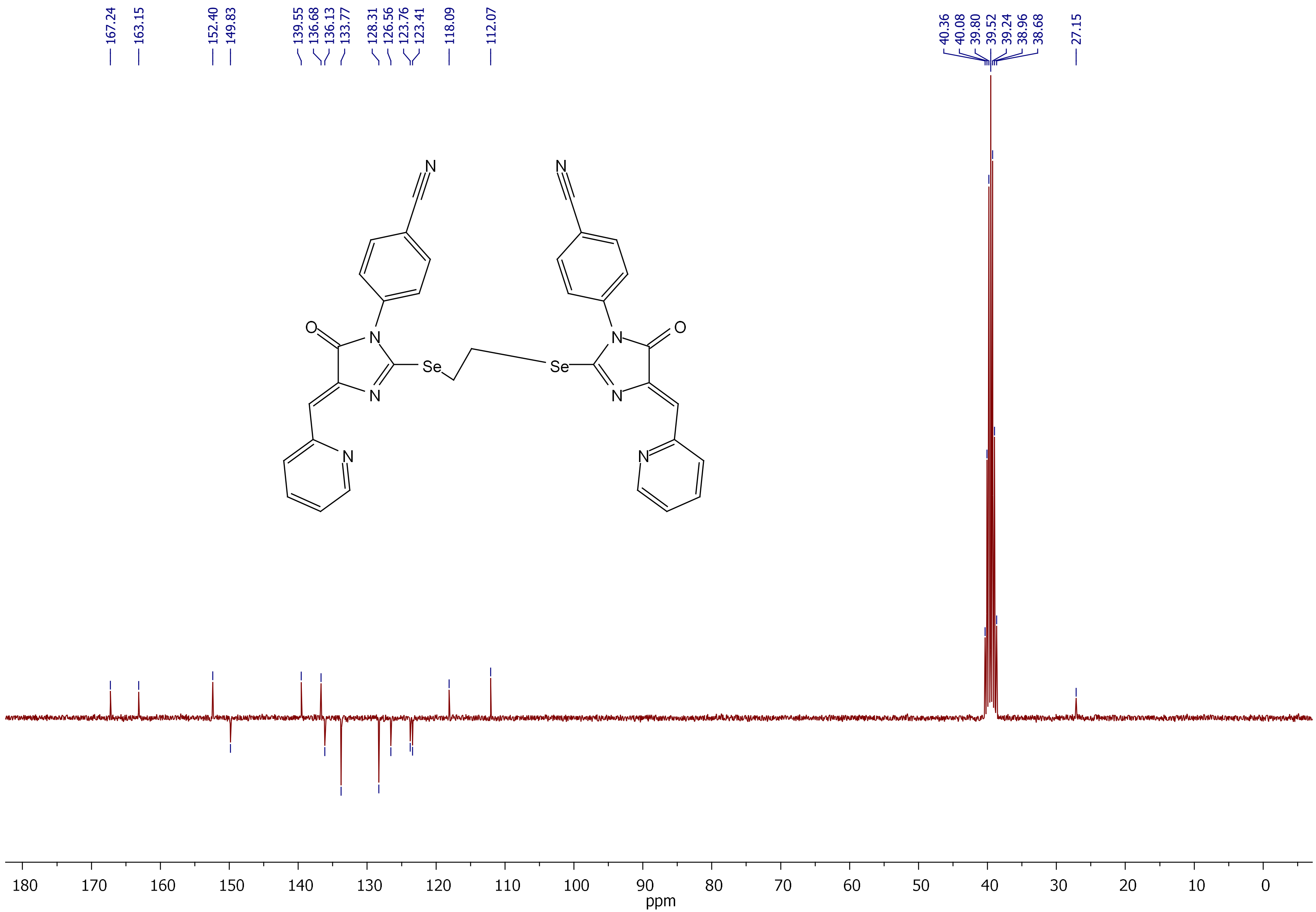

FTIR 4,4'-((4Z,4'Z)-2,2'-(ethane-1,2-diylbis(selanediyl))bis(5-oxo-4-(pyridin-2-ylmethylene)-4,5-dihydro-1H-imidazole-2,1-diyl))dibenzonitrile (**4b**).

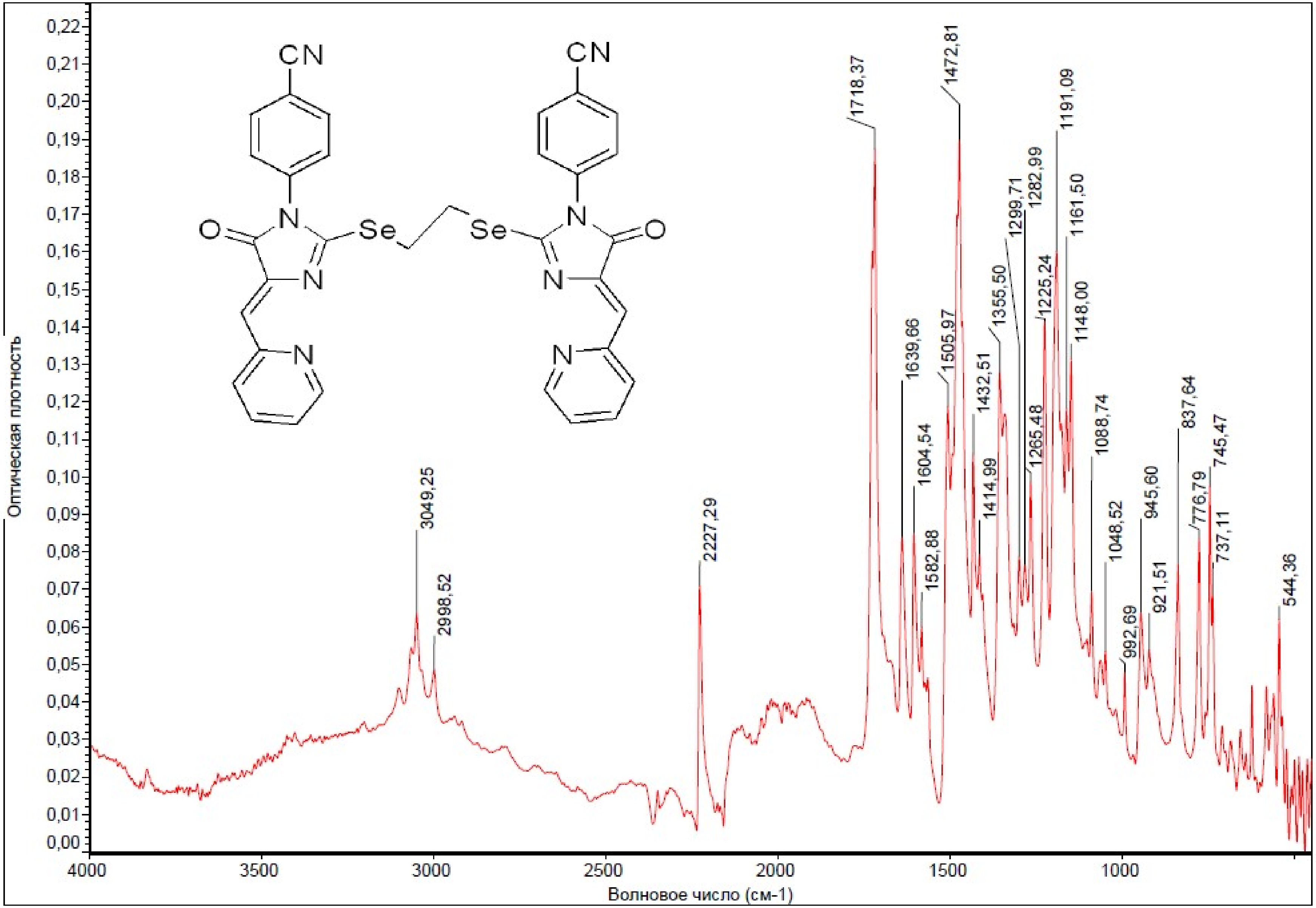

NMR ^{1}H (4Z,4'Z)-2,2'-(ethane-1,2-diylbis(selanediyl))bis(1-(4-chlorophenyl)-4-(pyridin-2-ylmethylene)-1H-imidazol-5(4H)-one) (**4c**).

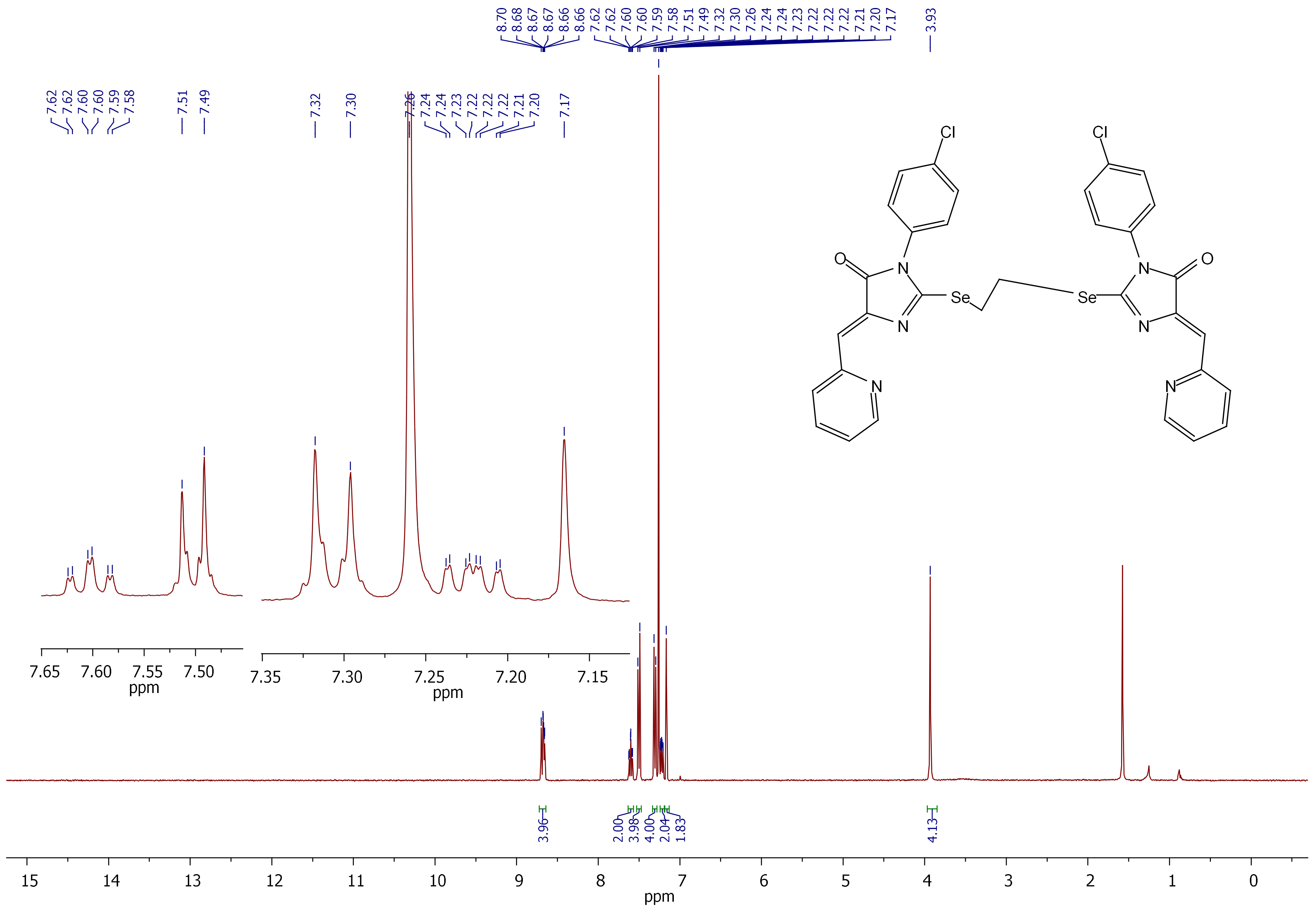

NMR ^{13}C (4Z,4'Z)-2,2'-(ethane-1,2-diylbis(selanediyl))bis(1-(4-chlorophenyl)-4-(pyridin-2-ylmethylene)-1H-imidazol-5(4H)-one) (**4c**).

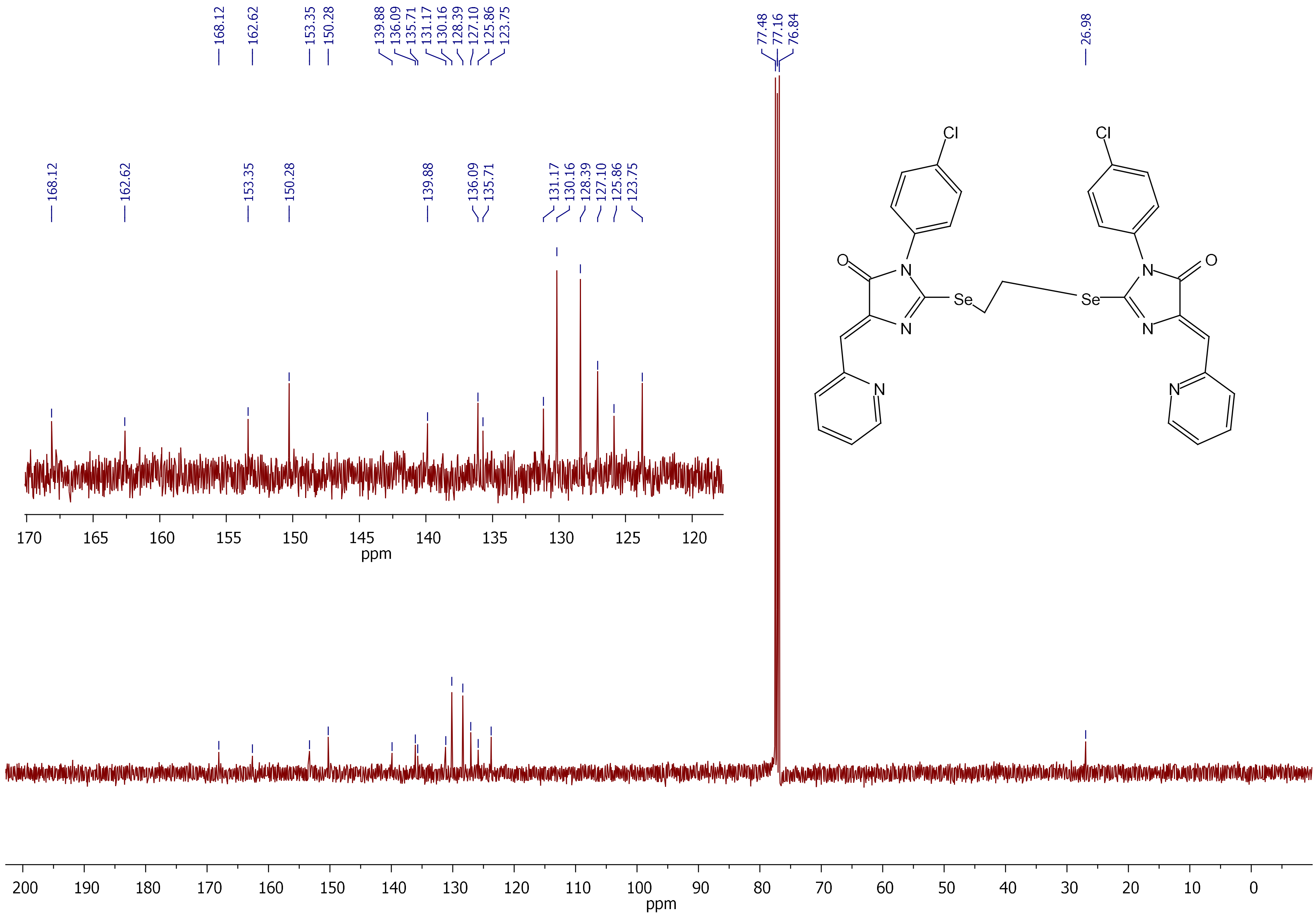

FTIR (4Z,4'Z)-2,2'-(ethane-1,2-diylbis(selanediyl))bis(1-(4-chlorophenyl)-4-(pyridin-2-ylmethylene)-1H-imidazol-5(4H)-one) (**4c**).

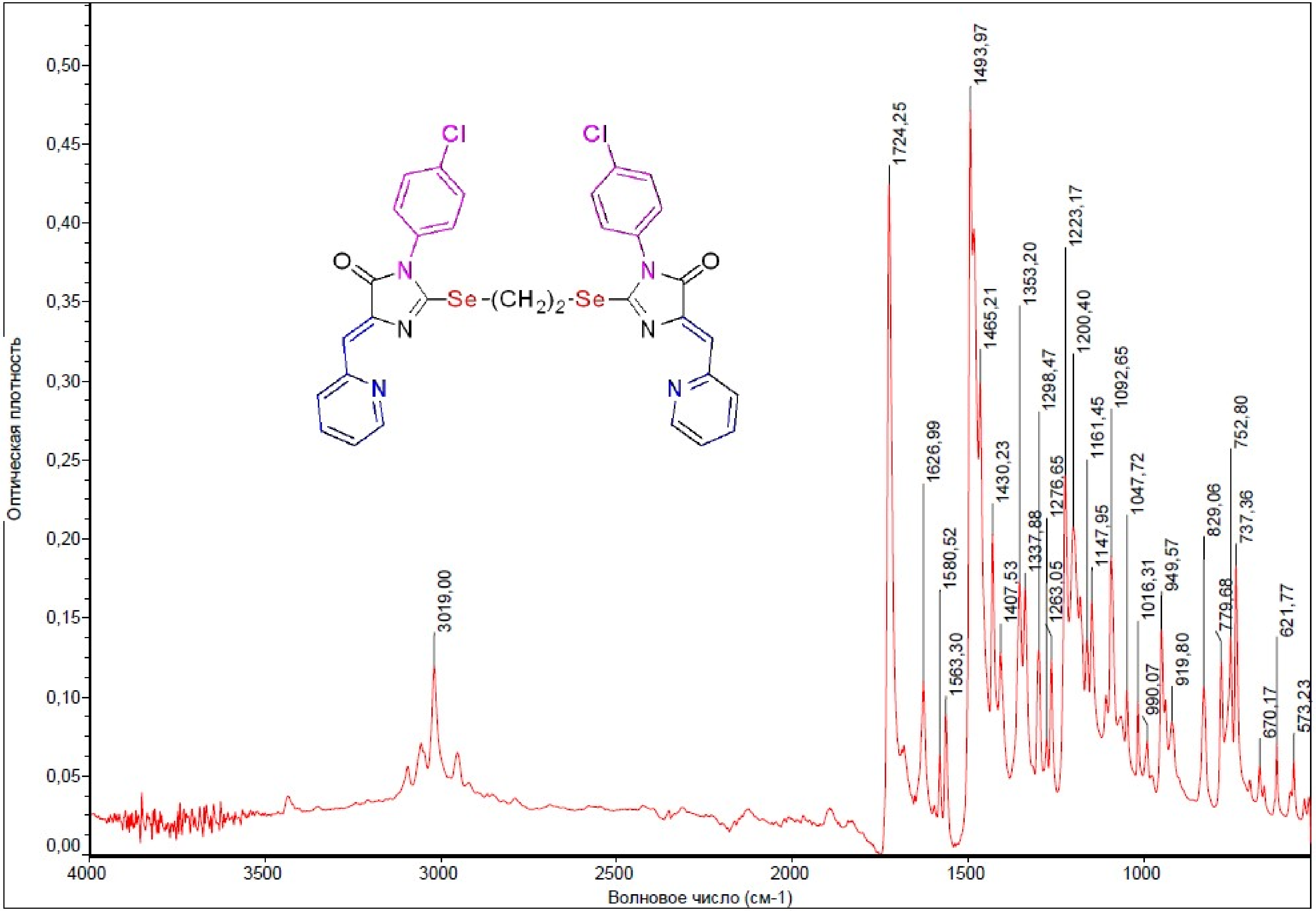

NMR ¹H (4Z,4'Z)-2,2'-(ethane-1,2-diylbis(selanediyl))bis(1-allyl-4-(pyridin-2-ylmethylene)-1H-imidazol-5(4H)-one) (**4d**).

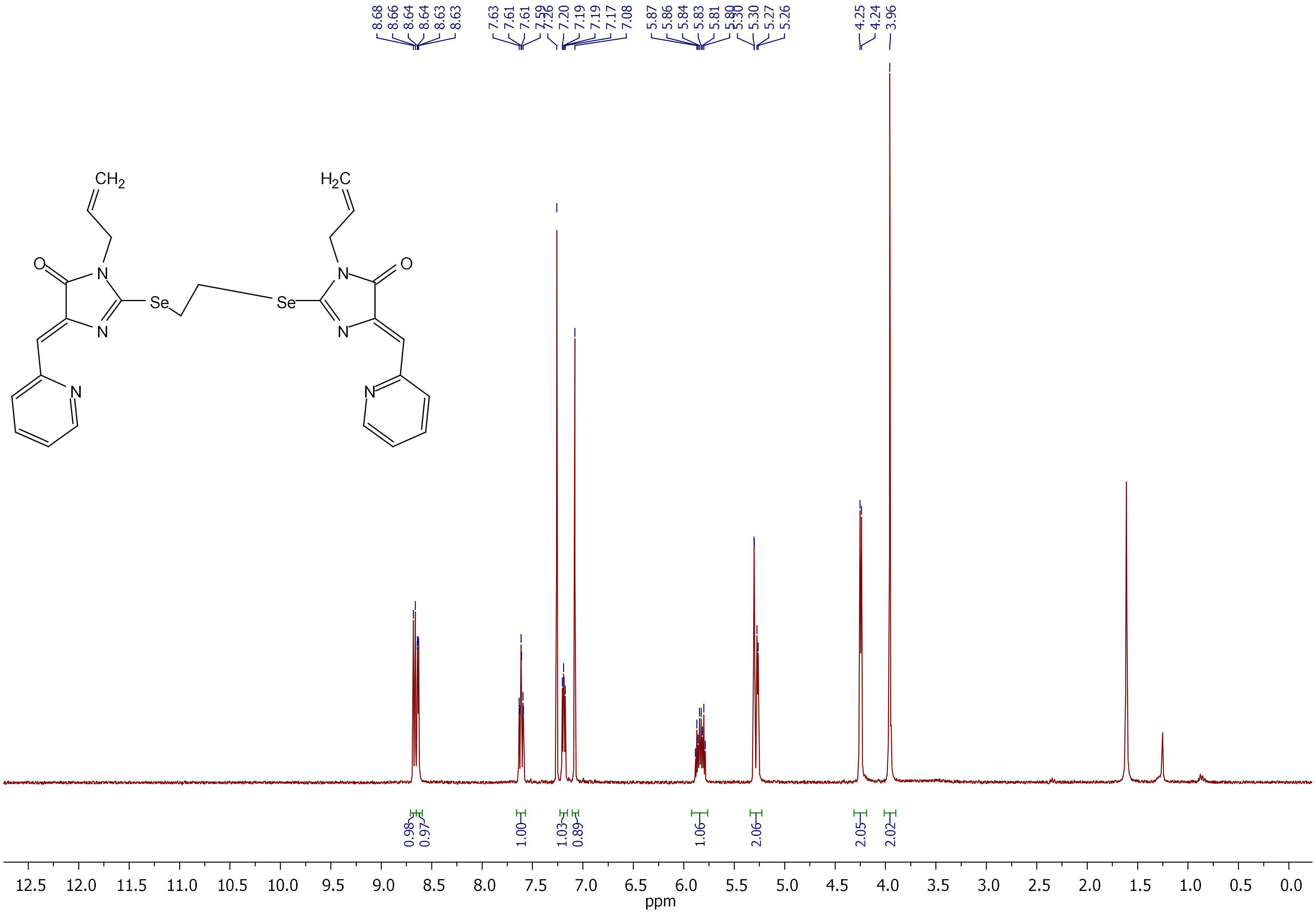

NMR ^{13}C (4Z,4'Z)-2,2'-(ethane-1,2-diylbis(selanediyl))bis(1-allyl-4-(pyridin-2-ylmethylene)-1H-imidazol-5(4H)-one) (**4d**).

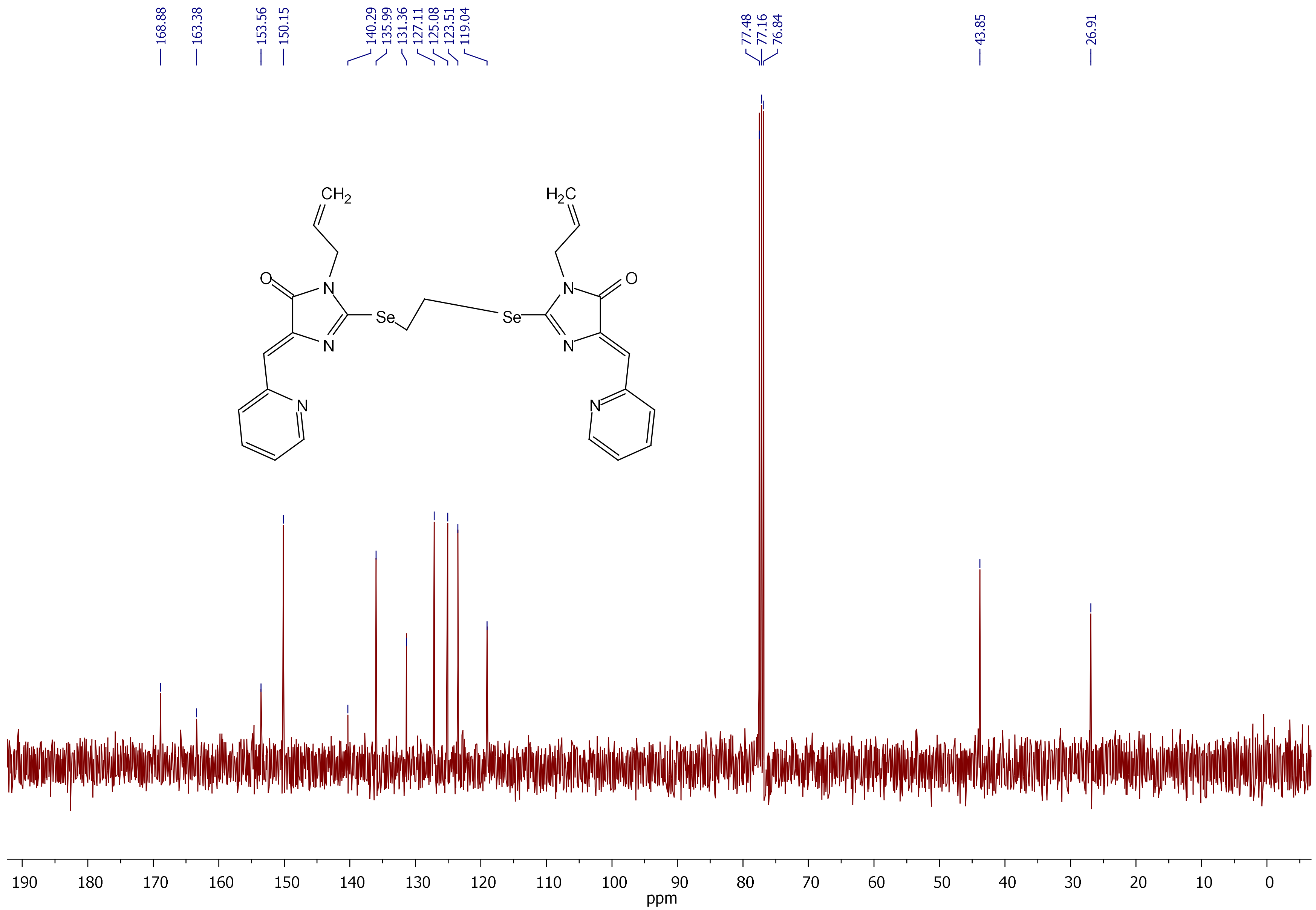

FTIR (4Z,4'Z)-2,2'-(ethane-1,2-diylbis(selanediyl))bis(1-allyl-4-(pyridin-2-ylmethylene)-1H-imidazol-5(4H)-one) (**4d**).

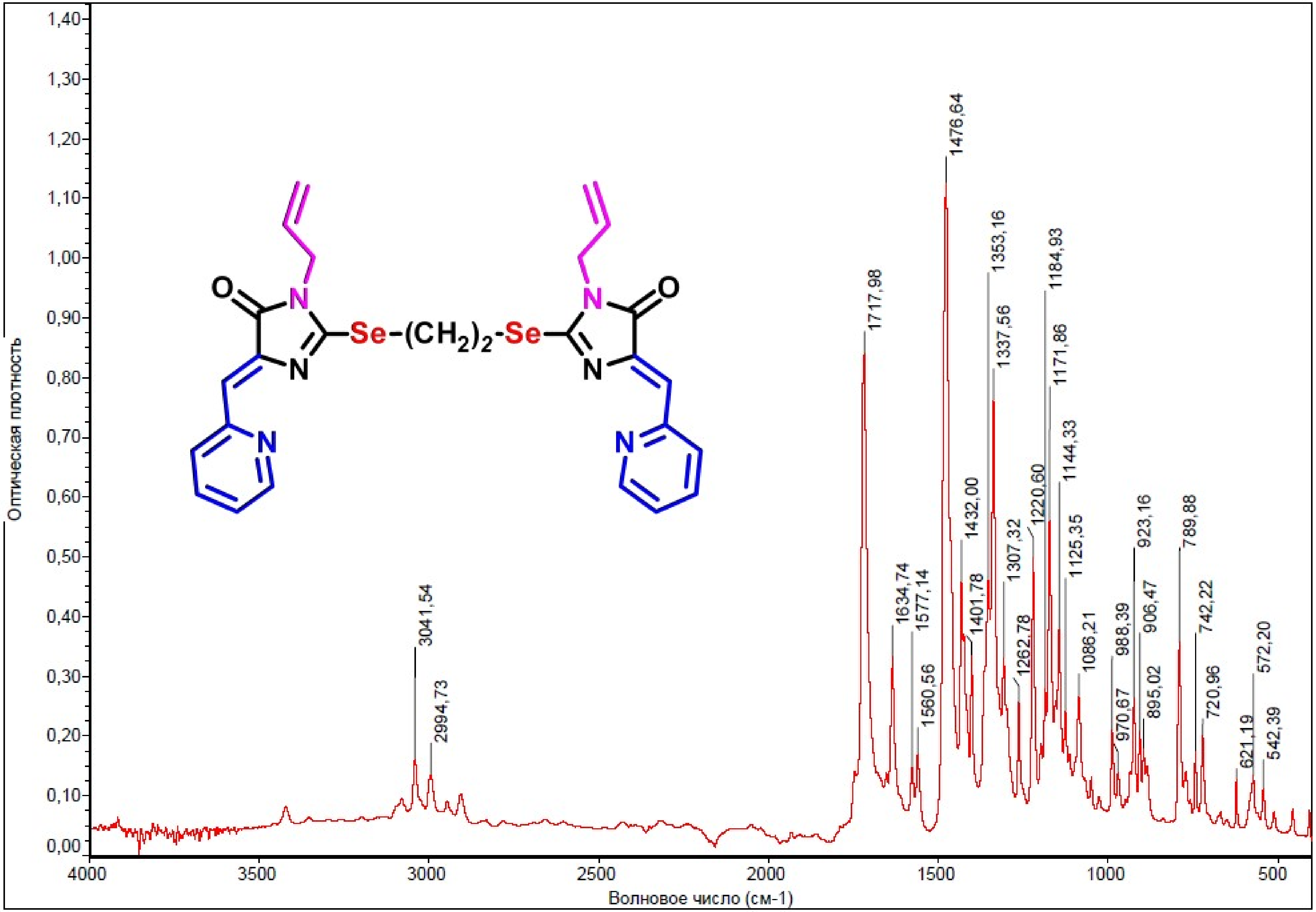

NMR ^{1}H (4Z,4'Z)-2,2'-(ethane-1,2-diylbis(selanediyl))bis(1-cyclopropyl-4-(pyridin-2-ylmethylene)-1H-imidazol-5(4H)-one) (**4e**).

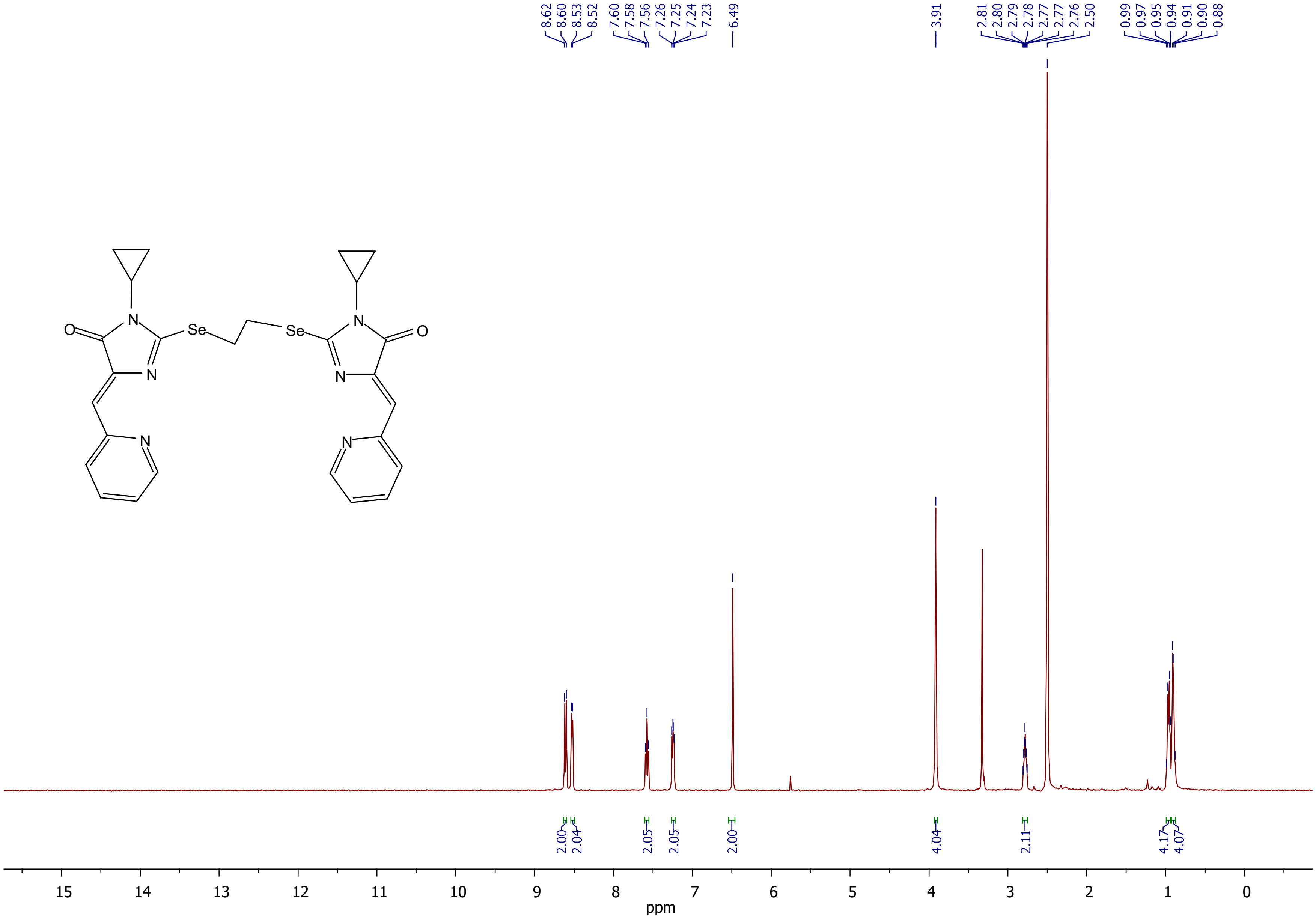

NMR ^{13}C (4Z,4'Z)-2,2'-(ethane-1,2-diylbis(selanediyl))bis(1-cyclopropyl-4-(pyridin-2-ylmethylene)-1H-imidazol-5(4H)-one) (**4e**).

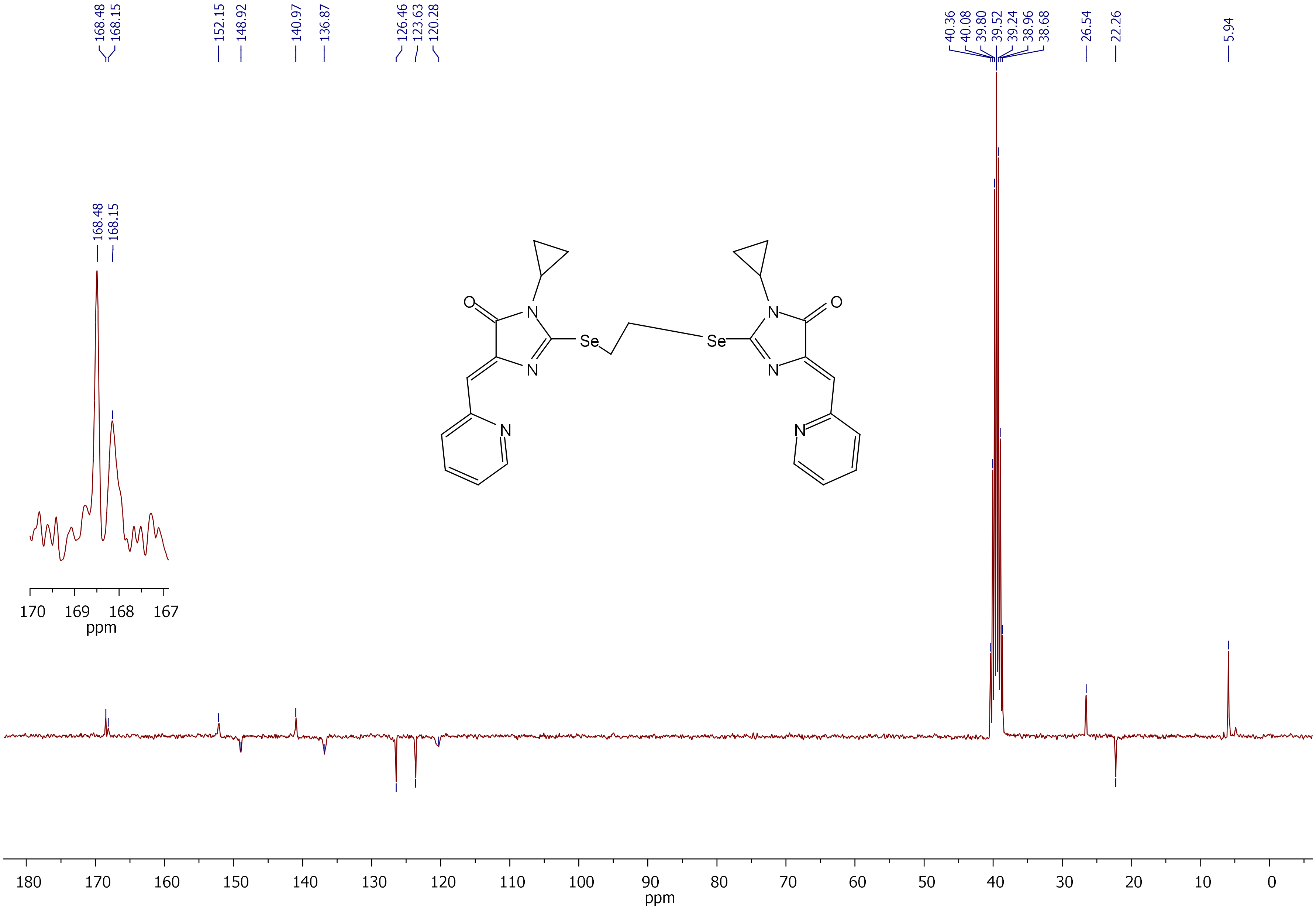

FTIR (4Z,4'Z)-2,2'-(ethane-1,2-diylbis(selanediyl))bis(1-cyclopropyl-4-(pyridin-2-ylmethylene)-1H-imidazol-5(4H)-one) (**4e**).

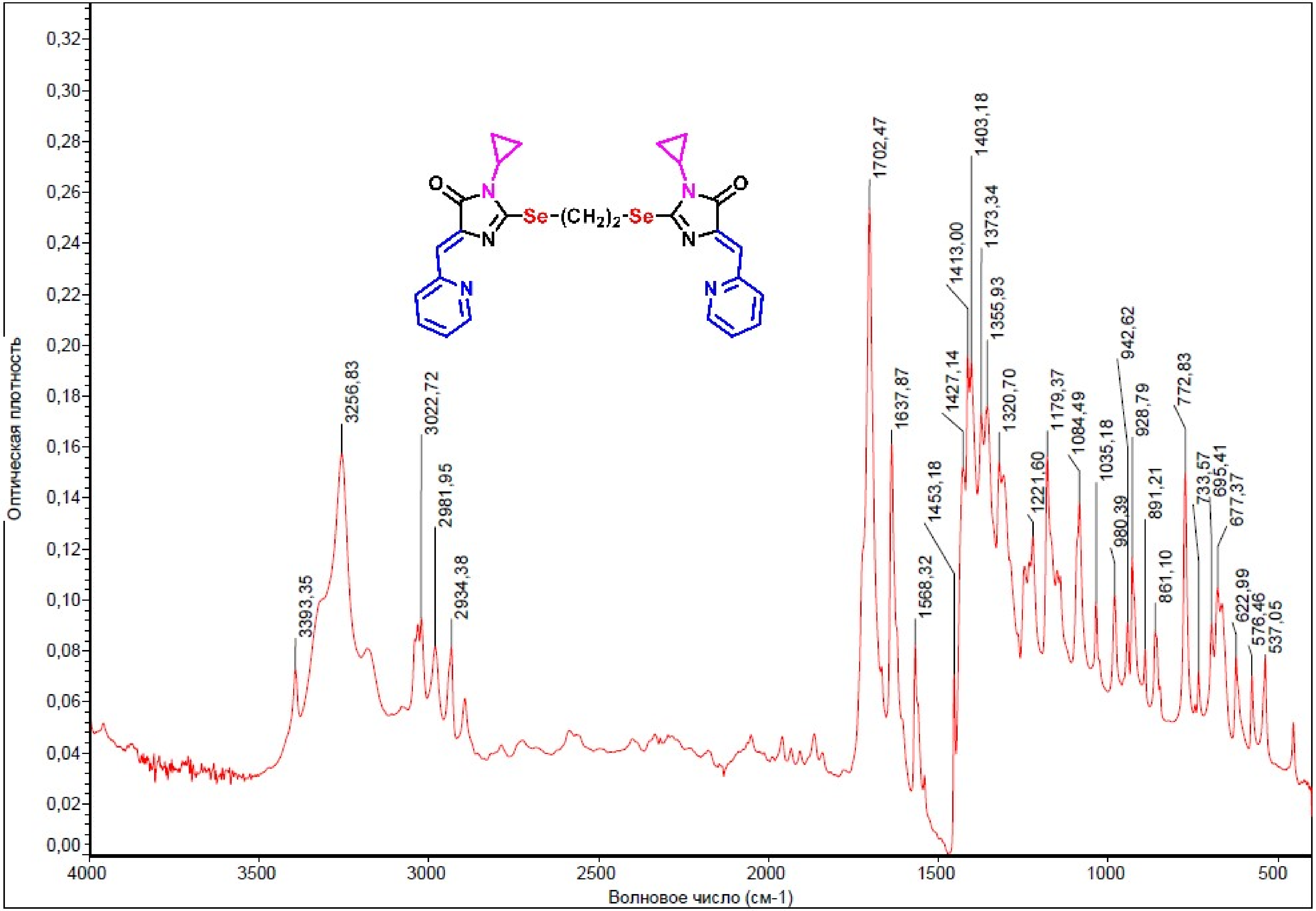

NMR ^{1}H (4Z,4'Z)-2,2'-(butane-1,4-diylbis(selanediyl))bis(1-allyl-4-(pyridin-2-ylmethylene)-1H-imidazol-5(4H)-one) (**4f**).

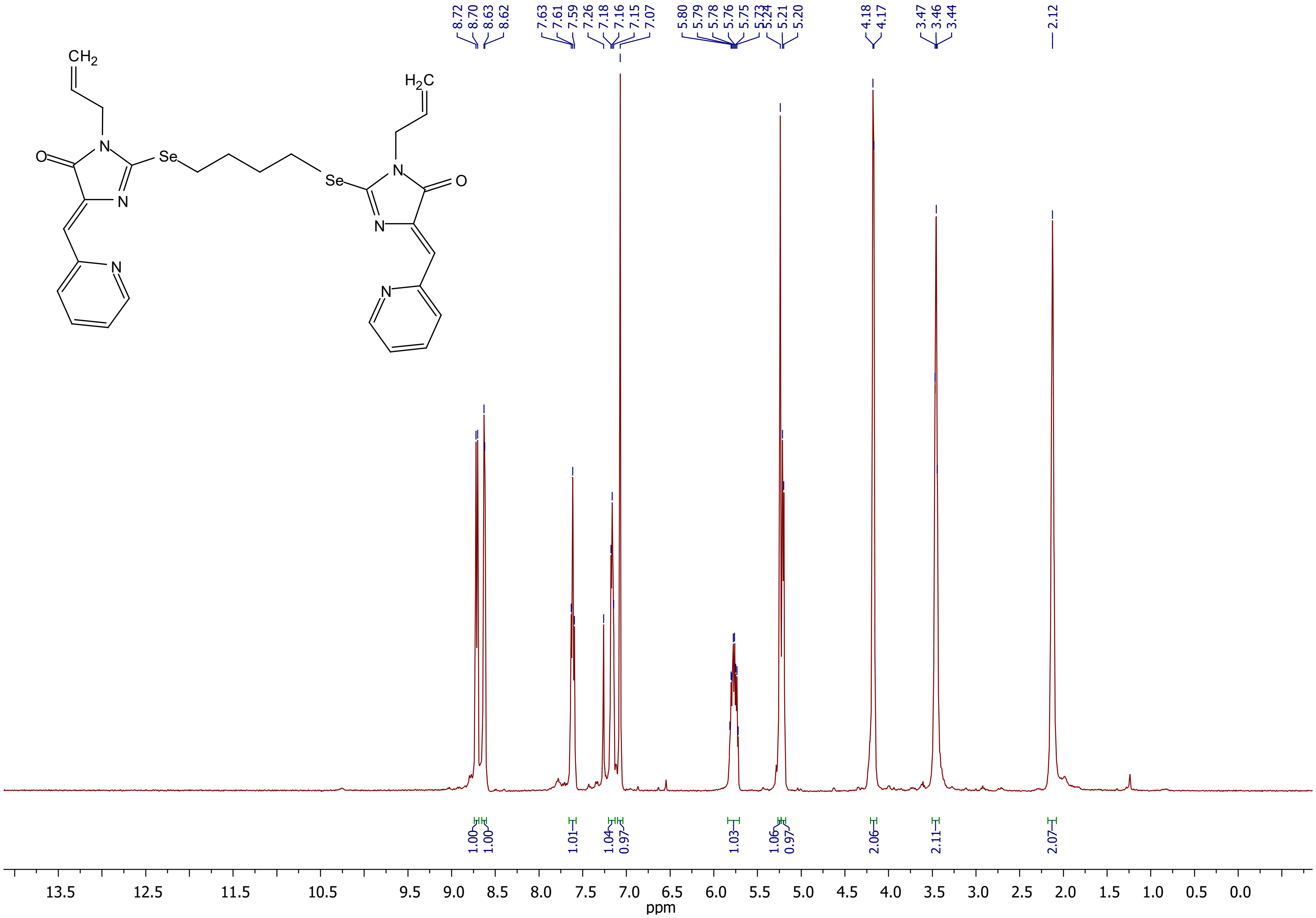

NMR ^{13}C (4Z,4'Z)-2,2'-(butane-1,4-diylbis(selanediyl))bis(1-allyl-4-(pyridin-2-ylmethylene)-1H-imidazol-5(4H)-one) (**4f**).

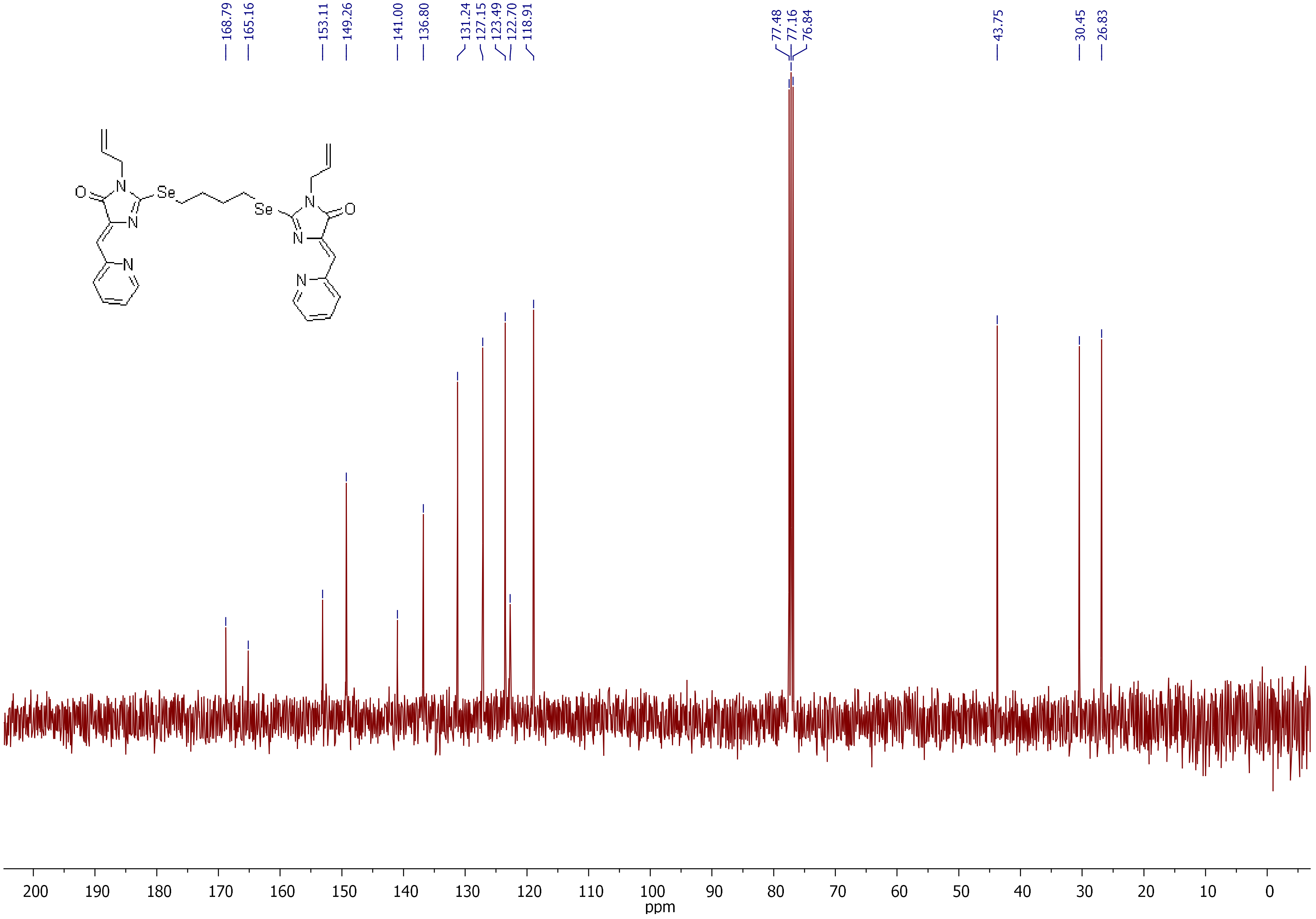

FTIR (4Z,4'Z)-2,2'-(butane-1,4-diylbis(selanediyl))bis(1-allyl-4-(pyridin-2-ylmethylene)-1H-imidazol-5(4H)-one) (**4e**).

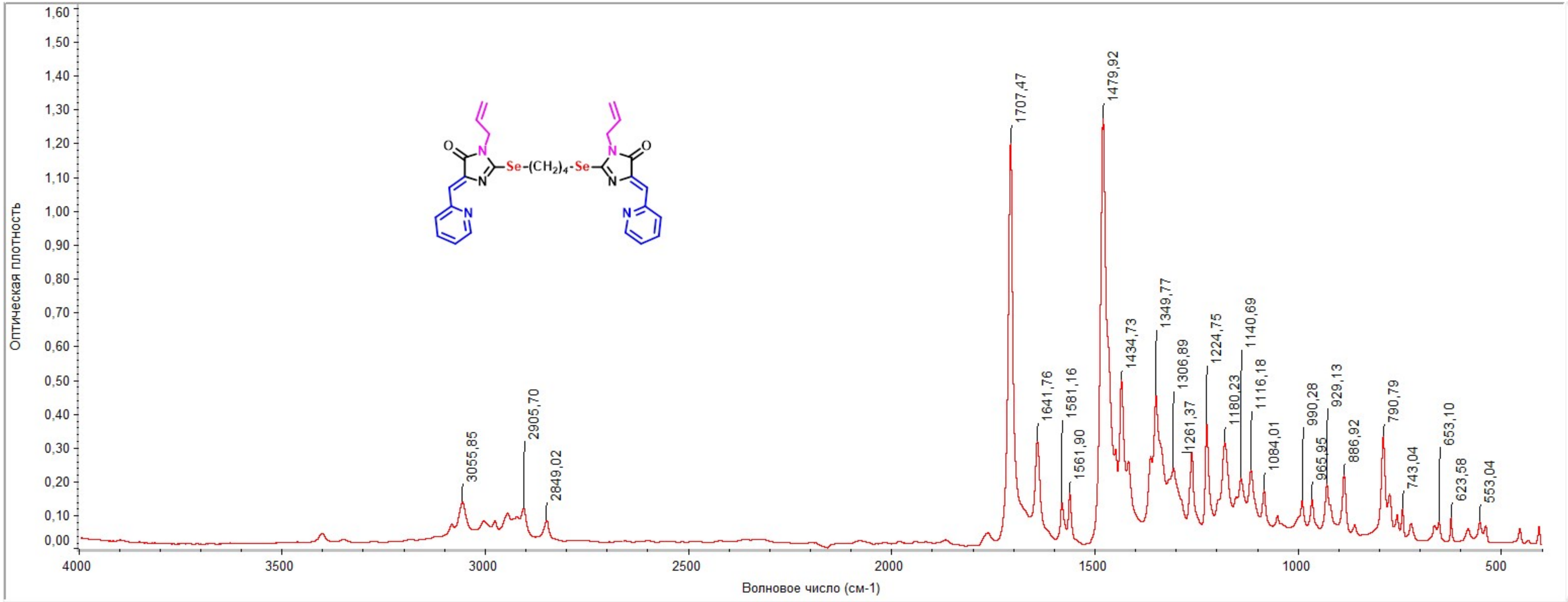

NMR ^{1}H (4Z,4'Z)-2,2'-(butane-1,4-diylbis(selanediyl))bis(1-cyclopropyl-4-(pyridin-2-ylmethylene)-1H-imidazol-5(4H)-one) (**4g**).

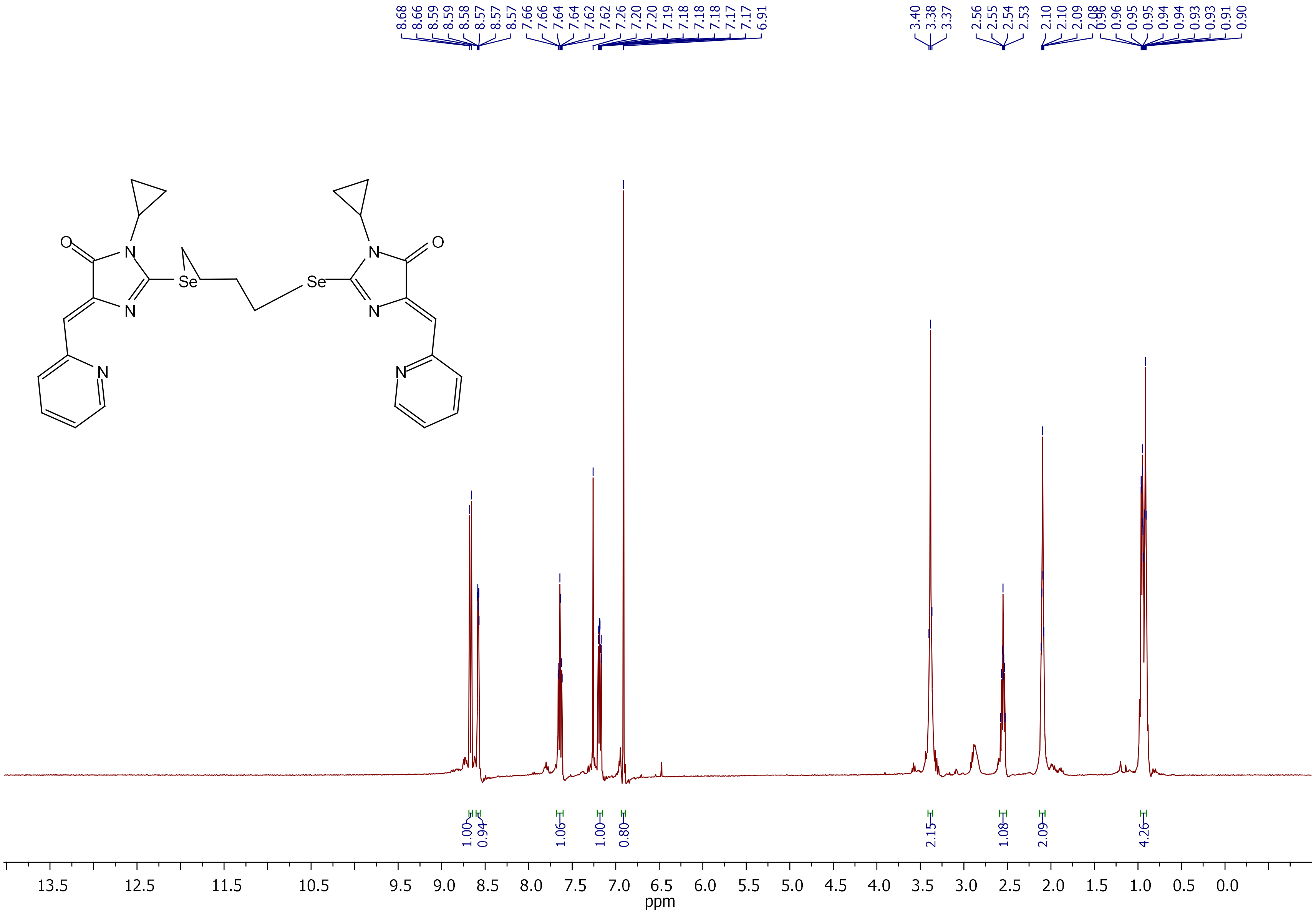

NMR ^{13}C (4Z,4'Z)-2,2'-(butane-1,4-diylbis(selanediyl))bis(1-cyclopropyl-4-(pyridin-2-ylmethylene)-1H-imidazol-5(4H)-one) (**4g**).

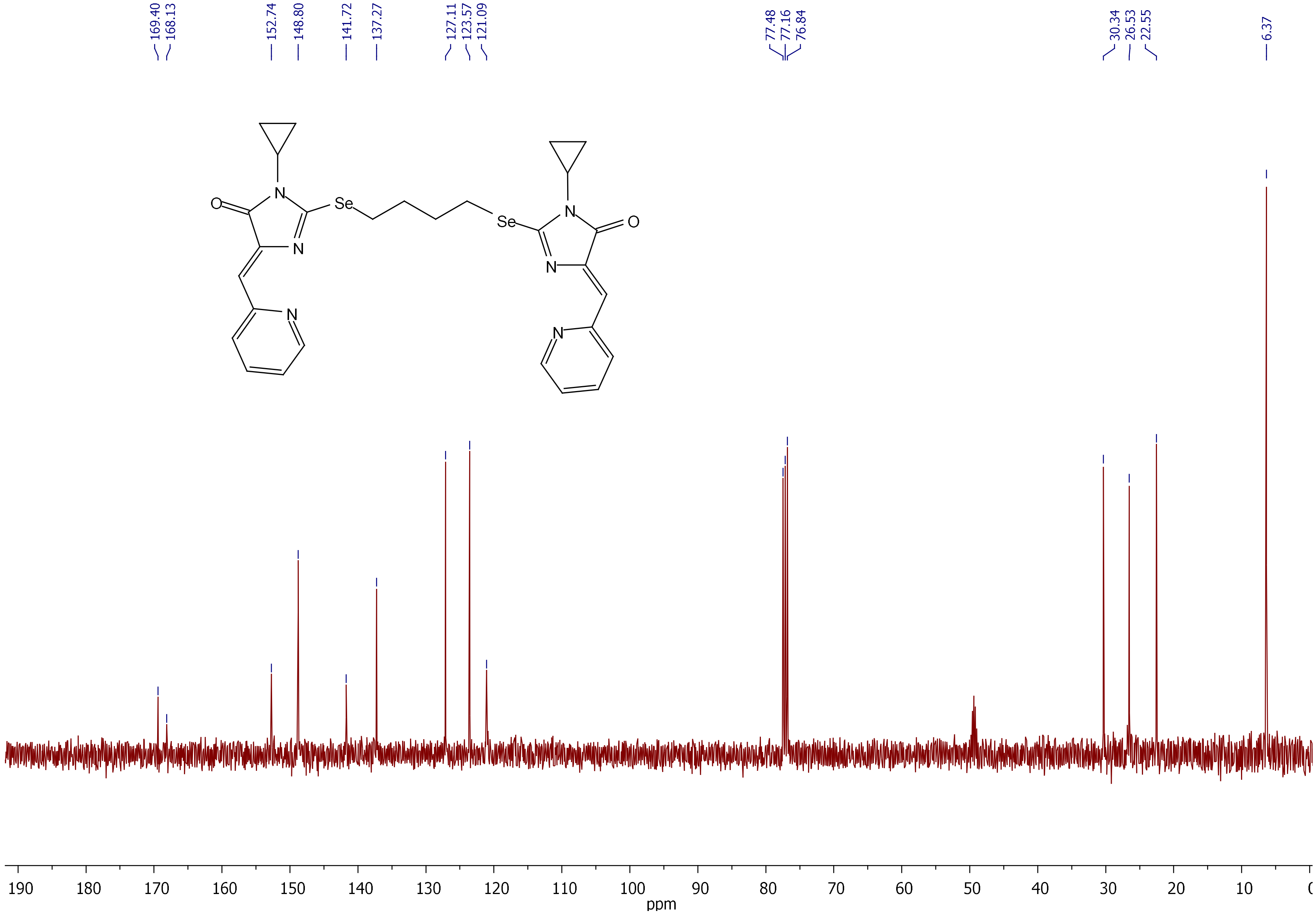

FTIR (4Z,4'Z)-2,2'-(butane-1,4-diylbis(selanediyl))bis(1-cyclopropyl-4-(pyridin-2-ylmethylene)-1H-imidazol-5(4H)-one) (**4g**).

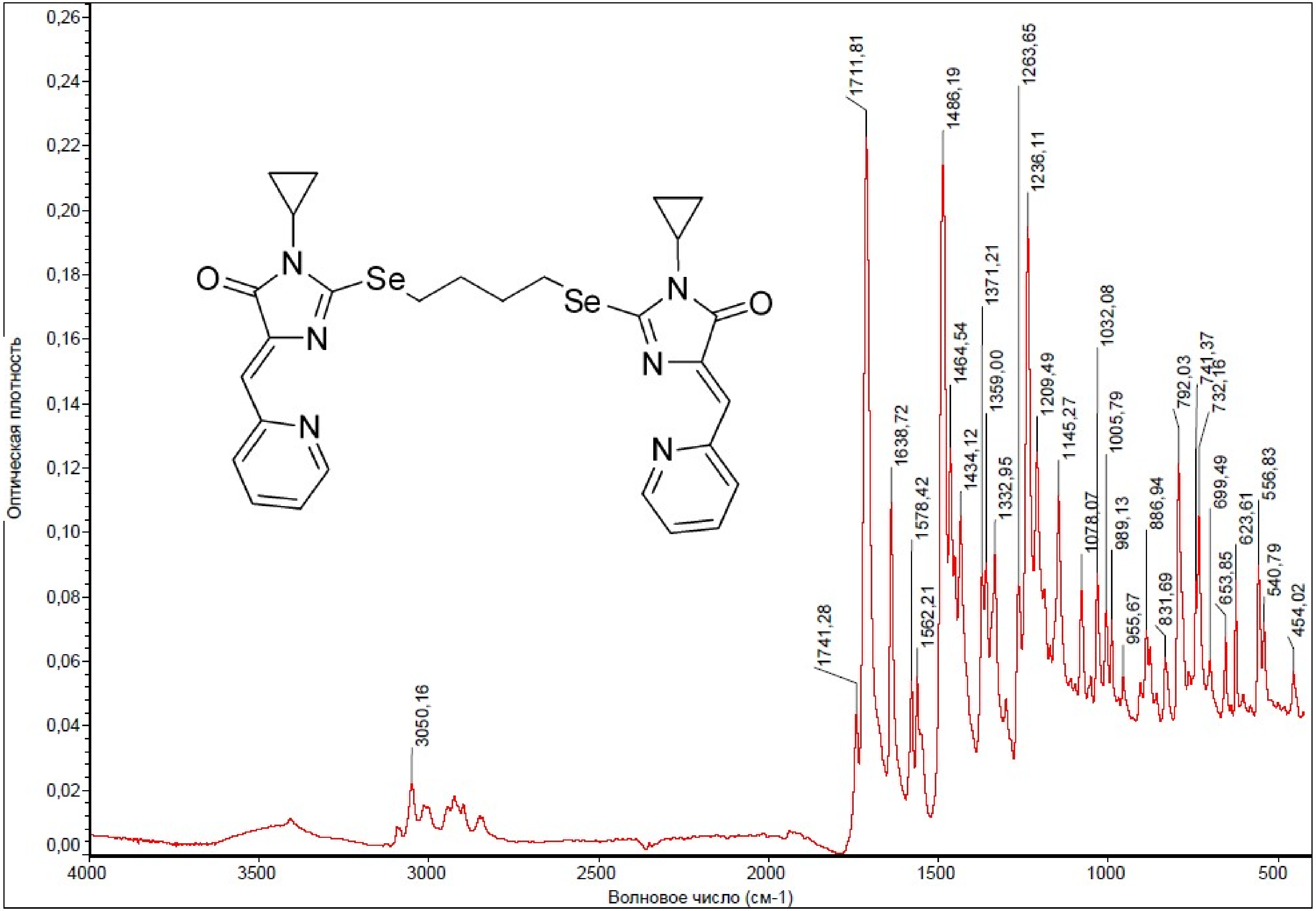

NMR ^{1}H (4Z,4'Z)-2,2'-(hexane-1,6-diylbis(selanediyl))bis(1-allyl-4-(pyridin-2-ylmethylene)-1H-imidazol-5(4H)-one) (**4h**).

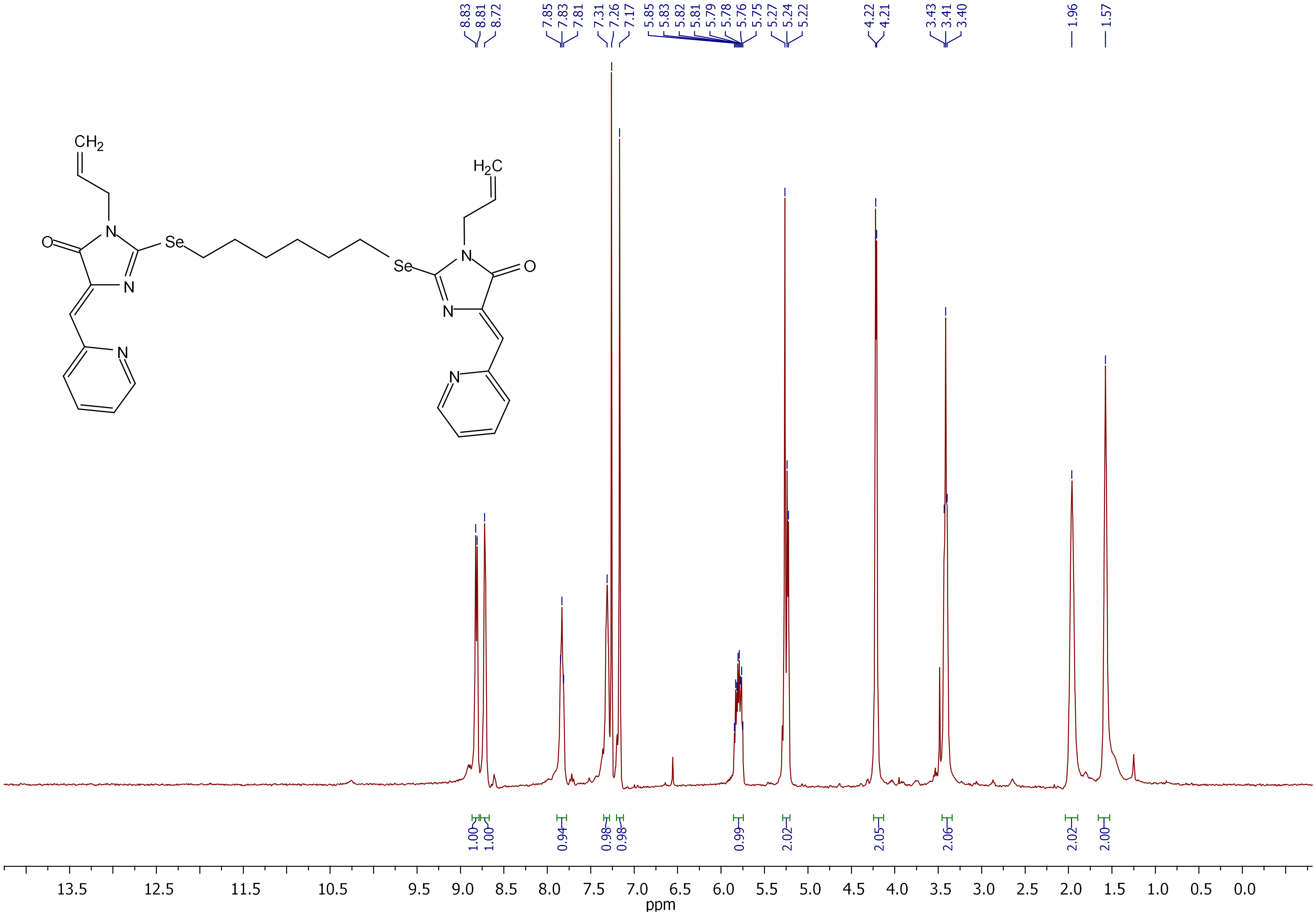

NMR ^{13}C (4Z,4'Z)-2,2'-(hexane-1,6-diylbis(selanediyl))bis(1-allyl-4-(pyridin-2-ylmethylene)-1H-imidazol-5(4H)-one) (**4h**).

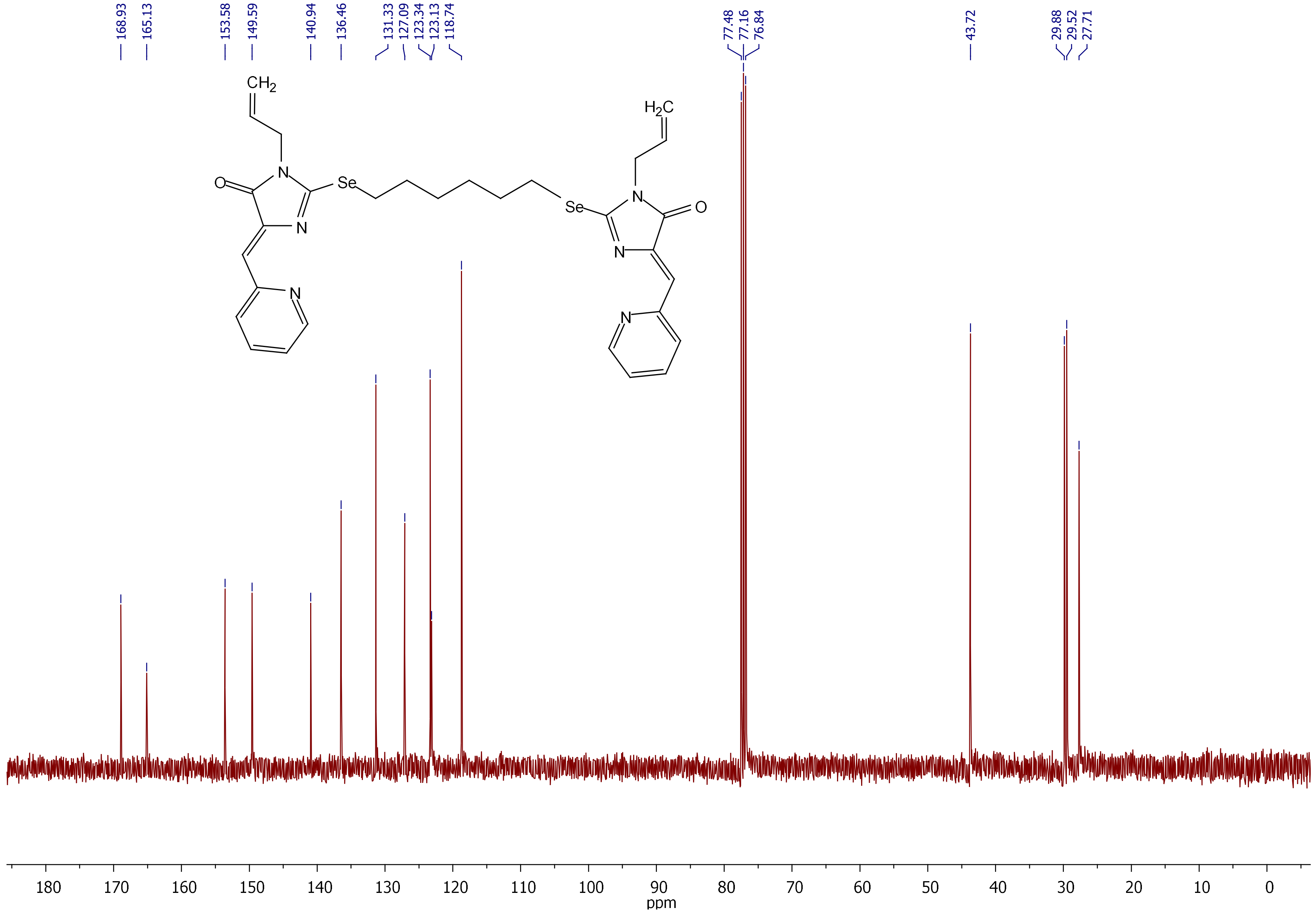

FTIR (4Z,4'Z)-2,2'-(hexane-1,6-diylbis(selanediyl))bis(1-allyl-4-(pyridin-2-ylmethylene)-1H-imidazol-5(4H)-one) (**4h**).

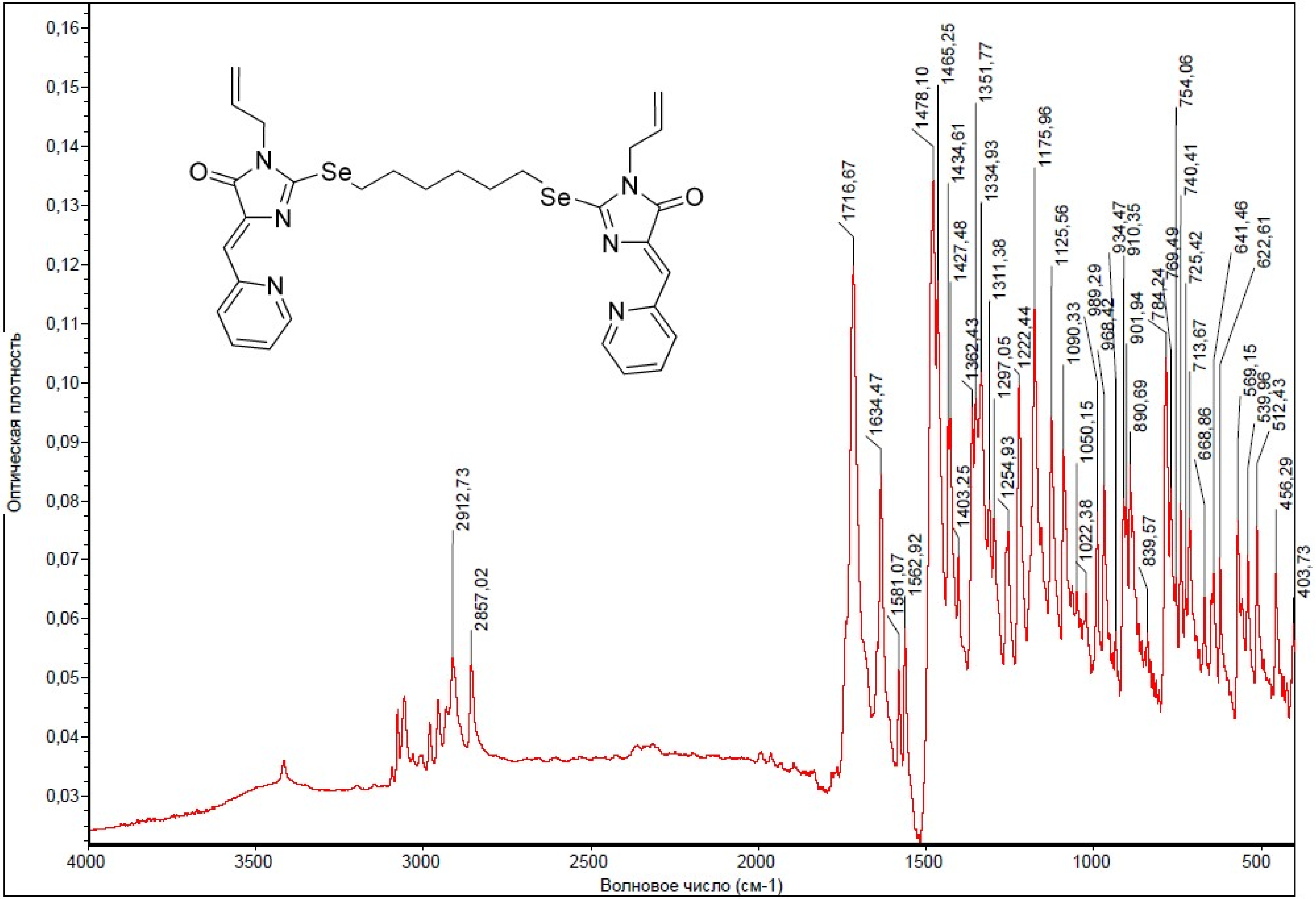

NMR ¹H (4Z,4'Z)-2,2'-(hexane-1,6-diylbis(selanediyl))bis(1-cyclopropyl-4-(pyridin-2-ylmethylene)-1H-imidazol-5(4H)-one) (**4i**).

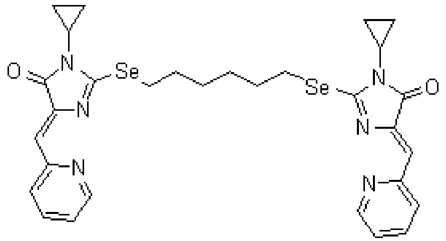

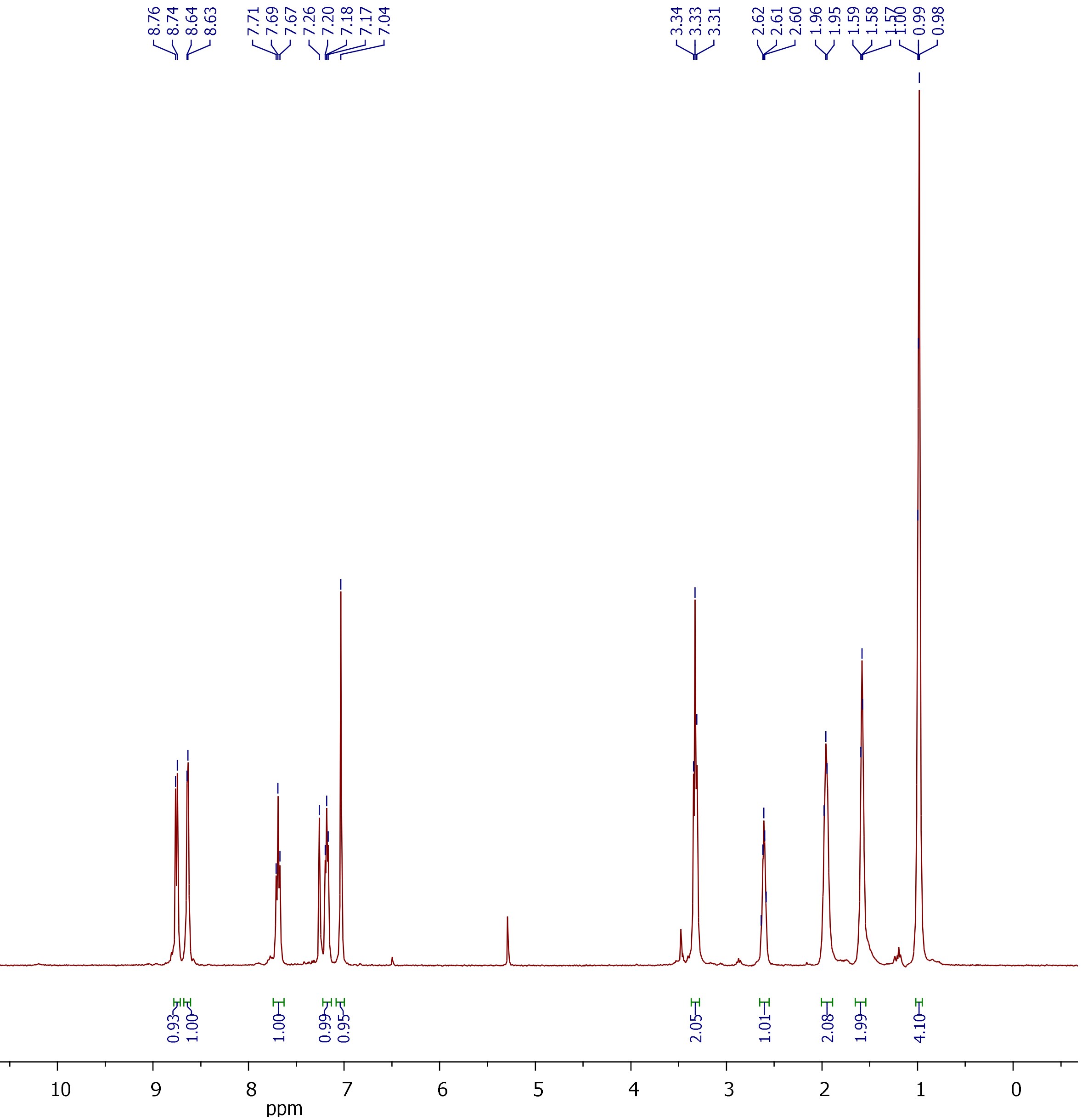

NMR ^{13}C (4Z,4'Z)-2,2'-(hexane-1,6-diylbis(selanediyl))bis(1-cyclopropyl-4-(pyridin-2-ylmethylene)-1H-imidazol-5(4H)-one) (**4i**).

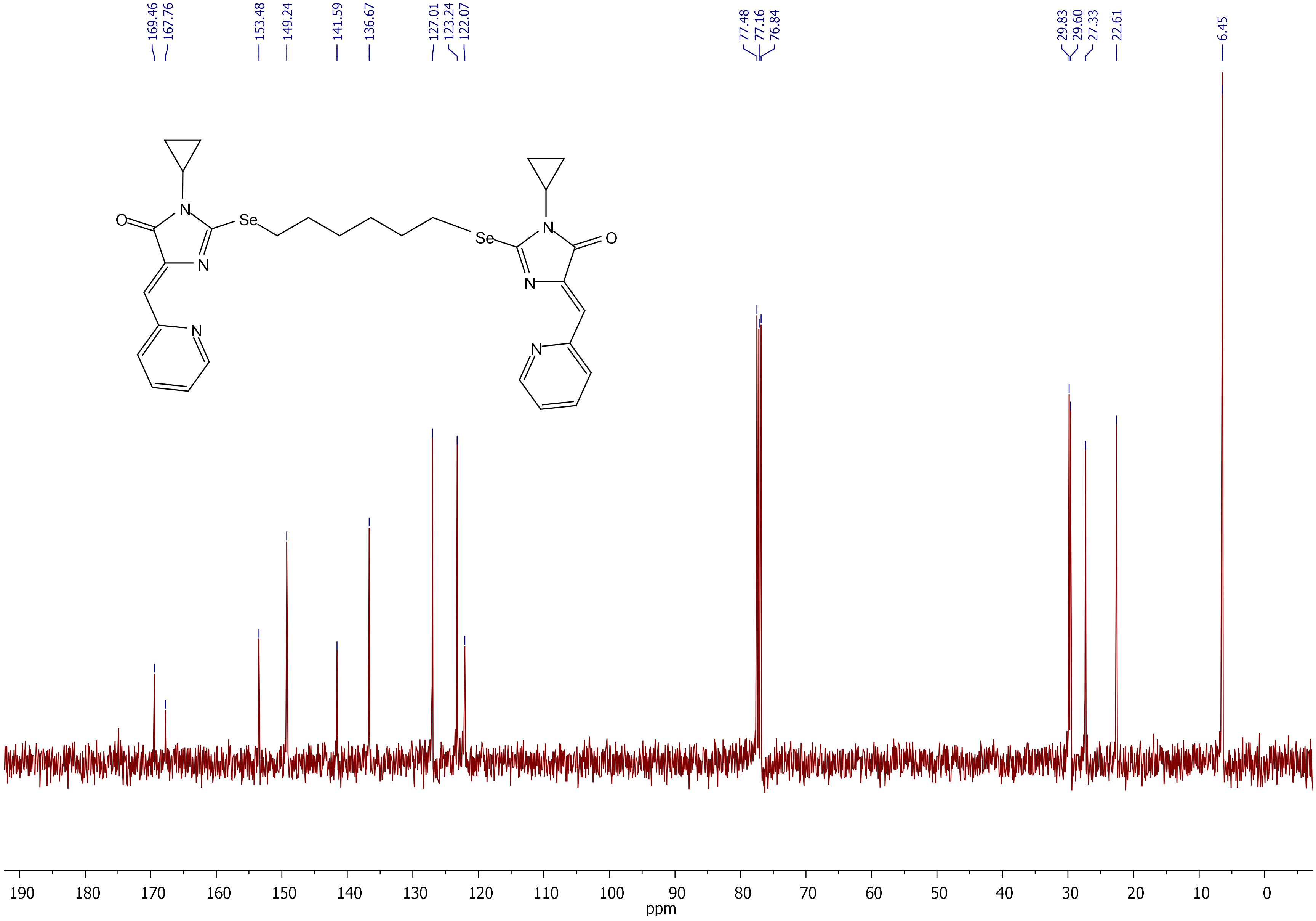

FTIR (4Z,4'Z)-2,2'-(hexane-1,6-diylbis(selanediyl))bis(1-cyclopropyl-4-(pyridin-2-ylmethylene)-1H-imidazol-5(4H)-one) (**4i**).

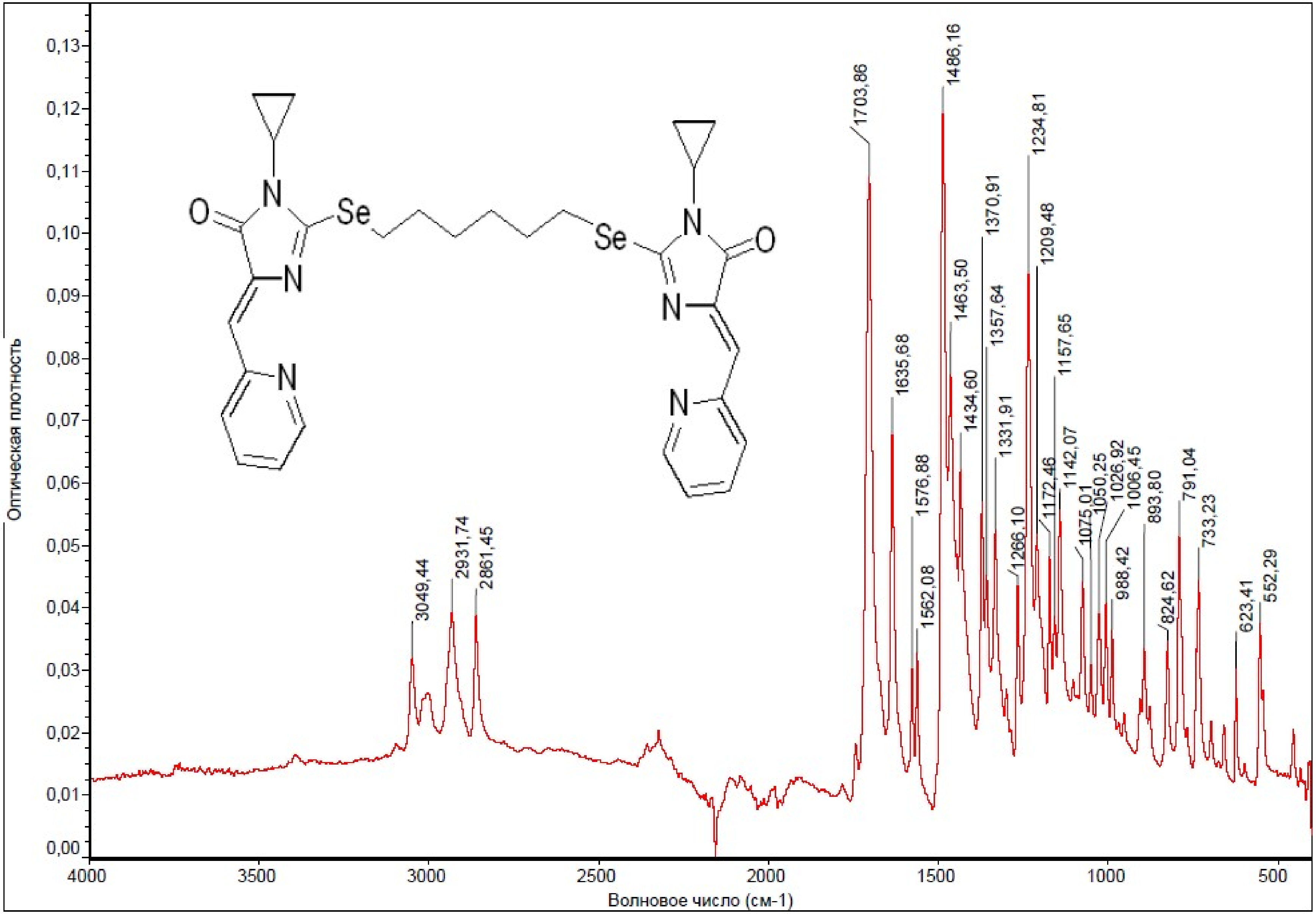

NMR ^{1}H (4Z,4'Z)-2,2'-(decane-1,10-diylbis(selanediyl))bis(1-allyl-4-(pyridin-2-ylmethylene)-1H-imidazol-5(4H)-one) (**4j**).

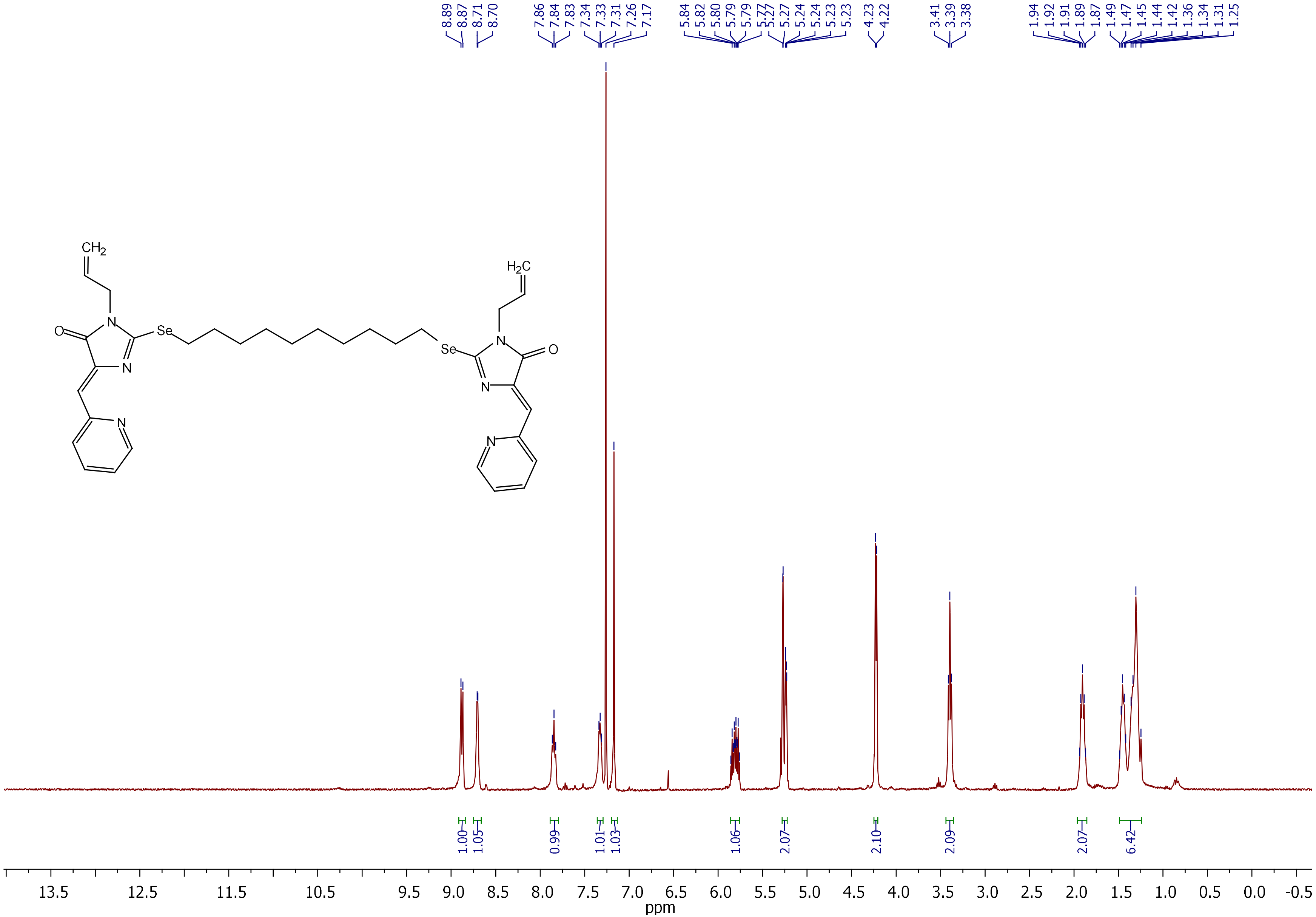

NMR ^{13}C *(4Z,4'Z)-2,2'-(decane-1,10-diylbis(selanediyl))bis(1-allyl-4-(pyridin-2-ylmethylene)-1H-imidazol-5(4H)-one)* (**4j**).

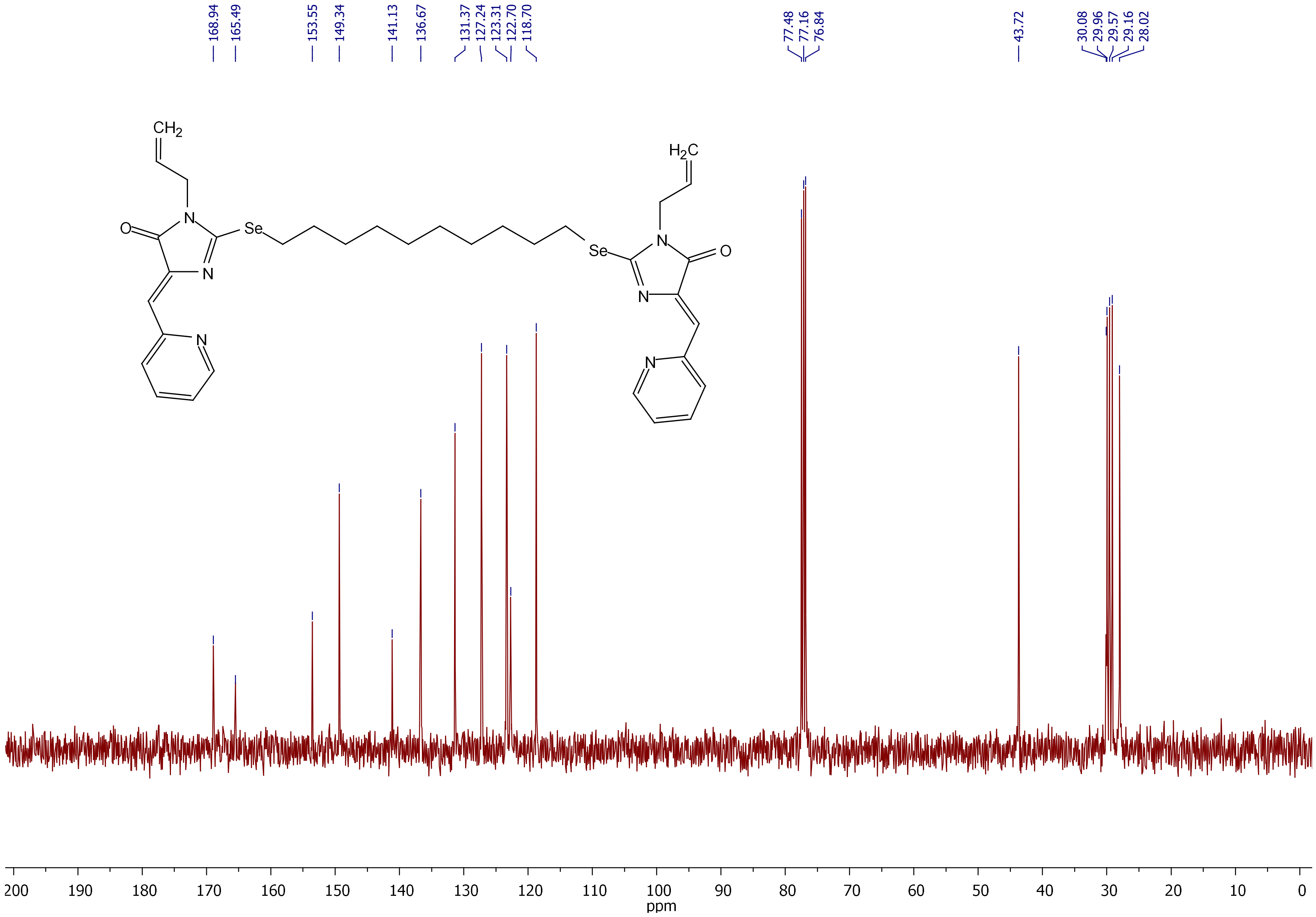

FTIR (4Z,4'Z)-2,2'-(decane-1,10-diylbis(selanediyl))bis(1-allyl-4-(pyridin-2-ylmethylene)-1H-imidazol-5(4H)-one) (**4j**).

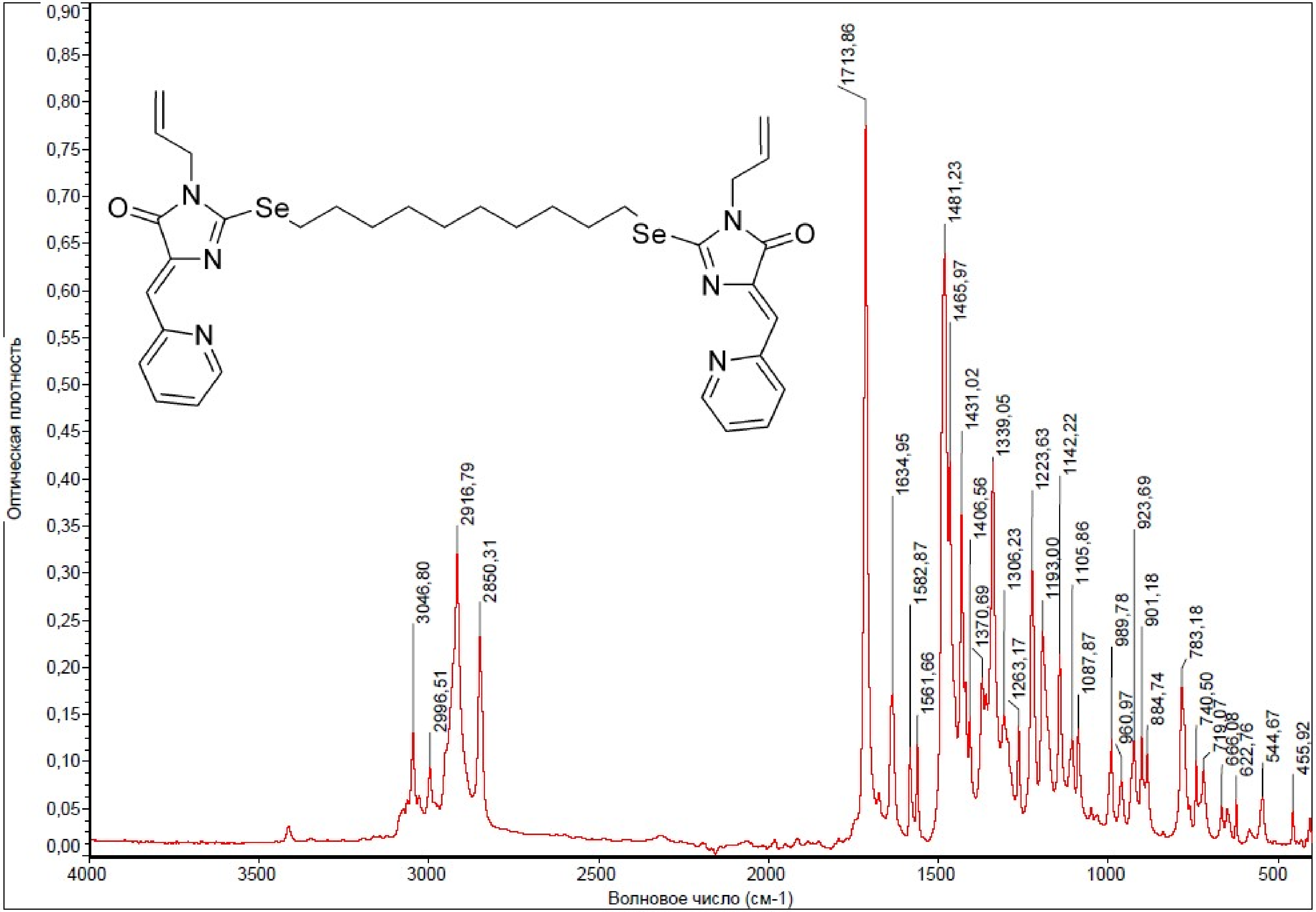

NMR ^{1}H(4Z,4'Z)-2,2'-(decane-1,10-diylbis(selanediyl))bis(1-cyclopropyl-4-(pyridin-2-ylmethylene)-1H-imidazol-5(4H)-one) (**4k**).

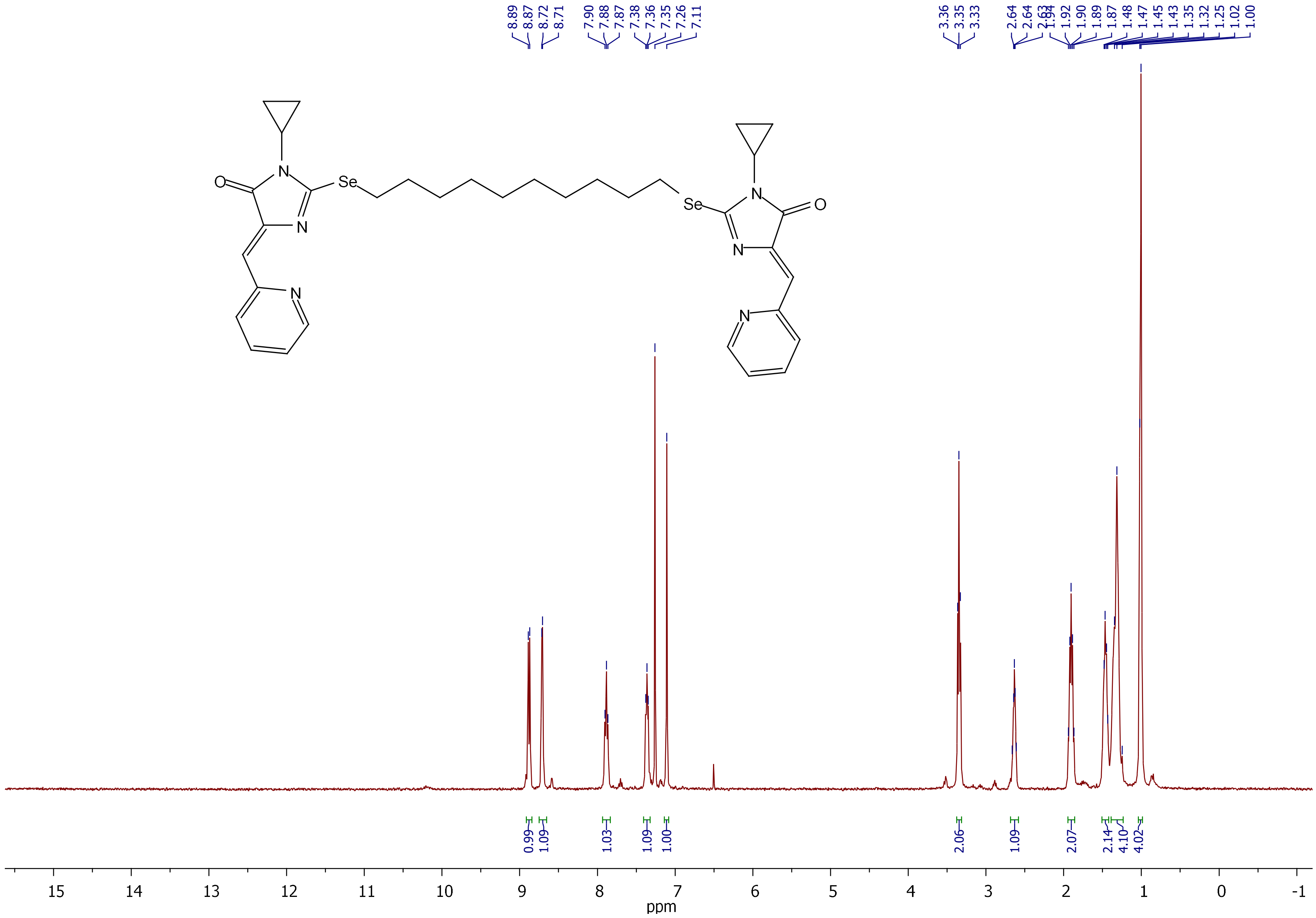

NMR ^{13}C (4Z,4'Z)-2,2'-(decane-1,10-diylbis(selanediyl))bis(1-cyclopropyl-4-(pyridin-2-ylmethylene)-1H-imidazol-5(4H)-one) (**4k**).

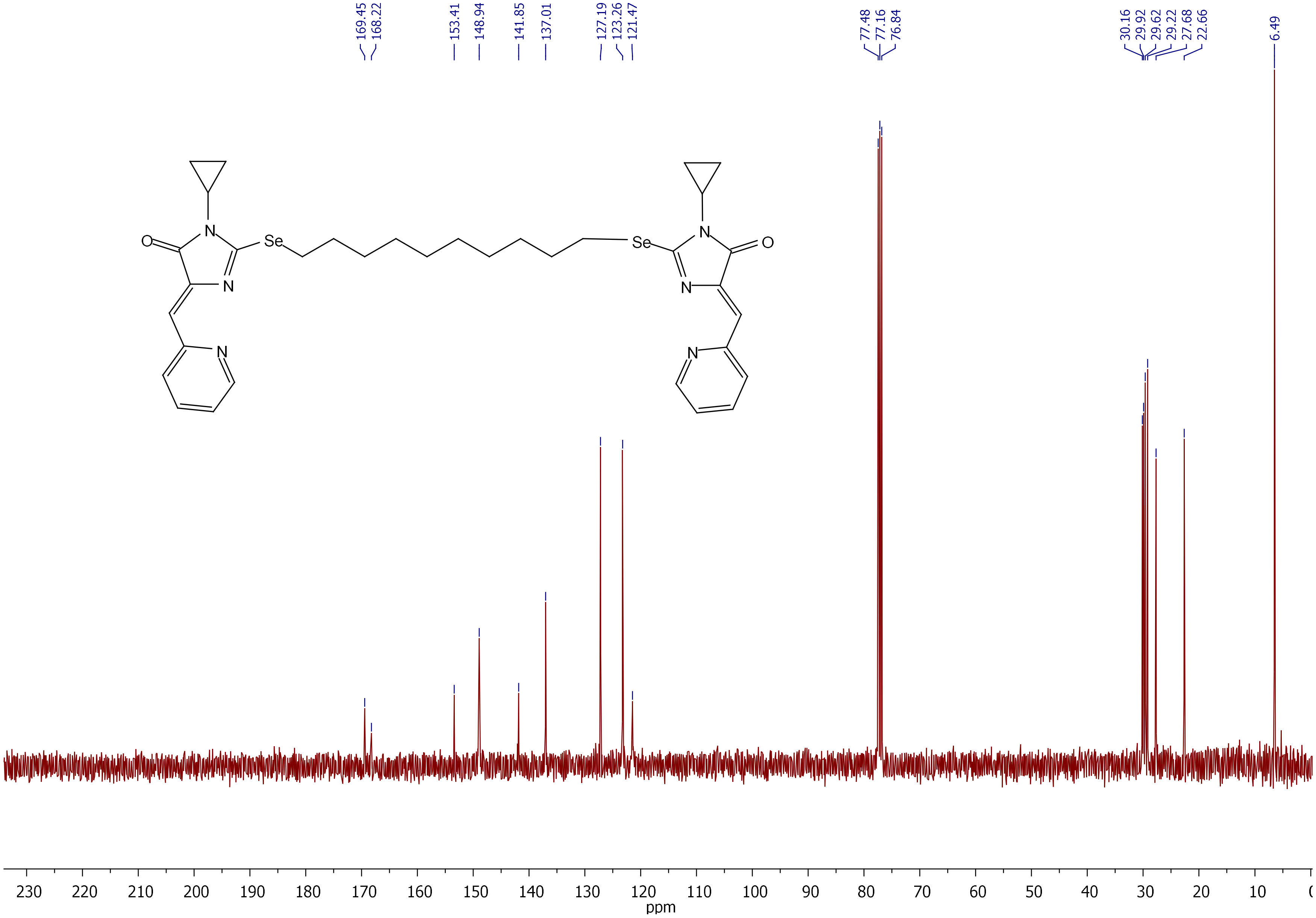

FTIR (4Z,4'Z)-2,2'-(decane-1,10-diylbis(selanediyl))bis(1-cyclopropyl-4-(pyridin-2-ylmethylene)-1H-imidazol-5(4H)-one) (**4k**).

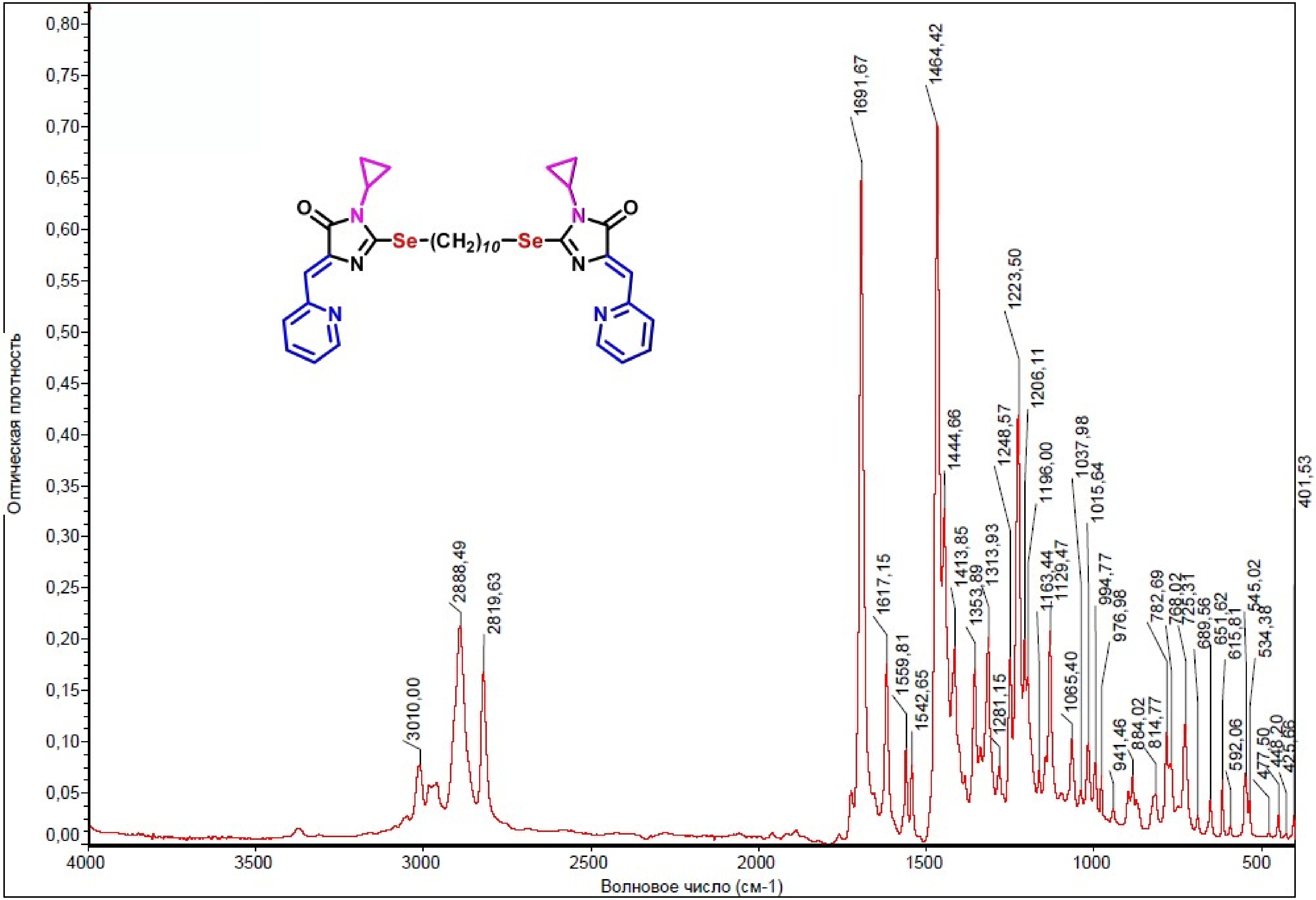

FTIR (4Z,4'Z)-2,2'-(Ethane-1,2-diylbis(selanediyl))bis(1-(4-ethoxyphenyl)-4-(pyridin-2-ylmethylene)-1H-imidazol-5(4H)-one)dicopper(I,II) trichloride **(5a)**

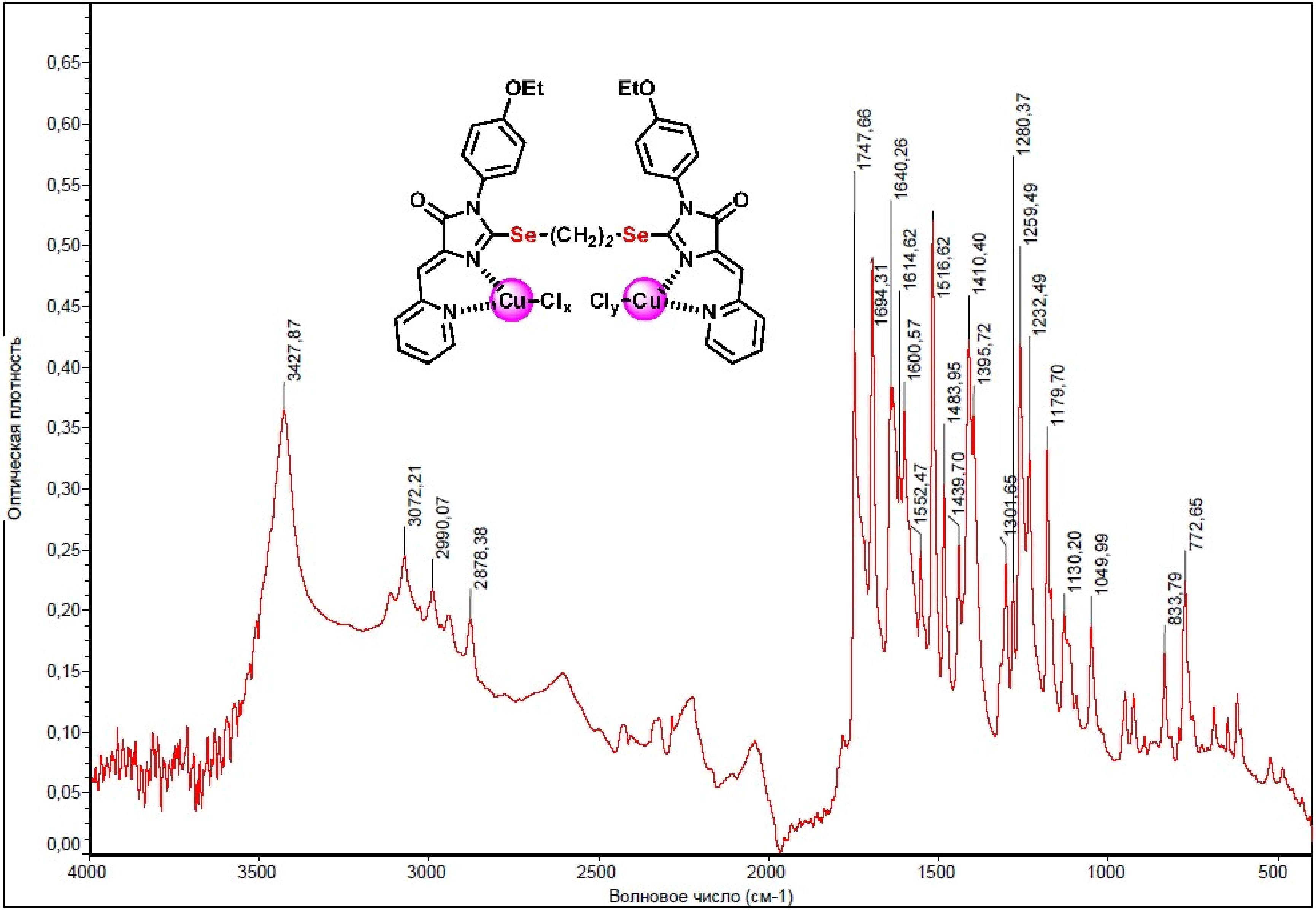

FTIR (4Z,4'Z)-2,2'-(Ethane-1,2-diylbis(selanediyl))bis(1-(4-cyanophenyl)-4-(pyridin-2-ylmethylene)-1H-imidazol-5(4H)-one)dicopper(I) dichloride **(5b)**

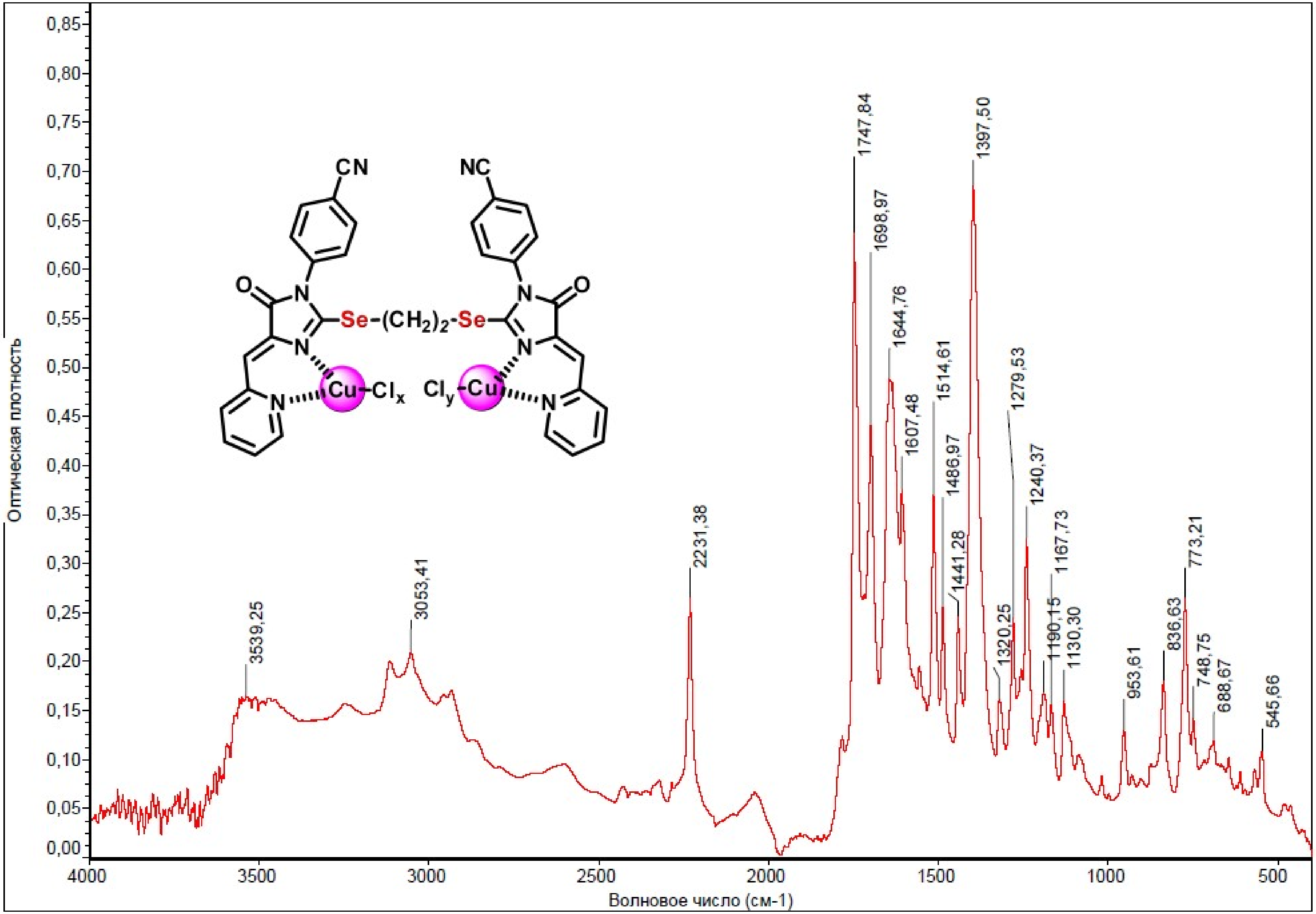

FTIR (4Z,4'Z)-2,2'-(Ethane-1,2-diylbis(selanediyl))bis(1-(4-allyl)-4-(pyridin-2-ylmethylene)-1H-imidazol-5(4H)-one)dicopper(I) dichloride **(5d).**

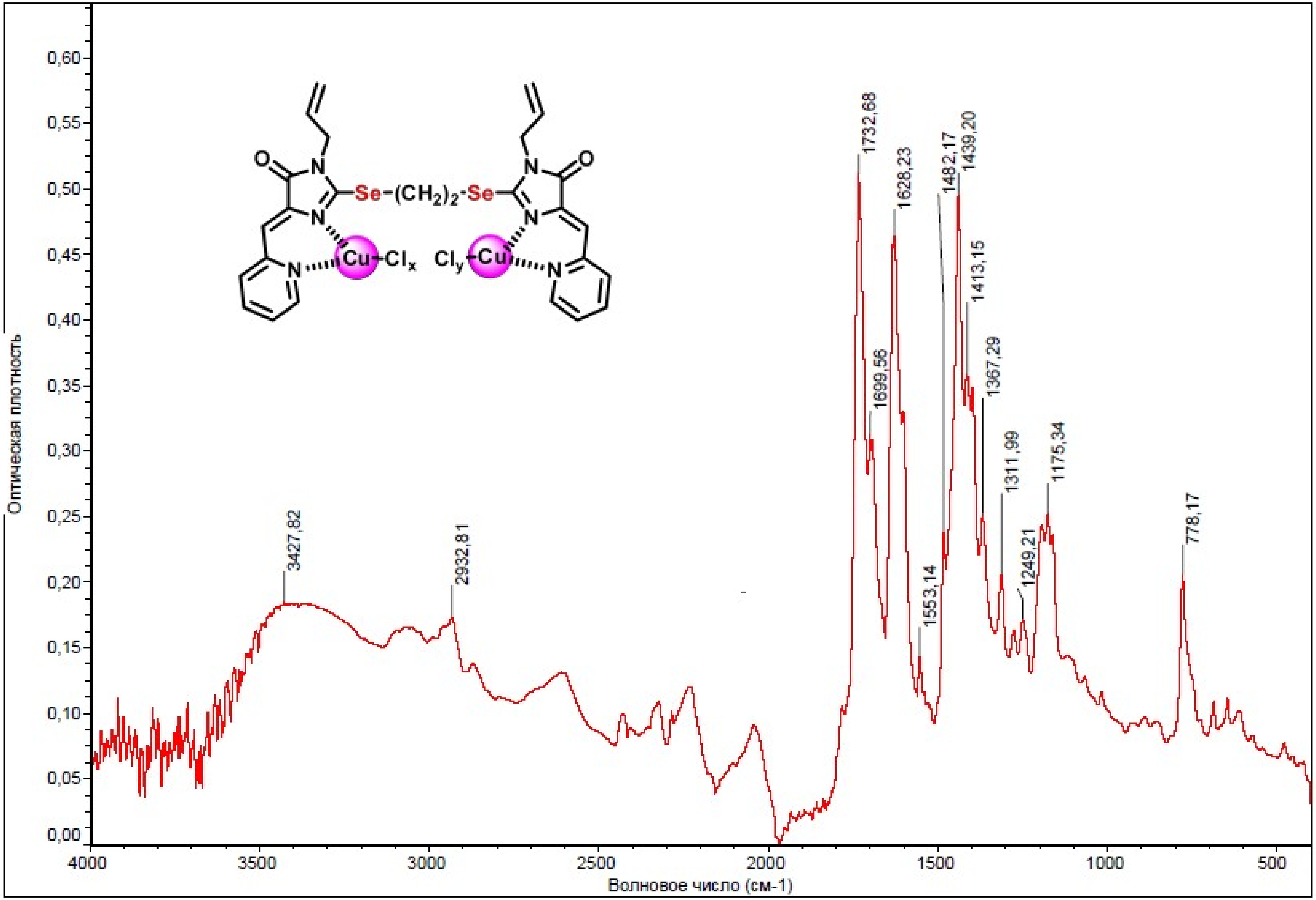

FTIR (4Z,4'Z)-2,2'-(Ethane-1,2-diylbis(selanediyl))bis(1-(4-cyclopropyl)-4-(pyridin-2-ylmethylene)-1H-imidazol-5(4H)-one)dicopper(I,II) trichloride **(5e).**

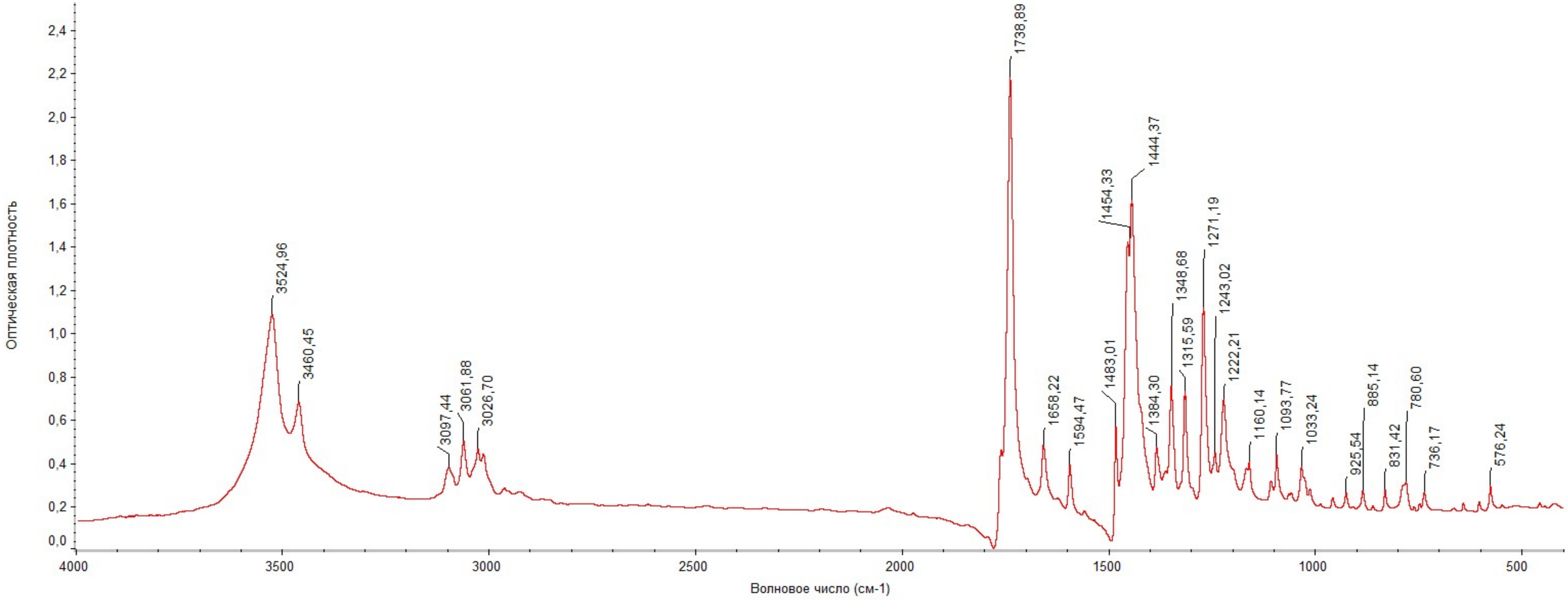

FTIR (4Z,4'Z)-2,2'-(butane-1,4-diylbis(selanediyl))bis(1-allyl-4-(pyridin-2-ylmethylene)-1H-imidazol-5(4H)-one)dicopper(I,II) trichloride **(5f).**

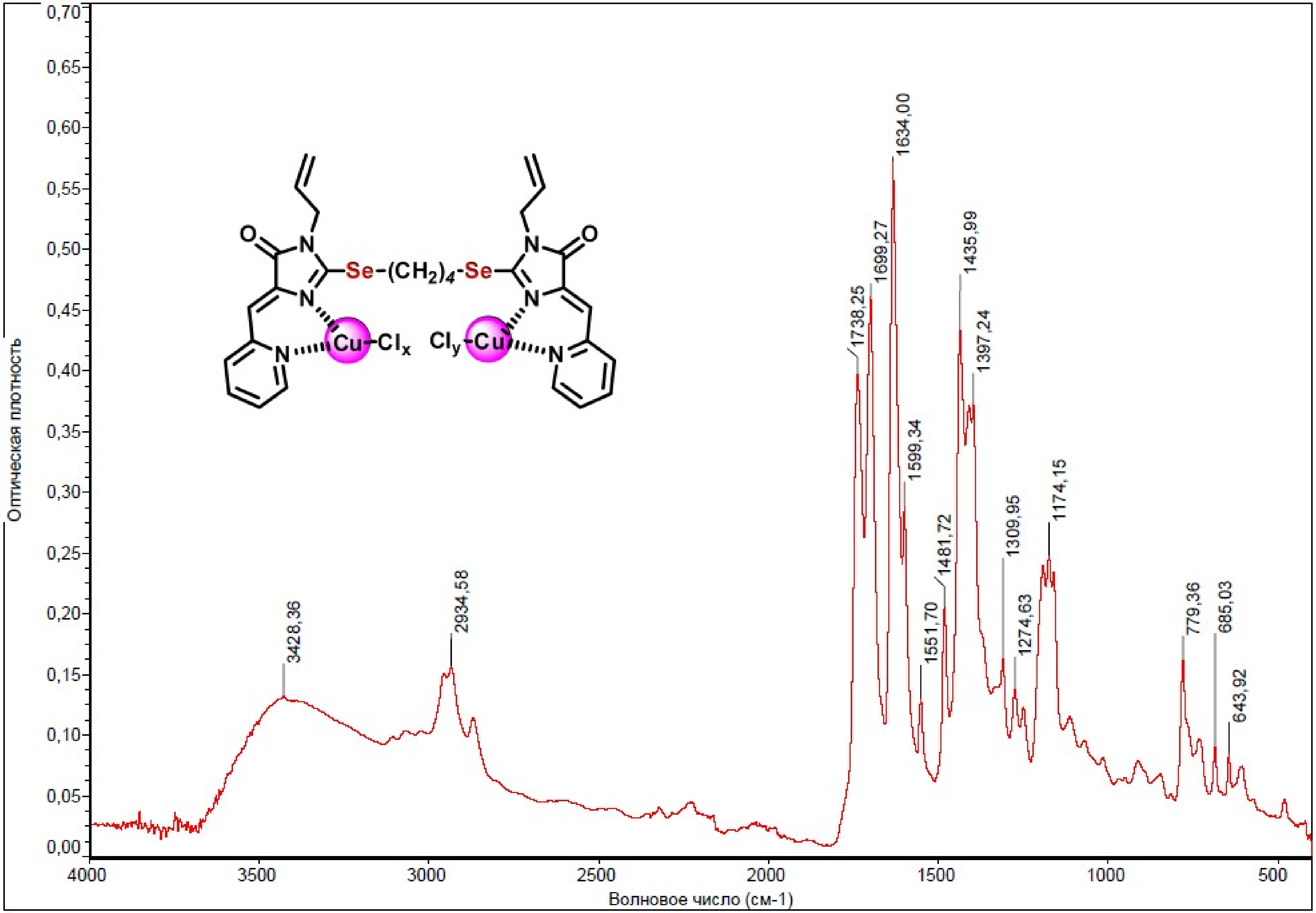

FTIR (4Z,4'Z)-2,2'-(hexane-1,6-diylbis(selanediyl))bis(1-allyl-4-(pyridin-2-ylmethylene)-1H-imidazol-5(4H)-one)dicopper(I,II) trichloride **(5h).**

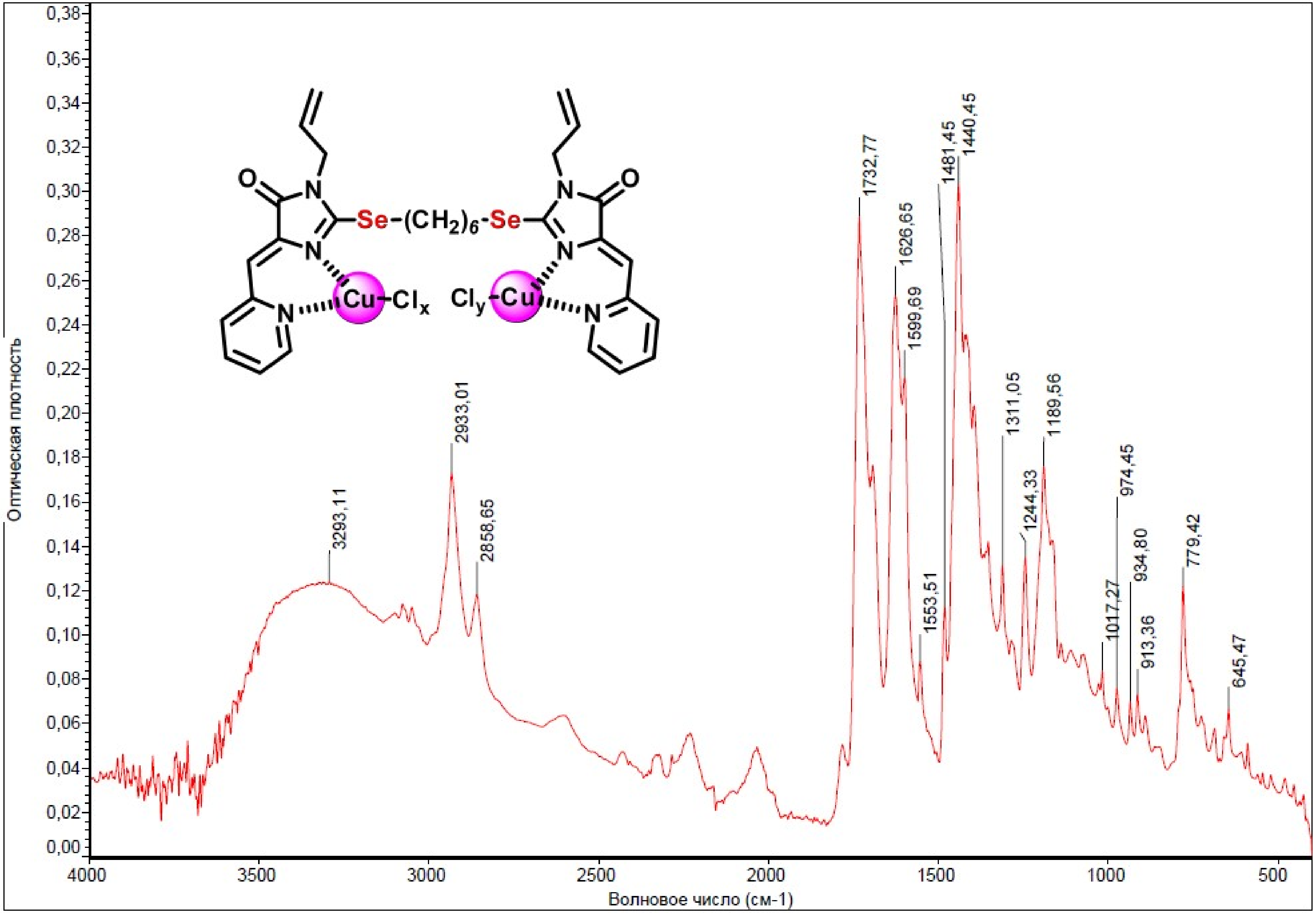

FTIR (4Z,4'Z)-2,2'-(hexane-1,6-diylbis(selanediyl))bis(1-cyclopropyl-4-(pyridin-2-ylmethylene)-1H-imidazol-5(4H)-one)dicopper(II) tetrachloride **(5i).**

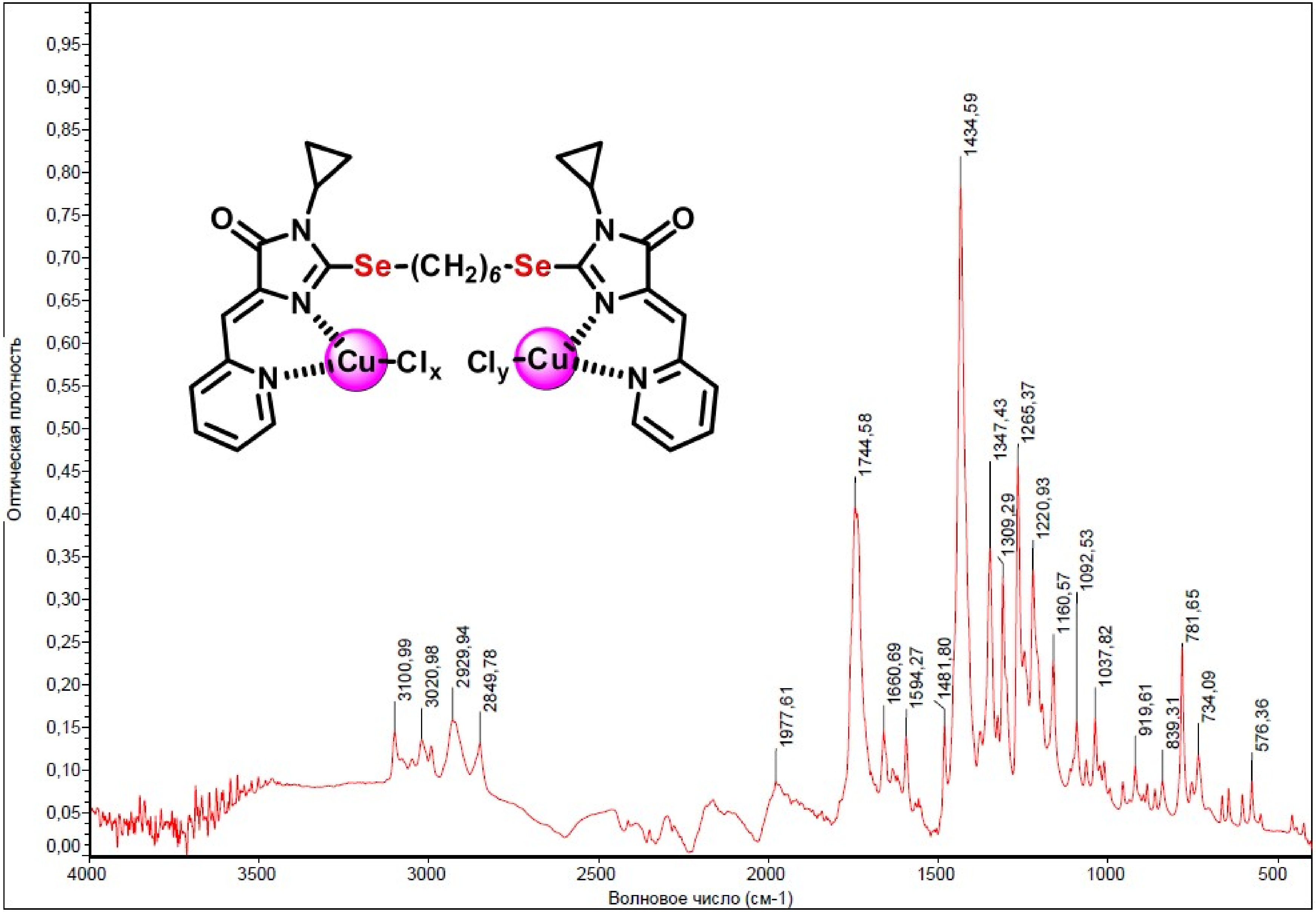

FTIR (4Z,4'Z)-2,2'-(decane-1,10-diylbis(selanediyl))bis(1-cyclopropyl-4-(pyridin-2-ylmethylene)-1H-imidazol-5(4H)-one)dicopper(I,II) trichloride **(5k).**

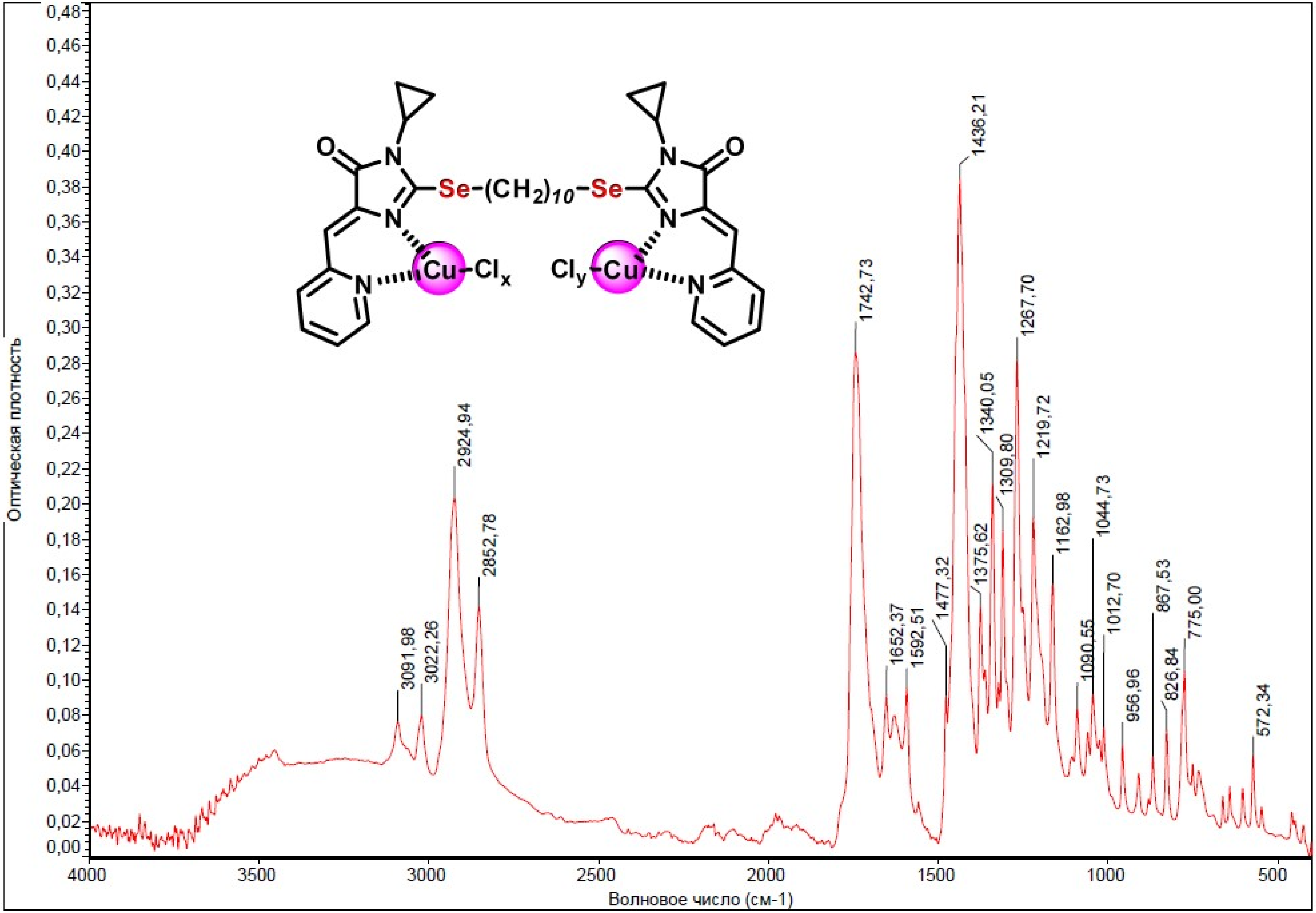

FTIR (4Z,4'Z)-2,2'-(Ethane-1,2-diylbis(selanediyl))bis(1-(4-allyl)-4-(pyridin-2-ylmethylene)-1H-imidazol-5(4H)-one)copper(II) diperchlorate **(6e)**

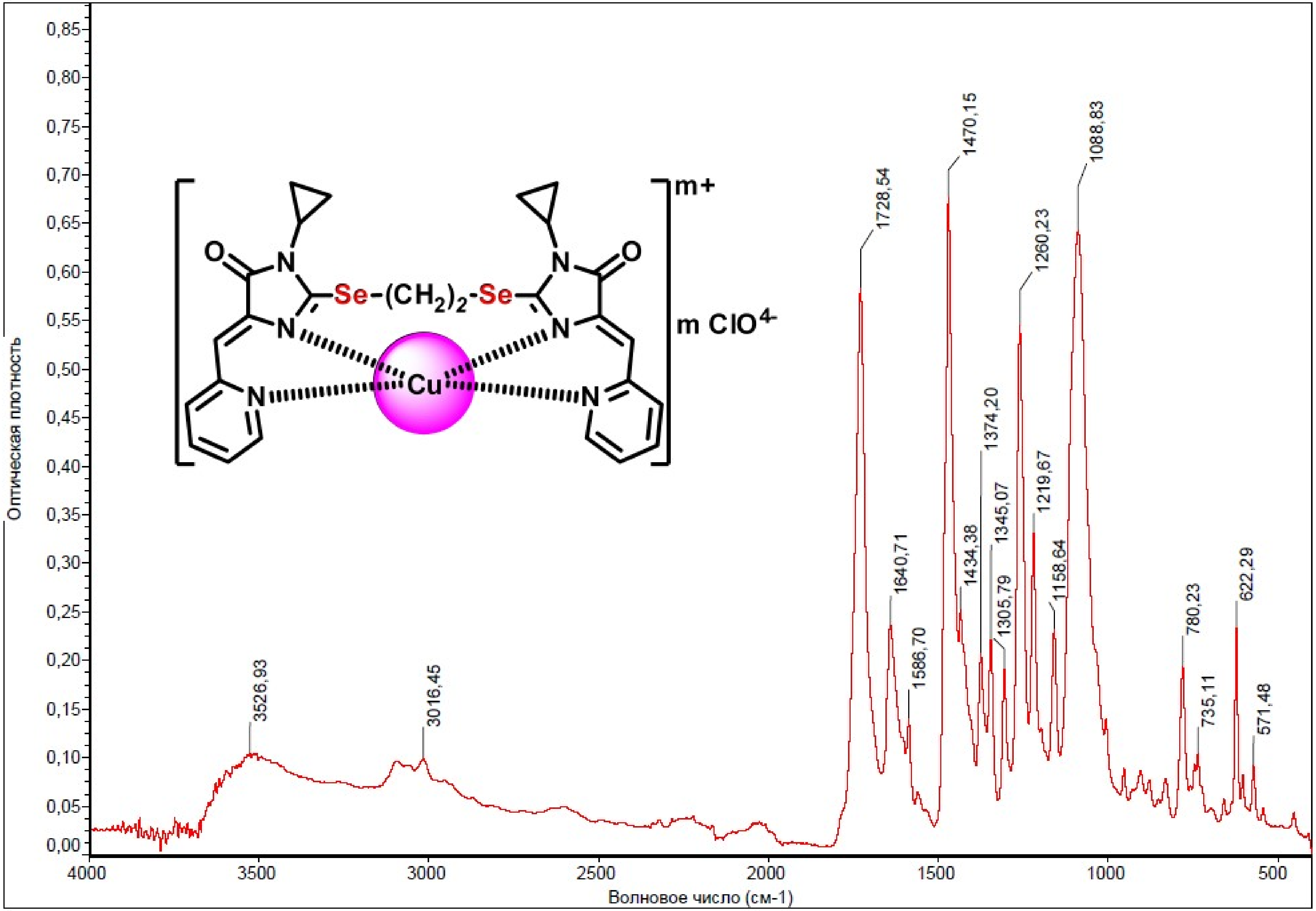

FTIR (4Z,4'Z)-2,2'-(butane-1,4-diylbis(selanediyl))bis(1-cyclopropyl-4-(pyridin-2-ylmethylene)-1H-imidazol-5(4H)-one)copper(II) diperchlorate **(6g).**

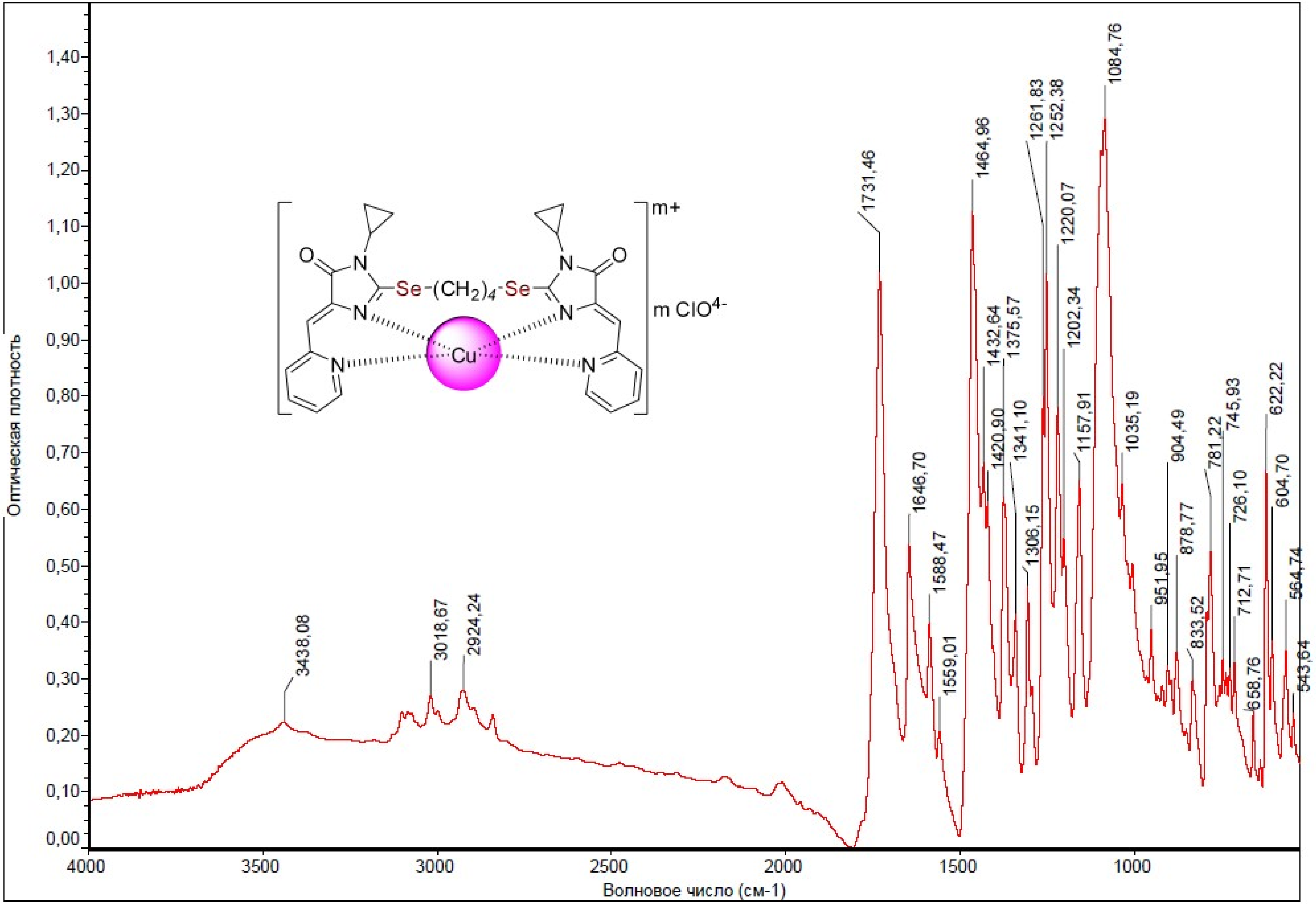

FTIR (4Z,4'Z)-2,2'-(hexane-1,6-diylbis(selanediyl))bis(1-allyl-4-(pyridin-2-ylmethylene)-1H-imidazol-5(4H)-one)copper(II) diperchlorate **(6h).**

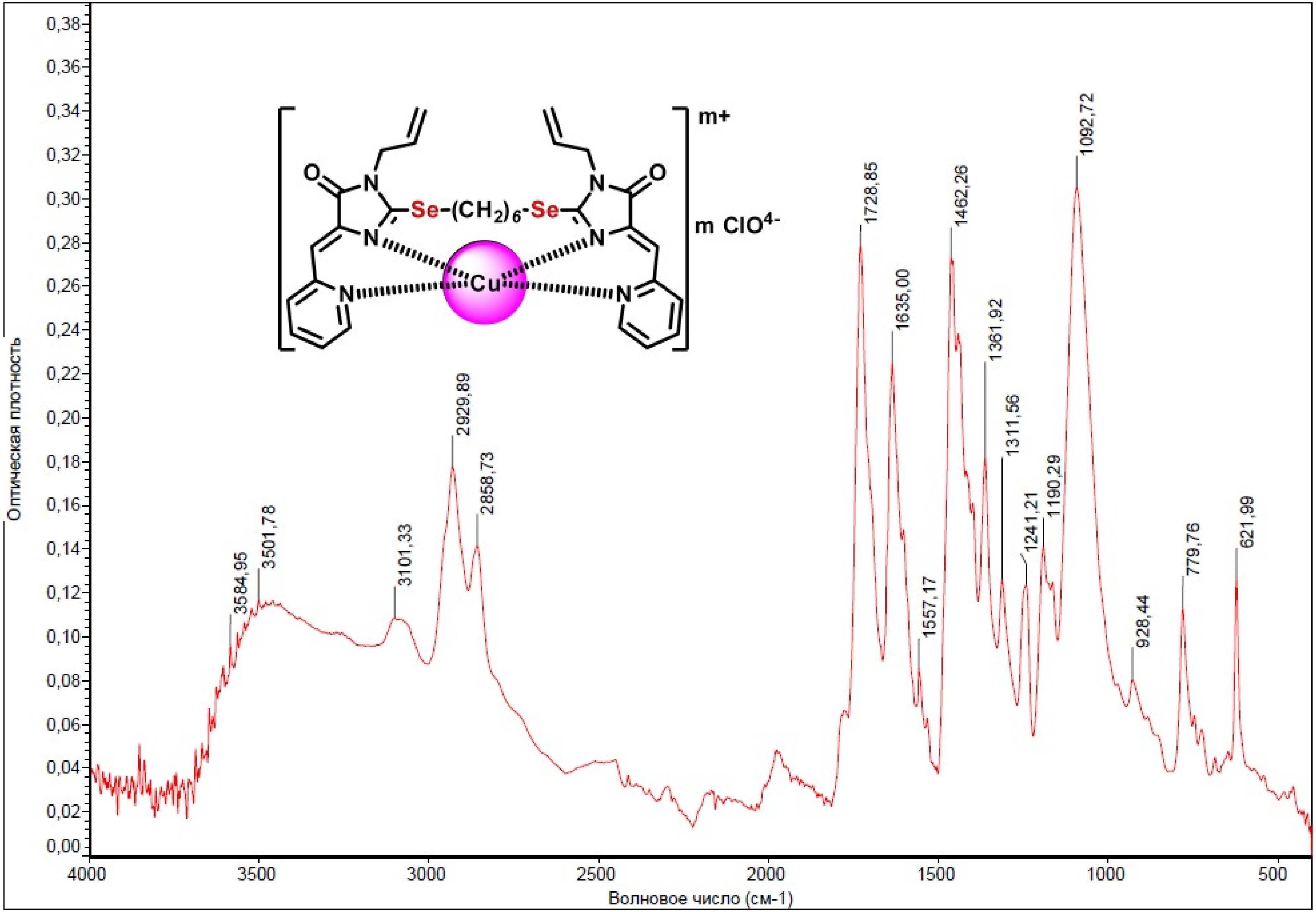

FTIR (4Z,4'Z)-2,2'-(decane-1,10-diylbis(selanediyl))bis(1-allyl-4-(pyridin-2-ylmethylene)-1H-imidazol-5(4H)-one)dicopper(I,II) trichloride **(6j).**

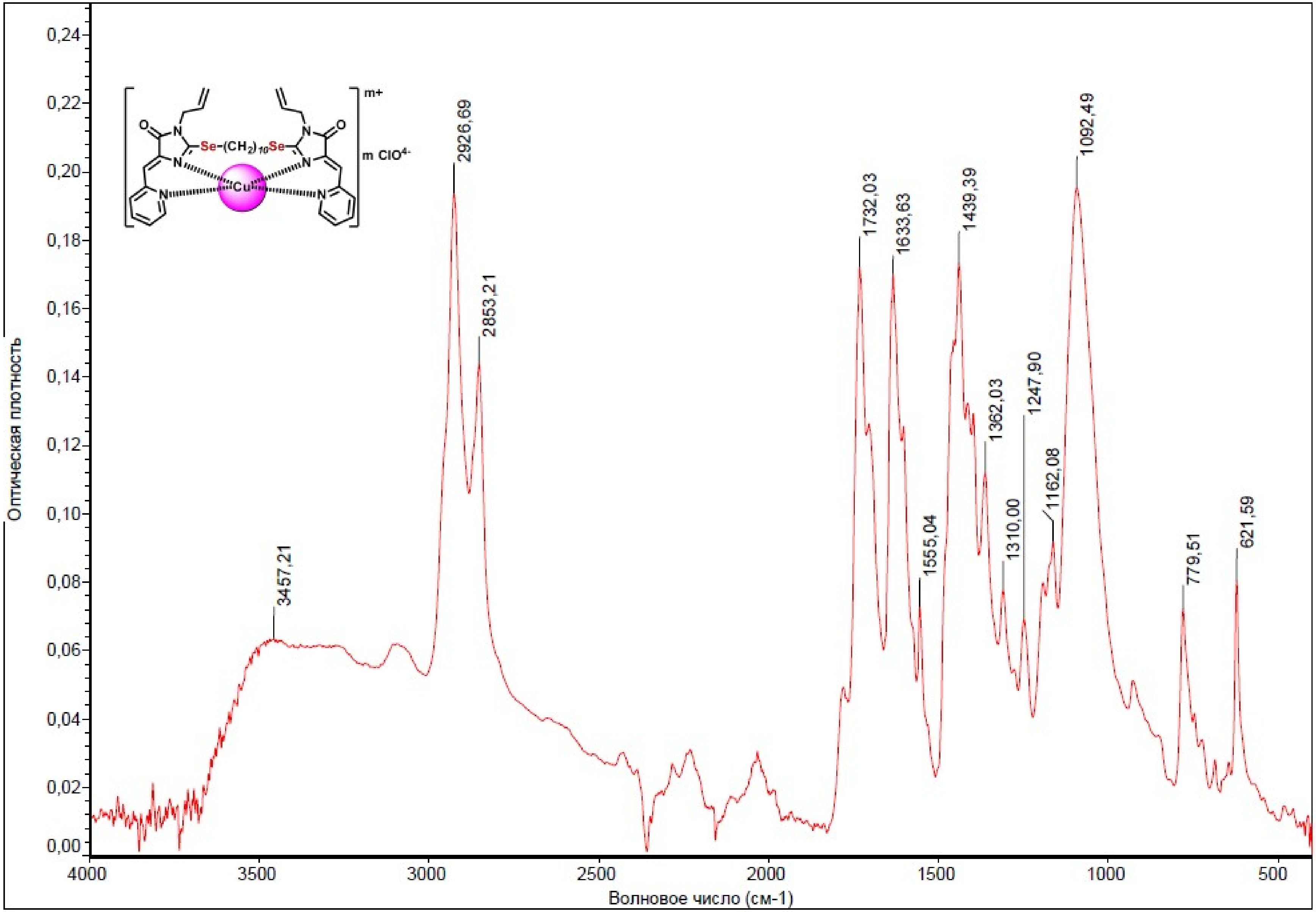

Voltammograms

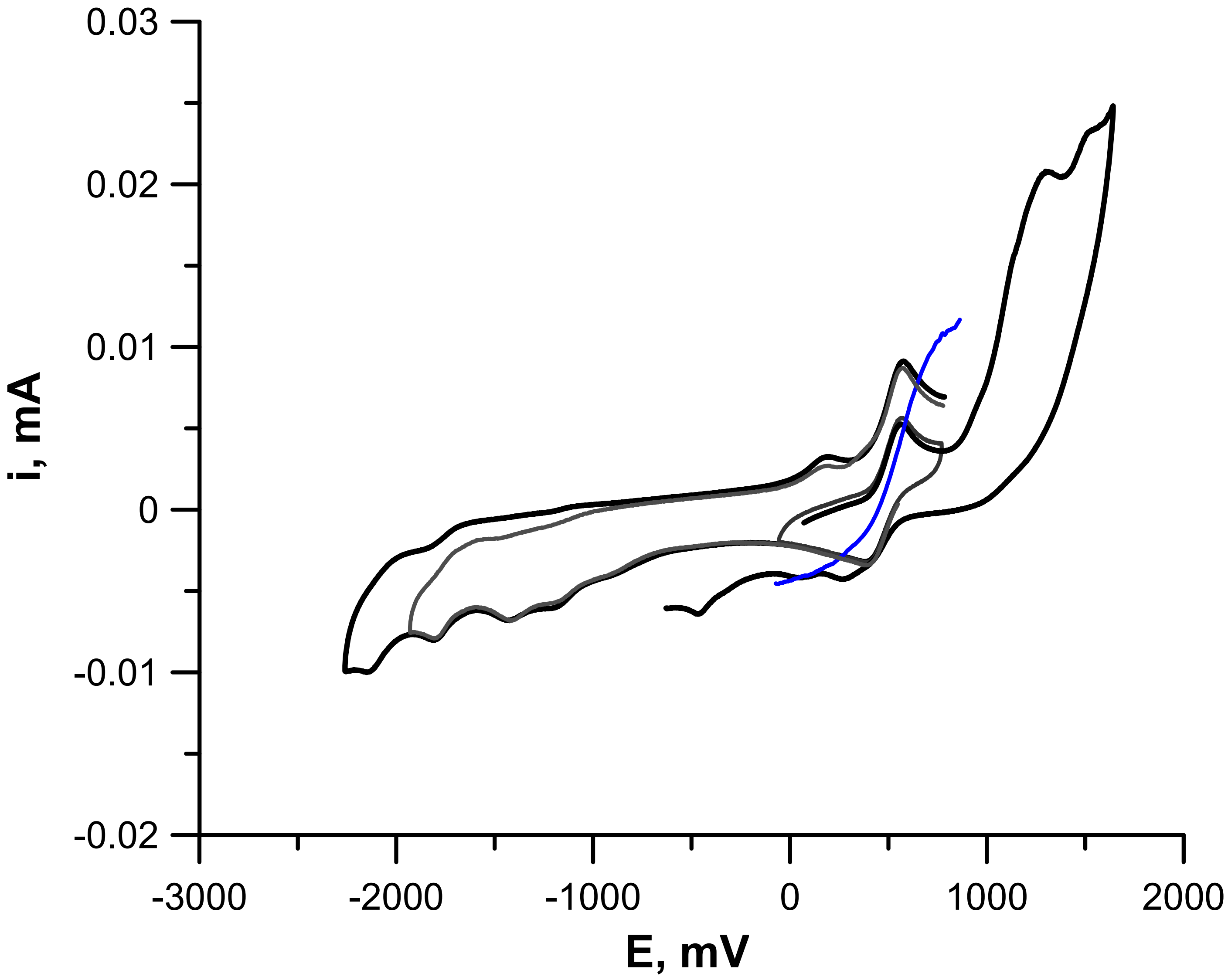

Figure 1S. Cyclic voltammograms (black) and RDE curves (color) of compound **5a**. DMF, 5·10^{-4}M, 0.1M Bu_4NClO_4.

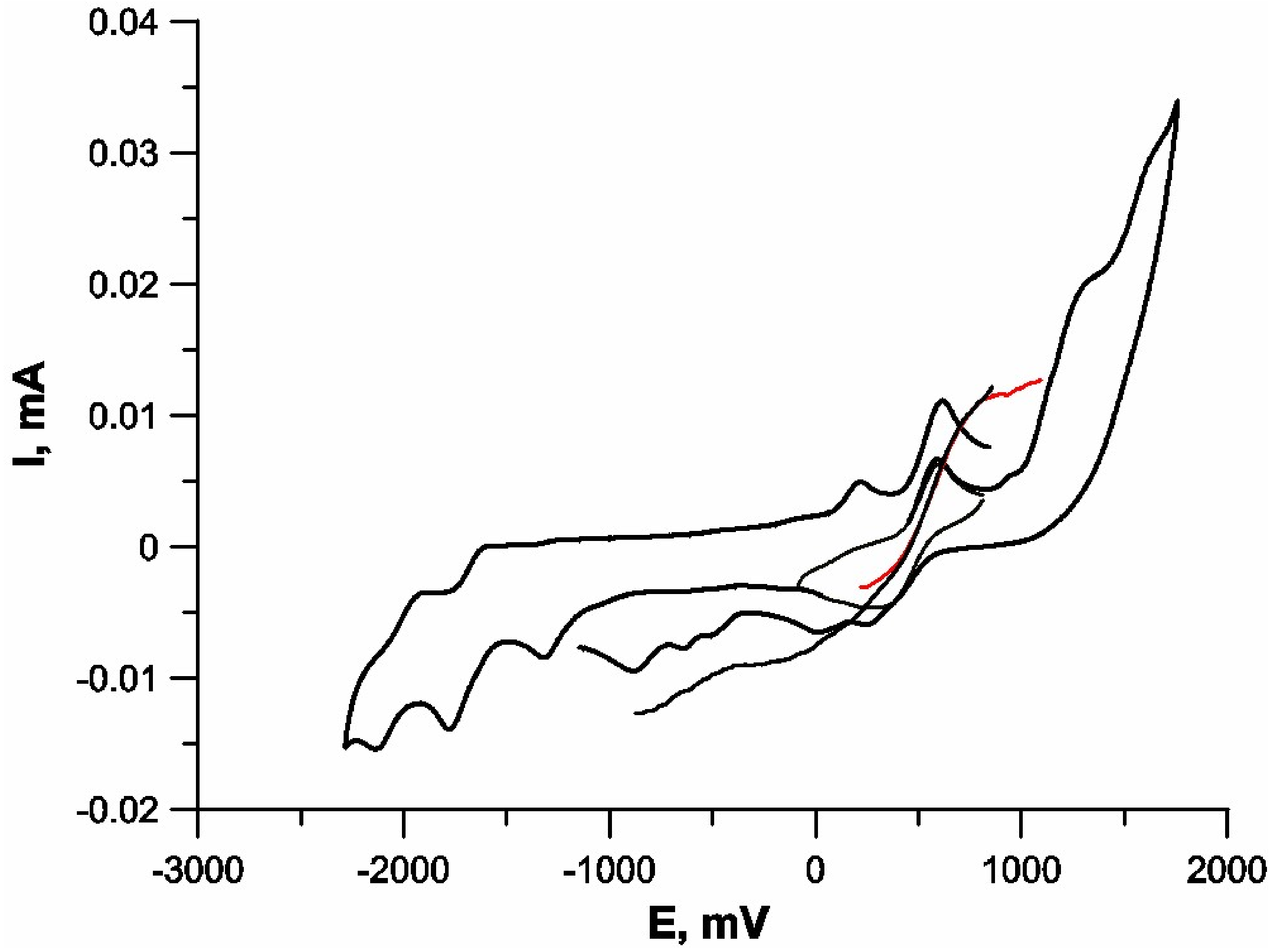

Figure 2S. Cyclic voltammograms (black) and RDE curves (color) of compound **5b**. DMF, 5·10^{-4}M, 0.1M Bu_4NClO_4.

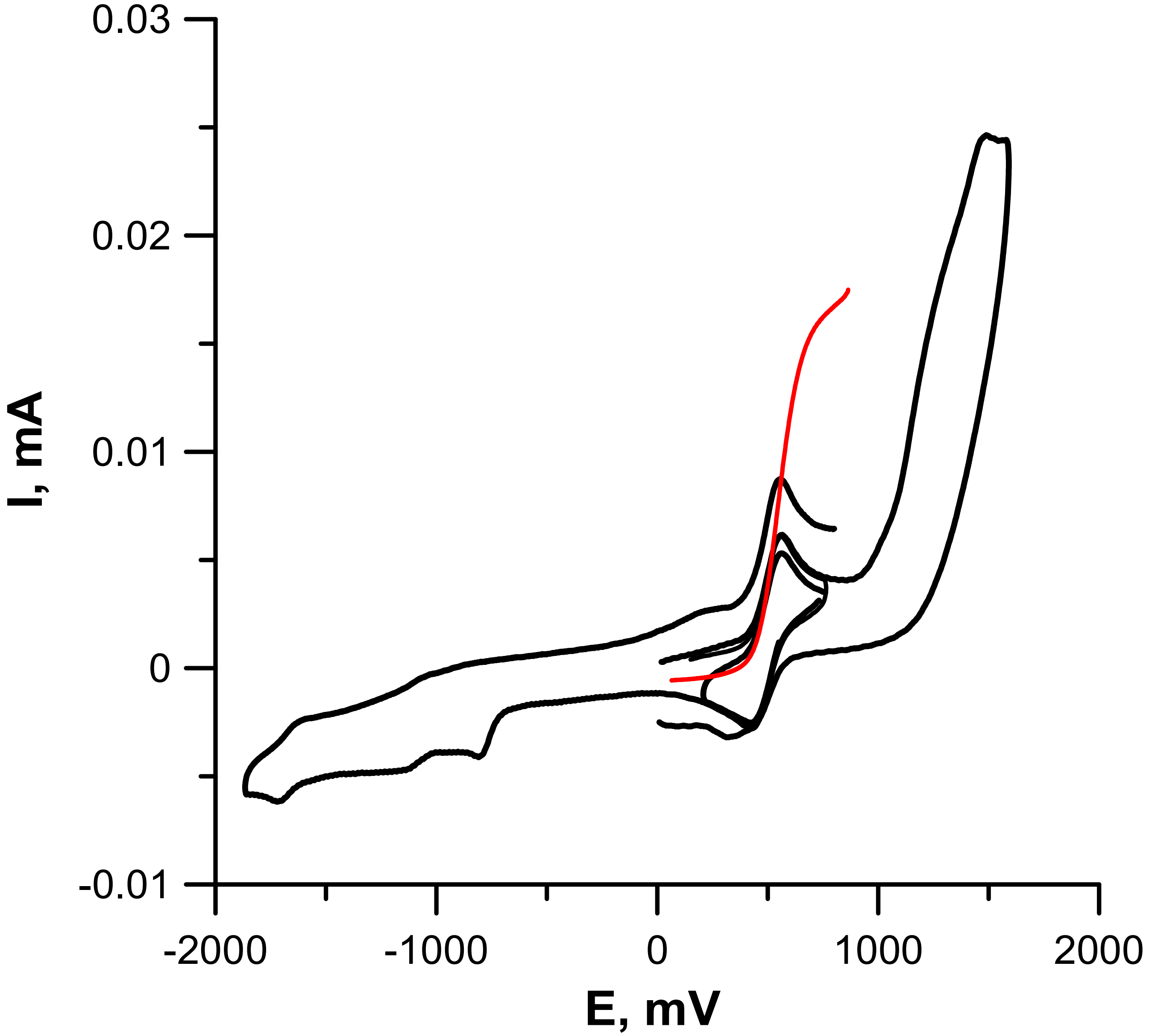

Figure 3S. Cyclic voltammograms (black) and RDE curves (color) of compound **5c**. DMF, 5·10^{-4}M, 0.1M Bu_4NClO_4.

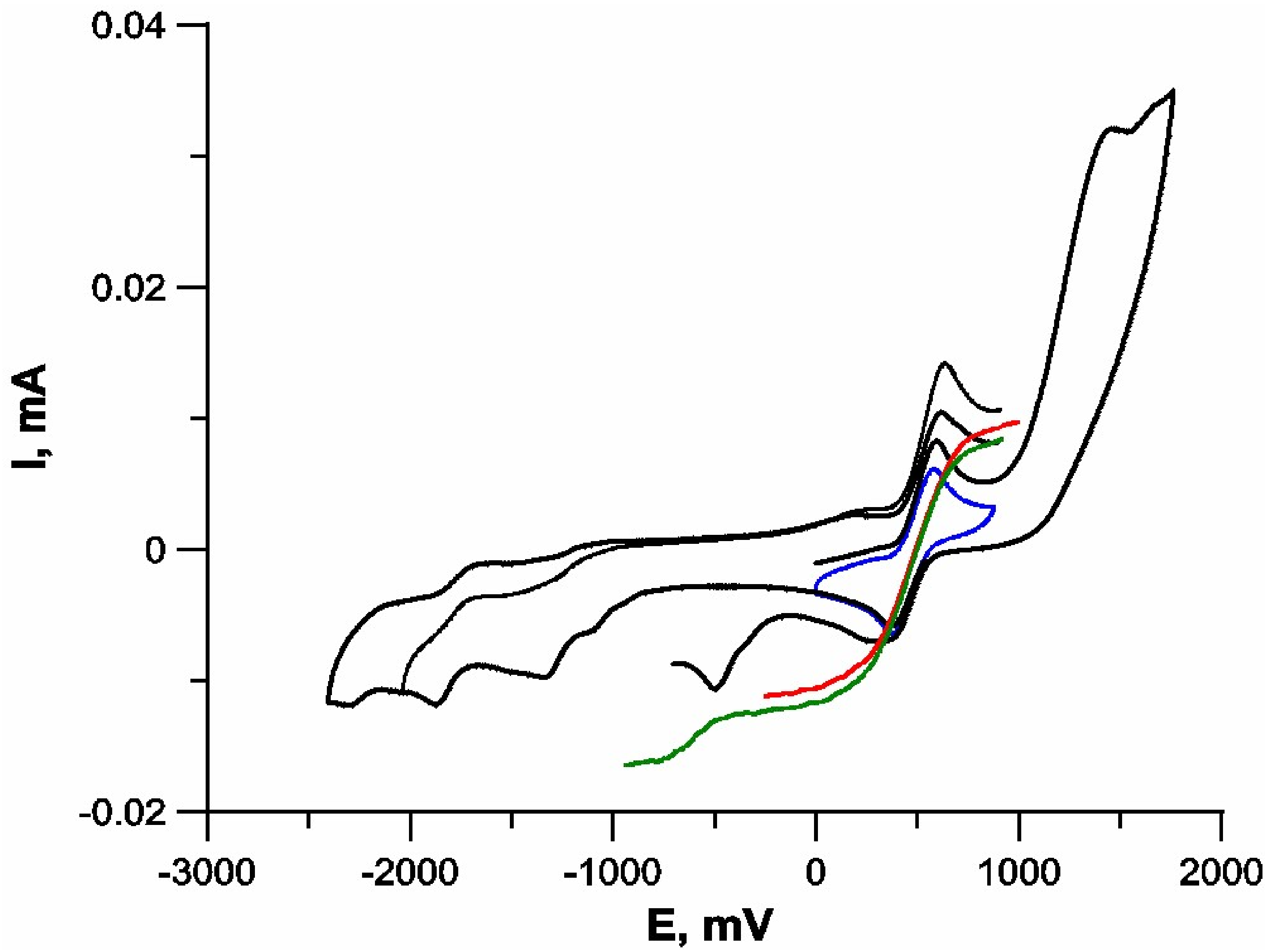

Figure 4S. Cyclic voltammograms (black) and RDE curves (color) of compound **5d**. DMF, 5·10^{-4}M, 0.1M Bu_4NClO_4.

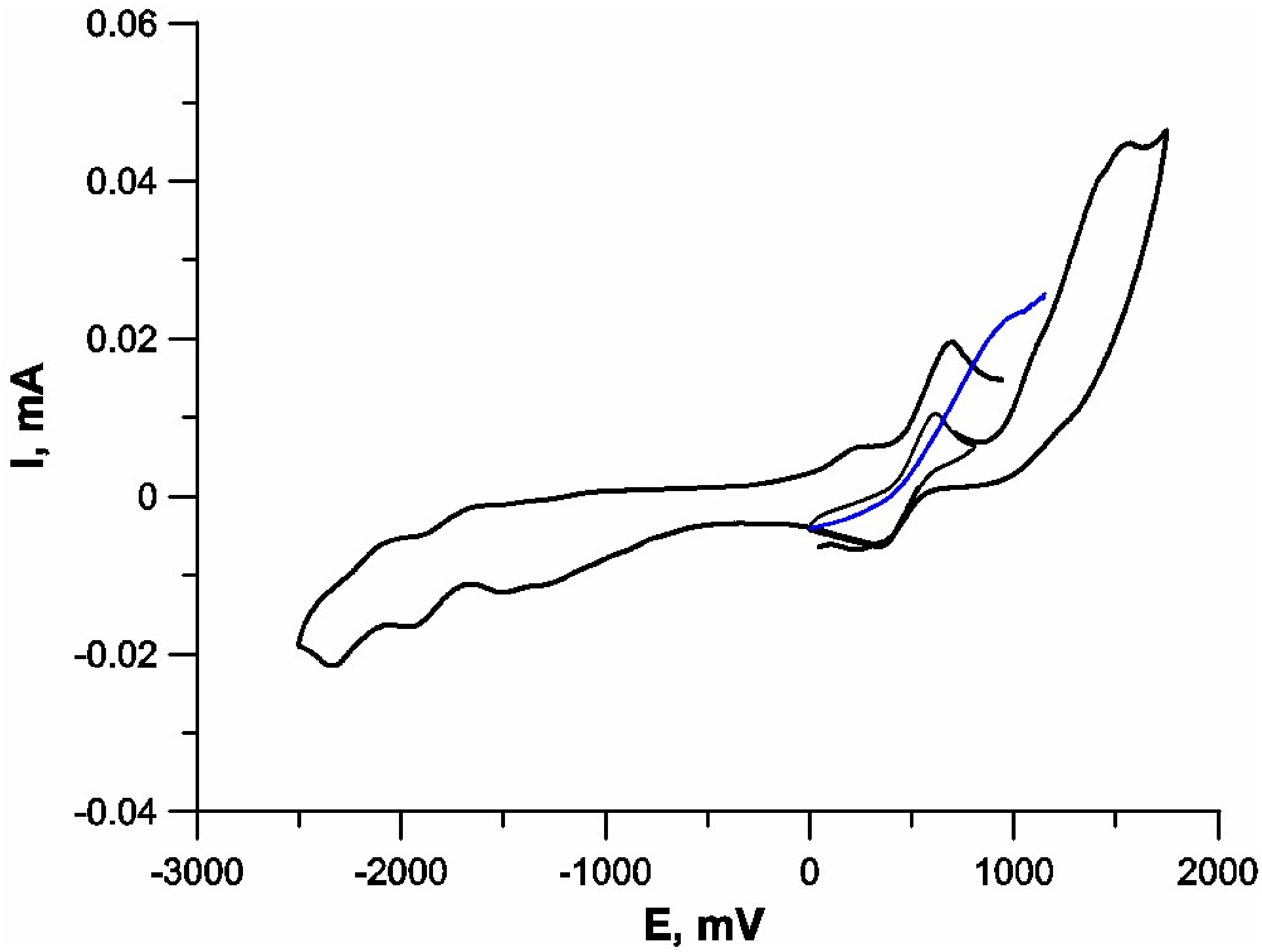

Figure 5S. Cyclic voltammograms (black) and RDE curves (color) of compound **5e**. DMF, $5 \cdot 10^{-4}$M, 0.1M Bu_4NClO_4.

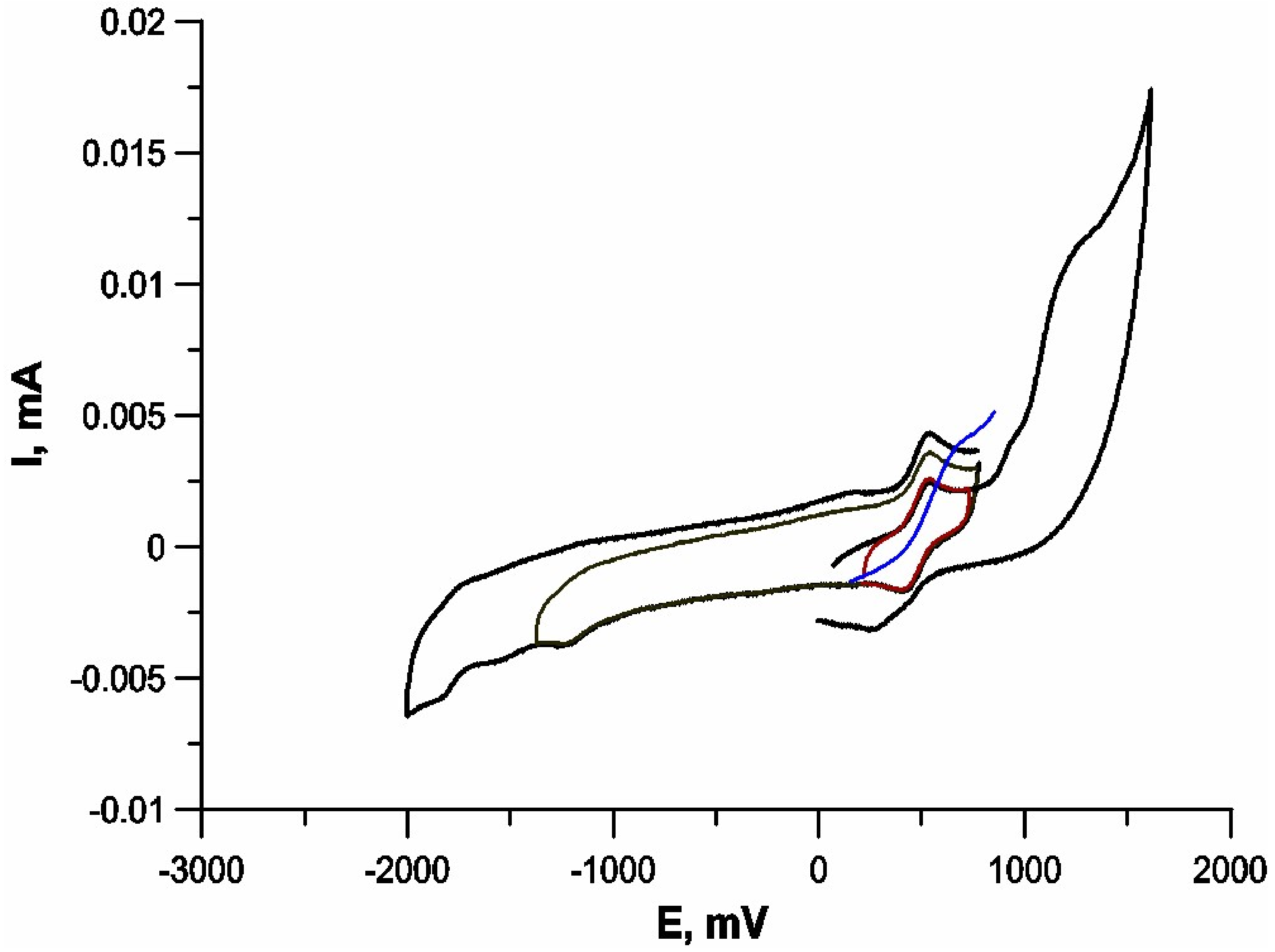

Figure 6S. Cyclic voltammograms (black) and RDE curves (color) of compound **5f**. DMF, $5 \cdot 10^{-4}$M, 0.1M Bu_4NClO_4.

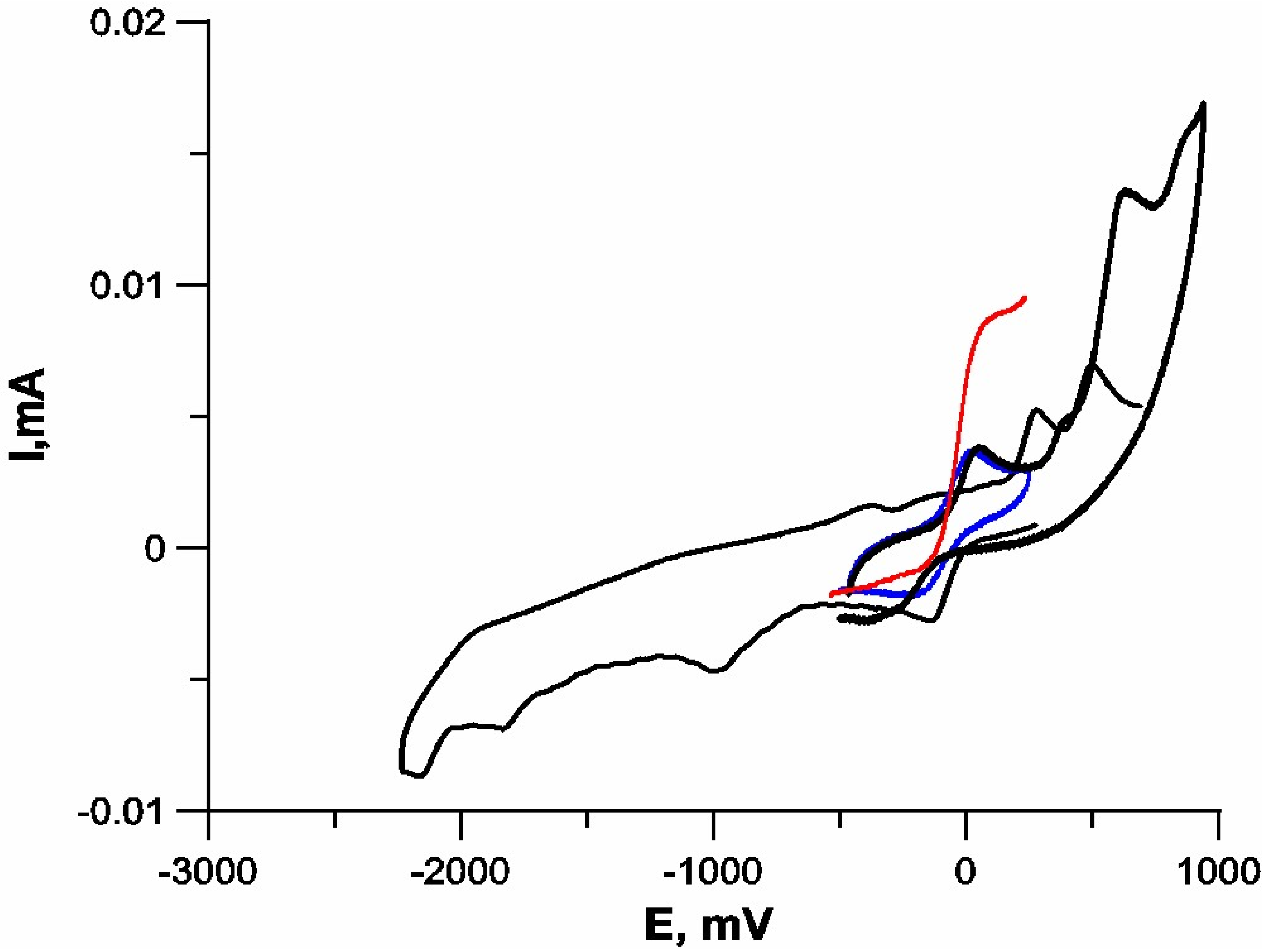

Figure 7S. Cyclic voltammograms (black) and RDE curves (color) of compound **5g**. DMF, 5·10^{-4}M, 0.1M Bu_4NClO_4.

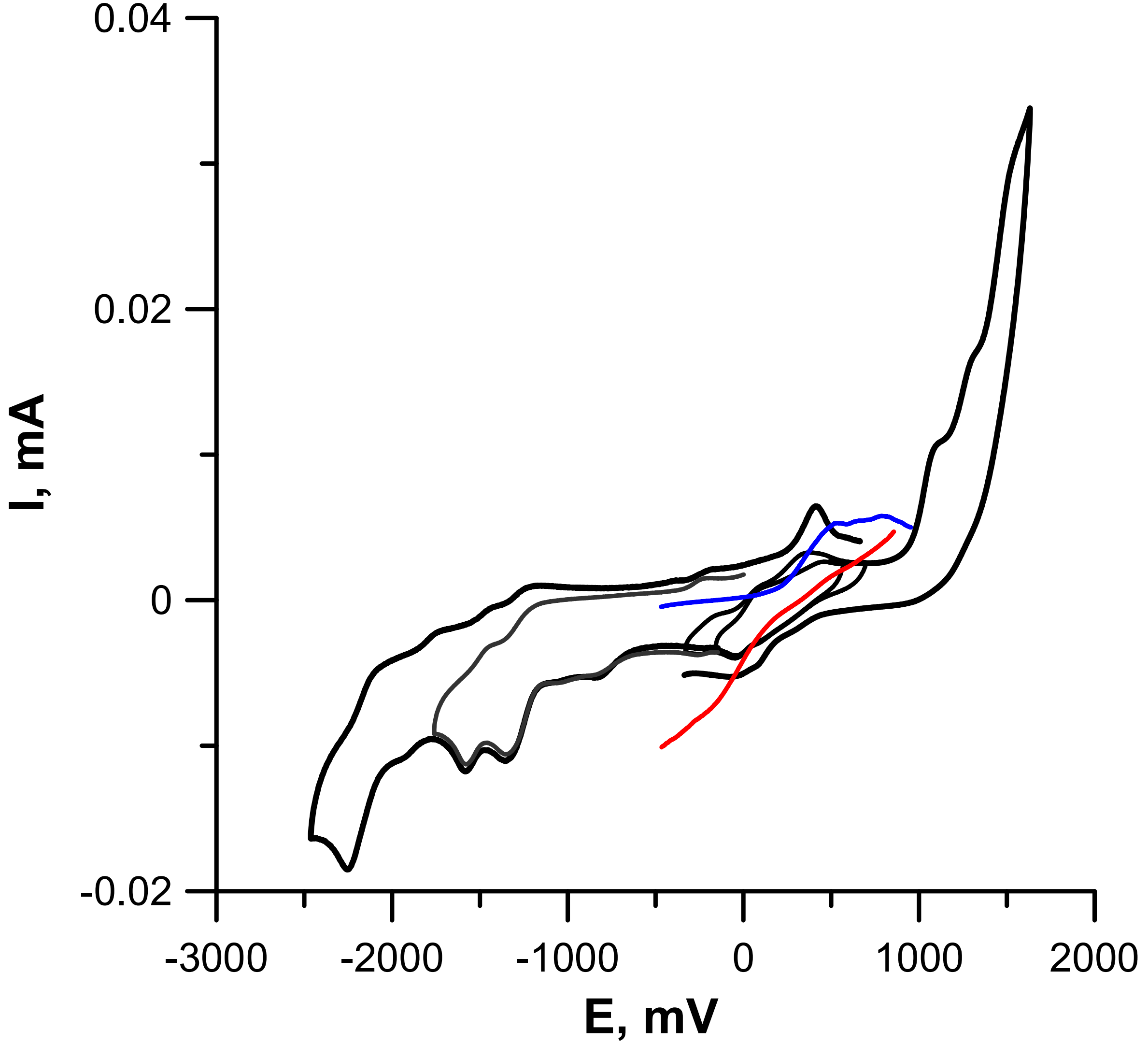

Figure 8S. Cyclic voltammograms (black) and RDE curves (color) of compound **5h**. DMF, $5{\cdot}10^{-4}$M, 0.1M Bu_4NClO_4.

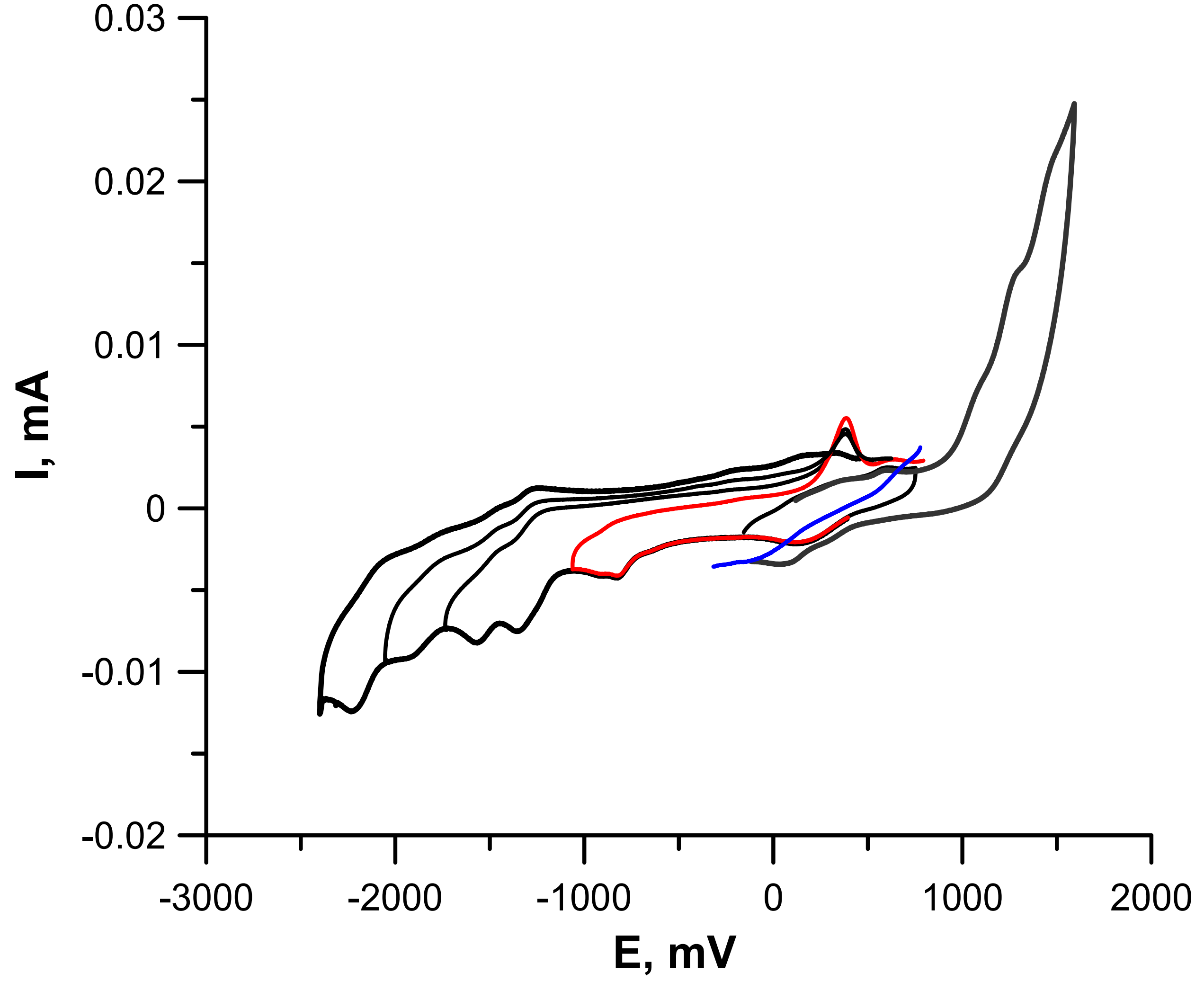

Figure 9S. Cyclic voltammograms (black) and RDE curves (color) of compound **5i**. DMF, 5·10^{-4}M, 0.1M Bu_4NClO_4.

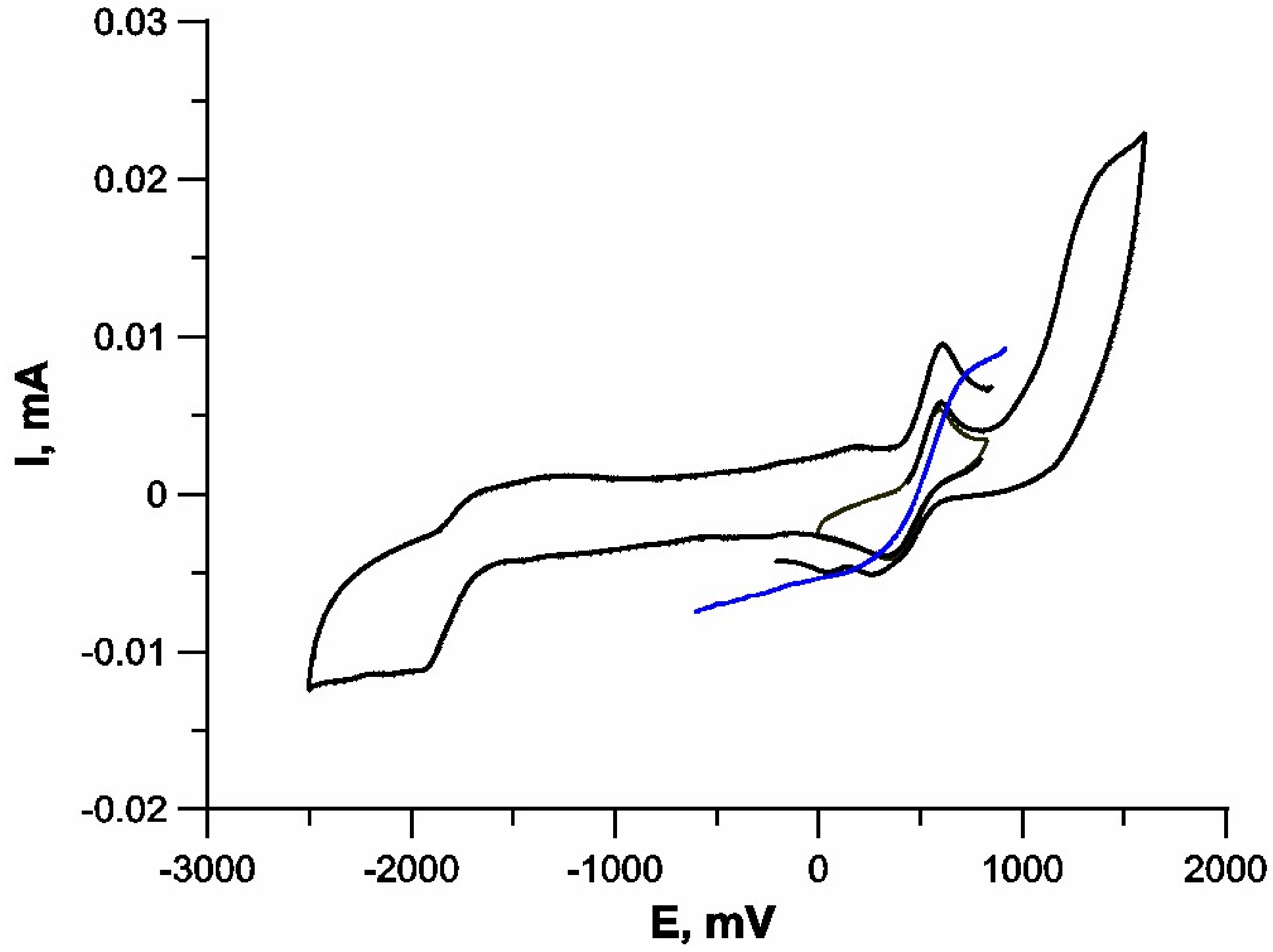

Figure 10S. Cyclic voltammograms (black) and RDE curves (color) of compound **5j**. DMF, $5 \cdot 10^{-4}$M, 0.1M Bu_4NClO_4.

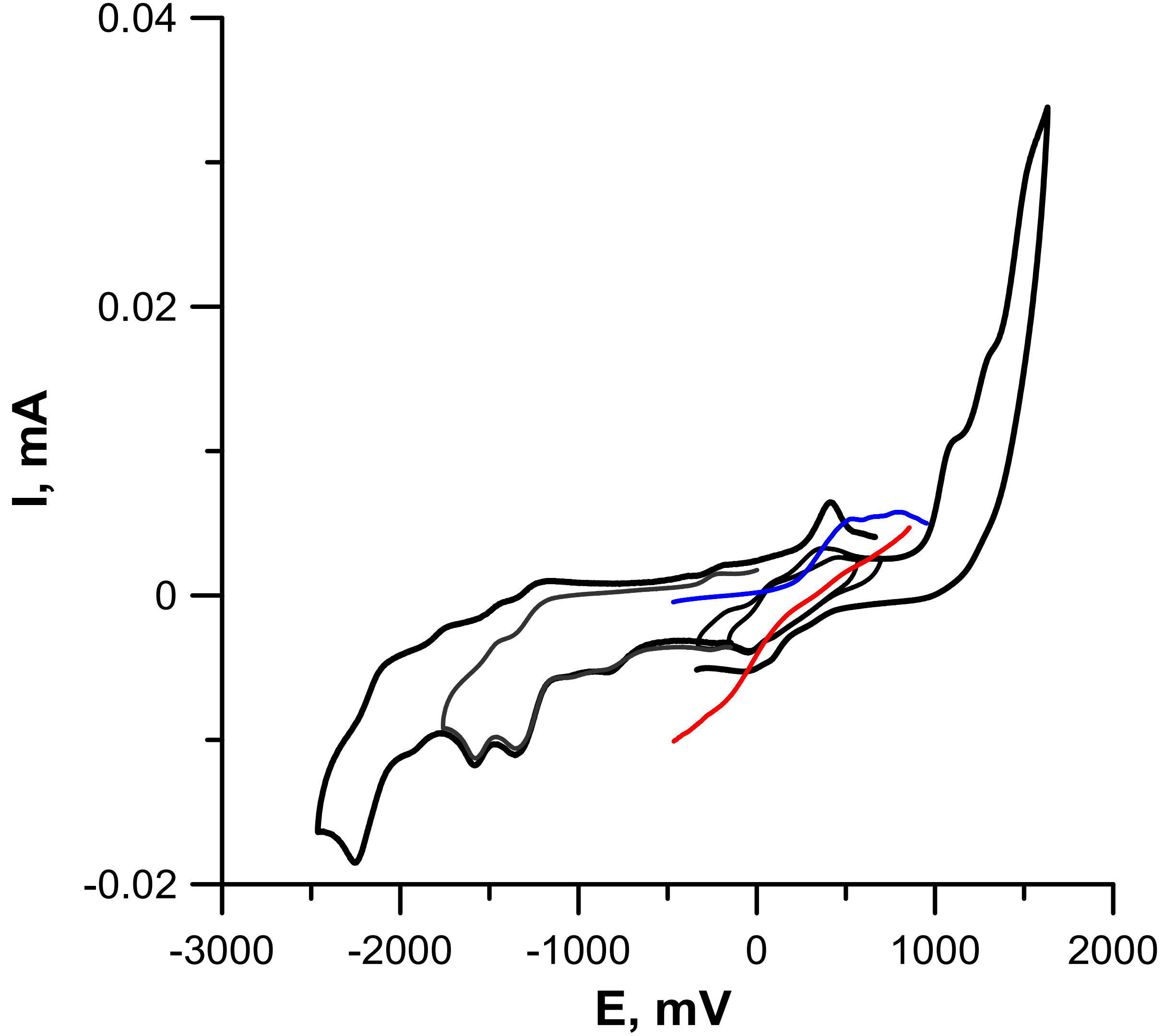

Figure 11S. Cyclic voltammograms (black) and RDE curves (color) of compound **5k**. DMF, $5{\cdot}10^{-4}$M, 0.1M Bu_4NClO_4.

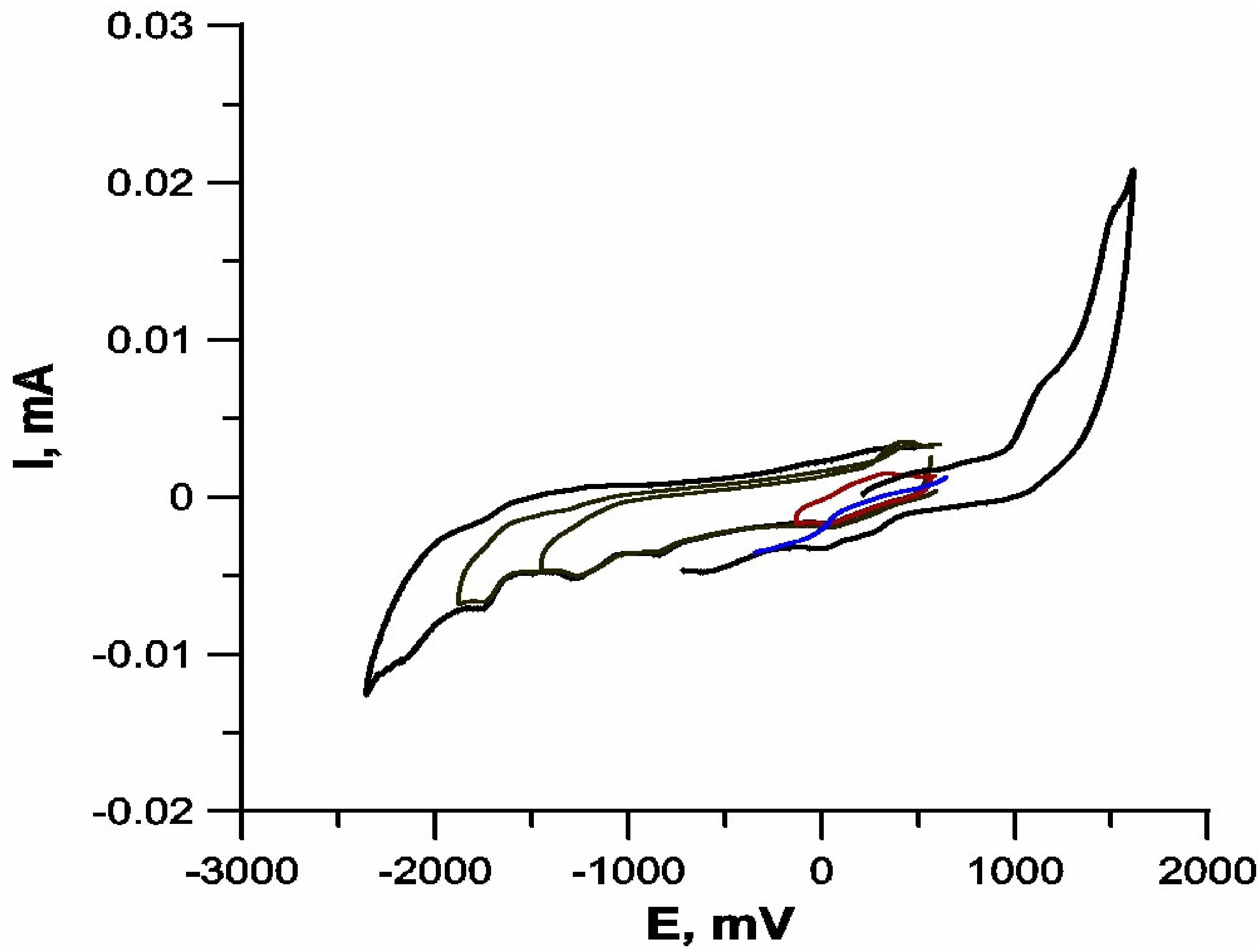

Figure 12S. Cyclic voltammograms (black) and RDE curves (color) of compound **6a**. DMF, $5 \cdot 10^{-4}$M, 0.1M Bu_4NClO_4.

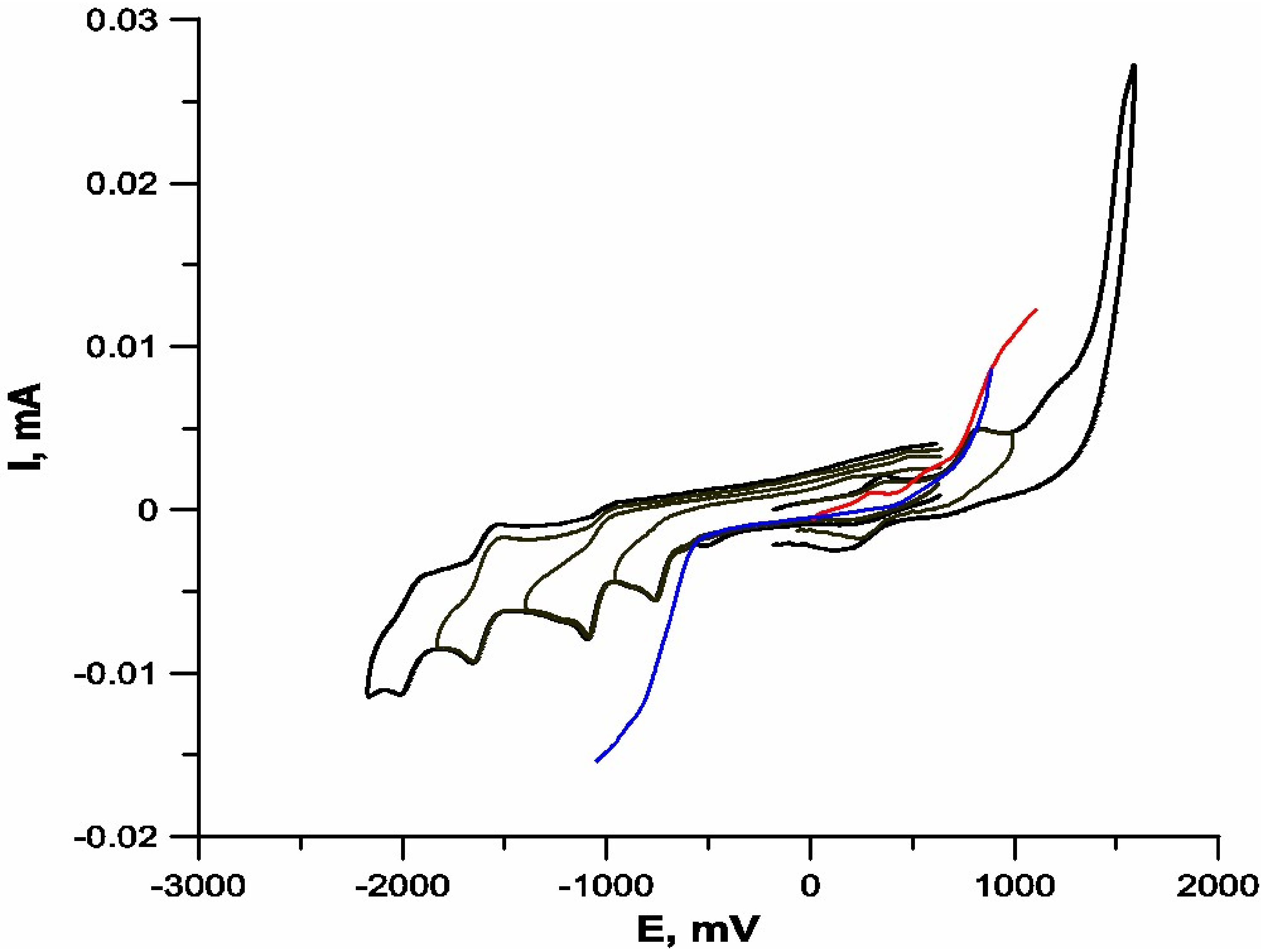

Figure 13S. Cyclic voltammograms (black) and RDE curves (color) of compound **6b**. DMF, $5 \cdot 10^{-4}$M, 0.1M Bu_4NClO_4.

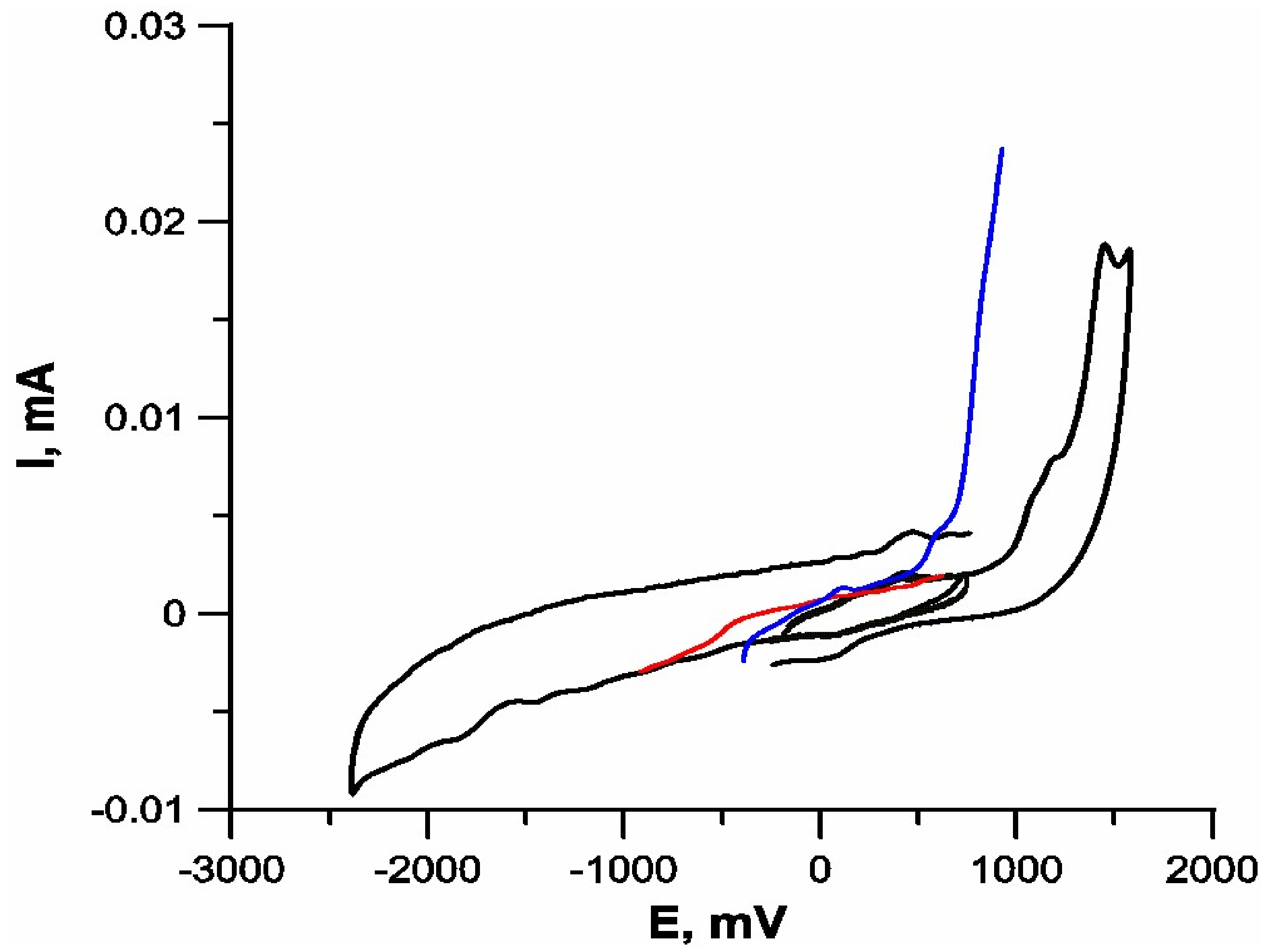

Figure 14S. Cyclic voltammograms (black) and RDE curves (color) of compound **6c**. DMF, $5 \cdot 10^{-4}$M, 0.1M Bu_4NClO_4.

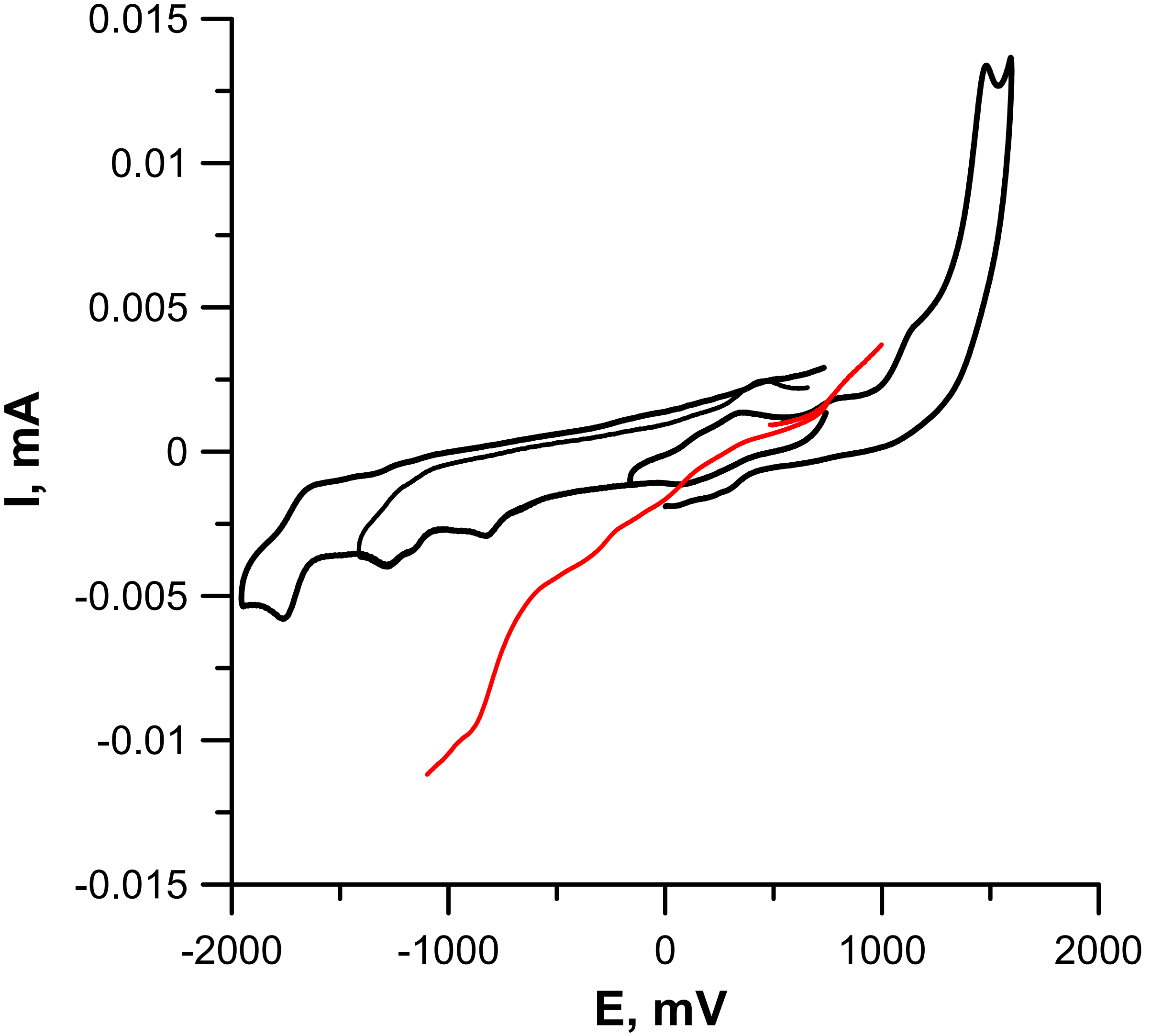

Figure 15S. Cyclic voltammograms (black) and RDE curves (color) of compound **6d**. DMF, 5·10^{-4}M, 0.1M Bu_4NClO_4.

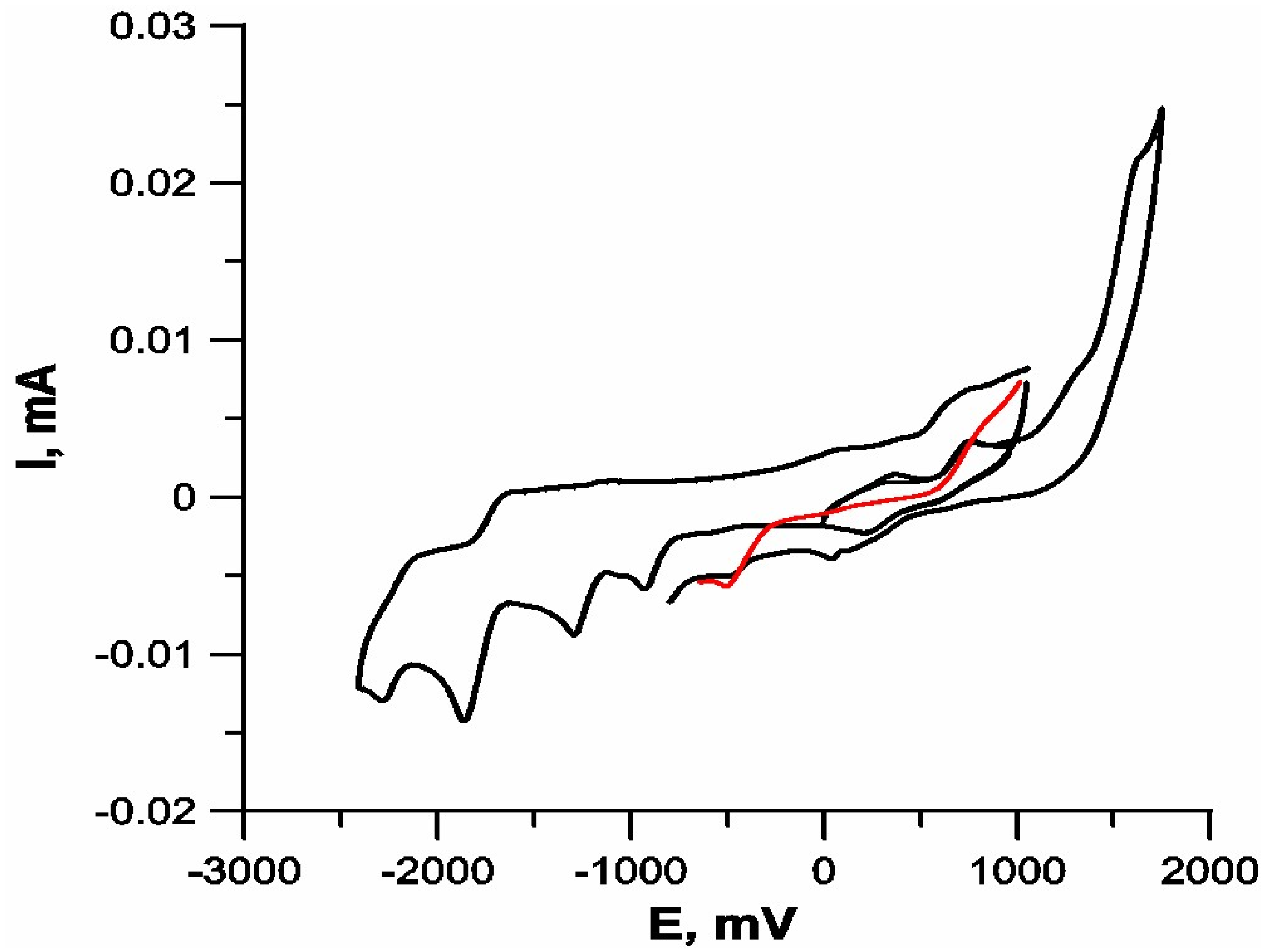

Figure 16S. Cyclic voltammograms (black) and RDE curves (color) of compound **6e**. DMF, $5 \cdot 10^{-4}$M, 0.1M Bu_4NClO_4.

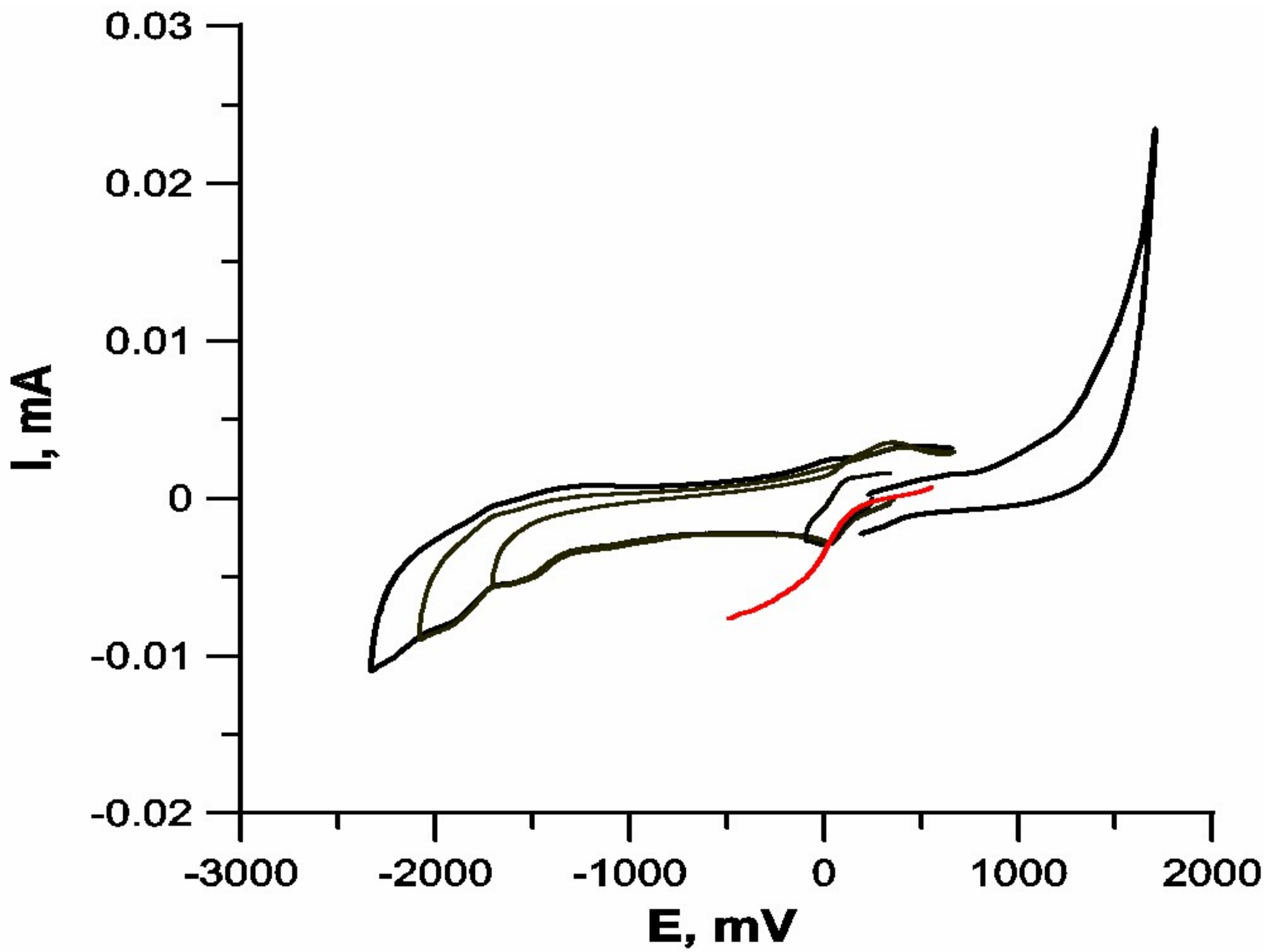

Figure 17S. Cyclic voltammograms (black) and RDE curves (color) of compound **6f**. DMF, $5{\cdot}10^{-4}$M, 0.1M Bu_4NClO_4.

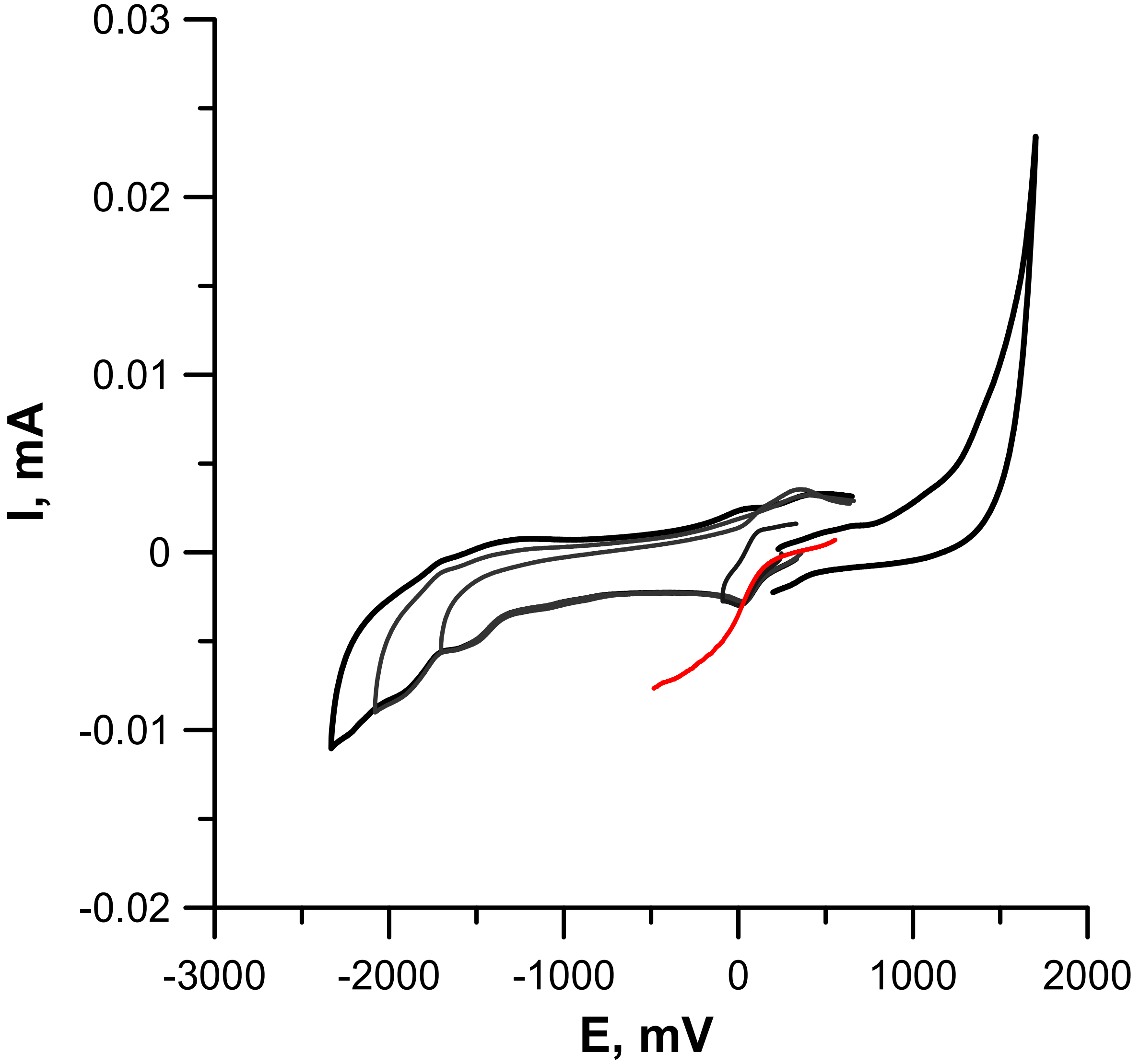

Figure 18S. Cyclic voltammograms (black) and RDE curves (color) of compound **6g**. DMF, 5·10^{-4}M, 0.1M Bu_4NClO_4.

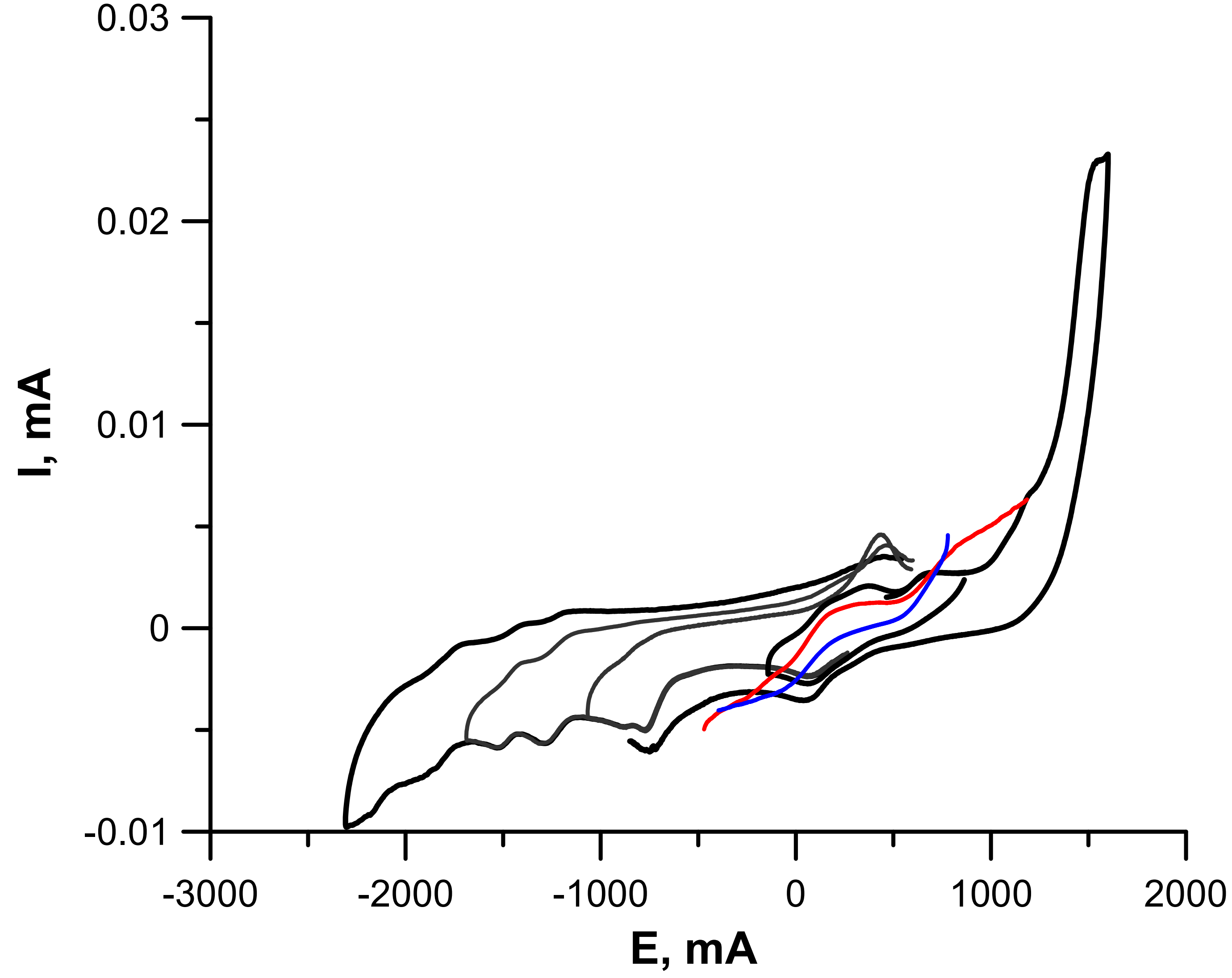

Figure 19S. Cyclic voltammograms (black) and RDE curves (color) of compound **6h**. DMF, 5·10^{-4}M, 0.1M Bu_4NClO_4.

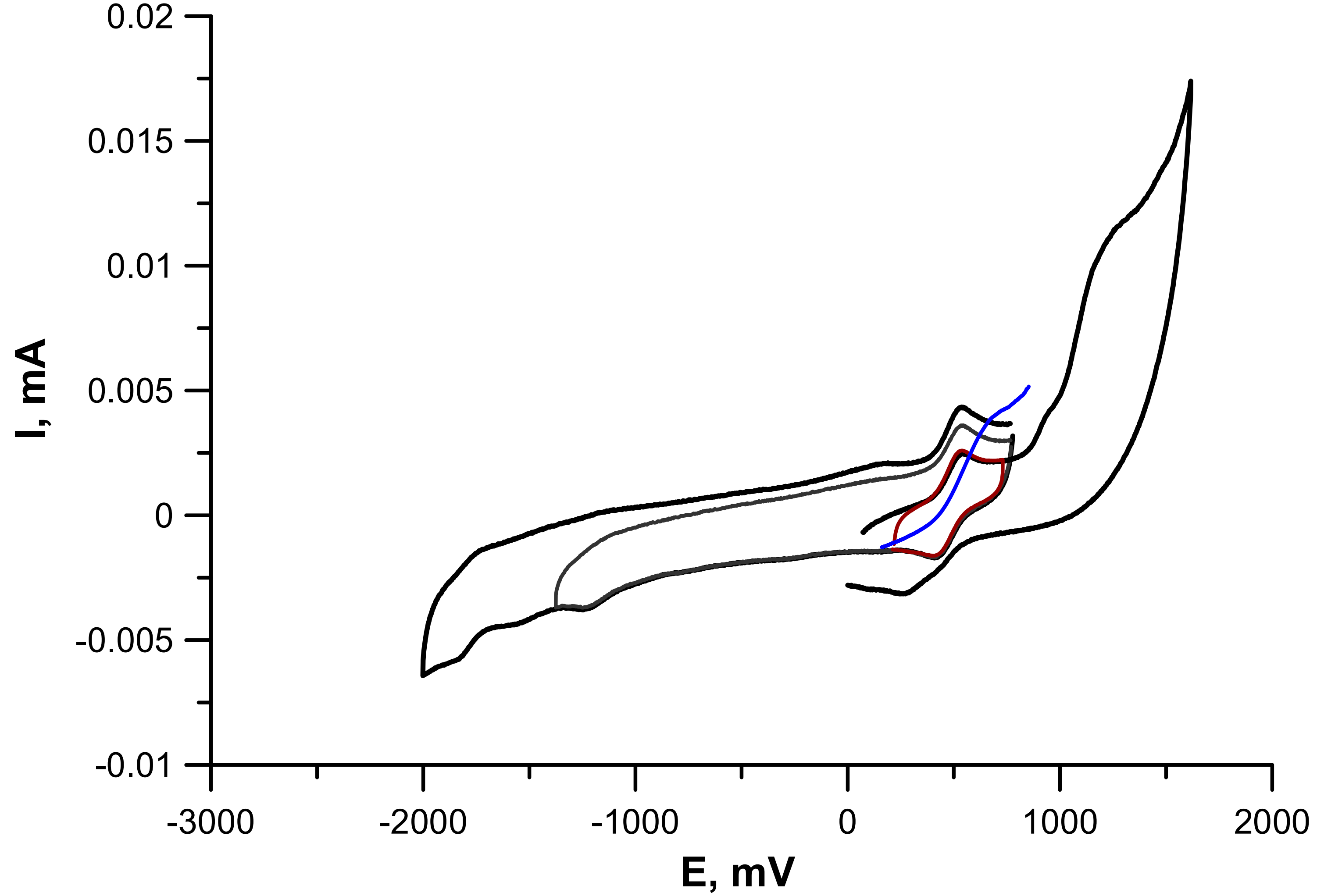

Figure 20S. Cyclic voltammograms (black) and RDE curves (color) of compound **6i**. DMF, $5{\cdot}10^{-4}$M, 0.1M Bu_4NClO_4.

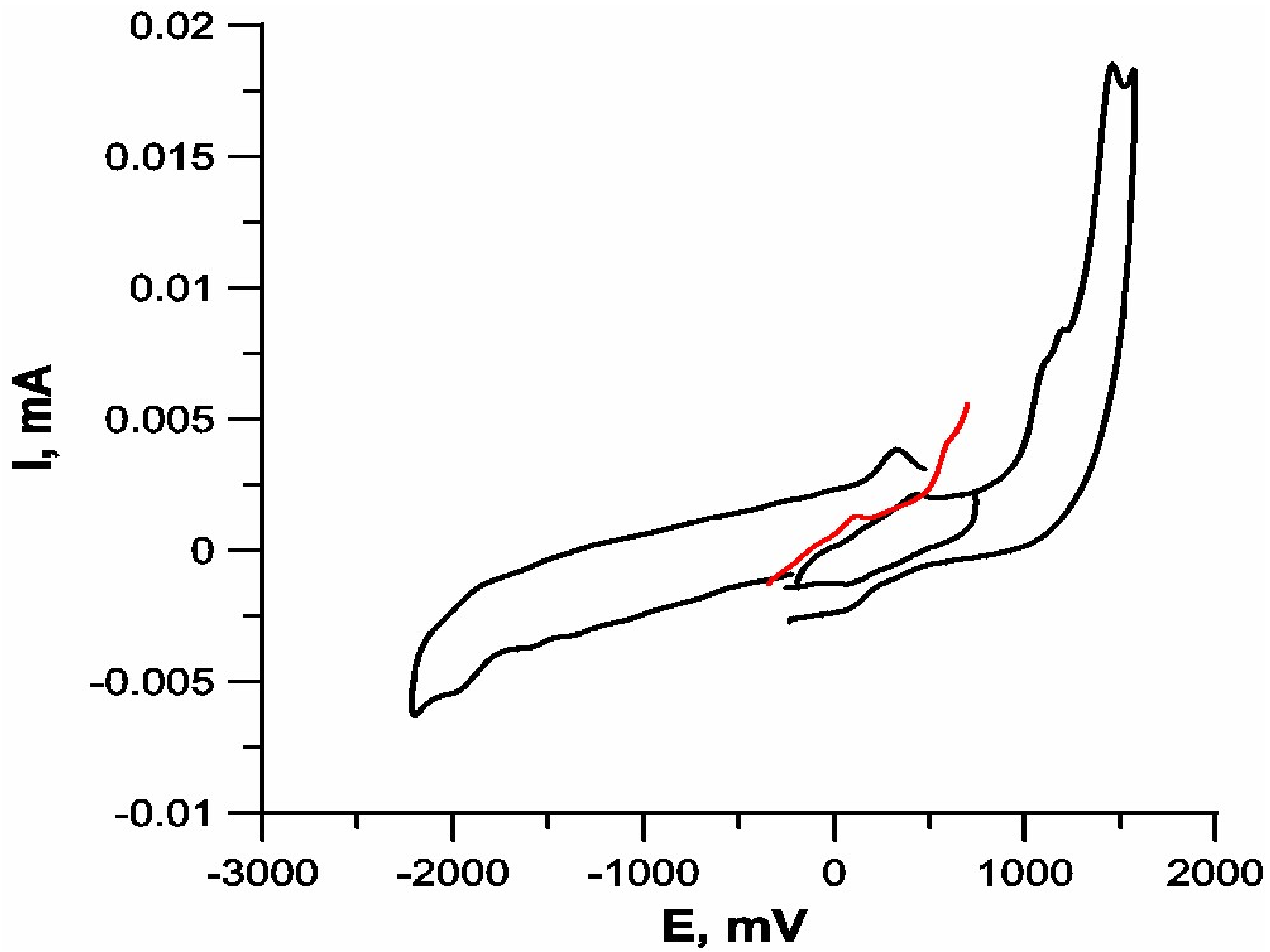

Figure 21S. Cyclic voltammograms (black) and RDE curves (color) of compound **6j**. DMF, $5\cdot10^{-4}$M, 0.1M Bu_4NClO_4.

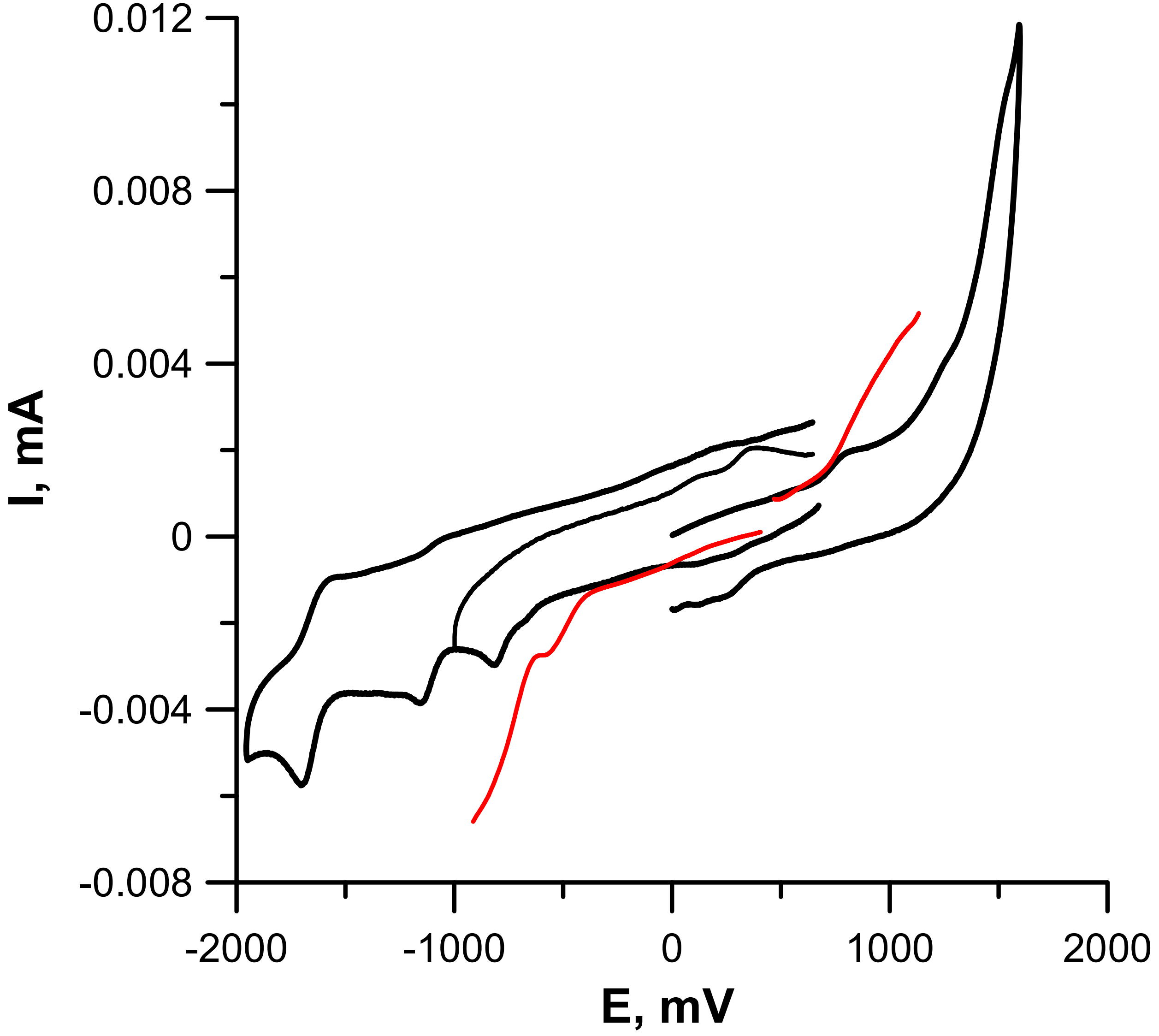

Figure 22S. Cyclic voltammograms (black) and RDE curves (color) of compound **6k**. DMF, $5 \cdot 10^{-4}$M, 0.1M Bu_4NClO_4.

Table 1S. Electrochemical reduction (E^{Red}) and oxidation (E^{Ox}) potentials of ligands and complexes on a GC electrode measured relative to Ag/AgCl/KCl(sat.). DMF, $5 \cdot 10^{-4}$M, 0.1M Bu_4NClO_4. E_p is the peak potential. Potential scan rate is 100 mV s^{-1}.

Compound	E_p^{Red} (V)	$E_p^{Ox.}$ (V)	Oxidation state of copper in the main product	Oxidation state of copper in the minor product
**5a**	0.38/0.55; -0.88; -1.17; -1.42; 1.80; -2.14	0.55/0.38; 1.13; 1.29; 1.5	$Cu^{+1}Cu^{+2}$	-
**6a**	0.35/0.58; -0.79; -1.10; -1.68	1.29; 1.61	Cu^{+2}	-
**5b**	-1.31; -1.77/-1.62; -2.13/-1.93	0.55/0.38; 1.37; 1.44	$Cu^{+1}Cu^{+1}$	$Cu^{+1}Cu^{+2}$ or/and $Cu^{+2}Cu^{+2}$
**6b**	-0.75; -1.09; -1.65; -2.00	0.81/0.34; 1.18; 1.55	Cu^{+1}	-
**5c**	-1.08; -1.59/-1.50; -2.04	0.58/0.29; 1.14; 1.57	$Cu^{+1}Cu^{+1}$	$Cu^{+1}Cu^{+2}$ or/and $Cu^{+2}Cu^{+2}$
**6c**	-1.22; -1.48; -1.78; -2.19	0.43/0.09	Cu^{+1}	-
**5e**	-1.13; -1.46; -1.90; -1.26	0.60/0.35; 1.41; 1.54	$Cu^{+1}Cu^{+1}$	$Cu^{+1}Cu^{+2}$ or/and $Cu^{+2}Cu^{+2}$
**6d**	0.35/0.60; -0.75; -1.43; -1.94/-1.68	-1.09; 1.41; 1.54	Cu^{+2}	-
**5d**	0.38/0.58; -0.94; -1.87/-1.69; -2.28	0.58/0.38; 1.04; 1.46	$Cu^{+1}Cu^{+2}$	$Cu^{+2}Cu^{+2}$
**6e**	-0.92; -1.29; -1.87/-1.7; -2.28/-2.15	0.59/0.34; 1.25; 1.58	Cu^{+1}	-
**5f**	0.53/0.61; -0.99; -1.34; -1.81	0.61/0.53; 0.99; 1.29; 1.44	$Cu^{+1}Cu^{+2}$	-
**6f**	0.07/0.34; -1.16; -1.27; -1.75	1.13	Cu^{+2}	-
**5g**	0.41/0.53; -1.22; -1.53; -1.83	0.53/0.41; 0.93; 1.2	$Cu^{+1}Cu^{+2}$	-
**6g**	0.03/0.11; -1.49; -1.59; -1.91; -2.20	1.41	Cu^{+2}	-
**5h**	0.06/0.38; -1.30; -1.57; -2.23	0.38/0.06; 1.12; 1.34	$Cu^{+1}Cu^{+2}$	-
**6h**	0.05/0.42; -0.76; -1.27; -1.51; -1.88; -2.17	0.78; 1.21	Cu^{+2}	Cu^{+1}
**5i**	0.40/0.55; -1.30; -1.60; -1.87	0.94; 1.20; 1.51	$Cu^{+2}Cu^{+2}$	$Cu^{+1}Cu^{+2}$ or/and $Cu^{+1}Cu^{+1}$
**6i**	0.43/0.50; -1.18; --1.58; -1.89	0.96; 1.20	Cu^{+2}	Cu^{+1}
**5j**	0.48/0.59; -1.92-2.31	0.59/0.48; 1.37	$Cu^{+1}Cu^{+2}$	$Cu^{+2}Cu^{+2}$
**6j**	0.08/0.41; -0.69; -1.09; -1.37; -1.57; -1.98	1.25; 1.52	Cu^{+2}	Cu^{+1}
**5k**	0.42/0.06; -0.75; -1.32; -1.63; -2.24	1.03; 1.25	$Cu^{+2}Cu^{+2}$	$Cu^{+1}Cu^{+2}$
**6k**	0.47/0.32; -0.81; -1.19; -1.69	0.87; 1.32	Cu^{+2}	Cu^{+1}

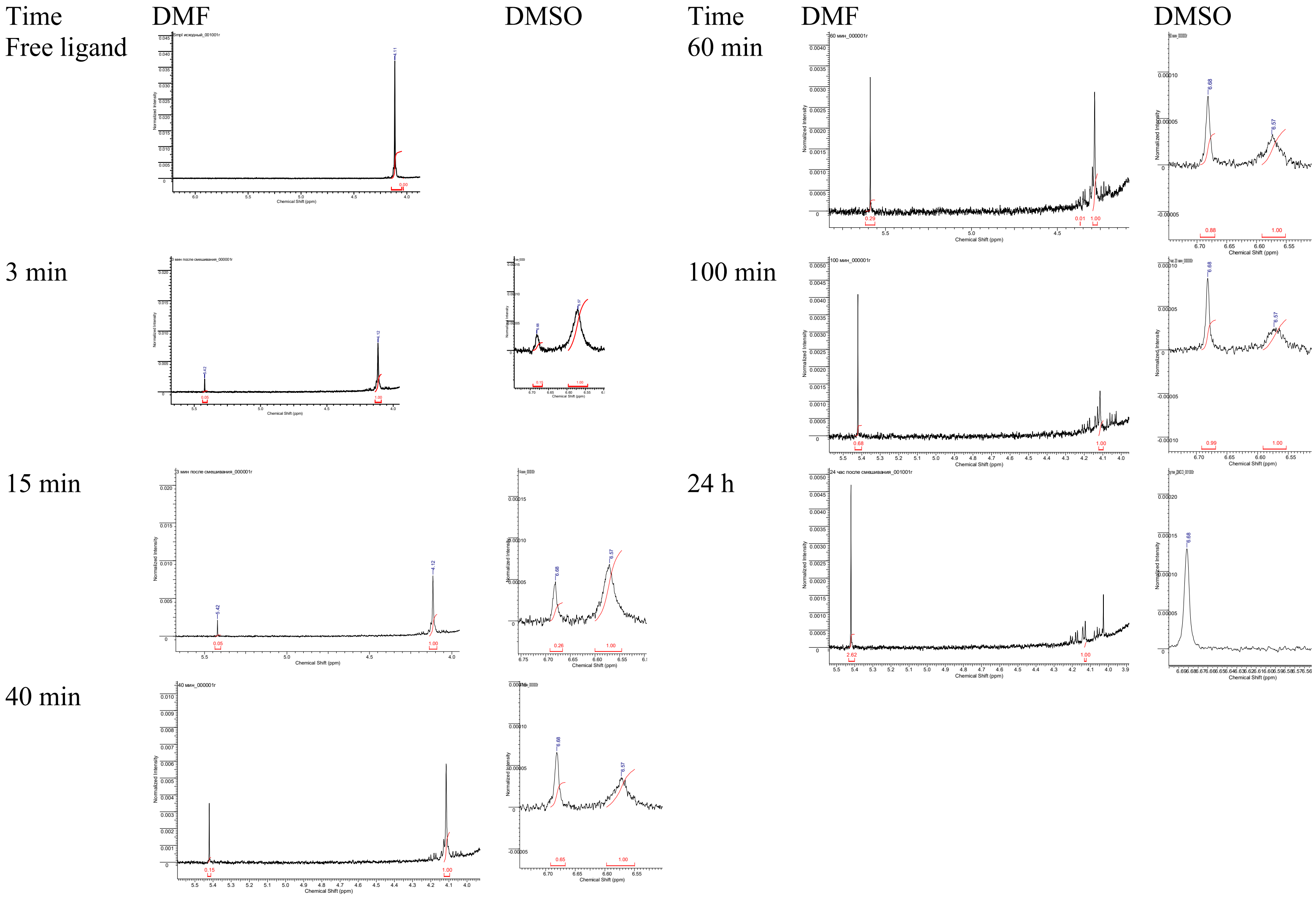

Figure 23S. ^{1}H NMR monitoring of the reaction mixture of ligand **4c** with $CuCl_2 \cdot 2H_2O$ in DMF and DMSO.

AT:

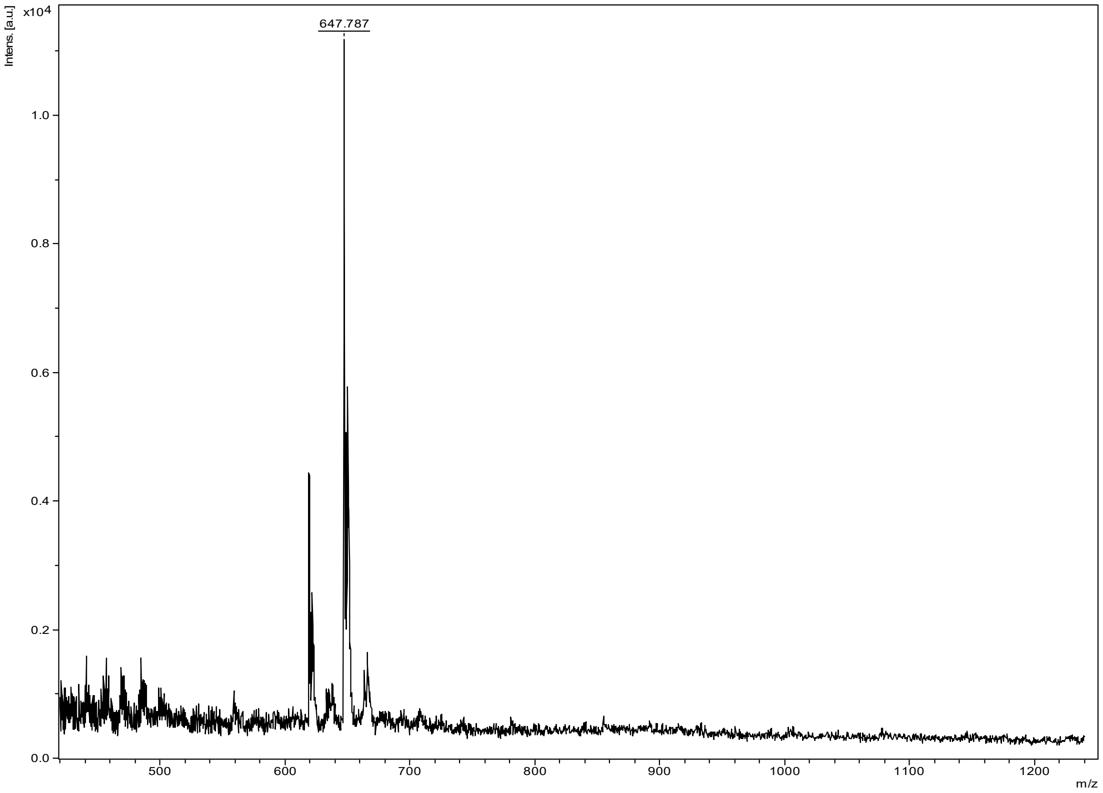

647.787
Intens. [a.u.]
x10^4
1.0
0.8
0.6
0.4
0.2
0.0
500
600
700
800
900
1000
1100
1200
m/z

DHB:

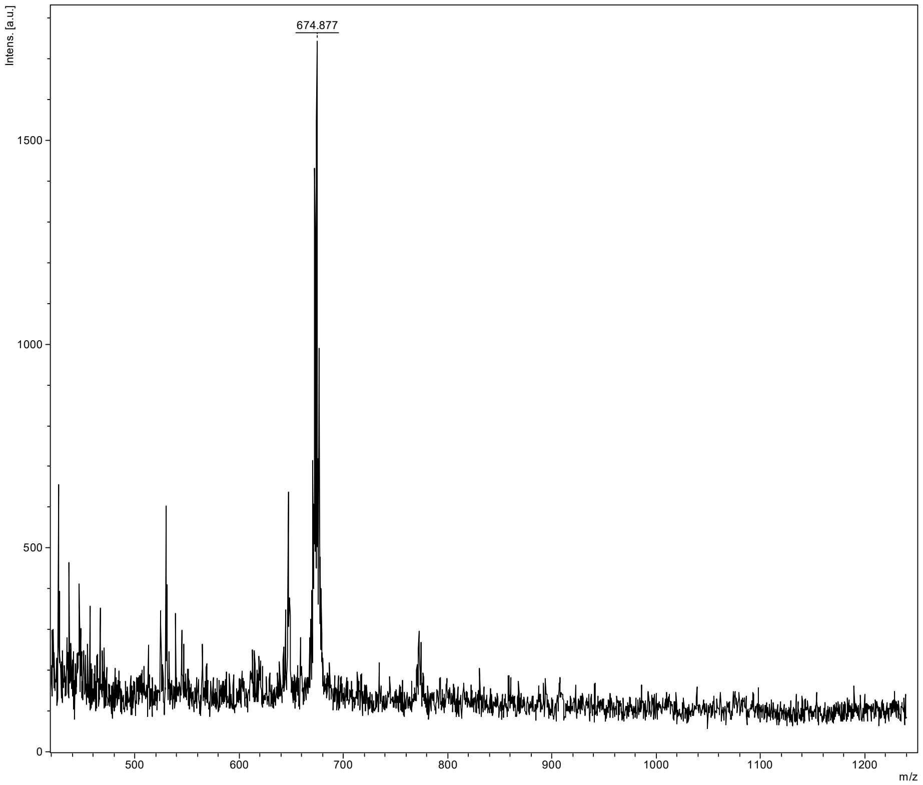

674.877
Intens. [a.u.]
1500
1000
500
0
500
600
700
800
900
1000
1100
1200
m/z

Fluorene:

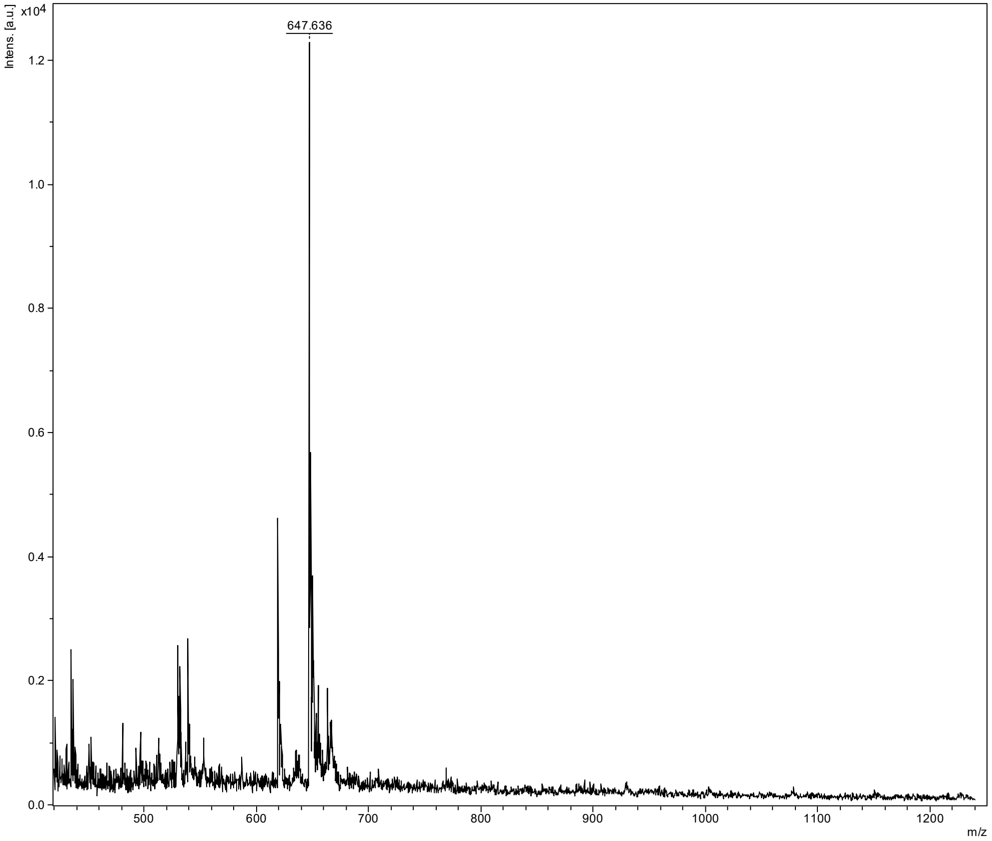

Phen:

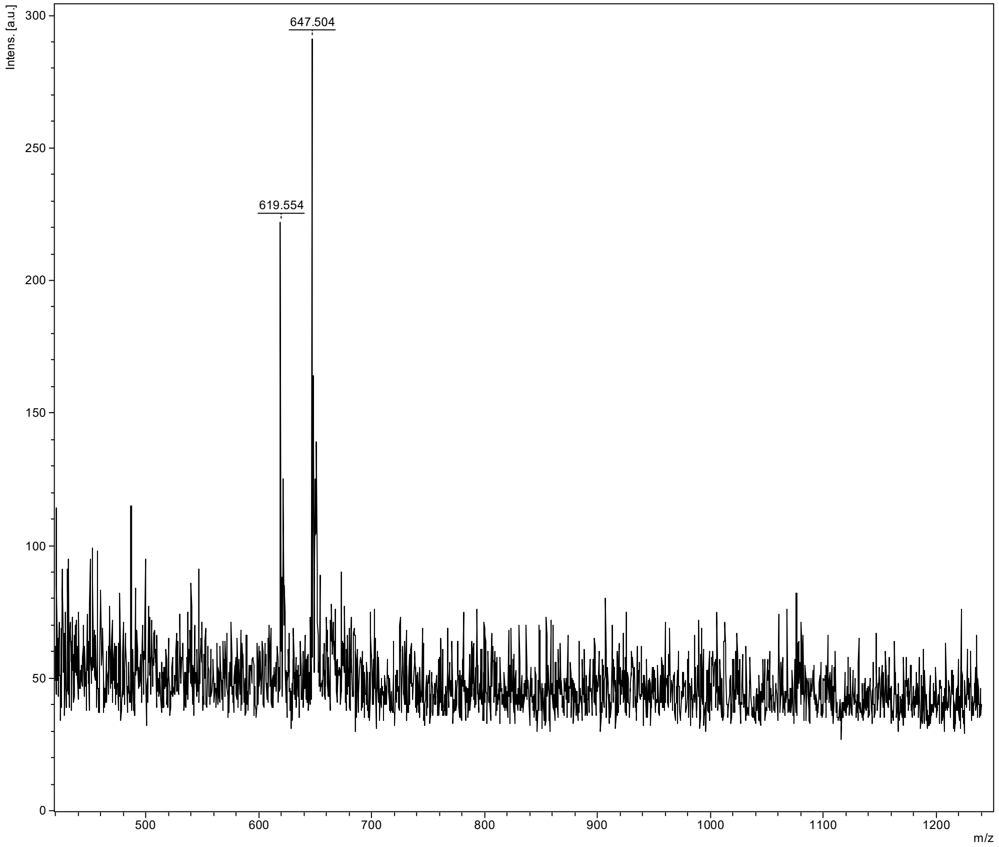

Antracene:

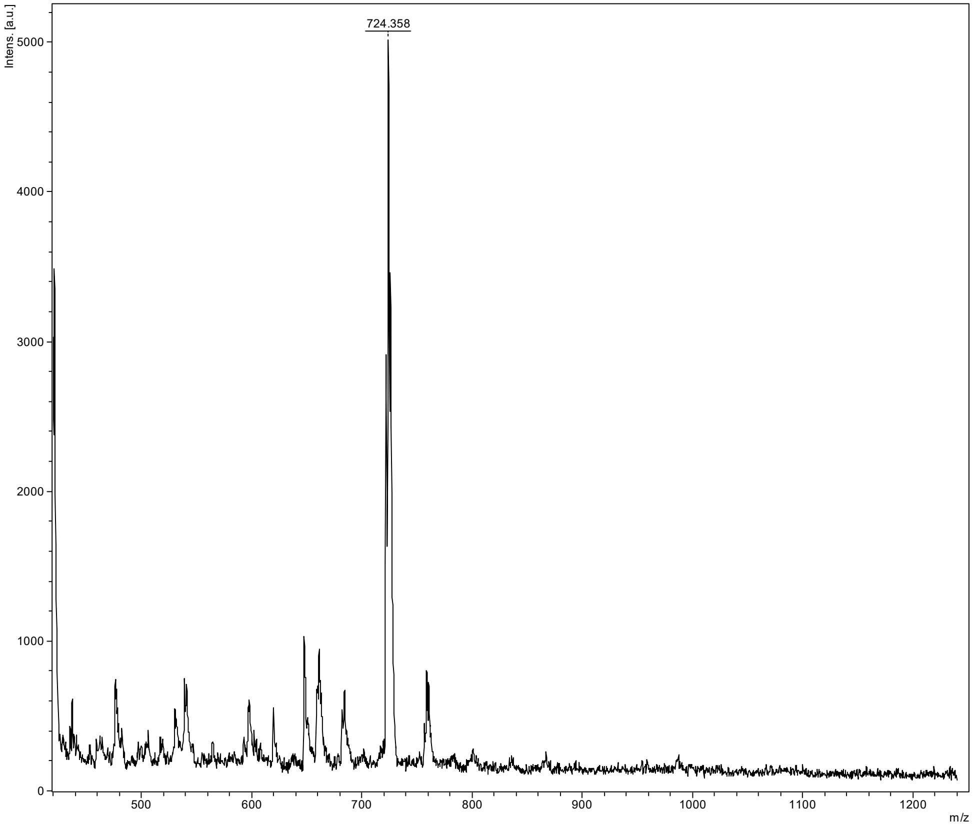

AT:

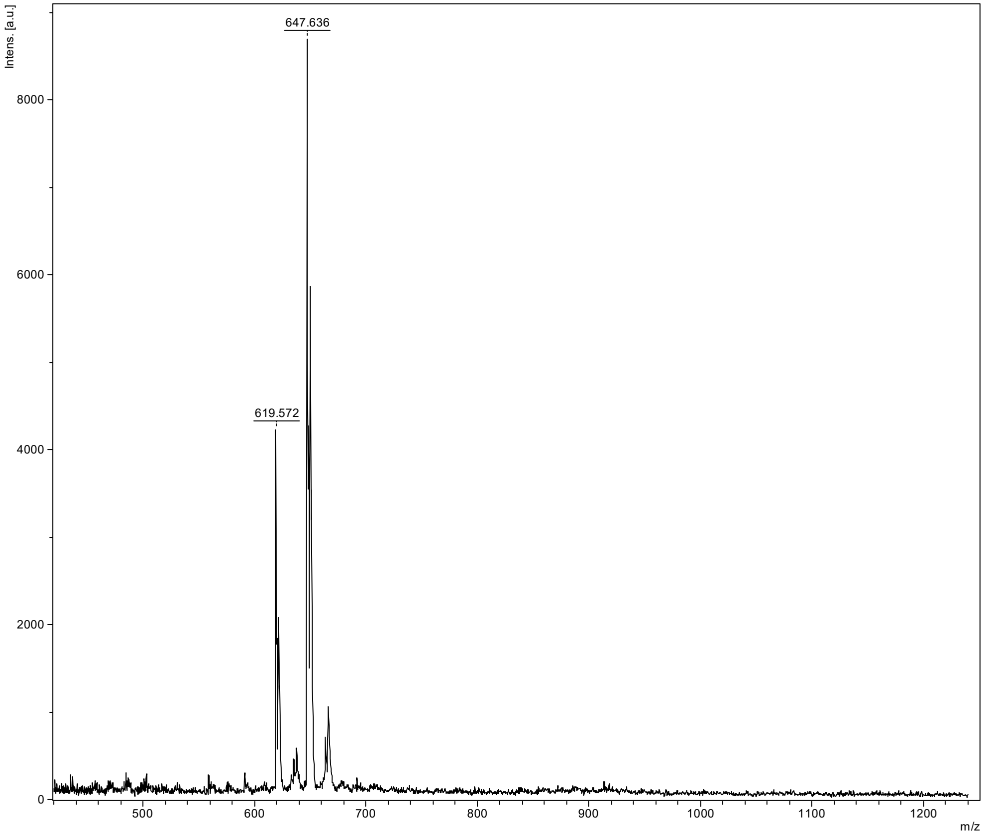

DCTB:

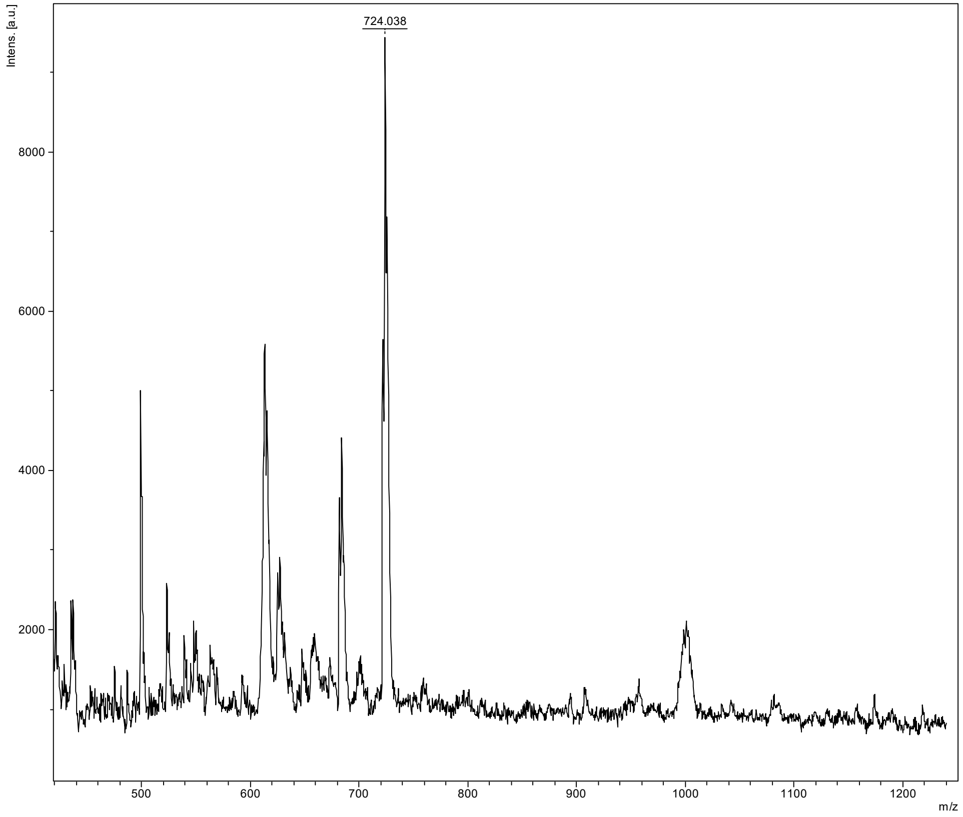

WM:

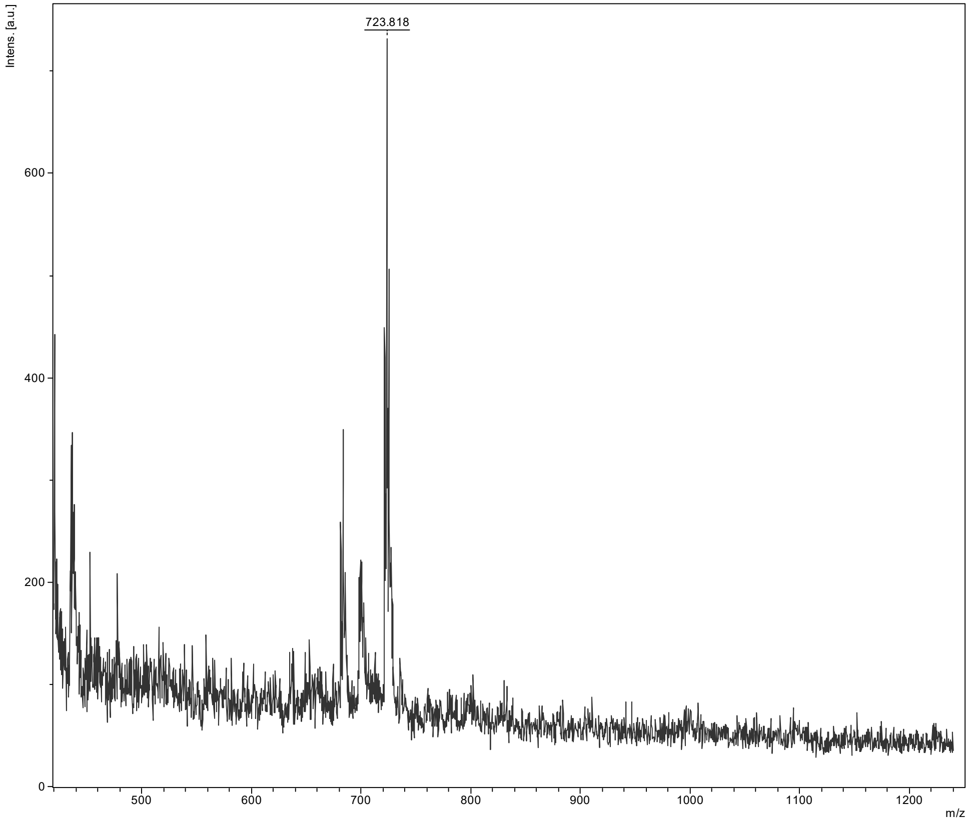

Figure 24S. MALDI spectra of complex **5c** on different matrices

X-Ray analysis

Table 2S. Crystal data and structure refinement for **4d** (CCDC 2017202).

Parameter	**4d** (CCDC 2017202)	
Empirical formula	$C_{13}H_{12}N_3OSe$	
Formula weight	305.22	
Temperature	293(2) *K*	
Wavelength	1.54186 E	
Crystal system	Monoclinic	
Space group	*P* 21/*c*	
Unit cell dimensions	*a* = 6.8328(2) E<br>*b* = 14.7453(3) E<br>*c* = 12.9522(2) E	α = 90°<br>β = 99.188(2)°<br>γ = 90°
Volume	1288.21(5) E^3	
Z	4	
Density (calculated)	1.574 Mg/m^3	
Absorption coefficient	3.881 mm^{-1}	
F(000)	612	
Theta range for data collection.	4.577 to 70.496°	
Index ranges	$-8 \leq h \leq 6$<br>$-14 \leq k \leq 17$<br>$-15 \leq l \leq 15$	
Reflections collected	13255	
Independent reflections	2441 [$R_{(int)}$ = 0.0441]	
Completeness to theta = 66.638°	99.6 %	
Refinement method	Full-matrix least-squares on F^2	
Data / restraints / parameters	2441 / 6 / 200	
Goodness-of-fit on F^2	1.038	
Final R indices [*I*>2sigma(*I*)]	R_1 = 0.0415, wR_2 = 0.1157	
R indices (all data)	R_1 = 0.0568, wR_2 = 0.1229	
Extinction coefficient	0.0014(4)	
Largest diff. peak and hole	0.825 and -0.502 e. E^{-3}	

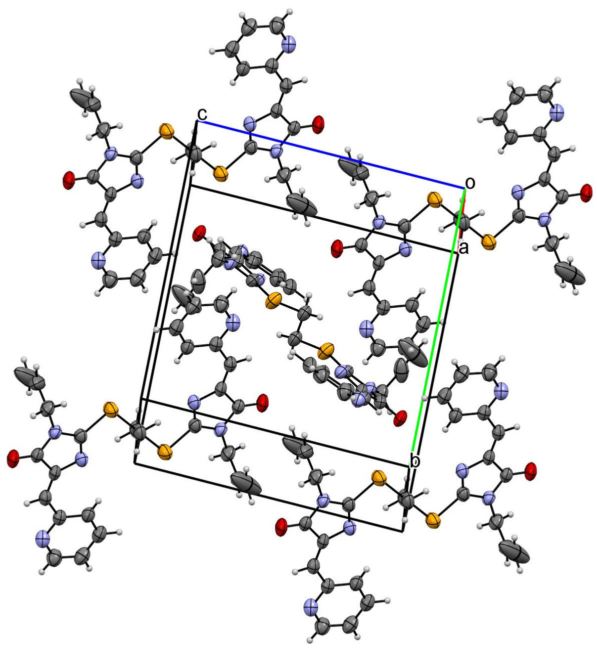

Table 3S. Bond lengths [E] and angles [°] for **4d**.

Se(1)-C(3)	1.884(3)
Se(1)-C(16)	1.963(4)
O(1)-C(1)	1.215(4)
N(2)-C(3)	1.384(4)
N(2)-C(1)	1.385(4)
N(2)-C(13)	1.459(4)
N(4)-C(3)	1.300(4)
N(4)-C(5)	1.401(4)
N(12)-C(11)	1.339(5)
N(12)-C(7)	1.348(4)
C(1)-C(5)	1.482(4)
C(5)-C(6)	1.342(5)
C(6)-C(7)	1.457(4)
C(6)-H(6)	0.93(4)
C(7)-C(8)	1.399(5)
C(8)-C(9)	1.377(5)
C(8)-H(8)	0.87(4)
C(9)-C(10)	1.369(6)
C(9)-H(9)	0.99(4)
C(10)-C(11)	1.381(6)
C(10)-H(10)	0.93(5)
C(11)-H(11)	0.94(4)
C(13)-C(14)	1.476(6)
C(13)-H(131)	0.95(5)
C(13)-H(132)	0.97(4)
C(14)-C(15)	1.087(9)
C(14)-H(14)	0.9300
C(15)-H(15A)	0.9300
C(15)-H(15B)	0.9300
C(16)-C(16)#1	1.506(9)
C(16)-H(161)	1.06(5)
C(16)-H(162)	0.98(4)
C(3)-Se(1)-C(16)	95.96(16)
C(3)-N(2)-C(1)	107.3(3)
C(3)-N(2)-C(13)	127.8(3)
C(1)-N(2)-C(13)	124.9(3)
C(3)-N(4)-C(5)	105.3(3)
C(11)-N(12)-C(7)	117.2(3)
O(1)-C(1)-N(2)	125.6(3)
O(1)-C(1)-C(5)	130.8(3)
N(2)-C(1)-C(5)	103.6(2)
N(4)-C(3)-N(2)	114.9(3)

N(4)-C(3)-Se(1)	125.3(2)
N(2)-C(3)-Se(1)	119.8(2)
C(6)-C(5)-N(4)	128.9(3)
C(6)-C(5)-C(1)	122.2(3)
N(4)-C(5)-C(1)	108.9(3)
C(5)-C(6)-C(7)	129.3(3)
C(5)-C(6)-H(6)	116(2)
C(7)-C(6)-H(6)	114(2)
N(12)-C(7)-C(8)	121.9(3)
N(12)-C(7)-C(6)	114.1(3)
C(8)-C(7)-C(6)	124.0(3)
C(9)-C(8)-C(7)	119.2(3)
C(9)-C(8)-H(8)	122(3)
C(7)-C(8)-H(8)	119(3)
C(10)-C(9)-C(8)	119.1(3)
C(10)-C(9)-H(9)	121(2)
C(8)-C(9)-H(9)	120(2)
C(9)-C(10)-C(11)	118.6(4)
C(9)-C(10)-H(10)	119(3)
C(11)-C(10)-H(10)	122(3)
N(12)-C(11)-C(10)	123.9(4)
N(12)-C(11)-H(11)	115(3)
C(10)-C(11)-H(11)	121(3)
N(2)-C(13)-C(14)	112.9(4)
N(2)-C(13)-H(131)	106(3)
C(14)-C(13)-H(131)	111(3)
N(2)-C(13)-H(132)	109(2)
C(14)-C(13)-H(132)	112(2)
H(131)-C(13)-H(132)	105(4)
C(15)-C(14)-C(13)	136.6(7)
C(15)-C(14)-H(14)	111.7
C(13)-C(14)-H(14)	111.7
C(14)-C(15)-H(15A)	120.0
C(14)-C(15)-H(15B)	120.0
H(15A)-C(15)-H(15B)	120.0
C(16)#1-C(16)-Se(1)	110.8(4)
C(16)#1-C(16)-H(161)	116(2)
Se(1)-C(16)-H(161)	96(2)
C(16)#1-C(16)-H(162)	118(2)
Se(1)-C(16)-H(162)	101(2)
H(161)-C(16)-H(162)	110(3)

Symmetry transformations used to generate equivalent atoms:

#1 -x+1,-y+1,-z+1

Table 4S. Torsion angles [°] for **4d**.

C(3)-N(2)-C(1)-O(1)	179.8(3)
C(13)-N(2)-C(1)-O(1)	0.2(5)
C(3)-N(2)-C(1)-C(5)	-0.4(3)
C(13)-N(2)-C(1)-C(5)	-180.0(3)
C(5)-N(4)-C(3)-N(2)	0.4(4)
C(5)-N(4)-C(3)-Se(1)	-177.5(2)
C(1)-N(2)-C(3)-N(4)	0.0(4)
C(13)-N(2)-C(3)-N(4)	179.5(3)
C(1)-N(2)-C(3)-Se(1)	178.1(2)
C(13)-N(2)-C(3)-Se(1)	-2.4(5)
C(16)-Se(1)-C(3)-N(4)	7.4(3)
C(16)-Se(1)-C(3)-N(2)	-170.5(3)
C(3)-N(4)-C(5)-C(6)	178.5(3)
C(3)-N(4)-C(5)-C(1)	-0.7(3)
O(1)-C(1)-C(5)-C(6)	1.2(5)
N(2)-C(1)-C(5)-C(6)	-178.6(3)
O(1)-C(1)-C(5)-N(4)	-179.5(3)
N(2)-C(1)-C(5)-N(4)	0.7(3)
N(4)-C(5)-C(6)-C(7)	0.8(6)
C(1)-C(5)-C(6)-C(7)	179.9(3)
C(11)-N(12)-C(7)-C(8)	0.2(5)
C(11)-N(12)-C(7)-C(6)	-178.9(3)
C(5)-C(6)-C(7)-N(12)	172.5(3)
C(5)-C(6)-C(7)-C(8)	-6.5(5)
N(12)-C(7)-C(8)-C(9)	-1.0(5)
C(6)-C(7)-C(8)-C(9)	178.0(3)
C(7)-C(8)-C(9)-C(10)	0.9(5)
C(8)-C(9)-C(10)-C(11)	0.0(5)
C(7)-N(12)-C(11)-C(10)	0.8(5)
C(9)-C(10)-C(11)-N(12)	-1.0(6)
C(3)-N(2)-C(13)-C(14)	-82.7(4)
C(1)-N(2)-C(13)-C(14)	96.8(4)
N(2)-C(13)-C(14)-C(15)	-13.5(11)

Symmetry transformations used to generate equivalent atoms:

#1 -x+1,-y+1,-z+1

Table 5S. Hydrogen bonds for **4d** [E and °].

D-H...A	d(D-H)	d(H...A)	d(D...A)	<(DHA)
C(8)-H(8)…N(4)	0.87(4)	2.47(4)	3.072(5)	128(4)
C(10)-H(10)...O(1)#2	0.93(5)	2.54(5)	3.275(4)	137(3)

Symmetry transformations used to generate equivalent atoms:

#1 -x+1,-y+1,-z+1 #2 x+1,-y+1/2,z-1/2

Table 6S. Crystal data and structure refinement for **5d** (CCDC 2064245).

Parameter	**5d** (CCDC 2064245)	
Empirical formula	$C_{26}H_{24}Cl_4Cu_2N_6O_3Se_2$	
Formula weight	895.31	
Temperature	295(2) *K*	
Wavelength	1.54186 Å	
Crystal system	Monoclinic	
Space group	C 2/c	
Unit cell dimensions	*a* = 12.6876(9) Å<br>*b* = 27.208(2) Å<br>*c* = 9.5136(8) Å	*α* = 90°<br>*β* = 100.228(4)°<br>*γ* = 90°
Volume	3231.9(4) Å	
Z	4	
Density (calculated)	1.840 Mg/m^3	
Absorption coefficient	7.575 mm^{-1}	
F(000)	1760	
Theta range for data collection.	5.620 to 66.990°	
Index ranges	-15≤h≤14<br>-32≤k≤15<br>-10≤l≤11	
Reflections collected	11381	
Independent reflections	2846 [$R_{(int)}$ = 0.1509]	
Completeness to theta = 66.690°	98.5 %	
Refinement method	Full-matrix least-squares on F^2	
Data / restraints / parameters	2846 / 1 / 192	
Goodness-of-fit on F^2	0.818	
Final R indices [*I*>2sigma(*I*)]	R_1 = 0.0855, wR_2 = 0.2145	
R indices (all data)	R_1 = 0.1688, wR_2 = 0.2654	
Extinction coefficient	0.00049(13)	
Largest diff. peak and hole	0.638 and -0.968 e.Å^{-3}	

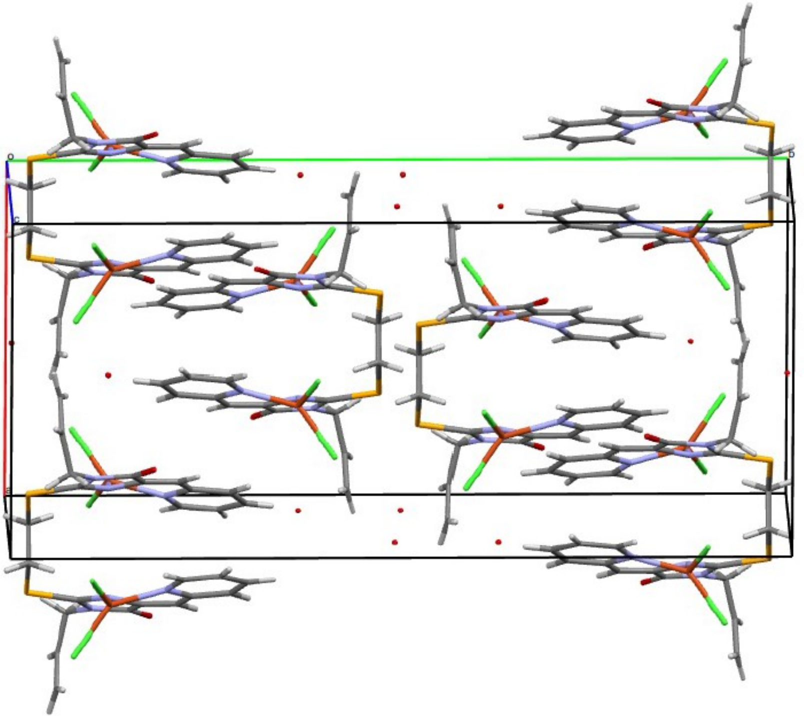

Table 7S. Bond lengths [Å] and angles [°] for **5d**.

Se(1)-C(4)	1.828(14)
Se(1)-C(14)	1.938(14)
Cu(1)-N(2)	1.992(9)
Cu(1)-N(3)	2.044(11)
Cu(1)-Cl(1)	2.199(3)
Cu(1)-Cl(2)	2.200(4)
O(1)-C(2)	1.235(13)
N(1)-C(2)	1.353(15)
N(1)-C(4)	1.395(14)
N(1)-C(11)	1.483(15)
N(2)-C(4)	1.322(14)
N(2)-C(3)	1.405(15)
N(3)-C(10)	1.358(16)
N(3)-C(6)	1.374(16)
C(2)-C(3)	1.480(15)
C(3)-C(5)	1.318(15)
C(5)-C(6)	1.431(17)
C(5)-H(5)	0.9300
C(6)-C(7)	1.405(17)
C(7)-C(8)	1.380(18)
C(7)-H(7)	0.9300
C(8)-C(9)	1.38(2)
C(8)-H(8)	0.9300
C(9)-C(10)	1.34(2)
C(9)-H(9)	0.9300
C(10)-H(10)	0.9300
C(11)-C(12)	1.48(2)
C(11)-H(11A)	0.9700
C(11)-H(11B)	0.9700
C(12)-C(13)	1.404(9)
C(12)-H(12)	0.9300
C(13)-H(13A)	1.16(15)
C(13)-H(13B)	0.50(15)
C(14)-C(14)#1	1.37(3)
C(14)-H(14A)	0.9700
C(14)-H(14B)	0.9700
C(4)-Se(1)-C(14)	99.9(6)
N(2)-Cu(1)-N(3)	92.7(4)
N(2)-Cu(1)-Cl(1)	144.7(3)
N(3)-Cu(1)-Cl(1)	98.6(3)
N(2)-Cu(1)-Cl(2)	95.0(3)
N(3)-Cu(1)-Cl(2)	139.5(3)
Cl(1)-Cu(1)-Cl(2)	97.68(16)
C(2)-N(1)-C(4)	110.4(10)
C(2)-N(1)-C(11)	123.9(9)
C(4)-N(1)-C(11)	125.7(11)
C(4)-N(2)-C(3)	109.4(10)
C(4)-N(2)-Cu(1)	130.2(8)
C(3)-N(2)-Cu(1)	116.5(7)
C(10)-N(3)-C(6)	116.9(12)

C(10)-N(3)-Cu(1)	119.1(10)
C(6)-N(3)-Cu(1)	122.9(8)
O(1)-C(2)-N(1)	125.3(11)
O(1)-C(2)-C(3)	129.7(13)
N(1)-C(2)-C(3)	105.0(10)
C(5)-C(3)-N(2)	130.7(10)
C(5)-C(3)-C(2)	123.3(12)
N(2)-C(3)-C(2)	105.9(11)
N(2)-C(4)-N(1)	109.3(11)
N(2)-C(4)-Se(1)	130.9(9)
N(1)-C(4)-Se(1)	119.8(9)
C(3)-C(5)-C(6)	127.1(11)
C(3)-C(5)-H(5)	116.4
C(6)-C(5)-H(5)	116.4
N(3)-C(6)-C(7)	120.5(11)
N(3)-C(6)-C(5)	121.0(11)
C(7)-C(6)-C(5)	118.4(12)
C(8)-C(7)-C(6)	121.7(13)
C(8)-C(7)-H(7)	119.1
C(6)-C(7)-H(7)	119.1
C(7)-C(8)-C(9)	115.3(14)
C(7)-C(8)-H(8)	122.4
C(9)-C(8)-H(8)	122.4
C(10)-C(9)-C(8)	122.7(14)
C(10)-C(9)-H(9)	118.6
C(8)-C(9)-H(9)	118.6
C(9)-C(10)-N(3)	122.8(14)
C(9)-C(10)-H(10)	118.6
N(3)-C(10)-H(10)	118.6
C(12)-C(11)-N(1)	111.6(12)
C(12)-C(11)-H(11A)	109.3
N(1)-C(11)-H(11A)	109.3
C(12)-C(11)-H(11B)	109.3
N(1)-C(11)-H(11B)	109.3
H(11A)-C(11)-H(11B)	108.0
C(13)-C(12)-C(11)	140.5(19)
C(13)-C(12)-H(12)	109.8
C(11)-C(12)-H(12)	109.8
C(12)-C(13)-H(13A)	90(7)
C(12)-C(13)-H(13B)	125(10)
H(13A)-C(13)-H(13B)	109(10)
C(14)#1-C(14)-Se(1)	117.1(10)
C(14)#1-C(14)-H(14A)	108.0
Se(1)-C(14)-H(14A)	108.0
C(14)#1-C(14)-H(14B)	108.0
Se(1)-C(14)-H(14B)	108.0
H(14A)-C(14)-H(14B)	107.3

Symmetry transformations used to generate equivalent atoms:

#1 -x,y,-z+3/2

Table 8S. Torsion angles [°] for **5d**.

C(4)-N(1)-C(2)-O(1)	-179.8(13)
C(11)-N(1)-C(2)-O(1)	-1(2)
C(4)-N(1)-C(2)-C(3)	2.0(14)
C(11)-N(1)-C(2)-C(3)	-179.1(11)
C(4)-N(2)-C(3)-C(5)	178.3(14)
Cu(1)-N(2)-C(3)-C(5)	-21.8(18)
C(4)-N(2)-C(3)-C(2)	1.1(13)
Cu(1)-N(2)-C(3)-C(2)	161.0(8)
O(1)-C(2)-C(3)-C(5)	3(2)
N(1)-C(2)-C(3)-C(5)	-179.3(13)
O(1)-C(2)-C(3)-N(2)	-180.0(14)
N(1)-C(2)-C(3)-N(2)	-1.9(13)
C(3)-N(2)-C(4)-N(1)	0.1(14)
Cu(1)-N(2)-C(4)-N(1)	-156.2(8)
C(3)-N(2)-C(4)-Se(1)	-177.4(9)
Cu(1)-N(2)-C(4)-Se(1)	26.3(17)
C(2)-N(1)-C(4)-N(2)	-1.4(14)
C(11)-N(1)-C(4)-N(2)	179.7(10)
C(2)-N(1)-C(4)-Se(1)	176.4(9)
C(11)-N(1)-C(4)-Se(1)	-2.5(16)
C(14)-Se(1)-C(4)-N(2)	19.8(12)
C(14)-Se(1)-C(4)-N(1)	-157.5(9)
N(2)-C(3)-C(5)-C(6)	0(2)
C(2)-C(3)-C(5)-C(6)	177.0(12)
C(10)-N(3)-C(6)-C(7)	1.5(17)
Cu(1)-N(3)-C(6)-C(7)	-166.8(9)
C(10)-N(3)-C(6)-C(5)	-174.2(12)
Cu(1)-N(3)-C(6)-C(5)	17.5(15)
C(3)-C(5)-C(6)-N(3)	3(2)
C(3)-C(5)-C(6)-C(7)	-173.3(13)
N(3)-C(6)-C(7)-C(8)	-2.2(19)
C(5)-C(6)-C(7)-C(8)	173.7(12)
C(6)-C(7)-C(8)-C(9)	2(2)
C(7)-C(8)-C(9)-C(10)	-1(2)
C(8)-C(9)-C(10)-N(3)	0(2)
C(6)-N(3)-C(10)-C(9)	-1(2)
Cu(1)-N(3)-C(10)-C(9)	168.2(12)
C(2)-N(1)-C(11)-C(12)	86.6(16)
C(4)-N(1)-C(11)-C(12)	-94.6(15)
N(1)-C(11)-C(12)-C(13)	20(3)

Symmetry transformations used to generate equivalent atoms:

#1 -x,y,-z+3/2

Table 9S. Hydrogen bonds for **5d** [Å and °].

D-H...A	d(D-H)	d(H...A)	d(D...A)	<(DHA)
C(5)-H(5)...O(1)#2	0.93	2.41	3.298(15)	159.4
C(10)-H(10)...Cl(1)	0.93	2.59	3.223(17)	125.5
C(11)-H(11A)...Se(1)#3	0.97	3.03	3.757(13)	133.3
C(12)-H(12)...O(3)#4	0.93	2.31	3.17(3)	154.0
C(14)-H(14A)...Se(1)#5	0.97	3.14	3.895(11)	136.3
C(14)-H(14B)...Cl(1)	0.97	2.75	3.635(13)	151.7
C(14)-H(14B)...N(2)	0.97	2.68	3.195(16)	113.6

Symmetry transformations used to generate equivalent atoms:

#1 -x,y,-z+3/2   #2 -x+1/2,-y+1/2,-z+1   #3 x,-y,z-1/2

#4 -x+1,-y,-z+1   #5 x,-y,z+1/2

Table 10S. Crystal data and structure refinement for **5e** (CCDC 2017198).

Parameter	**5e** (CCDC 2017198)	
Empirical formula	$C_{26}H_{24}Cl_4Cu_2N_6O_3Se_2$	
Formula weight	895.31	
Temperature	293(2) *K*	
Wavelength	1.54186 Å	
Crystal system	Monoclinic	
Space group	*P* 21/n	
Unit cell dimensions	*a* = 10.0633(3) Å	*α* = 90°
	b = 28.0640(10) Å	*β* = 93.491(2)°
	c = 11.2909(3) Å	*γ* = 90°
Volume	3182.82(17) Å	
Z	4	
Density (calculated)	1.868 Mg/m^3	
Absorption coefficient	7.692 mm^{-1}	
F(000)	1760	
Theta range for data collection.	4.227 to 66.950°	
Index ranges	-11≤h≤7<br>-33≤k≤33<br>-13≤l≤12	
Reflections collected	32166	
Independent reflections	5468 [$R_{(int)}$ = 0.1833]	
Completeness to theta = 66.638°	96.5 %	
Refinement method	Full-matrix least-squares on F^2	
Data / restraints / parameters	5468 / 3 / 394	
Goodness-of-fit on F^2	0.774	
Final R indices [*I*>2sigma(*I*)]	R_1 = 0.0571, wR_2 = 0.1125	
R indices (all data)	R_1 = 0.1419, wR_2 = 0.1345	
Extinction coefficient	n/a	
Largest diff. peak and hole	0.841 and -1.266 e.Å^{-3}	

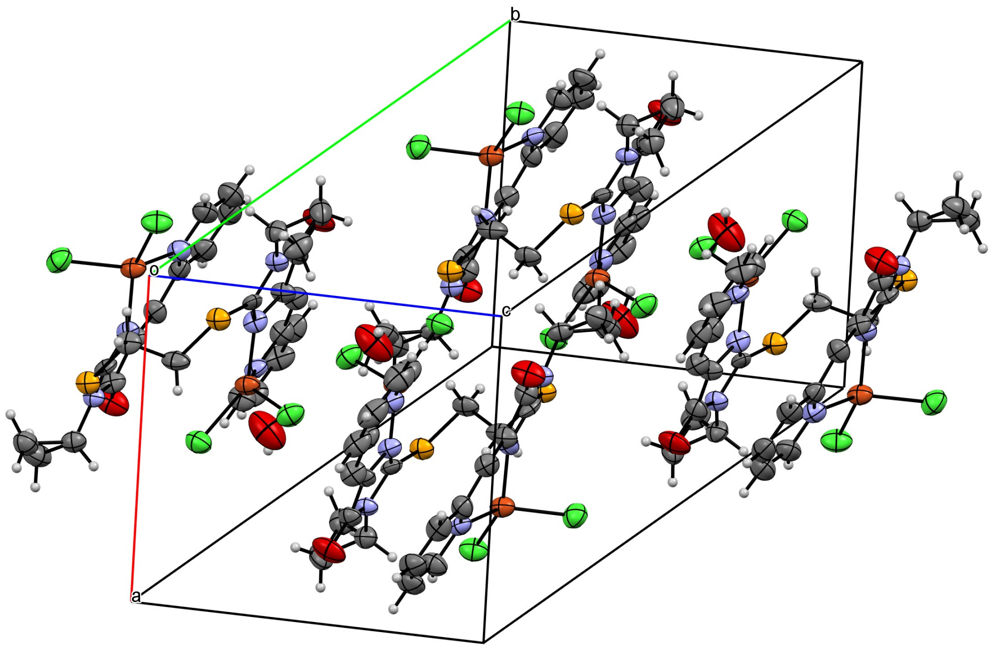

Table 11S. Bond lengths [Å] and angles [°] for **5e**.

Cu(1A)-N(2A)	1.966(7)
Cu(1A)-N(1A)	2.040(7)
Cu(1A)-Cl(1A)	2.214(3)
Cu(1A)-Cl(2A)	2.220(2)
Se(1A)-C(10A)	1.871(9)
Se(1A)-C(14A)	1.968(9)
O(1A)-C(9A)	1.211(10)
N(1A)-C(6A)	1.366(11)
N(1A)-C(2A)	1.367(11)
N(2A)-C(10A)	1.334(11)
N(2A)-C(8A)	1.400(10)
N(3A)-C(9A)	1.379(10)
N(3A)-C(10A)	1.400(11)
N(3A)-C(11A)	1.431(11)
C(2A)-C(3A)	1.351(13)
C(2A)-H(2A)	0.9300
C(3A)-C(4A)	1.357(15)
C(3A)-H(3A)	0.9300
C(4A)-C(5A)	1.347(14)
C(4A)-H(4A)	0.9300
C(5A)-C(6A)	1.408(12)
C(5A)-H(5A)	0.9300
C(6A)-C(7A)	1.447(13)
C(7A)-C(8A)	1.326(11)
C(8A)-C(9A)	1.480(13)
C(11A)-C(13A)	1.492(14)
C(11A)-C(12A)	1.499(13)
C(11A)-H(11A)	0.9800
C(12A)-C(13A)	1.461(15)
C(12A)-H(12A)	0.9700
C(12A)-H(12B)	0.9700
C(13A)-H(13A)	0.9700
C(13A)-H(13B)	0.9700
C(14A)-C(14B)	1.509(13)
C(14A)-H(14A)	0.9700
C(14A)-H(14B)	0.9700
Cu(1B)-N(2B)	1.972(7)
Cu(1B)-N(1B)	2.045(7)
Cu(1B)-Cl(2B)	2.203(2)
Cu(1B)-Cl(1B)	2.231(3)
Se(1B)-C(10B)	1.873(8)
Se(1B)-C(14B)	1.962(9)
O(1B)-C(9B)	1.203(10)
N(1B)-C(6B)	1.365(12)
N(1B)-C(2B)	1.373(12)
N(2B)-C(10B)	1.322(11)
N(2B)-C(8B)	1.394(10)
N(3B)-C(9B)	1.361(10)
N(3B)-C(10B)	1.386(10)
N(3B)-C(11B)	1.451(11)
C(2B)-C(3B)	1.347(14)
C(2B)-H(2B)	0.9300
C(3B)-C(4B)	1.377(17)
C(3B)-H(3B)	0.9300
C(4B)-C(5B)	1.361(15)
C(4B)-H(4B)	0.9300
C(5B)-C(6B)	1.385(12)
C(5B)-H(5B)	0.9300
C(6B)-C(7B)	1.449(13)
C(7B)-C(8B)	1.333(11)
C(8B)-C(9B)	1.496(13)
C(11B)-C(12B)	1.483(13)
C(11B)-C(13B)	1.490(14)
C(11B)-H(11B)	0.9800
C(12B)-C(13B)	1.456(15)
C(12B)-H(12C)	0.9700
C(12B)-H(12D)	0.9700
C(13B)-H(13C)	0.9700
C(13B)-H(13D)	0.9700
C(14B)-H(14C)	0.9700
C(14B)-H(14D)	0.9700
O(2)-H(22)	0.901(10)
O(2)-H(23)	0.901(11)
N(2A)-Cu(1A)-N(1A)	92.4(3)
N(2A)-Cu(1A)-Cl(1A)	95.4(2)
N(1A)-Cu(1A)-Cl(1A)	135.5(2)
N(2A)-Cu(1A)-Cl(2A)	145.1(2)
N(1A)-Cu(1A)-Cl(2A)	98.9(2)
Cl(1A)-Cu(1A)-Cl(2A)	99.16(11)
C(10A)-Se(1A)-C(14A)	97.9(4)
C(6A)-N(1A)-C(2A)	116.2(7)
C(6A)-N(1A)-Cu(1A)	123.0(6)
C(2A)-N(1A)-Cu(1A)	119.7(6)
C(10A)-N(2A)-C(8A)	105.1(7)
C(10A)-N(2A)-Cu(1A)	131.3(6)
C(8A)-N(2A)-Cu(1A)	119.4(5)
C(9A)-N(3A)-C(10A)	107.9(7)
C(9A)-N(3A)-C(11A)	126.1(7)
C(10A)-N(3A)-C(11A)	125.5(7)
C(3A)-C(2A)-N(1A)	123.5(9)
C(3A)-C(2A)-H(2A)	118.3
N(1A)-C(2A)-H(2A)	118.3
C(2A)-C(3A)-C(4A)	120.4(10)
C(2A)-C(3A)-H(3A)	119.8
C(4A)-C(3A)-H(3A)	119.8
C(5A)-C(4A)-C(3A)	118.5(9)
C(5A)-C(4A)-H(4A)	120.8
C(3A)-C(4A)-H(4A)	120.8
C(4A)-C(5A)-C(6A)	121.0(9)
C(4A)-C(5A)-H(5A)	119.5

C(6A)-C(5A)-H(5A)	119.5
N(1A)-C(6A)-C(5A)	120.4(8)
N(1A)-C(6A)-C(7A)	119.6(8)
C(5A)-C(6A)-C(7A)	120.0(8)
C(8A)-C(7A)-C(6A)	129.7(8)
C(7A)-C(8A)-N(2A)	126.2(8)
C(7A)-C(8A)-C(9A)	124.2(8)
N(2A)-C(8A)-C(9A)	109.6(7)
O(1A)-C(9A)-N(3A)	126.9(9)
O(1A)-C(9A)-C(8A)	128.9(8)
N(3A)-C(9A)-C(8A)	104.0(7)
N(2A)-C(10A)-N(3A)	113.4(7)
N(2A)-C(10A)-Se(1A)	130.4(6)
N(3A)-C(10A)-Se(1A)	116.2(6)
N(3A)-C(11A)-C(13A)	119.3(9)
N(3A)-C(11A)-C(12A)	117.5(8)
C(13A)-C(11A)-C(12A)	58.4(7)
N(3A)-C(11A)-H(11A)	116.4
C(13A)-C(11A)-H(11A)	116.4
C(12A)-C(11A)-H(11A)	116.4
C(13A)-C(12A)-C(11A)	60.5(7)
C(13A)-C(12A)-H(12A)	117.7
C(11A)-C(12A)-H(12A)	117.7
C(13A)-C(12A)-H(12B)	117.7
C(11A)-C(12A)-H(12B)	117.7
H(12A)-C(12A)-H(12B)	114.8
C(12A)-C(13A)-C(11A)	61.0(7)
C(12A)-C(13A)-H(13A)	117.7
C(11A)-C(13A)-H(13A)	117.7
C(12A)-C(13A)-H(13B)	117.7
C(11A)-C(13A)-H(13B)	117.7
H(13A)-C(13A)-H(13B)	114.8
C(14B)-C(14A)-Se(1A)	111.0(6)
C(14B)-C(14A)-H(14A)	109.4
Se(1A)-C(14A)-H(14A)	109.4
C(14B)-C(14A)-H(14B)	109.4
Se(1A)-C(14A)-H(14B)	109.4
H(14A)-C(14A)-H(14B)	
N(2B)-Cu(1B)-N(1B)	92.7(3)
N(2B)-Cu(1B)-Cl(2B)	147.6(2)
N(1B)-Cu(1B)-Cl(2B)	98.5(2)
N(2B)-Cu(1B)-Cl(1B)	93.9(2)
N(1B)-Cu(1B)-Cl(1B)	137.1(2)
Cl(2B)-Cu(1B)-Cl(1B)	98.12(11)
C(10B)-Se(1B)-C(14B)	99.9(4)
C(6B)-N(1B)-C(2B)	116.4(8)
C(6B)-N(1B)-Cu(1B)	123.2(6)
C(2B)-N(1B)-Cu(1B)	119.1(7)
C(10B)-N(2B)-C(8B)	107.0(7)
C(10B)-N(2B)-Cu(1B)	131.9(6)
C(8B)-N(2B)-Cu(1B)	117.2(5)
C(9B)-N(3B)-C(10B)	108.7(6)

C(9B)-N(3B)-C(11B)	125.5(6)
C(10B)-N(3B)-C(11B)	124.6(7)
C(3B)-C(2B)-N(1B)	122.6(11)
C(3B)-C(2B)-H(2B)	118.7
N(1B)-C(2B)-H(2B)	118.7
C(2B)-C(3B)-C(4B)	120.8(10)
C(2B)-C(3B)-H(3B)	119.6
C(4B)-C(3B)-H(3B)	119.6
C(5B)-C(4B)-C(3B)	117.8(10)
C(5B)-C(4B)-H(4B)	121.1
C(3B)-C(4B)-H(4B)	121.1
C(4B)-C(5B)-C(6B)	120.5(10)
C(4B)-C(5B)-H(5B)	119.7
C(6B)-C(5B)-H(5B)	119.7
N(1B)-C(6B)-C(5B)	121.7(9)
N(1B)-C(6B)-C(7B)	119.7(8)
C(5B)-C(6B)-C(7B)	118.6(9)
C(8B)-C(7B)-C(6B)	127.4(8)
C(7B)-C(8B)-N(2B)	129.8(8)
C(7B)-C(8B)-C(9B)	122.9(8)
N(2B)-C(8B)-C(9B)	107.3(7)
O(1B)-C(9B)-N(3B)	127.3(8)
O(1B)-C(9B)-C(8B)	128.3(8)
N(3B)-C(9B)-C(8B)	104.4(6)
N(2B)-C(10B)-N(3B)	112.3(7)
N(2B)-C(10B)-Se(1B)	131.0(6)
N(3B)-C(10B)-Se(1B)	116.7(6)
N(3B)-C(11B)-C(12B)	116.4(8)
N(3B)-C(11B)-C(13B)	117.7(9)
C(12B)-C(11B)-C(13B)	58.7(7)
N(3B)-C(11B)-H(11B)	117.1
C(12B)-C(11B)-H(11B)	117.1
C(13B)-C(11B)-H(11B)	117.1
C(13B)-C(12B)-C(11B)	60.9(7)
C(13B)-C(12B)-H(12C)	117.7
C(11B)-C(12B)-H(12C)	117.7
C(13B)-C(12B)-H(12D)	117.7
C(11B)-C(12B)-H(12D)	117.7
H(12C)-C(12B)-H(12D)	114.8
C(12B)-C(13B)-C(11B)	60.4(7)
C(12B)-C(13B)-H(13C)	117.7
C(11B)-C(13B)-H(13C)	117.7
C(12B)-C(13B)-H(13D)	117.7
C(11B)-C(13B)-H(13D)	117.7
H(13C)-C(13B)-H(13D)	114.8
C(14A)-C(14B)-Se(1B)	109.2(5)
C(14A)-C(14B)-H(14C)	109.8
Se(1B)-C(14B)-H(14C)	109.8
C(14A)-C(14B)-H(14D)	109.8
Se(1B)-C(14B)-H(14D)	109.8
H(14C)-C(14B)-H(14D)	108.3
H(22)-O(2)-H(23)	103.6(16)

Table 12S. Torsion angles [°] for **5e**.

C(6A)-N(1A)-C(2A)-C(3A)	0.4(15)
Cu(1A)-N(1A)-C(2A)-C(3A)	169.4(9)
N(1A)-C(2A)-C(3A)-C(4A)	-0.9(18)
C(2A)-C(3A)-C(4A)-C(5A)	-0.5(18)
C(3A)-C(4A)-C(5A)-C(6A)	2.3(18)
C(2A)-N(1A)-C(6A)-C(5A)	1.4(14)
Cu(1A)-N(1A)-C(6A)-C(5A)	-167.2(7)
C(2A)-N(1A)-C(6A)-C(7A)	-176.2(10)
Cu(1A)-N(1A)-C(6A)-C(7A)	15.2(13)
C(4A)-C(5A)-C(6A)-N(1A)	-2.8(16)
C(4A)-C(5A)-C(6A)-C(7A)	174.8(11)
N(1A)-C(6A)-C(7A)-C(8A)	8.1(17)
C(5A)-C(6A)-C(7A)-C(8A)	-169.5(11)
C(6A)-C(7A)-C(8A)-N(2A)	-5.4(18)
C(6A)-C(7A)-C(8A)-C(9A)	174.4(11)
C(10A)-N(2A)-C(8A)-C(7A)	-179.6(10)
Cu(1A)-N(2A)-C(8A)-C(7A)	-20.3(13)
C(10A)-N(2A)-C(8A)-C(9A)	0.6(11)
Cu(1A)-N(2A)-C(8A)-C(9A)	159.9(7)
C(10A)-N(3A)-C(9A)-O(1A)	-175.4(11)
C(11A)-N(3A)-C(9A)-O(1A)	-3.3(17)
C(10A)-N(3A)-C(9A)-C(8A)	1.3(10)
C(11A)-N(3A)-C(9A)-C(8A)	173.4(9)
C(7A)-C(8A)-C(9A)-O(1A)	-4.4(19)
N(2A)-C(8A)-C(9A)-O(1A)	175.4(11)
C(7A)-C(8A)-C(9A)-N(3A)	179.0(9)
N(2A)-C(8A)-C(9A)-N(3A)	-1.2(11)
C(8A)-N(2A)-C(10A)-N(3A)	0.3(11)
Cu(1A)-N(2A)-C(10A)-N(3A)	-155.5(6)
C(8A)-N(2A)-C(10A)-Se(1A)	-176.3(7)
Cu(1A)-N(2A)-C(10A)-Se(1A)	27.9(14)
C(9A)-N(3A)-C(10A)-N(2A)	-1.1(11)
C(11A)-N(3A)-C(10A)-N(2A)	-173.2(9)
C(9A)-N(3A)-C(10A)-Se(1A)	176.1(6)
C(11A)-N(3A)-C(10A)-Se(1A)	3.9(12)
C(14A)-Se(1A)-C(10A)-N(2A)	16.9(10)
C(14A)-Se(1A)-C(10A)-N(3A)	-159.7(7)
C(9A)-N(3A)-C(11A)-C(13A)	118.9(10)
C(10A)-N(3A)-C(11A)-C(13A)	-70.4(13)
C(9A)-N(3A)-C(11A)-C(12A)	51.5(13)
C(10A)-N(3A)-C(11A)-C(12A)	-137.8(10)
N(3A)-C(11A)-C(12A)-C(13A)	109.1(10)
N(3A)-C(11A)-C(13A)-C(12A)	-106.1(10)
C(6B)-N(1B)-C(2B)-C(3B)	1.9(15)

Cu(1B)-N(1B)-C(2B)-C(3B)	169.4(8)
N(1B)-C(2B)-C(3B)-C(4B)	-4.4(17)
C(2B)-C(3B)-C(4B)-C(5B)	3.2(18)
C(3B)-C(4B)-C(5B)-C(6B)	0.3(17)
C(2B)-N(1B)-C(6B)-C(5B)	1.7(14)
Cu(1B)-N(1B)-C(6B)-C(5B)	-165.2(7)
C(2B)-N(1B)-C(6B)-C(7B)	-176.1(10)
Cu(1B)-N(1B)-C(6B)-C(7B)	17.0(13)
C(4B)-C(5B)-C(6B)-N(1B)	-2.8(16)
C(4B)-C(5B)-C(6B)-C(7B)	175.1(11)
N(1B)-C(6B)-C(7B)-C(8B)	6.5(17)
C(5B)-C(6B)-C(7B)-C(8B)	-171.4(11)
C(6B)-C(7B)-C(8B)-N(2B)	-4.9(19)
C(6B)-C(7B)-C(8B)-C(9B)	175.8(10)
C(10B)-N(2B)-C(8B)-C(7B)	179.8(10)
Cu(1B)-N(2B)-C(8B)-C(7B)	-19.8(14)
C(10B)-N(2B)-C(8B)-C(9B)	-0.8(11)
Cu(1B)-N(2B)-C(8B)-C(9B)	159.5(6)
C(10B)-N(3B)-C(9B)-O(1B)	-175.6(11)
C(11B)-N(3B)-C(9B)-O(1B)	-7.9(16)
C(10B)-N(3B)-C(9B)-C(8B)	4.4(10)
C(11B)-N(3B)-C(9B)-C(8B)	172.1(9)
C(7B)-C(8B)-C(9B)-O(1B)	-2.9(18)
N(2B)-C(8B)-C(9B)-O(1B)	177.7(11)
C(7B)-C(8B)-C(9B)-N(3B)	177.2(9)
N(2B)-C(8B)-C(9B)-N(3B)	-2.3(10)
C(8B)-N(2B)-C(10B)-N(3B)	3.7(11)
Cu(1B)-N(2B)-C(10B)-N(3B)	-152.6(7)
C(8B)-N(2B)-C(10B)-Se(1B)	-176.4(7)
Cu(1B)-N(2B)-C(10B)-Se(1B)	27.3(14)
C(9B)-N(3B)-C(10B)-N(2B)	-5.4(11)
C(11B)-N(3B)-C(10B)-N(2B)	-173.2(9)
C(9B)-N(3B)-C(10B)-Se(1B)	174.7(6)
C(11B)-N(3B)-C(10B)-Se(1B)	6.9(12)
C(14B)-Se(1B)-C(10B)-N(2B)	18.0(10)
C(14B)-Se(1B)-C(10B)-N(3B)	-162.1(7)
C(9B)-N(3B)-C(11B)-C(12B)	55.1(13)
C(10B)-N(3B)-C(11B)-C(12B)	-139.1(9)
C(9B)-N(3B)-C(11B)-C(13B)	121.8(9)
C(10B)-N(3B)-C(11B)-C(13B)	-72.3(12)
N(3B)-C(11B)-C(12B)-C(13B)	107.8(10)
N(3B)-C(11B)-C(13B)-C(12B)	-105.6(9)
Se(1A)-C(14A)-C(14B)-Se(1B)	-175.7(4)

Table 13S. Hydrogen bonds for **5e** [Å and °].

D-H...A	d(D-H)	d(H...A)	d(D...A)	<(DHA)
C(2A)-H(2A)...Cl(2A)	0.93	2.62	3.249(10)	125.7
C(5A)-H(5A)...O(1B)#1	0.93	2.66	3.467(13)	146.0
C(11A)-H(11A)...Cl(2A)#2	0.98	2.98	3.823(10)	145.3
C(12A)-H(12A)...Cl(1A)#2	0.97	2.87	3.830(12)	172.3
C(13A)-H(13B)...Se(1B)#3	0.97	3.12	3.912(11)	139.9
C(14A)-H(14A)...Se(1B)#4	0.97	3.06	3.985(10)	158.9
C(14A)-H(14B)...Cl(2A)	0.97	2.88	3.609(9)	132.3
C(14A)-H(14B)...N(2A)	0.97	2.58	3.174(12)	119.3
C(2B)-H(2B)...Cl(2B)	0.93	2.58	3.213(12)	125.6
C(5B)-H(5B)...O(1A)#5	0.93	2.64	3.464(13)	147.5
C(11B)-H(11B)...Cl(2B)#6	0.98	2.88	3.706(11)	142.4
C(12B)-H(12C)...Cl(1B)#6	0.97	2.98	3.941(11)	172.0
C(14B)-H(14C)...Cl(2B)#3	0.97	2.76	3.624(9)	148.2
C(14B)-H(14D)...Cl(2B)	0.97	2.84	3.760(10)	157.7
O(2)-H(22)...Cl(1B)#7	0.901(10)	2.39(4)	3.263(10)	163(13)
O(2)-H(23)...Cl(1A)	0.901(11)	2.50(10)	3.250(10)	140(13)

Symmetry transformations used to generate equivalent atoms:

#1 x-1/2,-y+1/2,z+1/2 #2 x-1,y,z #3 -x+1,-y+1,-z+1 #4 -x+2,-y+1,-z+1 #5 x+1/2,-y+1/2,z-1/2 #6 x+1,y,z #7 x,y,z+1

Table 14S. Crystal data and structure refinement for **5i** (CCDC 2125344).

Parameter	**5i** (CCDC 2125344)	
Empirical formula	$C_{30}H_{32}Cl_4Cu_2N_6O_2Se_2$	
Formula weight	935.41	
Temperature	296(2) *K*	
Wavelength	1.54186 Å	
Crystal system	Monoclinic	
Space group	C 2/c	
Unit cell dimensions	a = 27.8120(10) Å<br>b = 10.3367(4) Å<br>c = 13.4574(6) Å	α = 90°<br>β = 118.406(3)°<br>γ = 90°
Volume	3403.0(3) Å	
Z	4	
Density (calculated)	1.826 Mg/m^3	
Absorption coefficient	7.201 mm^{-1}	
F(000)	1856	
Theta range for data collection.	3.614 to 66.990°	
Index ranges	$-33 \leq h \leq 33$<br>$-8 \leq k \leq 12$<br>$-15 \leq l \leq 15$	
Reflections collected	16893	
Independent reflections	2989 [$R_{(int)}$ = 0.1318]	
Completeness to theta = 66.638°	98.6 %	
Refinement method	Full-matrix least-squares on F^2	
Data / restraints / parameters	2989 / 5 / 193	
Goodness-of-fit on F^2	0.920	
Final R indices [I>2sigma(I)]	R_1 = 0.0855, wR_2 = 0.2388	
R indices (all data)	R_1 = 0.1520, wR_2 = 0.2776	
Extinction coefficient	n/a	
Largest diff. peak and hole	1.506 and -1.529 e.$Å^{-3}$	

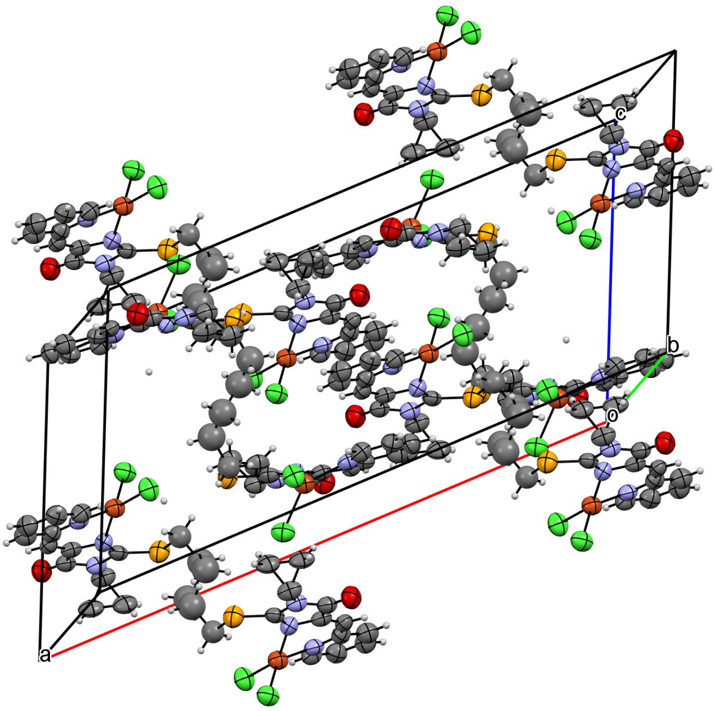

Table 15S. Bond lengths [Å] and angles [°] for **5i**.

Cu(1)-N(2)	1.974(9)
Cu(1)-N(1)	2.072(10)
Cu(1)-Cl(1)	2.232(3)
Cu(1)-Cl(2)	2.253(4)
O(1)-C(9)	1.198(13)
N(1)-C(6)	1.334(14)
N(1)-C(2)	1.349(14)
N(2)-C(10)	1.351(14)
N(2)-C(8)	1.411(14)
N(3)-C(9)	1.407(15)
N(3)-C(10)	1.410(13)
N(3)-C(11)	1.415(13)
C(2)-C(3)	1.374(18)
C(2)-H(2)	0.9300
C(3)-C(4)	1.387(19)
C(3)-H(3)	0.9300
C(4)-C(5)	1.406(16)
C(4)-H(4)	0.9300
C(5)-C(6)	1.392(17)
C(5)-H(5)	0.9300
C(6)-C(7)	1.454(15)
C(7)-C(8)	1.324(16)
C(7)-H(7)	0.9300
C(8)-C(9)	1.492(15)
C(10)-Se(1)	1.827(12)
C(11)-C(12)	1.471(17)
C(11)-C(13)	1.476(18)
C(11)-H(11)	0.45(17)
C(12)-C(13)	1.459(19)
C(12)-H(12A)	0.9700
C(12)-H(12B)	0.9700
C(13)-H(13A)	0.9700
C(13)-H(13B)	0.9700
Se(1)-C(14)	1.912(13)
C(14)-C(15)	1.491(9)
C(14)-H(14A)	0.9700
C(14)-H(14B)	0.9700
C(15)-C(16)	1.486(9)
C(15)-H(15A)	0.9700
C(15)-H(15B)	0.9700
C(16)-C(16)#1	1.557(10)
C(16)-H(16A)	0.9700
C(16)-H(16B)	0.9700
N(2)-Cu(1)-N(1)	91.4(4)
N(2)-Cu(1)-Cl(1)	147.7(3)
N(1)-Cu(1)-Cl(1)	98.4(3)
N(2)-Cu(1)-Cl(2)	95.1(3)
N(1)-Cu(1)-Cl(2)	139.9(3)
Cl(1)-Cu(1)-Cl(2)	96.83(13)
C(6)-N(1)-C(2)	118.4(11)
C(6)-N(1)-Cu(1)	122.2(7)
C(2)-N(1)-Cu(1)	118.8(8)
C(10)-N(2)-C(8)	106.5(8)
C(10)-N(2)-Cu(1)	131.2(8)
C(8)-N(2)-Cu(1)	117.3(7)
C(9)-N(3)-C(10)	109.3(9)
C(9)-N(3)-C(11)	124.3(9)
C(10)-N(3)-C(11)	126.4(11)
N(1)-C(2)-C(3)	123.2(11)
N(1)-C(2)-H(2)	118.4
C(3)-C(2)-H(2)	118.4
C(2)-C(3)-C(4)	119.5(11)
C(2)-C(3)-H(3)	120.2
C(4)-C(3)-H(3)	120.2
C(3)-C(4)-C(5)	117.0(12)
C(3)-C(4)-H(4)	121.5
C(5)-C(4)-H(4)	121.5
C(6)-C(5)-C(4)	120.2(12)
C(6)-C(5)-H(5)	119.9
C(4)-C(5)-H(5)	119.9
N(1)-C(6)-C(5)	121.3(10)
N(1)-C(6)-C(7)	121.0(10)
C(5)-C(6)-C(7)	117.7(11)
C(8)-C(7)-C(6)	128.2(11)
C(8)-C(7)-H(7)	115.9
C(6)-C(7)-H(7)	115.9
C(7)-C(8)-N(2)	127.0(10)
C(7)-C(8)-C(9)	123.6(11)
N(2)-C(8)-C(9)	109.4(10)
O(1)-C(9)-N(3)	126.5(10)
O(1)-C(9)-C(8)	130.5(12)
N(3)-C(9)-C(8)	103.1(9)
N(2)-C(10)-N(3)	111.5(10)
N(2)-C(10)-Se(1)	131.9(8)
N(3)-C(10)-Se(1)	116.5(8)
N(3)-C(11)-C(12)	119.7(11)
N(3)-C(11)-C(13)	121.4(10)
C(12)-C(11)-C(13)	59.4(9)
N(3)-C(11)-H(11)	115.0
C(12)-C(11)-H(11)	115.0
C(13)-C(11)-H(11)	115.0
C(13)-C(12)-C(11)	60.5(9)
C(13)-C(12)-H(12A)	117.7
C(11)-C(12)-H(12A)	117.7
C(13)-C(12)-H(12B)	117.7
C(11)-C(12)-H(12B)	117.7
H(12A)-C(12)-H(12B)	114.8
C(12)-C(13)-C(11)	60.2(9)
C(12)-C(13)-H(13A)	117.8
C(11)-C(13)-H(13A)	117.8
C(12)-C(13)-H(13B)	117.8
C(11)-C(13)-H(13B)	117.8
H(13A)-C(13)-H(13B)	114.9
C(10)-Se(1)-C(14)	101.7(6)
C(15)-C(14)-Se(1)	107.0(10)
C(15)-C(14)-H(14A)	110.3
Se(1)-C(14)-H(14A)	110.3
C(15)-C(14)-H(14B)	110.3
Se(1)-C(14)-H(14B)	110.3
H(14A)-C(14)-H(14B)	108.6
C(16)-C(15)-C(14)	121.5(11)
C(16)-C(15)-H(15A)	106.9
C(14)-C(15)-H(15A)	106.9
C(16)-C(15)-H(15B)	106.9
C(14)-C(15)-H(15B)	106.9
H(15A)-C(15)-H(15B)	106.7
C(15)-C(16)-C(16)#1	113.4(10)
C(15)-C(16)-H(16A)	108.9
C(16)#1-C(16)-H(16A)	108.9
C(15)-C(16)-H(16B)	108.9
C(16)#1-C(16)-H(16B)	108.9
H(16A)-C(16)-H(16B)	107.7

Symmetry transformations used to generate equivalent atoms:

#1 -x+3/2,-y+1/2,-z+2

Table 16S. Torsion angles [°] for **5i**.

C(6)-N(1)-C(2)-C(3)	4.7(18)
Cu(1)-N(1)-C(2)-C(3)	-167.1(10)
N(1)-C(2)-C(3)-C(4)	0.1(19)
C(2)-C(3)-C(4)-C(5)	-3.1(19)
C(3)-C(4)-C(5)-C(6)	1.5(19)
C(2)-N(1)-C(6)-C(5)	-6.4(17)
Cu(1)-N(1)-C(6)-C(5)	165.2(9)
C(2)-N(1)-C(6)-C(7)	173.5(10)
Cu(1)-N(1)-C(6)-C(7)	-15.0(15)
C(4)-C(5)-C(6)-N(1)	3.4(19)
C(4)-C(5)-C(6)-C(7)	-176.5(11)
N(1)-C(6)-C(7)-C(8)	-9.3(18)
C(5)-C(6)-C(7)-C(8)	170.6(12)
C(6)-C(7)-C(8)-N(2)	2.8(19)
C(6)-C(7)-C(8)-C(9)	-177.8(10)
C(10)-N(2)-C(8)-C(7)	-175.4(11)
Cu(1)-N(2)-C(8)-C(7)	26.8(14)
C(10)-N(2)-C(8)-C(9)	5.0(12)
Cu(1)-N(2)-C(8)-C(9)	-152.7(7)
C(10)-N(3)-C(9)-O(1)	-177.4(11)
C(11)-N(3)-C(9)-O(1)	0.7(19)
C(10)-N(3)-C(9)-C(8)	2.5(11)
C(11)-N(3)-C(9)-C(8)	-179.4(10)
C(7)-C(8)-C(9)-O(1)	-4(2)
N(2)-C(8)-C(9)-O(1)	175.3(12)
C(7)-C(8)-C(9)-N(3)	175.8(10)
N(2)-C(8)-C(9)-N(3)	-4.6(11)
C(8)-N(2)-C(10)-N(3)	-3.5(12)
Cu(1)-N(2)-C(10)-N(3)	150.0(7)
C(8)-N(2)-C(10)-Se(1)	-179.3(9)
Cu(1)-N(2)-C(10)-Se(1)	-25.9(15)
C(9)-N(3)-C(10)-N(2)	0.5(12)
C(11)-N(3)-C(10)-N(2)	-177.6(10)
C(9)-N(3)-C(10)-Se(1)	177.0(7)
C(11)-N(3)-C(10)-Se(1)	-1.1(15)
C(9)-N(3)-C(11)-C(12)	52.1(17)
C(10)-N(3)-C(11)-C(12)	-130.1(13)
C(9)-N(3)-C(11)-C(13)	122.3(13)
C(10)-N(3)-C(11)-C(13)	-59.9(17)
N(3)-C(11)-C(12)-C(13)	111.1(13)
N(3)-C(11)-C(13)-C(12)	-108.2(14)
N(2)-C(10)-Se(1)-C(14)	-8.7(12)
N(3)-C(10)-Se(1)-C(14)	175.6(8)
Se(1)-C(14)-C(15)-C(16)	141.7(13)
C(14)-C(15)-C(16)-C(16)#1	-104(2)

Symmetry transformations used to generate equivalent atoms:

#1 -x+3/2,-y+1/2,-z+2

Table 17S. Hydrogen bonds for **5i** [Å and °].

D-H...A	d(D-H)	d(H...A)	d(D...A)	<(DHA)
C(2)-H(2)...Cl(1)	0.93	2.58	3.222(14)	127.0
C(3)-H(3)...O(1)#2	0.93	2.66	3.455(14)	143.9
C(5)-H(5)...Cl(2)#3	0.93	2.84	3.676(13)	149.9
C(7)-H(7)...Cl(2)#3	0.93	2.79	3.667(12)	156.7
C(14)-H(14A)...Cl(1)	0.97	2.88	3.716(15)	145.0
C(14)-H(14A)...N(2)	0.97	2.69	3.241(18)	116.8

Symmetry transformations used to generate equivalent atoms:

#1 -x+3/2,-y+1/2,-z+2   #2 x,y-1,z   #3 -x+1,-y+1,-z+1

Table 18S. Crystal data and structure refinement for **6b** (CCDC 2017203).

Parameter	**6b** (CCDC 2017203)	
Empirical formula	$C_{34}H_{23}ClCuN_8O_6Se_2$	
Formula weight	904.51	
Temperature	293(2) *K*	
Wavelength	1.54186 Å	
Crystal system	Monoclinic	
Space group	P 21/c	
Unit cell dimensions	*a* = 20.283(2) Å<br>*b* = 11.7660(10) Å<br>*c* = 16.376(10) Å	*α* = 90°<br>*β* = 104.470(10)°<br>*γ* = 90°
Volume	3784(2) Å	
Z	4	
Density (calculated)	1.588 Mg/m^3	
Absorption coefficient	4.137 mm^{-1}	
F(000)	1796	
Theta range for data collection.	4.381 to 66.429°	
Index ranges	-22≤h≤24<br>-13≤k≤9<br>-19≤l≤19	
Reflections collected	34924	
Independent reflections	6523 [$R_{(int)}$ = 0.3224]	
Completeness to theta = 67.429°	98.3 %	
Refinement method	Full-matrix least-squares on F^2	
Data / restraints / parameters	6523 / 7 / 480	
Goodness-of-fit on F^2	0.733	
Final R indices [*I*>2sigma(*I*)]	R_1 = 0.0818, wR_2 = 0.1609	
R indices (all data)	R_1 = 0.3000, wR_2 = 0.2125	
Extinction coefficient	0.00049(7)	
Largest diff. peak and hole	0.589 and -0.469 e.E Å^{-3}	

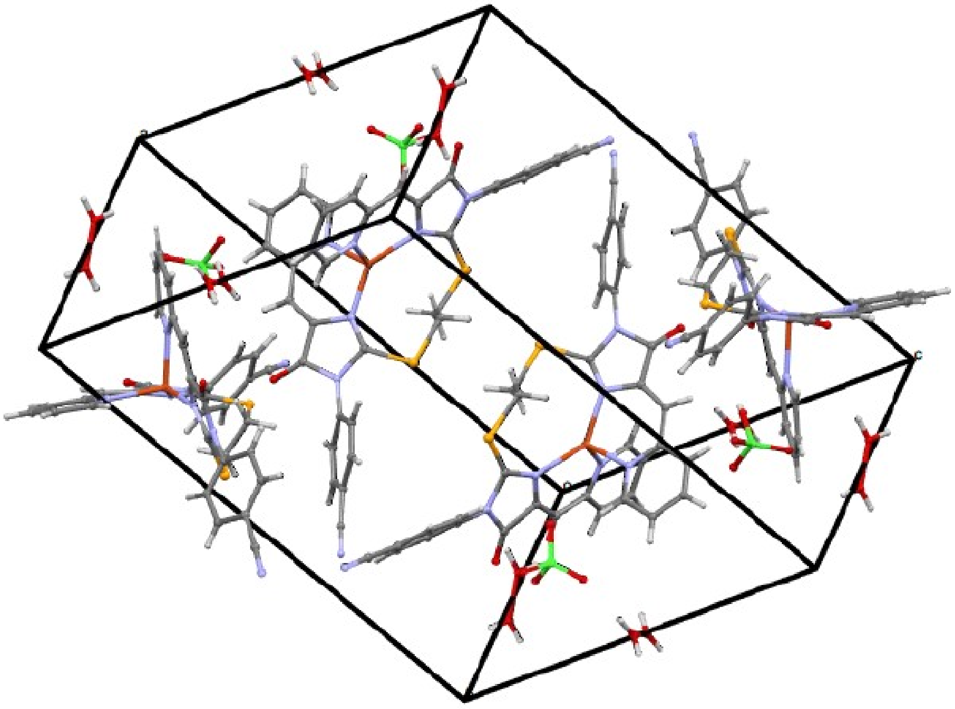

Table 19S. Bond lengths [E] and angles [°] for **6b**.

Se(1A)-C(10A)	1.882(14)
Se(1A)-C(18A)	1.987(10)
O(1A)-C(9A)	1.230(15)
N(1A)-C(2A)	1.307(15)
N(1A)-C(6A)	1.412(16)
N(1A)-Cu(1)	2.110(11)
N(2A)-C(10A)	1.264(15)
N(2A)-C(8A)	1.423(16)
N(2A)-Cu(1)	2.046(13)
N(3A)-C(11A)	1.421(15)
N(3A)-C(9A)	1.436(17)
N(3A)-C(10A)	1.477(18)
N(4A)-C(17A)	1.106(18)
C(2A)-C(3A)	1.410(19)
C(2A)-H(2A)	0.9300
C(3A)-C(4A)	1.41(2)
C(3A)-H(3A)	0.9300
C(4A)-C(5A)	1.295(19)
C(4A)-H(4A)	0.9300
C(5A)-C(6A)	1.40(2)
C(5A)-H(5A)	0.9300
C(6A)-C(7A)	1.415(18)
C(7A)-C(8A)	1.340(18)
C(7A)-H(7A)	0.9300
C(8A)-C(9A)	1.483(18)
C(11A)-C(16A)	1.397(16)
C(11A)-C(12A)	1.422(18)
C(12A)-C(13A)	1.353(18)
C(12A)-H(12A)	0.9300
C(13A)-C(14A)	1.364(19)
C(13A)-H(13A)	0.9300
C(14A)-C(15A)	1.394(19)
C(14A)-C(17A)	1.46(2)
C(15A)-C(16A)	1.330(17)
C(15A)-H(15A)	0.9300
C(16A)-H(16A)	0.9300
C(18A)-C(18B)	1.483(15)
C(18A)-H(18A)	0.9700
C(18A)-H(18B)	0.9700
Se(1B)-C(10B)	1.673(14)
Se(1B)-C(18B)	2.011(11)
O(1B)-C(9B)	1.207(15)
N(1B)-C(2B)	1.355(16)
N(1B)-C(6B)	1.38(2)
N(1B)-Cu(1)	2.049(13)
N(2B)-C(8B)	1.317(18)
N(2B)-C(10B)	1.362(15)
N(2B)-Cu(1)	2.130(13)
N(3B)-C(9B)	1.366(17)
N(3B)-C(11B)	1.441(15)
N(3B)-C(10B)	1.498(15)
N(4B)-C(17B)	1.143(18)
C(2B)-C(3B)	1.343(19)
C(2B)-H(2B)	0.9300
C(3B)-C(4B)	1.46(2)
C(3B)-H(3B)	0.9300
C(4B)-C(5B)	1.44(2)
C(4B)-H(4B)	0.9300
C(5B)-C(6B)	1.31(2)
C(5B)-H(5B)	0.9300
C(6B)-C(7B)	1.45(2)
C(7B)-C(8B)	1.28(2)
C(7B)-H(7B)	0.9300
C(8B)-C(9B)	1.546(19)
C(11B)-C(16B)	1.325(16)
C(11B)-C(12B)	1.368(16)
C(12B)-C(13B)	1.377(17)
C(12B)-H(12B)	0.9300
C(13B)-C(14B)	1.373(18)
C(13B)-H(13B)	0.9300
C(14B)-C(15B)	1.426(18)
C(14B)-C(17B)	1.47(2)
C(15B)-C(16B)	1.362(18)
C(15B)-H(15B)	0.9300
C(16B)-H(16B)	0.9300
C(18B)-H(18C)	0.9700
C(18B)-H(18D)	0.9700
Cl(1)-O(52)	1.336(9)
Cl(1)-O(54)	1.336(9)
Cl(1)-O(53)	1.342(9)
Cl(1)-O(51)	1.380(8)
OW-OW#1	1.78(6)
OW-H(1)	0.8600(11)
OW-H(11)	0.8600(11)
C(10A)-Se(1A)-C(18A)	98.8(8)
C(2A)-N(1A)-C(6A)	119.8(13)
C(2A)-N(1A)-Cu(1)	119.0(12)
C(6A)-N(1A)-Cu(1)	121.2(13)
C(10A)-N(2A)-C(8A)	110.2(14)
C(10A)-N(2A)-Cu(1)	133.1(12)
C(8A)-N(2A)-Cu(1)	115.1(13)
C(11A)-N(3A)-C(9A)	124.8(12)
C(11A)-N(3A)-C(10A)	130.5(14)
C(9A)-N(3A)-C(10A)	104.2(14)
N(1A)-C(2A)-C(3A)	121.8(14)
N(1A)-C(2A)-H(2A)	119.1
C(3A)-C(2A)-H(2A)	119.1
C(4A)-C(3A)-C(2A)	117.5(16)
C(4A)-C(3A)-H(3A)	121.2
C(2A)-C(3A)-H(3A)	121.2
C(5A)-C(4A)-C(3A)	121.0(18)
C(5A)-C(4A)-H(4A)	119.5
C(3A)-C(4A)-H(4A)	119.5
C(4A)-C(5A)-C(6A)	121.6(18)
C(4A)-C(5A)-H(5A)	119.2
C(6A)-C(5A)-H(5A)	119.2
C(5A)-C(6A)-N(1A)	118.2(17)
C(5A)-C(6A)-C(7A)	119.8(19)
N(1A)-C(6A)-C(7A)	122.0(16)
C(8A)-C(7A)-C(6A)	130.0(15)
C(8A)-C(7A)-H(7A)	115.0
C(6A)-C(7A)-H(7A)	115.0
C(7A)-C(8A)-N(2A)	131.4(16)
C(7A)-C(8A)-C(9A)	121.9(15)
N(2A)-C(8A)-C(9A)	106.6(15)
O(1A)-C(9A)-N(3A)	123.3(15)
O(1A)-C(9A)-C(8A)	130.8(17)
N(3A)-C(9A)-C(8A)	105.8(15)
N(2A)-C(10A)-N(3A)	112.8(14)
N(2A)-C(10A)-Se(1A)	132.0(15)
N(3A)-C(10A)-Se(1A)	114.4(13)
C(16A)-C(11A)-N(3A)	118.6(16)
C(16A)-C(11A)-C(12A)	120.3(15)
N(3A)-C(11A)-C(12A)	121.0(16)
C(13A)-C(12A)-C(11A)	119.9(16)
C(13A)-C(12A)-H(12A)	120.1
C(11A)-C(12A)-H(12A)	120.1
C(12A)-C(13A)-C(14A)	119.4(18)
C(12A)-C(13A)-H(13A)	120.3
C(14A)-C(13A)-H(13A)	120.3
C(13A)-C(14A)-C(15A)	119.9(18)
C(13A)-C(14A)-C(17A)	118.9(18)
C(15A)-C(14A)-C(17A)	121.0(17)
C(16A)-C(15A)-C(14A)	123.2(16)
C(16A)-C(15A)-H(15A)	118.4
C(14A)-C(15A)-H(15A)	118.4
C(15A)-C(16A)-C(11A)	117.1(14)
C(15A)-C(16A)-H(16A)	121.5
C(11A)-C(16A)-H(16A)	121.5
N(4A)-C(17A)-C(14A)	175.4(18)
C(18B)-C(18A)-Se(1A)	108.1(7)
C(18B)-C(18A)-H(18A)	110.1
Se(1A)-C(18A)-H(18A)	110.1
C(18B)-C(18A)-H(18B)	110.1
Se(1A)-C(18A)-H(18B)	110.1
H(18A)-C(18A)-H(18B)	108.4
C(10B)-Se(1B)-C(18B)	102.2(7)
C(2B)-N(1B)-C(6B)	121.7(15)
C(2B)-N(1B)-Cu(1)	119.0(13)
C(6B)-N(1B)-Cu(1)	118.8(13)

C(8B)-N(2B)-C(10B)	117.3(13)
C(8B)-N(2B)-Cu(1)	109.2(12)
C(10B)-N(2B)-Cu(1)	133.4(10)
C(9B)-N(3B)-C(11B)	124.4(12)
C(9B)-N(3B)-C(10B)	114.6(15)
C(11B)-N(3B)-C(10B)	120.8(15)
C(3B)-C(2B)-N(1B)	122.3(15)
C(3B)-C(2B)-H(2B)	118.9
N(1B)-C(2B)-H(2B)	118.9
C(2B)-C(3B)-C(4B)	121.7(17)
C(2B)-C(3B)-H(3B)	119.1
C(4B)-C(3B)-H(3B)	119.1
C(5B)-C(4B)-C(3B)	108.4(15)
C(5B)-C(4B)-H(4B)	125.8
C(3B)-C(4B)-H(4B)	125.8
C(6B)-C(5B)-C(4B)	131(2)
C(6B)-C(5B)-H(5B)	114.4
C(4B)-C(5B)-H(5B)	114.4
C(5B)-C(6B)-N(1B)	115(2)
C(5B)-C(6B)-C(7B)	123(2)
N(1B)-C(6B)-C(7B)	122.8(15)
C(8B)-C(7B)-C(6B)	128.3(17)
C(8B)-C(7B)-H(7B)	115.9
C(6B)-C(7B)-H(7B)	115.9
C(7B)-C(8B)-N(2B)	138.6(18)

C(7B)-C(8B)-C(9B)	114.4(19)
N(2B)-C(8B)-C(9B)	106.9(14)
O(1B)-C(9B)-N(3B)	129.6(15)
O(1B)-C(9B)-C(8B)	129.5(18)
N(3B)-C(9B)-C(8B)	100.9(15)
N(2B)-C(10B)-N(3B)	99.8(12)
N(2B)-C(10B)-Se(1B)	132.6(13)
N(3B)-C(10B)-Se(1B)	127.4(13)
C(16B)-C(11B)-C(12B)	126.8(15)
C(16B)-C(11B)-N(3B)	117.8(16)
C(12B)-C(11B)-N(3B)	115.4(15)
C(11B)-C(12B)-C(13B)	116.8(14)
C(11B)-C(12B)-H(12B)	121.6
C(13B)-C(12B)-H(12B)	121.6
C(14B)-C(13B)-C(12B)	119.5(15)
C(14B)-C(13B)-H(13B)	120.2
C(12B)-C(13B)-H(13B)	120.2
C(13B)-C(14B)-C(15B)	120.1(15)
C(13B)-C(14B)-C(17B)	125.0(16)
C(15B)-C(14B)-C(17B)	114.9(18)
C(16B)-C(15B)-C(14B)	119.6(14)
C(16B)-C(15B)-H(15B)	120.2
C(14B)-C(15B)-H(15B)	120.2
C(11B)-C(16B)-C(15B)	117.1(15)
C(11B)-C(16B)-H(16B)	121.5

C(15B)-C(16B)-H(16B)	121.5
N(4B)-C(17B)-C(14B)	177.9(19)
C(18A)-C(18B)-Se(1B)	108.9(7)
C(18A)-C(18B)-H(18C)	109.9
Se(1B)-C(18B)-H(18C)	109.9
C(18A)-C(18B)-H(18D)	109.9
Se(1B)-C(18B)-H(18D)	109.9
H(18C)-C(18B)-H(18D)	108.3
N(2A)-Cu(1)-N(1B)	107.6(6)
N(2A)-Cu(1)-N(1A)	96.0(6)
N(1B)-Cu(1)-N(1A)	102.9(4)
N(2A)-Cu(1)-N(2B)	138.4(6)
N(1B)-Cu(1)-N(2B)	97.4(6)
N(1A)-Cu(1)-N(2B)	110.4(5)
O(52)-Cl(1)-O(54)	95.1(15)
O(52)-Cl(1)-O(53)	109.1(16)
O(54)-Cl(1)-O(53)	111.5(14)
O(52)-Cl(1)-O(51)	112.7(13)
O(54)-Cl(1)-O(51)	107.5(11)
O(53)-Cl(1)-O(51)	118.5(11)
OW#1-OW-H(1)	135(6)
OW#1-OW-H(11)	120(6)
H(1)-OW-H(11)	104.6(11)

Symmetry transformations used to generate equivalent atoms:

#1 -x,-y,-z+1

Table 20S. Torsion angles [°] for **6b**.

C(6A)-N(1A)-C(2A)-C(3A)	-4(2)
Cu(1)-N(1A)-C(2A)-C(3A)	177.1(12)
N(1A)-C(2A)-C(3A)-C(4A)	3(2)
C(2A)-C(3A)-C(4A)-C(5A)	-1(3)
C(3A)-C(4A)-C(5A)-C(6A)	0(3)
C(4A)-C(5A)-C(6A)-N(1A)	0(3)
C(4A)-C(5A)-C(6A)-C(7A)	-179.9(19)
C(2A)-N(1A)-C(6A)-C(5A)	2(2)
Cu(1)-N(1A)-C(6A)-C(5A)	-178.9(12)
C(2A)-N(1A)-C(6A)-C(7A)	-178.1(14)
Cu(1)-N(1A)-C(6A)-C(7A)	1.1(18)
C(5A)-C(6A)-C(7A)-C(8A)	-167.4(18)
N(1A)-C(6A)-C(7A)-C(8A)	13(3)
C(6A)-C(7A)-C(8A)-N(2A)	-4(3)
C(6A)-C(7A)-C(8A)-C(9A)	176.4(14)
C(10A)-N(2A)-C(8A)-C(7A)	176.4(17)
Cu(1)-N(2A)-C(8A)-C(7A)	-16(2)
C(10A)-N(2A)-C(8A)-C(9A)	-4.0(16)
Cu(1)-N(2A)-C(8A)-C(9A)	163.8(8)
C(11A)-N(3A)-C(9A)-O(1A)	6(2)
C(10A)-N(3A)-C(9A)-O(1A)	178.7(12)
C(11A)-N(3A)-C(9A)-C(8A)	-178.2(12)
C(10A)-N(3A)-C(9A)-C(8A)	-5.9(12)
C(7A)-C(8A)-C(9A)-O(1A)	1(2)
N(2A)-C(8A)-C(9A)-O(1A)	-178.8(13)
C(7A)-C(8A)-C(9A)-N(3A)	-174.2(14)
N(2A)-C(8A)-C(9A)-N(3A)	6.2(13)
C(8A)-N(2A)-C(10A)-N(3A)	0.1(17)
Cu(1)-N(2A)-C(10A)-N(3A)	-164.7(9)
C(8A)-N(2A)-C(10A)-Se(1A)	-168.2(10)
Cu(1)-N(2A)-C(10A)-Se(1A)	27(2)
C(11A)-N(3A)-C(10A)-N(2A)	175.6(14)
C(9A)-N(3A)-C(10A)-N(2A)	3.8(16)
C(11A)-N(3A)-C(10A)-Se(1A)	-13.9(18)
C(9A)-N(3A)-C(10A)-Se(1A)	174.3(9)
C(18A)-Se(1A)-C(10A)-N(2A)	4.7(17)
C(18A)-Se(1A)-C(10A)-N(3A)	-163.5(10)
C(9A)-N(3A)-C(11A)-C(16A)	119.4(14)
C(10A)-N(3A)-C(11A)-C(16A)	-51(2)
C(9A)-N(3A)-C(11A)-C(12A)	-62.3(18)
C(10A)-N(3A)-C(11A)-C(12A)	127.4(14)
C(16A)-C(11A)-C(12A)-C(13A)	-4(2)
N(3A)-C(11A)-C(12A)-C(13A)	177.8(14)
C(11A)-C(12A)-C(13A)-C(14A)	1(2)
C(12A)-C(13A)-C(14A)-C(15A)	2(3)
C(12A)-C(13A)-C(14A)-C(17A)	-172.8(14)
C(13A)-C(14A)-C(15A)-C(16A)	0(3)
C(17A)-C(14A)-C(15A)-C(16A)	174.1(15)
C(14A)-C(15A)-C(16A)-C(11A)	-3(2)
N(3A)-C(11A)-C(16A)-C(15A)	-176.5(14)
C(12A)-C(11A)-C(16A)-C(15A)	5(2)
C(6B)-N(1B)-C(2B)-C(3B)	1(2)
Cu(1)-N(1B)-C(2B)-C(3B)	172.1(12)

N(1B)-C(2B)-C(3B)-C(4B)	-4(2)
C(2B)-C(3B)-C(4B)-C(5B)	4(2)
C(3B)-C(4B)-C(5B)-C(6B)	-3(3)
C(4B)-C(5B)-C(6B)-N(1B)	0(3)
C(4B)-C(5B)-C(6B)-C(7B)	179.0(15)
C(2B)-N(1B)-C(6B)-C(5B)	1(2)
Cu(1)-N(1B)-C(6B)-C(5B)	-170.4(11)
C(2B)-N(1B)-C(6B)-C(7B)	-177.9(12)
Cu(1)-N(1B)-C(6B)-C(7B)	10.7(16)
C(5B)-C(6B)-C(7B)-C(8B)	-174.6(17)
N(1B)-C(6B)-C(7B)-C(8B)	4(2)
C(6B)-C(7B)-C(8B)-N(2B)	0(3)
C(6B)-C(7B)-C(8B)-C(9B)	175.7(12)
C(10B)-N(2B)-C(8B)-C(7B)	167.7(17)
Cu(1)-N(2B)-C(8B)-C(7B)	-16(2)
C(10B)-N(2B)-C(8B)-C(9B)	-7.8(14)
Cu(1)-N(2B)-C(8B)-C(9B)	168.1(7)
C(11B)-N(3B)-C(9B)-O(1B)	5(2)
C(10B)-N(3B)-C(9B)-O(1B)	-179.9(12)
C(11B)-N(3B)-C(9B)-C(8B)	-174.1(12)
C(10B)-N(3B)-C(9B)-C(8B)	1.4(12)
C(7B)-C(8B)-C(9B)-O(1B)	8(2)
N(2B)-C(8B)-C(9B)-O(1B)	-175.3(13)
C(7B)-C(8B)-C(9B)-N(3B)	-173.4(12)
N(2B)-C(8B)-C(9B)-N(3B)	3.4(13)
C(8B)-N(2B)-C(10B)-N(3B)	8.1(13)
Cu(1)-N(2B)-C(10B)-N(3B)	-166.5(8)
C(8B)-N(2B)-C(10B)-Se(1B)	-167.6(10)
Cu(1)-N(2B)-C(10B)-Se(1B)	17.8(18)
C(9B)-N(3B)-C(10B)-N(2B)	-5.5(12)
C(11B)-N(3B)-C(10B)-N(2B)	170.3(11)
C(9B)-N(3B)-C(10B)-Se(1B)	170.6(9)
C(11B)-N(3B)-C(10B)-Se(1B)	-13.7(16)
C(18B)-Se(1B)-C(10B)-N(2B)	12.5(13)
C(18B)-Se(1B)-C(10B)-N(3B)	-162.2(9)
C(9B)-N(3B)-C(11B)-C(16B)	129.5(15)
C(10B)-N(3B)-C(11B)-C(16B)	-45.8(19)
C(9B)-N(3B)-C(11B)-C(12B)	-50.6(18)
C(10B)-N(3B)-C(11B)-C(12B)	134.1(12)
C(16B)-C(11B)-C(12B)-C(13B)	0(2)
N(3B)-C(11B)-C(12B)-C(13B)	-179.7(14)
C(11B)-C(12B)-C(13B)-C(14B)	1(2)
C(12B)-C(13B)-C(14B)-C(15B)	1(2)
C(12B)-C(13B)-C(14B)-C(17B)	178.9(14)
C(13B)-C(14B)-C(15B)-C(16B)	-2(2)
C(17B)-C(14B)-C(15B)-C(16B)	179.0(15)
C(12B)-C(11B)-C(16B)-C(15B)	-2(3)
N(3B)-C(11B)-C(16B)-C(15B)	177.8(14)
C(14B)-C(15B)-C(16B)-C(11B)	3(2)
Se(1A)-C(18A)-C(18B)-Se(1B)	176.2(7)

Symmetry transformations used to generate equivalent atoms:

#1 -x,-y,-z+1

Table 21S. Hydrogen bonds for **6b** [E and °].

D-H...A	d(D-H)	d(H...A)	d(D...A)	<(DHA)
C(12A)-H(12A)...N(4A)#2	0.93	2.67	3.47(2)	144.5
C(16A)-H(16A)...O(54)	0.93	2.53	3.30(2)	141.3
C(18A)-H(18A)...N(4A)#3	0.97	2.49	3.20(2)	130.4
C(18A)-H(18B)...N(2B)	0.97	2.45	3.171(19)	131.3
C(16B)-H(16B)...O(51)#4	0.93	2.54	3.32(2)	140.7
C(18B)-H(18D)...N(2A)	0.97	2.50	3.232(17)	131.8
OW-H(11)...O(53)	0.8600(11)	2.54(6)	3.02(3)	116(5)

Symmetry transformations used to generate equivalent atoms:
#1 -x,-y,-z+1 #2 -x+1,y+1/2,-z+3/2 #3 -x+1,-y,-z+1 #4 x,y+1,z

Table 22S. Crystal data and structure refinement for **6c** (CCDC 2125345).

Parameter	**6c** (CCDC 2125345)	
Empirical formula	$C_{34}H_{21}Cl_3CuN_7O_6Se_2$	
Formula weight	951.39	
Temperature	293(2) *K*	
Wavelength	1.54186 Å	
Crystal system	Monoclinic	
Space group	P 21/c	
Unit cell dimensions	*a* = 8.1846(4) Å<br>*b* = 13.6156(7) Å<br>*c* = 33.9630(10) Å	*α* = 90°<br>*β* = 93.899(4)°<br>*γ* = 90°
Volume	3776.0(3) Å	
Z	4	
Density (calculated)	1.647 Mg/m^3	
Absorption coefficient	5.434 mm^{-1}	
F(000)	1880	
Theta range for data collection.	2,608 to 66.780°	
Index ranges	-9≤h≤4<br>-16≤k≤16<br>-40≤l≤40	
Reflections collected	12034	
Independent reflections	12034 [$R_{(int)}$ = 0.1724]	
Completeness to theta = 66.780°	83.8 %	
Refinement method	Full-matrix least-squares on F^2	
Data / restraints / parameters	12034 / 18 / 480	
Goodness-of-fit on F^2	0.837	
Final R indices [*I*>2sigma(*I*)]	R_1 = 0.0931, wR_2 = 0.2189	
R indices (all data)	R_1 = 0.1701, wR_2 = 0.2448	
Extinction coefficient	n/a	
Largest diff. peak and hole	1.354 and -1.131 e.E $Å^{-3}$	

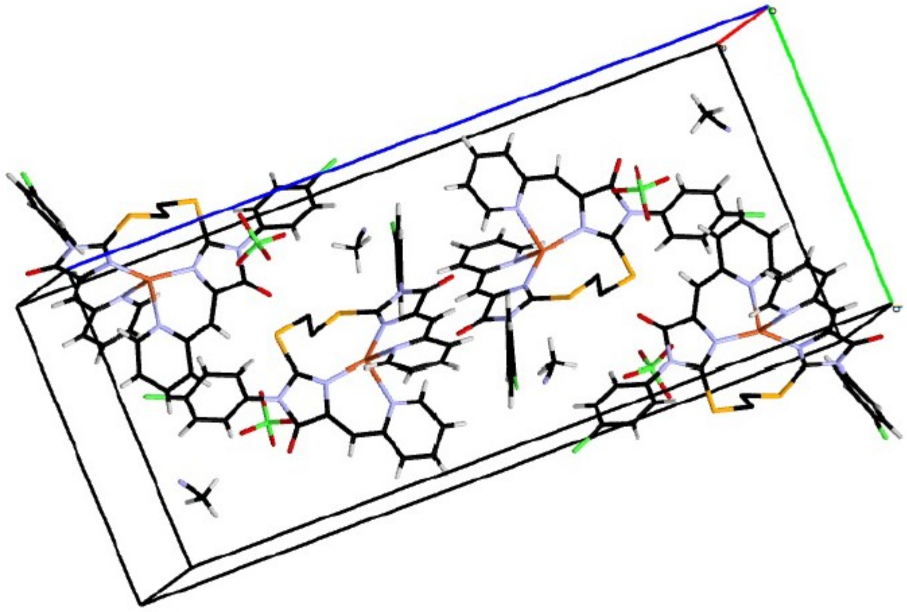

Table 23S. Bond lengths [Å] and angles [°] for **6c**.

Cu(1)-N(1A)	1.971(15)
Cu(1)-N(1B)	1.982(11)
Cu(1)-N(3B)	2.116(15)
Cu(1)-N(3A)	2.123(15)
Se(1A)-C(2A)	1.83(2)
Se(1A)-C(17A)	1.890(16)
Cl(2A)-C(14A)	1.72(2)
O(1A)-C(3A)	1.183(15)
N(1A)-C(2A)	1.37(2)
N(1A)-C(4A)	1.455(17)
N(2A)-C(3A)	1.41(2)
N(2A)-C(2A)	1.418(17)
N(2A)-C(11A)	1.47(2)
N(3A)-C(6A)	1.304(18)
N(3A)-C(10A)	1.34(2)
C(3A)-C(4A)	1.48(2)
C(4A)-C(5A)	1.33(2)
C(5A)-C(6A)	1.48(2)
C(5A)-H(5A)	0.9300
C(6A)-C(7A)	1.39(3)
C(7A)-C(8A)	1.33(2)
C(7A)-H(7A)	0.9300
C(8A)-C(9A)	1.33(2)
C(8A)-H(8A)	0.9300
C(9A)-C(10A)	1.40(3)
C(9A)-H(9A)	0.9300
C(10A)-H(10A)	0.9300
C(11A)-C(16A)	1.37(2)
C(11A)-C(12A)	1.37(3)
C(12A)-C(13A)	1.39(3)
C(12A)-H(12A)	0.9300
C(13A)-C(14A)	1.32(3)
C(13A)-H(13A)	0.9300
C(14A)-C(15A)	1.45(3)
C(15A)-C(16A)	1.43(3)
C(15A)-H(15A)	0.9300
C(16A)-H(16A)	0.9300
C(17A)-C(17B)	1.55(3)
Cl(1)-O(3)	1.406(12)
Cl(1)-O(4)	1.430(16)
Cl(1)-O(5)	1.447(15)
Cl(1)-O(2)	1.450(14)
Se(1B)-C(2B)	1.889(17)
Se(1B)-C(17B)	1.942(16)
Cl(2B)-C(14B)	1.744(16)
O(1B)-C(3B)	1.231(18)
N(1B)-C(2B)	1.316(17)
N(1B)-C(4B)	1.52(2)
N(2B)-C(2B)	1.338(17)
N(2B)-C(3B)	1.40(2)
N(2B)-C(11B)	1.445(18)
N(3B)-C(10B)	1.316(17)
N(3B)-C(6B)	1.38(2)
C(3B)-C(4B)	1.412(19)

C(4B)-C(5B)	1.28(2)
C(5B)-C(6B)	1.44(2)
C(5B)-H(5B)	0.9300
C(6B)-C(7B)	1.45(2)
C(7B)-C(8B)	1.36(2)
C(7B)-H(7B)	0.9300
C(8B)-C(9B)	1.41(3)
C(8B)-H(8B)	0.9300
C(9B)-C(10B)	1.35(3)
C(9B)-H(9B)	0.9300
C(10B)-H(10B)	0.9300
C(11B)-C(12B)	1.37(2)
C(11B)-C(16B)	1.40(2)
C(12B)-C(13B)	1.40(2)
C(12B)-H(12B)	0.9300
C(13B)-C(14B)	1.33(3)
C(13B)-H(13B)	0.9300
C(14B)-C(15B)	1.36(3)
C(15B)-C(16B)	1.37(2)
C(15B)-H(15B)	0.9300
C(16B)-H(16B)	0.9300
N(5)-C(18)	1.16(3)
C(18)-C(19)	1.44(4)
C(19)-H(19A)	0.9600
C(19)-H(19B)	0.9600
C(19)-H(19C)	0.9600
N(1A)-Cu(1)-N(1B)	143.7(6)
N(1A)-Cu(1)-N(3B)	107.4(5)
N(1B)-Cu(1)-N(3B)	97.8(5)
N(1A)-Cu(1)-N(3A)	93.1(6)
N(1B)-Cu(1)-N(3A)	112.3(6)
N(3B)-Cu(1)-N(3A)	91.6(6)
C(2A)-Se(1A)-C(17A)	99.3(7)
C(2A)-N(1A)-C(4A)	105.3(13)
C(2A)-N(1A)-Cu(1)	135.8(11)
C(4A)-N(1A)-Cu(1)	118.2(12)
C(3A)-N(2A)-C(2A)	109.4(14)
C(3A)-N(2A)-C(11A)	125.9(12)
C(2A)-N(2A)-C(11A)	124.6(15)
C(6A)-N(3A)-C(10A)	116.9(16)
C(6A)-N(3A)-Cu(1)	121.1(12)
C(10A)-N(3A)-Cu(1)	116.9(12)
N(1A)-C(2A)-N(2A)	111.8(15)
N(1A)-C(2A)-Se(1A)	129.1(11)
N(2A)-C(2A)-Se(1A)	118.9(15)
O(1A)-C(3A)-N(2A)	125.6(15)
O(1A)-C(3A)-C(4A)	130.3(16)
N(2A)-C(3A)-C(4A)	104.0(11)
C(5A)-C(4A)-N(1A)	128.0(15)
C(5A)-C(4A)-C(3A)	122.6(13)
N(1A)-C(4A)-C(3A)	109.4(15)
C(4A)-C(5A)-C(6A)	129.5(14)
C(4A)-C(5A)-H(5A)	115.2
C(6A)-C(5A)-H(5A)	115.2

N(3A)-C(6A)-C(7A)	123.9(16)
N(3A)-C(6A)-C(5A)	119.3(18)
C(7A)-C(6A)-C(5A)	116.8(14)
C(8A)-C(7A)-C(6A)	117.5(17)
C(8A)-C(7A)-H(7A)	121.3
C(6A)-C(7A)-H(7A)	121.3
C(7A)-C(8A)-C(9A)	121(2)
C(7A)-C(8A)-H(8A)	119.4
C(9A)-C(8A)-H(8A)	119.4
C(8A)-C(9A)-C(10A)	118.3(18)
C(8A)-C(9A)-H(9A)	120.9
C(10A)-C(9A)-H(9A)	120.9
N(3A)-C(10A)-C(9A)	121.3(14)
N(3A)-C(10A)-H(10A)	119.4
C(9A)-C(10A)-H(10A)	119.4
C(16A)-C(11A)-C(12A)	124(2)
C(16A)-C(11A)-N(2A)	119.2(19)
C(12A)-C(11A)-N(2A)	116.8(19)
C(11A)-C(12A)-C(13A)	119(2)
C(11A)-C(12A)-H(12A)	120.7
C(13A)-C(12A)-H(12A)	120.7
C(14A)-C(13A)-C(12A)	120(2)
C(14A)-C(13A)-H(13A)	120.0
C(12A)-C(13A)-H(13A)	120.0
C(13A)-C(14A)-C(15A)	123(2)
C(13A)-C(14A)-Cl(2A)	122(2)
C(15A)-C(14A)-Cl(2A)	115(2)
C(16A)-C(15A)-C(14A)	116(2)
C(16A)-C(15A)-H(15A)	122.2
C(14A)-C(15A)-H(15A)	122.2
C(11A)-C(16A)-C(15A)	118(2)
C(11A)-C(16A)-H(16A)	121.0
C(15A)-C(16A)-H(16A)	121.0
C(17B)-C(17A)-Se(1A)	113.4(12)
O(3)-Cl(1)-O(4)	107.6(11)
O(3)-Cl(1)-O(5)	115.5(13)
O(4)-Cl(1)-O(5)	103.5(11)
O(3)-Cl(1)-O(2)	111.9(10)
O(4)-Cl(1)-O(2)	107.1(11)
O(5)-Cl(1)-O(2)	110.5(10)
C(2B)-Se(1B)-C(17B)	99.3(8)
C(2B)-N(1B)-C(4B)	102.6(11)
C(2B)-N(1B)-Cu(1)	140.7(12)
C(4B)-N(1B)-Cu(1)	115.9(10)
C(2B)-N(2B)-C(3B)	106.7(11)
C(2B)-N(2B)-C(11B)	128.2(15)
C(3B)-N(2B)-C(11B)	125.1(12)
C(10B)-N(3B)-C(6B)	119.0(17)
C(10B)-N(3B)-Cu(1)	120.9(13)
C(6B)-N(3B)-Cu(1)	119.0(10)
N(1B)-C(2B)-N(2B)	116.9(15)
N(1B)-C(2B)-Se(1B)	124.9(11)
N(2B)-C(2B)-Se(1B)	118.3(11)

O(1B)-C(3B)-N(2B)	122.5(13)
O(1B)-C(3B)-C(4B)	129.2(19)
N(2B)-C(3B)-C(4B)	108.0(14)
C(5B)-C(4B)-C(3B)	125.8(18)
C(5B)-C(4B)-N(1B)	128.3(15)
C(3B)-C(4B)-N(1B)	105.8(15)
C(4B)-C(5B)-C(6B)	131.5(18)
C(4B)-C(5B)-H(5B)	114.3
C(6B)-C(5B)-H(5B)	114.3
N(3B)-C(6B)-C(5B)	123.9(17)
N(3B)-C(6B)-C(7B)	118.7(14)
C(5B)-C(6B)-C(7B)	117.3(15)
C(8B)-C(7B)-C(6B)	118.9(17)
C(8B)-C(7B)-H(7B)	120.5
C(6B)-C(7B)-H(7B)	120.5
C(7B)-C(8B)-C(9B)	120(2)
C(7B)-C(8B)-H(8B)	119.9
C(9B)-C(8B)-H(8B)	119.9
C(10B)-C(9B)-C(8B)	117.3(19)
C(10B)-C(9B)-H(9B)	121.3
C(8B)-C(9B)-H(9B)	121.3
N(3B)-C(10B)-C(9B)	126(2)
N(3B)-C(10B)-H(10B)	117.2
C(9B)-C(10B)-H(10B)	117.2
C(12B)-C(11B)-C(16B)	121.3(15)
C(12B)-C(11B)-N(2B)	120.1(15)
C(16B)-C(11B)-N(2B)	118.5(16)
C(11B)-C(12B)-C(13B)	116.9(17)
C(11B)-C(12B)-H(12B)	121.6
C(13B)-C(12B)-H(12B)	121.6
C(14B)-C(13B)-C(12B)	122(2)
C(14B)-C(13B)-H(13B)	118.9
C(12B)-C(13B)-H(13B)	118.9
C(13B)-C(14B)-C(15B)	120.4(17)
C(13B)-C(14B)-Cl(2B)	121(2)
C(15B)-C(14B)-Cl(2B)	118.7(17)
C(14B)-C(15B)-C(16B)	120.5(18)
C(14B)-C(15B)-H(15B)	119.7
C(16B)-C(15B)-H(15B)	119.7
C(15B)-C(16B)-C(11B)	118.5(18)
C(15B)-C(16B)-H(16B)	120.7
C(11B)-C(16B)-H(16B)	120.7
C(17A)-C(17B)-Se(1B)	114.6(12)
N(5)-C(18)-C(19)	177(4)
C(18)-C(19)-H(19A)	109.5
C(18)-C(19)-H(19B)	109.5
H(19A)-C(19)-H(19B)	109.5
C(18)-C(19)-H(19C)	109.5
H(19A)-C(19)-H(19C)	109.5
H(19B)-C(19)-H(19C)	109.5

Table 24S. Torsion angles [°] for **6c**.

C(4A)-N(1A)-C(2A)-N(2A)	-2(2)
Cu(1)-N(1A)-C(2A)-N(2A)	168.0(12)
C(4A)-N(1A)-C(2A)-Se(1A)	173.2(14)
Cu(1)-N(1A)-C(2A)-Se(1A)	-17(3)
C(3A)-N(2A)-C(2A)-N(1A)	3(2)
C(11A)-N(2A)-C(2A)-N(1A)	-179.0(17)
C(3A)-N(2A)-C(2A)-Se(1A)	-172.5(14)
C(11A)-N(2A)-C(2A)-Se(1A)	5(2)
C(17A)-Se(1A)-C(2A)-N(1A)	-6(2)
C(17A)-Se(1A)-C(2A)-N(2A)	169.0(14)
C(2A)-N(2A)-C(3A)-O(1A)	-179.7(19)
C(11A)-N(2A)-C(3A)-O(1A)	3(3)
C(2A)-N(2A)-C(3A)-C(4A)	-3.0(19)
C(11A)-N(2A)-C(3A)-C(4A)	179.4(16)
C(2A)-N(1A)-C(4A)-C(5A)	-179.3(19)
Cu(1)-N(1A)-C(4A)-C(5A)	8(3)
C(2A)-N(1A)-C(4A)-C(3A)	0(2)
Cu(1)-N(1A)-C(4A)-C(3A)	-172.0(11)
O(1A)-C(3A)-C(4A)-C(5A)	-2(3)
N(2A)-C(3A)-C(4A)-C(5A)	-178.7(18)
O(1A)-C(3A)-C(4A)-N(1A)	178(2)
N(2A)-C(3A)-C(4A)-N(1A)	1.7(19)
N(1A)-C(4A)-C(5A)-C(6A)	6(3)
C(3A)-C(4A)-C(5A)-C(6A)	-173.9(18)
C(10A)-N(3A)-C(6A)-C(7A)	-6(3)
Cu(1)-N(3A)-C(6A)-C(7A)	148.6(18)
C(10A)-N(3A)-C(6A)-C(5A)	173.3(19)
Cu(1)-N(3A)-C(6A)-C(5A)	-33(2)
C(4A)-C(5A)-C(6A)-N(3A)	9(3)
C(4A)-C(5A)-C(6A)-C(7A)	-172(2)
N(3A)-C(6A)-C(7A)-C(8A)	-2(4)
C(5A)-C(6A)-C(7A)-C(8A)	180(2)
C(6A)-C(7A)-C(8A)-C(9A)	2(4)
C(7A)-C(8A)-C(9A)-C(10A)	4(4)
C(6A)-N(3A)-C(10A)-C(9A)	12(3)
Cu(1)-N(3A)-C(10A)-C(9A)	-143.3(18)
C(8A)-C(9A)-C(10A)-N(3A)	-11(3)
C(3A)-N(2A)-C(11A)-C(16A)	62(2)
C(2A)-N(2A)-C(11A)-C(16A)	-115.4(19)
C(3A)-N(2A)-C(11A)-C(12A)	-117(2)
C(2A)-N(2A)-C(11A)-C(12A)	66(2)
C(16A)-C(11A)-C(12A)-C(13A)	5(3)
N(2A)-C(11A)-C(12A)-C(13A)	-175.8(17)
C(11A)-C(12A)-C(13A)-C(14A)	0(3)
C(12A)-C(13A)-C(14A)-C(15A)	-5(4)
C(12A)-C(13A)-C(14A)-Cl(2A)	179.4(16)
C(13A)-C(14A)-C(15A)-C(16A)	5(3)
Cl(2A)-C(14A)-C(15A)-C(16A)	-178.7(15)
C(12A)-C(11A)-C(16A)-C(15A)	-5(3)
N(2A)-C(11A)-C(16A)-C(15A)	176.4(15)
C(14A)-C(15A)-C(16A)-C(11A)	-1(3)
C(2A)-Se(1A)-C(17A)-C(17B)	-65.8(13)
C(4B)-N(1B)-C(2B)-N(2B)	-3(2)
Cu(1)-N(1B)-C(2B)-N(2B)	165.6(15)
C(4B)-N(1B)-C(2B)-Se(1B)	176.2(14)
Cu(1)-N(1B)-C(2B)-Se(1B)	-15(3)
C(3B)-N(2B)-C(2B)-N(1B)	2(2)
C(11B)-N(2B)-C(2B)-N(1B)	-178.9(18)
C(3B)-N(2B)-C(2B)-Se(1B)	-176.9(13)
C(11B)-N(2B)-C(2B)-Se(1B)	2(2)
C(17B)-Se(1B)-C(2B)-N(1B)	-11.9(19)
C(17B)-Se(1B)-C(2B)-N(2B)	167.3(15)
C(2B)-N(2B)-C(3B)-O(1B)	174.3(17)
C(11B)-N(2B)-C(3B)-O(1B)	-5(3)
C(2B)-N(2B)-C(3B)-C(4B)	0(2)
C(11B)-N(2B)-C(3B)-C(4B)	-179.2(16)
O(1B)-C(3B)-C(4B)-C(5B)	1(4)
N(2B)-C(3B)-C(4B)-C(5B)	176(2)
O(1B)-C(3B)-C(4B)-N(1B)	-175.5(19)
N(2B)-C(3B)-C(4B)-N(1B)	-1(2)
C(2B)-N(1B)-C(4B)-C(5B)	-174(2)
Cu(1)-N(1B)-C(4B)-C(5B)	14(3)
C(2B)-N(1B)-C(4B)-C(3B)	2(2)
Cu(1)-N(1B)-C(4B)-C(3B)	-169.6(12)
C(3B)-C(4B)-C(5B)-C(6B)	-177(2)
N(1B)-C(4B)-C(5B)-C(6B)	-1(4)
C(10B)-N(3B)-C(6B)-C(5B)	-179.8(19)
Cu(1)-N(3B)-C(6B)-C(5B)	-12(2)
C(10B)-N(3B)-C(6B)-C(7B)	3(3)
Cu(1)-N(3B)-C(6B)-C(7B)	171.7(14)
C(4B)-C(5B)-C(6B)-N(3B)	0(4)
C(4B)-C(5B)-C(6B)-C(7B)	176(2)
N(3B)-C(6B)-C(7B)-C(8B)	-3(3)
C(5B)-C(6B)-C(7B)-C(8B)	-179.9(18)
C(6B)-C(7B)-C(8B)-C(9B)	-2(3)
C(7B)-C(8B)-C(9B)-C(10B)	6(3)
C(6B)-N(3B)-C(10B)-C(9B)	1(3)
Cu(1)-N(3B)-C(10B)-C(9B)	-167(2)
C(8B)-C(9B)-C(10B)-N(3B)	-6(4)
C(2B)-N(2B)-C(11B)-C(12B)	59(2)
C(3B)-N(2B)-C(11B)-C(12B)	-122(2)
C(2B)-N(2B)-C(11B)-C(16B)	-123(2)
C(3B)-N(2B)-C(11B)-C(16B)	55(2)
C(16B)-C(11B)-C(12B)-C(13B)	3(3)
N(2B)-C(11B)-C(12B)-C(13B)	-179.8(16)
C(11B)-C(12B)-C(13B)-C(14B)	-3(3)
C(12B)-C(13B)-C(14B)-C(15B)	2(4)
C(12B)-C(13B)-C(14B)-Cl(2B)	179.3(16)
C(13B)-C(14B)-C(15B)-C(16B)	0(3)
Cl(2B)-C(14B)-C(15B)-C(16B)	-177.3(16)
C(14B)-C(15B)-C(16B)-C(11B)	-1(3)
C(12B)-C(11B)-C(16B)-C(15B)	-1(3)
N(2B)-C(11B)-C(16B)-C(15B)	-178.4(16)
Se(1A)-C(17A)-C(17B)-Se(1B)	-170.5(9)

Table 25S. Hydrogen bonds for **6c** [Å and °].

D-H...A	d(D-H)	d(H...A)	d(D...A)	<(DHA)
C(5A)-H(5A)...O(1A)#1	0.93	2.62	3.483(18)	155.5
C(7A)-H(7A)...O(1A)#1	0.93	2.54	3.39(2)	152.6
C(16A)-H(16A)...N(5)	0.93	2.53	3.19(3)	127.7
C(7B)-H(7B)...O(2)	0.93	2.65	3.50(2)	151.7
C(7B)-H(7B)...O(3)	0.93	2.41	3.25(3)	150.3
C(9B)-H(9B)...Cl(2A)#2	0.93	2.77	3.49(2)	134.8
C(19)-H(19C)...O(1B)#3	0.96	2.65	3.19(3)	116.2

Symmetry transformations used to generate equivalent atoms:

#1 -x+2,-y+1,-z+1 #2 -x+1,-y+1,-z+1 #3 -x+2,y+1/2,-z+1/2

UV-vis spectra

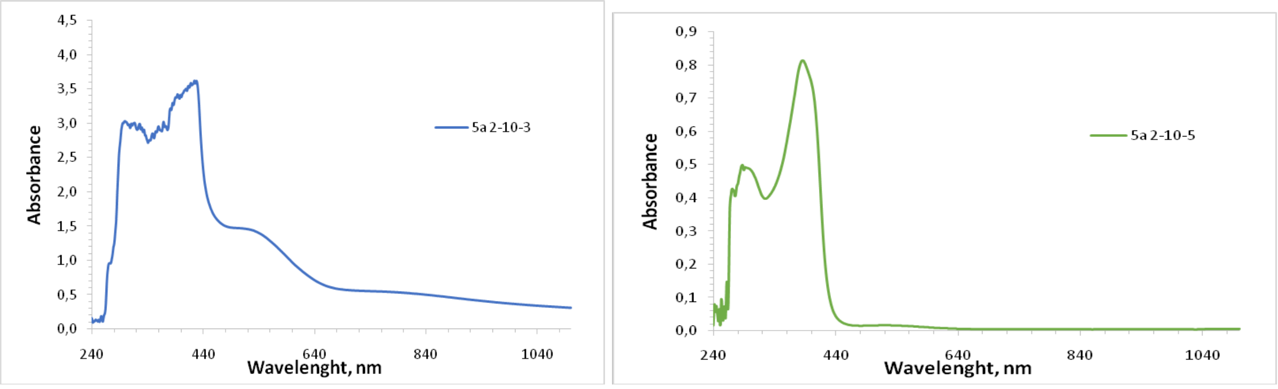

Figure 25S. Electronic spectra of coordination compound **5a** (DMF, $2\cdot10^{-3}$M (*left*), $2\cdot10^{-5}$M (*right*).

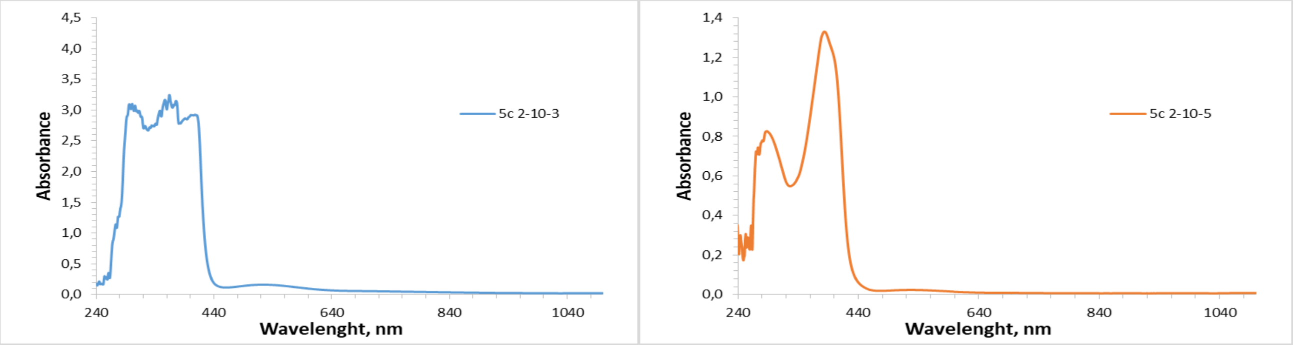

Figure 26S. Electronic spectra of coordination compound **5c** (DMF, $2\cdot10^{-3}$M (*left*), $2\cdot10^{-5}$M (*right*).

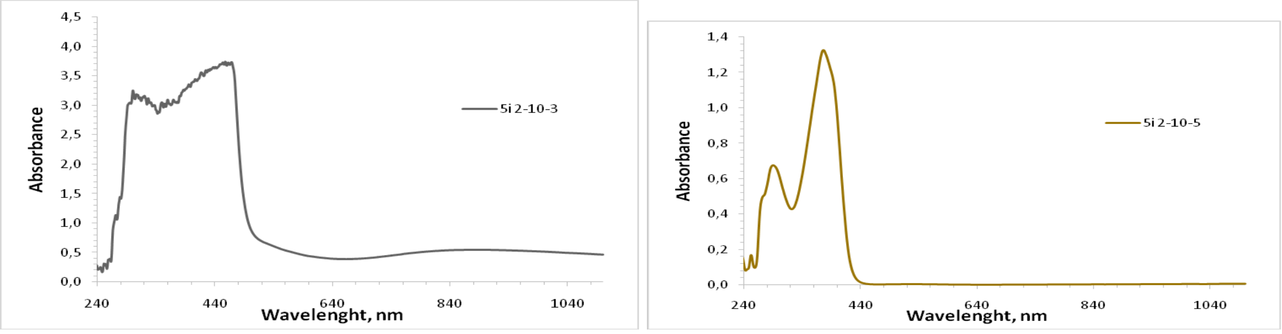

Figure 27S. Electronic spectra of coordination compound **5i** (DMF, $2\cdot10^{-3}$M (*left*), $2\cdot10^{-5}$M (*right*).

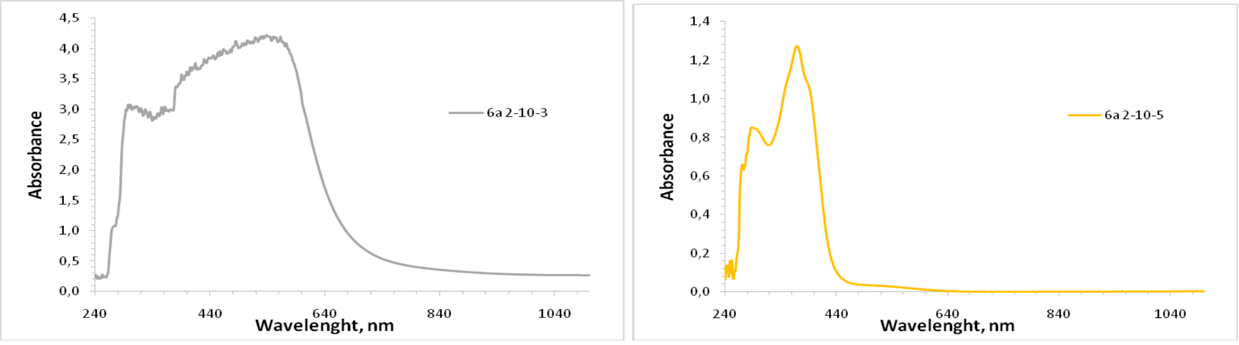

Figure 28S. Electronic spectra of coordination compound **6a** (DMF, $2\cdot10^{-3}$M (*left*), $2\cdot10^{-5}$M (*right*).

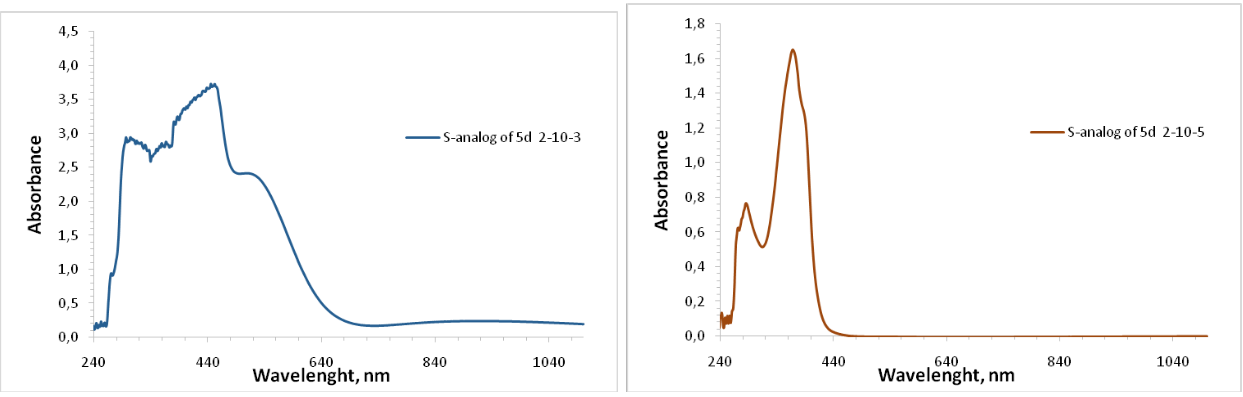

Figure 29S. Electronic spectra of S-analog of **5a** [A. G. Majouga, M. I. Zvereva, M. P. Rubtsova, D. A. Skvortsov, A. V. Mironov, D. M. Azhibek, O. O. Krasnovskaya, V. M. Gerasimov, A. V. Udina, N. I. Vorozhtsov, E. K. Beloglazkina, L. Agron, L.V. Mikhina, A. V. Tretyakova, N. V. Zyk, N. S. Zefirov, A. V. Kabanov, O. A. Dontsova, *J. Med. Chem.* 2014, 57, 6252-6258] (DMF, $2 \cdot 10^{-3}$M (*left*), $2 \cdot 10^{-5}$M (*right*).

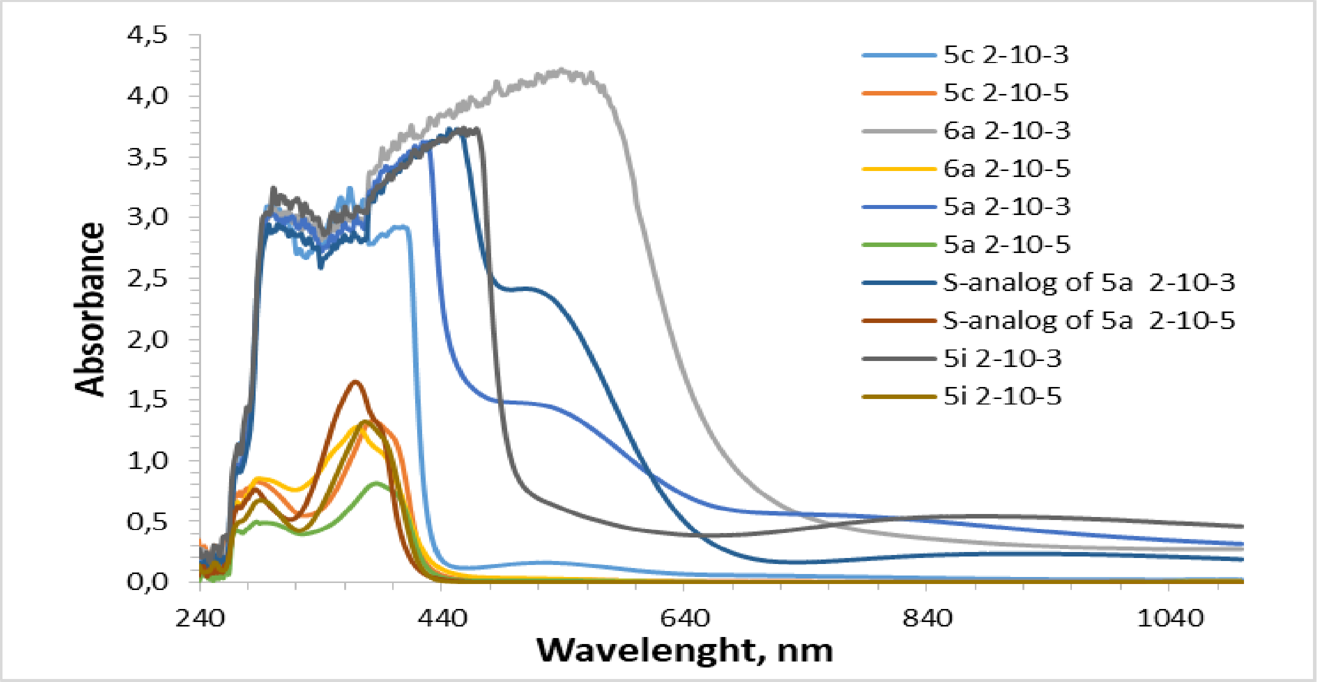

Figure 30S. Superposition of UV-vis spectra curves for complexes of different structural types (for details, see the legend in the figure).

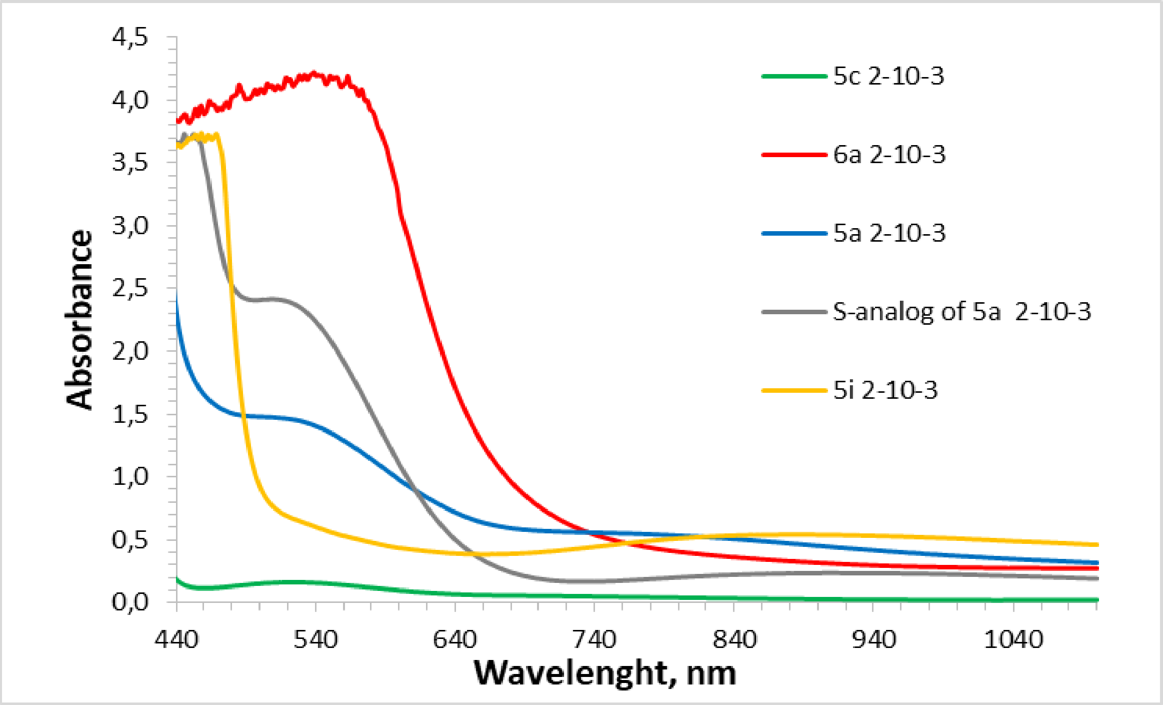

Figure 31S. Superposition of UV-vis spectra curves for complexes of different structural types in the visible region (for details, see the legend in the figure).

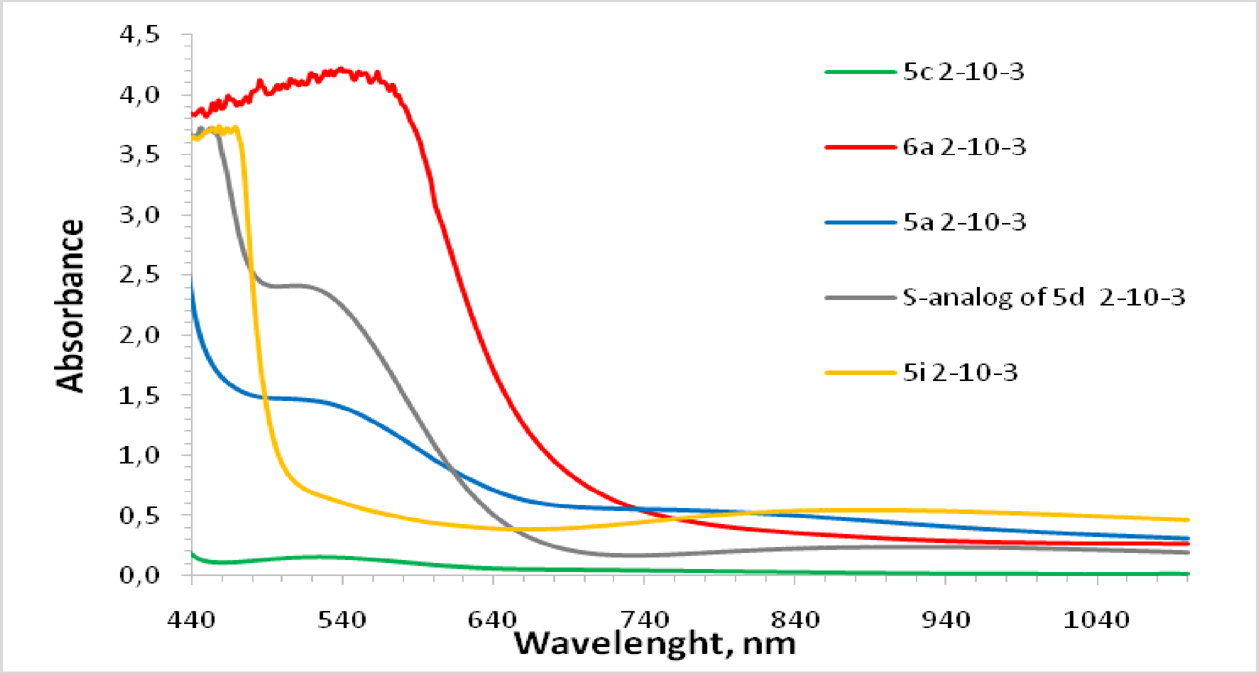

Figure 31S. Superposition of UV-vis spectra curves for complexes of different structural types in the UV-region (for deitals, see the legend in the figure).

EPR spectra

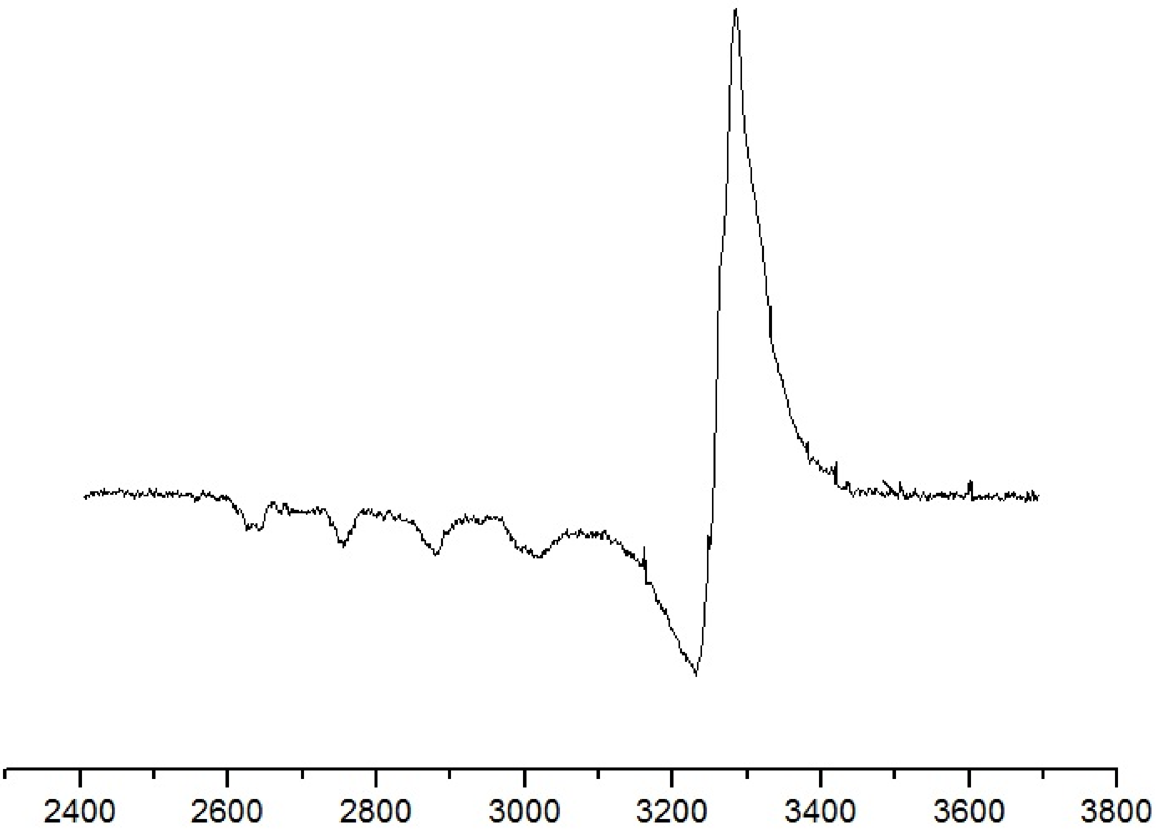

Figure 32S. EPR spectra for the complex **5a**.

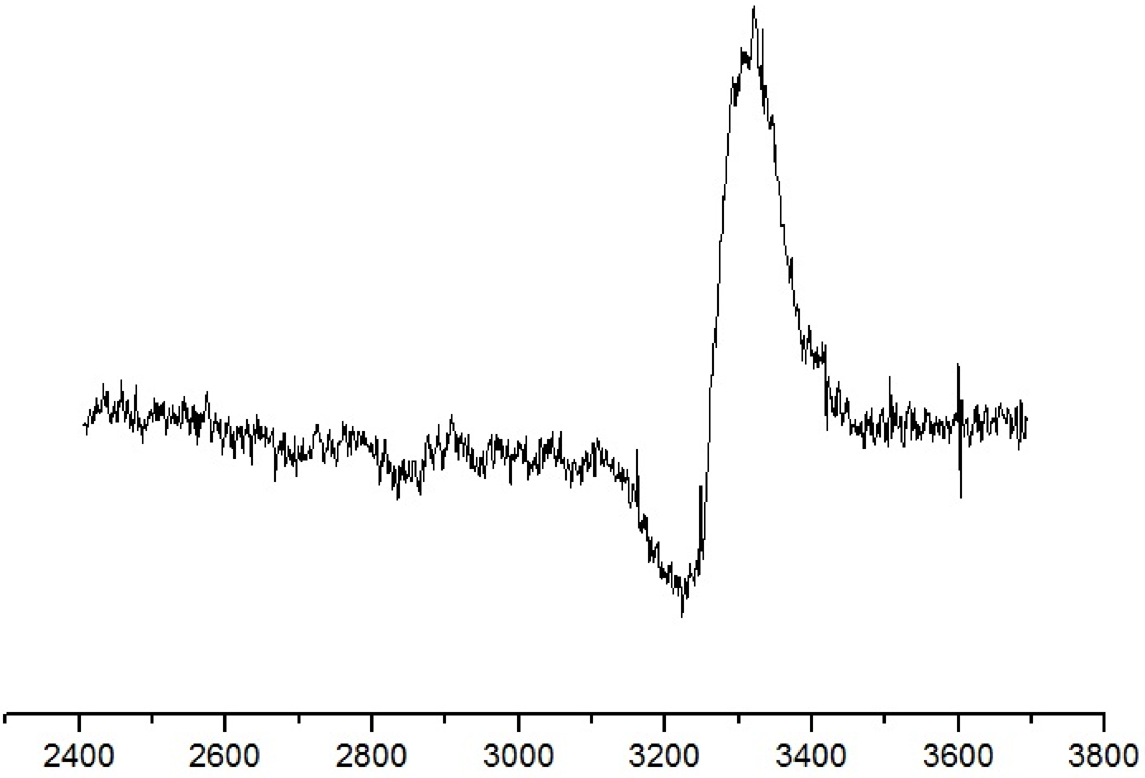

Figure 33S. EPR spectra for the complex **5c**.

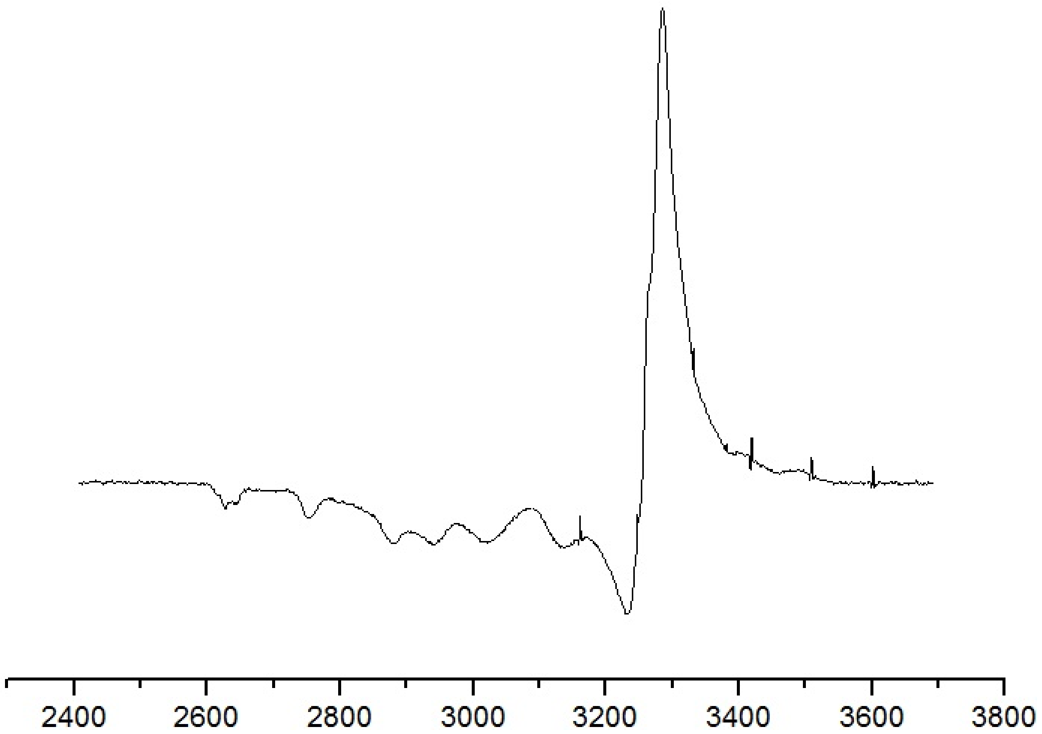

Figure 34S. EPR spectra for the complex **5i**.

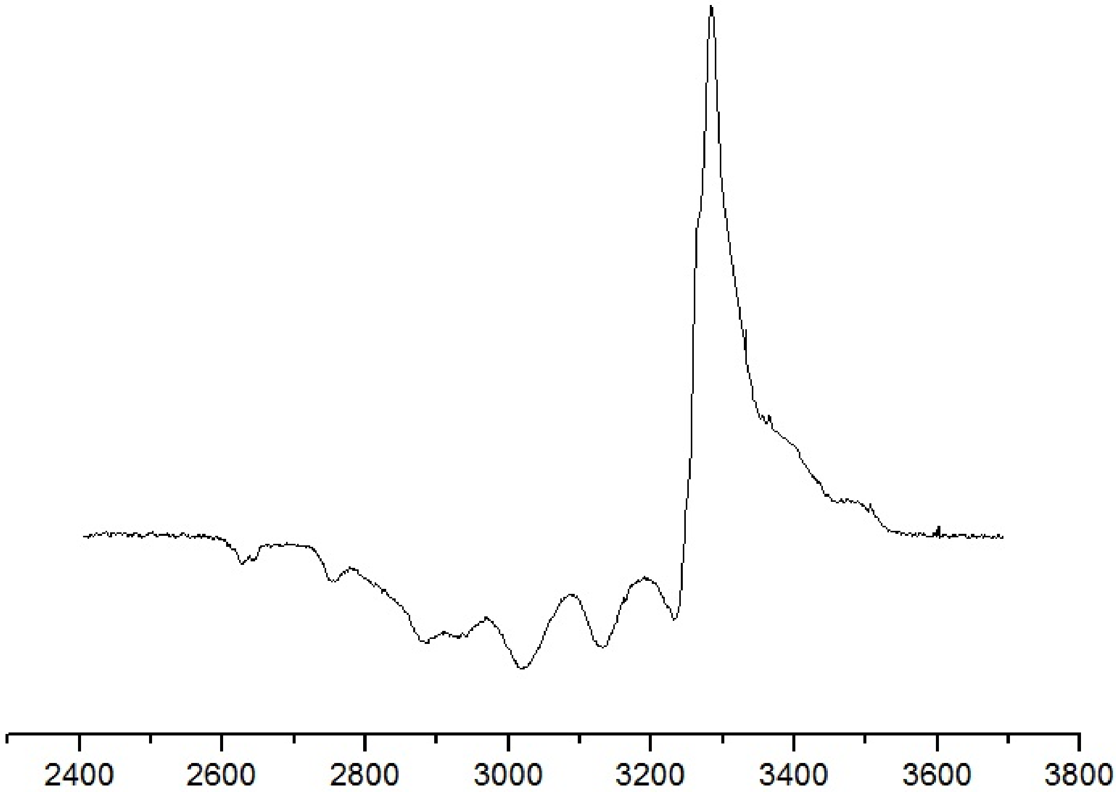

Figure 35S. EPR spectra for the S-analog of **5a**.

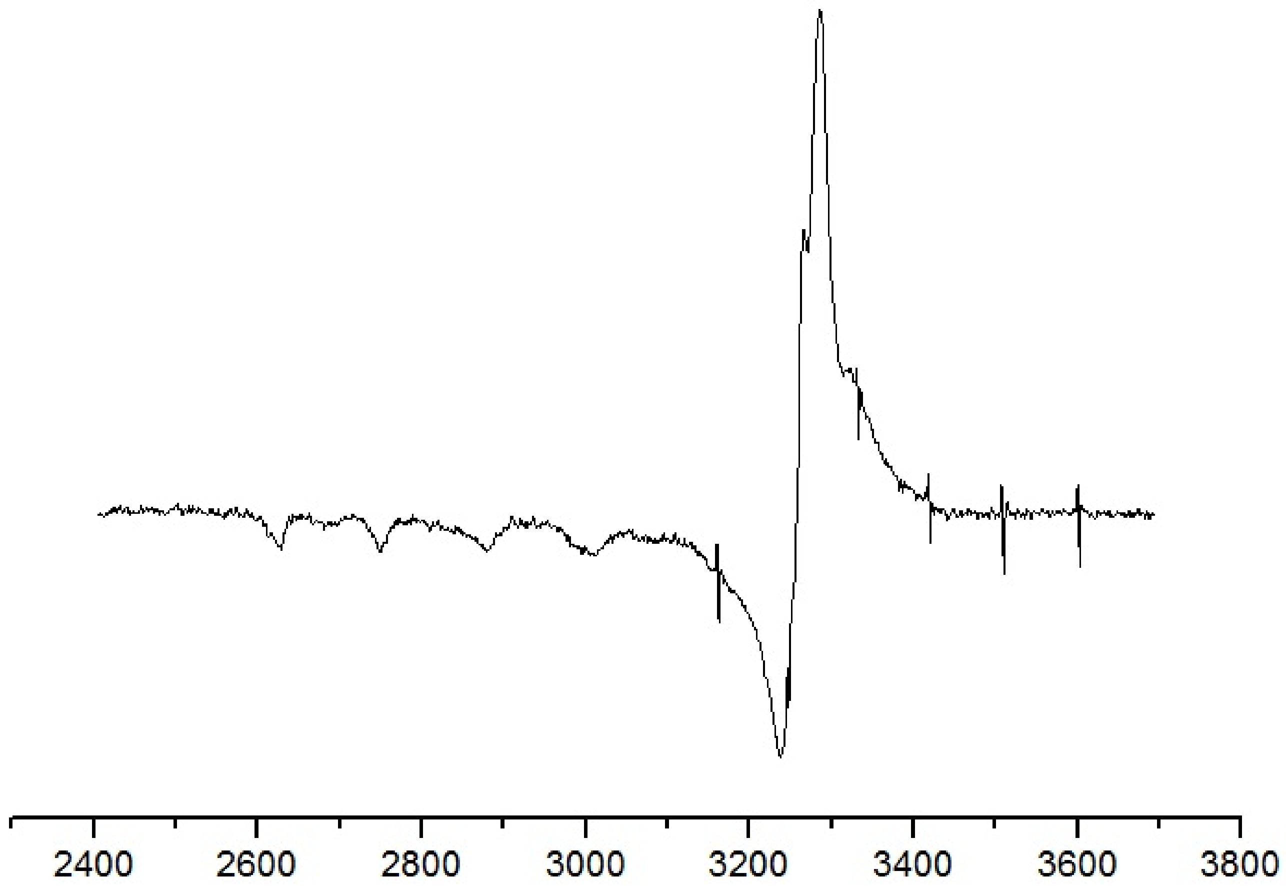

Figure 36S. EPR spectra for the complex **6a**.
